# Molecular laterality encodes stress susceptibility in the medial prefrontal cortex

**DOI:** 10.1186/s13041-021-00802-w

**Published:** 2021-06-14

**Authors:** Sujin Chae, Jiso Hong, Keunsoo Kang, Anna Shin, Dae-Gun Kim, Sinjeong Lee, Moo-young Kim, Inkyung Jung, Daesoo Kim

**Affiliations:** 1 Behavioral Genetics Lab., Department of Biological Sciences, Korea Advanced Institute of Science and Technology (KAIST), 291 Daehak-ro, Yuseong-gu, Daejeon, 305-701 Korea; 2 KAIST Institute for the BioCentury, Korea Advanced Institute of Science and Technology (KAIST), Daejeon, 305-701 Korea; 3 Department of Microbiology, College of Natural Sciences, Dankook University, Chungnam, 31116 Korea

**Keywords:** Molecular laterality, Social defeat stress, Connective tissue growth factor (CTGF), Medial prefrontal cortex, Depression

## Abstract

Functional lateralization of the prefrontal cortex has been implicated in stress and emotional disorders, yet underlying gene expression changes remains unknown. Here, we report molecular signatures lateralized by chronic social defeats between the two medial prefrontal cortices (mPFCs). Stressed mice show 526 asymmetrically expressed genes between the mPFCs. This cortical asymmetry selectively occurs in stressed mice with depressed social activity, but not in resilient mice with normal behavior. We have isolated highly asymmetric genes including connective tissue growth factor (CTGF), a molecule that modulates wound healing at the periphery. Knockdown of CTGF gene in the right mPFC by shRNA led to a stress-resistant behavioral phenotype. Overexpression of CTGF in the right mPFC using viral transduction induces social avoidance while the left mPFC thereof prevent stress-induced social avoidance. Our study provides a molecular window into the mechanism of stress-induced socioemotional disorders, which can pave the way for new interventions by targeting cortical asymmetry.

**Supplementary Information:**

The online version contains supplementary material available at 10.1186/s13041-021-00802-w.

## Introduction

Stress induces numerous physiological changes in the brain, but only some individuals develop emotional disorders. Studies have sought to identify key mechanisms and molecules that explain the relationship between stress and emotional disorders. Brain imaging studies have revealed that patients with post-traumatic stress disorder (PTSD) [1, 2] or depression [3–5] show asymmetric activity between the two prefrontal cortices (PFC). The subgenual PFC (sgPFC) is a common site of the asymmetric changes observed in patients with bipolar and unipolar depression [6]. Chronic stress has been shown to induce asymmetric changes in the structure [7, 8] and activity [9] of the medial PFC (mPFC), the rodent counterpart of the sgPFC, supporting the relevance of cortical asymmetry in stress-induced emotional disorders.

According to the functional divergence hypothesis, the two mPFC hemispheres play different roles in stress perception and resilience. In mice [9] and rats [10], a lesion in the right mPFC has been shown to cause a stress-resistant phenotype, whereas the effects of such lesions in the left mPFC are not significant [10]. Chronic social defeat stress specifically depresses activity in the left mPFC but not the right mPFC; consistent with this, optogenetic stimulation of activity in the depressed left mPFC, but not the depressed right mPFC, restores stress-induced social avoidance, which is a pro-resilience effect [9]. Despite the data derived from these lesion and gain-of-function studies, however, the physiological mechanisms and related molecular markers associated with stress-induced emotional changes remain unclear.

Several studies have sought to identify molecular effectors that mediate stress responses by exploring stress-induced changes of gene expression in the mPFC [11–13]. Comparative analyses have found that certain genes associated with the vascular system, brain injury, stress hormone responses, epigenetic modulation, and other phenomena are differentially expressed between mice that are susceptible and resilient to chronic social defeat stress [14–16], suggesting possible mechanisms underlying the resilience to chronic stress. However, whether the mPFC shows hemisphere-specific molecular changes under chronic stress was previously unknown. In the present study, we compared gene expression profiles between the two mPFC hemispheres in stressed mice that were classified into susceptible and resilient groups according to whether they showed social avoidance. We found that stress-induced gene expression profiles are lateralized between the left and right mPFC hemispheres, and that this can contribute to explaining some socio-emotional behaviors.

## Methods and materials

### Animals

C57BL/6 J male mice were used for all experiments. Before stress exposure, mice were housed in groups of 5 or 6 under a 12:12 h light:dark cycle (lights on, 07:00) with food and water available ad libitum. All animal care and experimental procedures were performed in accordance with protocols approved by the Animal Care and Use Committee of Korea Advanced Institute of Science and Technology (KAIST).

### Stereotaxic injection

Five-week-old mice were anesthetized with Avertin (2,2,2-tribomoethanol; Sigma, USA) and placed in a stereotaxic frame (Neurostar, Germany) for injection of viral vectors. For small hairpin RNA (shRNA)-mediated knockdown of CTGF, the viral vector AAV2/9-GFP-U6-mCTGF-shRNA (Vector Biolabs, USA, 0.5 ul of 10^13^ GC/ml titer) was delivered to the mPFC (anteroposterior, + 1.95 mm; mediolateral, ± 0.35 mm; dorsoventral, − 1.80 mm) using a Nanofil syringe with a 33-gague injection needle (World Precision Instruments, Inc., USA). As a control, AAV2/5-Scramble shRNA-CMV-mCherry-hGH (Penn Vector Core, USA, 0.5 μl of 2.4 $\times$ 10^12^ GC/ml titer) was injected into the same location. Overexpression of CTGF in the mPFC was achieved by delivering AAV2/9-CamKIIα-mCTGF-IRES-mCherry (Vector Biolabs, USA, 0.5 μl of 2.5 $\times$ 10^13^ GC/ml titer) to the mPFC; as a control, AAV2/5-CamKIIα-mCherry (Penn Vector Core, USA, 0.5 μl of 4 $\times$ 10^12^ GC/ml titer) was injected into the mPFC in the same manner. After surgery, mice were returned to their home cages and housed for 3 weeks to allow recovery and viral expression.

### Chronic social defeat stress and social interaction test

Retired CD-1 breeder mice (> 15 weeks old) were screened for aggression as previously described [15, 16]. Each 8-week-old experimental mouse was introduced into the home cage of an unfamiliar, aggressive CD-1 mouse for a 10-min physical defeat exposure. After the attack, the CD-1 and C57BL/6 J mice were separated by a transparent acryl panel containing multiple holes that enabled sensory contact. Control, non-defeated mice were housed in identical cages and rotated in a similar manner. This social defeat stress paradigm was performed in the few hours before the onset of the dark phase (17:00–18:00). After 10 days of social defeat, all mice were singly housed.

The test for social avoidance was performed in a dark room in a white acrylic open-field box (42 × 42 cm) with a removable iron mesh cage (10 × 6.5 × 42 cm) that was used to secure the social target in the middle of one side of the box. Each experimental mouse was placed in the center of the open field and allowed to move freely for 150 s in the absence and presence of a social target (i.e., an unfamiliar CD-1 mouse). All mouse behavior was recorded under infrared lights using a digital video camera, and the time spent in the interaction zone was analyzed using EthoVision XT (Noldus, Netherland). The interaction zone was defined as previously described (14 × 24 cm rectangular area, 8 cm around iron mesh cage) [17]. A social preference index (or interaction ratio) was calculated as the ratio of the time spent in the interaction zone in the presence or absence of the target CD-1 aggressor mouse. Mice with interaction ratios > 1 were defined as resilient, whereas mice with interaction ratios < 1 were deemed susceptible.

### Forced swim test (acute stress)

Experimental mice were subjected to a forced swim stress as previously reported [18]. Briefly, mice were placed in a transparent plastic cylinder (diameter 10 cm, height 30 cm) filled with 20 cm of water at a temperature of 24 °C ± 1 °C for 6 min. Mouse behavior was recorded using a video camera, and the onset and total duration of immobility during the last 4 min were analyzed using EthoVision XT (Noldus, Netherland).

### Sucrose preference test

The sucrose preference test (SPT) was adapted from the previously reported 8-day sucrose preference protocol [19], which is a highly reliable method of testing chronic stress-induced anhedonia. Mice subject to social defeat stress were introduced to the sucrose preference paradigm on the day of the social interaction test. The utilized SPT apparatus was designed to have 10 chambers of the same size. Adaptation was performed over day 1 to day 4, with day 1 to day 3 being used for sucrose solution adaptation (1% (wt/vol)) and day 3 and day 4 being used for apparatus adaptation. Two rounds of baseline measurements were collected from day 4 to day 6, after which the mice were deprived of food and water for 1 day. On day 7 to day 8, the preference test was carefully done for 12 h. The sucrose preference calculated by the equation, sucrose intake/total intake (sucrose + regular water) $\times$ 100%. Anhedonia was defined as a reduction of the sucrose preference compared to the control.

### Microarray experiments and data analysis

The raw microarray data have been deposited in the NCBI Gene Expression Omnibus at accession number GSE114224. Mice exposed to chronic social defeat stress were subdivided into two groups according to their sociability index (SI). The average of SI of mice for microarray analysis is 1.76 (control; n = 8), 1.61 (resilient; n = 8), and 0.5 (susceptible; n = 7). Non-defeated control mice were also selected from those who underwent a social interaction test [17]. One day after the social-interaction tests, the mice were sacrificed and their left and right mPFCs were dissected separately on an ice-chilled plate and immediately frozen in liquid nitrogen (LN_2_). Total RNA was prepared from each of the representative mice using the TRIzol reagent (Invitrogen, USA) and further purified using an RNeasy kit (Qiagen, USA). RNAs were isolated from the left and right mPFC tissues of susceptible, resilient, and control mice and used for triplicate microarray analyses on a MouseRef-8_V2 Expression BeadChip (Illumina, USA).

The prepared microarrays were scanned with an Illumina BeadArray reader and preprocessed with the Illumina GenomeStudio software (version 1.0.6). Raw signals of at least two biological replicates per condition were transformed to the log_2_ scale and normalized using the Bioconductor *lumi* package [20]. In cases where the probes for a given gene yielded a *p*-value (detection *p*-value) greater than 0.05, the gene was excluded from further analysis. Differentially expressed genes (DEGs) were identified using the Bioconductor *limma* package [21], with a false discovery rate (FDR)-adjusted *p*-value cutoff of 0.05. The expression of significant DEGs was compared in seven pairwise groups: susceptible left vs. susceptible right; resilient left vs. resilient right; control left vs. control right; susceptible left vs. control left; susceptible right vs. control right; resilient right vs. control right; and resilient left vs. control left.

The functions of DEGs with an FDR-adjusted *p*-value cutoff of 0.05 were predicted by gene ontology (GO) analysis using GeneMANIA [22]. Briefly, GeneMANIA receives a set of input genes as well as other genes related to the input and compares them to a large set of functional association data, including protein and genetic interactions, pathways, co-expression, co-localization, and protein domain similarity.

### Real time quantitative reverse transcription-polymerase chain reaction (RT-qPCR)

Our microarray gene expression results were confirmed by RT-qPCR using three biological replicates of the four independent samples applied to the microarray analyses. Commercially available TaqMan Gene Expression Assay primers for the target genes, *Cux2* (Mm00500377_m1), *Wfs1* (Mm00495979_m1), *Mbp* (Mm01266402_m1), and *Rprm* (Mm00469773_s1), *Ctgf* (Mm01192933_g1), and the reference gene, *Gapdh* (Mm99999915_g1), were obtained from Applied Biosystems (USA).

### Western blotting

Each mouse brain mPFC was harvested, snap-frozen in LN_2_, and stored at − 70 °C until use. The brain tissue was homogenized with T-Per buffer (tissue protein extraction buffer, #78510; Thermo Scientific, USA) containing 1 × protease inhibitor cocktail (cOmplete, EDTA-free; Roche Diagnostics, USA) and 1 × phosphatase inhibitor cocktail (PhosSTOP; Roche Diagnostics). Lysates were incubated on ice for 1 h with frequent vortexing, after which cleared lysates (supernatants) were collected by centrifugation at 13,000 rpm for 20 min at 4 °C. For detection of CTGF and β-actin, equal amounts of total protein (30 μg) were first incubated at 95 °C for 5 min in sodium dodecyl sulfate (SDS) sample buffer and then resolved by 12% SDS-PAGE (polyacrylamide gel electrophoresis). The resolved proteins were transferred to nitrocellulose (NC) membranes, which were incubated with primary anti-CTGF (sc-14939, sc-365970; Santa Cruz Biotechnology, USA) or anti-β-actin (sc47778; Santa Cruz Biotechnology) antibodies, and then with horseradish peroxidase (HRP)-conjugated secondary antibodies. Immunoreactive proteins were visualized using a SuperSignal West Pico System (Thermo Scientific). The amount of protein expressed was calculated using the ImageJ program (NIH, USA).

### Immunohistochemistry

After all behavioral experiments were completed, mice were overdosed with Avertin (2,2,2-tribomoethanol; Sigma) and perfused first with heparin solution (66.5 μg/ml) in phosphate-buffered saline (PBS) and then with 4% formaldehyde in PBS. Brains were removed, post-fixed by an overnight incubation in PBS containing 4% formaldehyde, and cut into 40-μm-thick coronal sections using a vibratome (VT1200S; Leica, Germany). The resulting brain slices were permeabilized with PBS containing 0.5% Triton-X for 30 min and then transferred to blocking solution containing 10% normal donkey serum (D9663; Sigma) for 1 h. Brain sections were incubated overnight at 4 °C with goat anti-CTGF primary antibody (1:200, sc-14939; Santa Cruz), and then incubated for 2 h with Cy5-conjugated anti-goat (1:200, 305–175-003; Jackson ImmunoResearch, USA) or fluorescein isothiocyanate (FITC)-conjugated anti-goat (1:200, 305-095-003; Jackson ImmunoResearch, USA) secondary antibodies plus 4’,6-diamidino-2-phenylindole (DAPI; 1:1000). Finally, brain sections were mounted on glass slides and imaged with a confocal laser-scanning microscope (LSM780; Zeiss, Germany).

### Statistical analysis

The numbers of mice used (n) are indicated in the Results and Figure Legends. The graphed values present the mean ± SEM. Comparison between two groups were analyzed using the two-tailed Student’s t-test. We used the two-way RM ANOVA when significant differences between paired two measurements in the same individual analyzed (e.g., no-target/target). When the data did not pass normality, we used the Mann–Whitney Rank Sum Test instead of t-test. Comparisons across more than two groups and between multiple variables were made using two-way ANOVA, followed by post-hoc tests when any of the main effect or interaction was significant at *p* < 0.05. All statistical analyses were performed using SigmaPlot 12.0 (Systat Software Inc.) with a significance threshold of *p* < 0.05. (Indicated as n.s. > 0.05, * < 0.05, ** < 0.01) (Additional file 9: Table S7).

## Results

### Chronic social defeat stress induces asymmetric gene expression in the mPFC

**Fig. 1:**
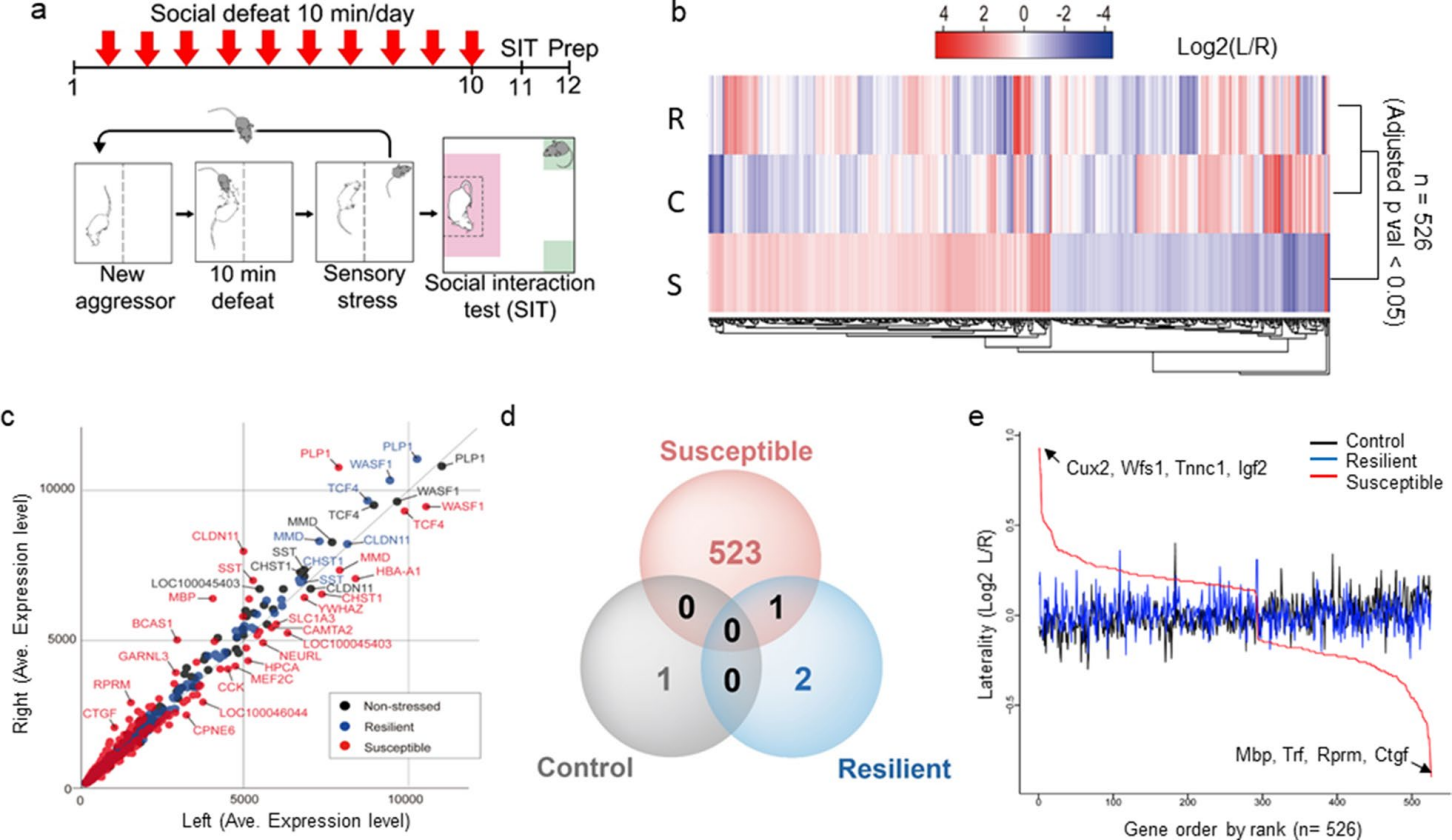
Experimental scheme for social defeat stress and differences in stress-induced gene expression profiles between the mPFC cortices. **a** Timeline of the utilized social defeat stress paradigm; mPFC tissue was prepared as indicated 1 day after the social interaction test. **b** Log_2_ L/R values are graphically presented in heatmap format, where an intense red color indicates that the gene is highly expressed in the left (L) mPFC and an intense blue color indicates that the gene is highly expressed in the right (R) mPFC. Genes with an FDR (false discovery rate) adjusted p-value less than 0.05 are rearranged according to the log_2_ L/R ratio. Genes with a *p*-value > 0.05 were not included in the analysis. S = Susceptible, R = Resilient, C = Control. **c** Averaged gene expression levels from microarray experiments, with left mPFC values plotted on the *x*-axis and right mPFC values plotted on the *y*-axis. The distribution of genes expressed in susceptible mice (red dots) is compared with that of genes from resilient mice (blue dots) and non-stressed control mice (black dots). **d** Venn diagram showing the number of genes exhibiting significant laterality in the various mouse groups. Red circle, 524 genes exhibited laterality in susceptible mice (one gene in common with resilient mice); blue circle, three genes exhibited laterality in resilient mice (one gene in common with susceptible mice); black circle, one gene exhibited laterality in non-stressed control mice. The total list is presented in Additional file 4: Table S2. **e** The *x*-axis indicates genes ordered by rank and the *y*-axis indicates genes ordered by laterality value (log_2_ L/R). Positive laterality indicates genes that are highly expressed in the left mPFC relative to the right mPFC, whereas negative laterality denotes genes that are highly expressed in the right mPFC. *Cux2, Wfs1, Tnnc1*, and *Igf2* are left-mPFC lateralized, while *Ctgf, Rprm, Trf*, and *Mbp* are right-mPFC oriented genes

To determine whether stress induces gene expression asymmetry in the mPFC, we performed chronic social defeat experiments using C57BL/6 J mice, as previously described [17] (Fig. 1a). We first divided stressed mice into two groups—resilient and susceptible—according to their sociability index (SI), which is a measure of the presence or absence of a social preference for a novel encounter (CD-1 mice) (Additional file 1: Figure S1a). The average SI of non-stressed control, resilient, and susceptible mice were 1.5, 1.5, and 0.8, respectively (Additional file 1: Figure S1a), which is consistent with previous descriptions [23–25]. We then excluded mice with intermediate SI values between 1.1 ~ 0.9, as susceptible and resilience traits may potentially overlap in this range. For the mice that were finally selected in this way for our microarray analysis, the average SI were 1.76 (control; n = 8), 1.61 (resilient; n = 8), and 0.5 (susceptible; n = 7) (Additional file 1: Figure S1b, c). One day after the sociability tests, we collected the left and right mPFC, including the infralimbic and prelimbic areas, from the three groups of mice. We extracted and pooled equal amounts of RNAs from individual tissues and performed a microarray analysis (Fig. 1a, Additional file 1: Figure S1c).

To assort DEGs according to functions, we calculated a laterality score, defined as the log ratio of gene expression intensity between the left (L) and right (R) mPFC (log_2_ L/R) of in each of control, resilient and susceptible mice. The laterality of genes between hemispheres of control and stressed mice were analyzed. A correlation analysis using 526 DEGs with meaningful log_2_ L/R values (FDR-adjusted *p*-value < 0.05) revealed that susceptible mice showed a higher laterality score than resilient and non-stressed control mice (Fig. 1b, Additional file 4: Table S2). Resilient mice showed a smaller number of lateralized genes (Fig. 1c, 1d, Additional file 5: Table S3) despite changes in a significant number of genes compared with non-stressed controls (Additional file 3: Table S1), suggesting that these genes are changed in a similar direction in both hemispheres.

### Highly asymmetric genes in susceptible mice

We then performed a rank-order analysis of the 526 genes according to their Log_2_ L/R values to identify individual genes that showed extreme laterality in the mPFC (Fig. 1e, Additional file 6: Table S4). Genes showing greater expression in the left than right mPFC were *Cux2* (cut-like homeobox 2), *Wfs1* (wolframin ER transmembrane glycoprotein), *Tnnc1* (troponin C, cardiac/slow skeletal) and *Igf2* (insulin-like growth factor 2). Genes with higher expression in the right mPFC were *Mbp* (myelin basic protein), *Trf* (transferrin), *Rprm* (reprimo, TP53-dependent G2 arrest mediator candidate) and *Ctgf* (connective tissue growth factor). The laterality of these genes was confirmed by RT-qPCR analysis of individual RNA samples obtained from susceptible mice (Additional file 2: Figure S2). The right or left lateralized genes were checked for their transcript expressions compare to control and resilient mice. The normalized expression (by control left) and laterality ratio (L/R) are shown in several highly lateralized genes. The left oriented laterality genes (Cux2, Wfs1) (Additional file 2: Figure S2a, c) and the expression of right oriented genes (*Ctgf, Mbp, Rprm*) (Additional file 2: Figure S2b, d) are analyzed and its expression is confirmed.

To check the possibility that the DEGs results from difference in the amount of total RNA, we compared the expression levels of the housekeeping genes, *Gapdh* (glyceraldehyde-3-phosphate dehydrogenase), *Actb* (β-actin), and *B2m* (β_2_ microglobin). We found that these genes showed no difference between the two hemispheres in pairwise group comparisons (*p* > 0.1 for *Actb*; *p* > 0.4 for *Gapdh; p* > *0.1* for *B2m*), indicating that DEGs are screened from the equivalent amounts of RNA from each hemisphere (Additional file 7: Table S5). These results suggest that the gene expression laterality between the two mPFC hemispheres involves stress susceptibility.

### Functional annotation of lateralized genes in susceptible mice

**Fig. 2:**
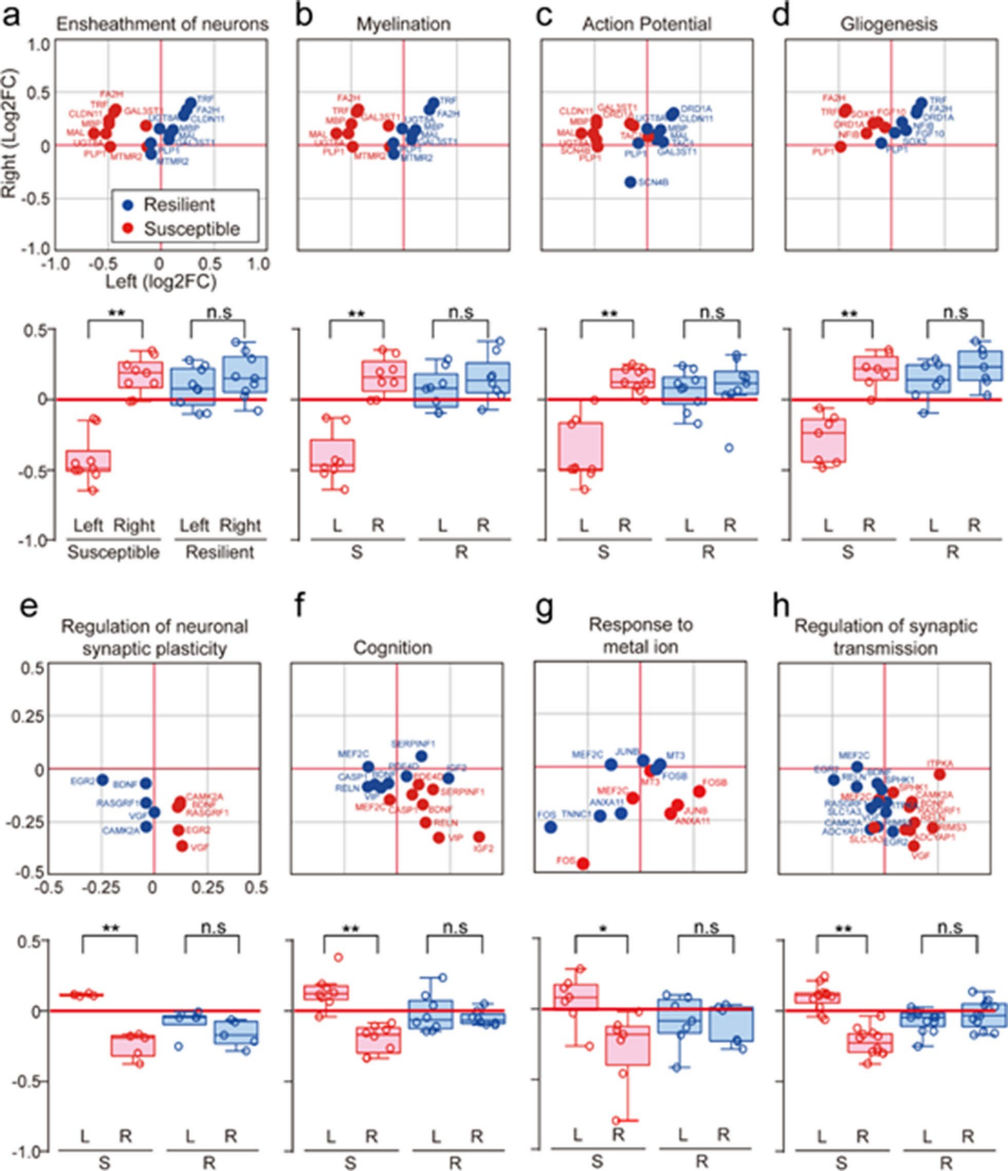
Functional annotation of genes that show lateralization in susceptible mice. GO analysis of genes exhibiting laterality. The GeneMANIA program with the Cytoscape plugin was used for GO analysis. **a** Upper panel: The genes *Cldn11, Fa2h, Gal3st1, Mal, Mbp, Mtmr2, Plp1, Trf*, and *Ugt8a* were associated with the GO term, ensheathment of neurons, and were dominantly expressed in the right mPFC hemisphere of susceptible mice. Fold changes (FC) in gene expression relative to controls, expressed as log_2_ FC values, are shown in a dot plot, where the *x*-axis represents values for the left mPFC and the *y*-axis represents values for the right mPFC. Blue, resilient values; red, susceptible values. Lower panel: Results are presented in box plot format (**p = 0.000000330 for susceptible left vs. susceptible right, *t*_*16*_ = -8.324, t-test, n = 9). **b** Upper panel: The genes *Fa2h, Gal3st1, Mal, Mbp, Mtmr2, Plp1, Trf* and *Ugt8a* were associated with the GO term, myelination, and were dominantly expressed in the right mPFC hemisphere of susceptible mice. Log_2_ FC values are shown in a dot plot. Blue, resilient values; red, susceptible values. Lower panel Results are presented in box plot format (**p = 0.00000432 for susceptible left vs. susceptible right, *t*_*14*_ = -7.235, t-test, n = 8). **c** Upper panel: The genes *Cldn11, Drd1a, Gal3st1, Mal, Mbp, Plp1, Scn4b, Tac1*, and *Ugt8a* were associated with the GO term, action potential, and were dominantly expressed in the right hemisphere of susceptible mice. Log_2_ FC values are shown in a dot plot. Blue, resilient values; red, susceptible values. Lower panel: Results are presented in box plot format (**p = 0.00000462 for susceptible left vs. susceptible right, *t*_*16*_ = -6.756, t-test, n = 9). **d** Upper panel: The genes *Drd1a, Fa2h, Fgf10, Nfib, Plp1, Sox5*, and *Trf* were associated with the GO term, gliogenesis, and were dominantly expressed in the right hemisphere of susceptible mice. Log_2_ FC values are shown in a dot plot. Blue, resilient values; red, susceptible values. *Lower panel:* Results are presented in box plot format (**p = 0.0000522 for susceptible left vs. susceptible right, *t*_*12*_ = -6.114, t-test, n = 7). **e** Upper panel: The genes *Bdnf, Camk2a, Egr2, Rasgrf1*, and *Vgf* were associated with the GO term, neural synaptic plasticity, and were dominantly expressed in the left hemisphere of susceptible mice. Log_2_ FC values are shown in a dot plot. Blue, resilient values; red, susceptible values. Lower panel: Results are presented in box plot format (**p = 0.0000245 for susceptible left vs. right, *t*_*8*_ = 8.665, t-test, n = 5). **f** Upper panel: The genes *Bdnf, Casp1, Igf2, Mef2c, Pde4d, Reln, Serpinf1*, and *Vip* were associated with the GO term, cognition, and were dominantly expressed in the left hemisphere of susceptible mice. Log_2_ FC values are shown in a dot plot. Blue, resilient values; red, susceptible values. Lower panel: Results are presented in box plot format (**p = 0.0000276 for susceptible left vs. susceptible right, *t14* = 6.096, t-test, n = 8). **g** Upper panel: The genes *Anxa11, Fos, Fosb, Junb, Mef2c, Mt3*, and *Tnnc1* were associated with the GO term, response to metal ion, and were dominantly expressed in the left hemisphere of susceptible mice. Log_2_ FC values are shown in a dot plot. Blue, resilient values; red, susceptible values. Lower panel: Results are presented in box plot format (*p = 0.0148 for susceptible left vs. susceptible right, *t*_*12*_ = 2.844, t-test, n = 7). **h** Upper panel: The genes *Adcyap1, Bdnf, Camk2a, Egr2, Itpka, Mef2c, Rasgrf1, Reln, Rims3, Slc1a3, Sphk1*, and *Vgf* were associated with the GO term, synaptic transmission, and were dominantly expressed in the left hemisphere of susceptible mice. Log_2_ FC values are shown in a dot plot. Blue, resilient values; red, susceptible values. Lower panel: Results are presented in box plot format (**p = 0.0000000233 for susceptible left vs. susceptible right, *t*_*22*_ = 8.454, t-test, n = 12)

**Fig. 3:**
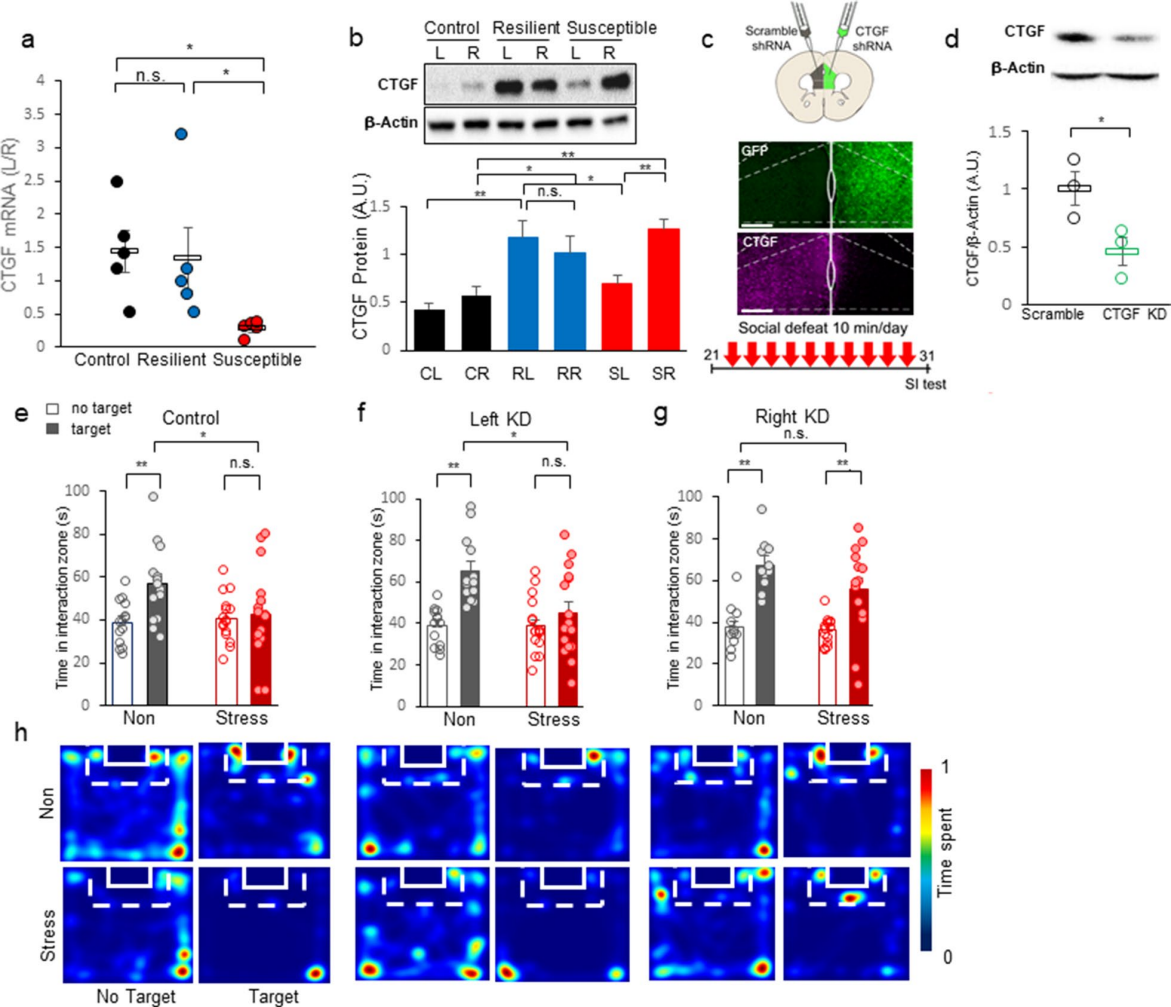
CTGF knockdown in the right mPFC prevents chronic social defeat stress-induced increases in social avoidance. **a** The L/R expression ratio of *Ctgf* in the mPFC. The L/R ratio of susceptible mice is significantly less than those of control and resilient mice (H = 9.620, df = 2, **p = 0.008, Kruskal–Wallis One Way Analysis of Variance on Ranks; between control and susceptible, *p < 0.05, between control and resilient, *p* > 0.05. between resilient and susceptible, *p < 0.05, Post-hoc analysis with Turkey test). **b** Upper panel: A representative blot of CTGF protein expression. Lower panel: CTGF protein expression in the mPFC, presented as a bar graph. The CTGF level is significantly different between the left and right mPFC in susceptible mice (For group $\times$ laterality F(2, 38) = 4.489, *p = 0.018; **p = 0.002, laterality within resilient *p* = 0.357; two-way ANOVA with post-hoc analysis). The CTGF expression in resilient and susceptible mice showed a significant difference compare to control (within left mPFC, control and resilient, **p < 0.001, resilient and susceptible, *p = 0.016; within right mPFC, control and resilient, *p = 0.025, control and susceptible, **p < 0.001; two-way ANOVA with post-hoc analysis). **c** Upper panel: Scheme of injection to the mPFC. Middle panel: Representative immunohistochemistry image showing shRNA-mediated CTGF knockdown. Scale bar = 200 μm. Lower panel: Experimental timescale of CTGF shRNA expression followed by chronic social defeat stress and behavioral analysis. **d** Upper panel: Confirmation of CTGF knockdown (KO) by Western blot analysis. Lower panel: Quantitative analysis of CTGF knockdown, expressed as the ratio CTGF/β-actin in arbitrary units. Image quantification and analysis was done using the ImageJ program (*p = 0.0464 for scrambled [Control] vs. KO, t-test, t_4_ = 2.850; Control, n = 3; KO, n = 3). **e** Interaction times with CD-1 mice under non-stressed (Con) and stressed (+ Stress) conditions are presented as means ± standard deviation. Comparison of time spent in the interaction zone by non-stressed (black bar) and stressed (red bar) mice injected in both hemispheres of the mPFC with scrambled shRNA. Open bar, time spent in the interaction zone without a target; closed bar, time spent in the interaction zone with a target present (For stress $\times$ target, F(1, 28) = 5.126, *p = 0.032, two-way RM ANOVA; within non-stressed, effect of target, **p = 0.002; within stressed, effect of target, *p* = 0.709; within non-target, effect of stress, *p* = 0.739, within target, effect of stress, *p = 0.019, Post-hoc analysis with Holm-Sidak method). **f** Comparison of time spent in the interaction zone by non-stressed and stressed mice with CTGF shRNA expression in the left mPFC and scrambled shRNA in the right hemisphere (For stress $\times$ target, F(1, 28) = 4.478, *p = 0.043, two-way RM ANOVA; within non-stressed, effect of target, **p < 0.001; within stressed, effect of target, *p* = 0.080; within non-target, effect of stress, *p* = 0.949, within target, effect of stress, *p = 0.014, Post-hoc analysis with Holm-Sidak method). **g** Comparison of time spent in the interaction zone by non-stressed and stressed mice with CTGF shRNA expression in the right mPFC and scrambled shRNA in the left hemisphere (For target F(1, 24) = 58.986, **p < 0.001, stress $\times$ target, F(1, 24) = 2.480, *p* = 0.128, two-way RM ANOVA; within non-stressed, effect of target, **p < 0.001; within stressed, effect of target, **p < 0.001; Post-hoc analysis with Holm-Sidak method). **h** Left: Representative heatmaps of normalized time spent by mice in the indicated locations without (No Target) and with (Target) a CD-1 target mouse. Non-stressed mice (Non) and stressed mice (Stress) that had been injected in both hemispheres of the mPFC with scrambled shRNA (Control). Center: Representative heatmaps of normalized time spent in the indicated locations by mice with CTGF shRNA expression in the left mPFC and scrambled shRNA in the right mPFC (Left KD). Right: Representative heatmaps of normalized time spent in the indicated locations by mice with CTGF shRNA expression in the right mPFC and scrambled shRNA in the left hemisphere (Right KD)

To better understand the functions of lateralized genes in susceptible mice, we categorized them based on their functional properties in the gene ontology (GO) database (Fig. 2, Additional file 8: Table S6). The GO terms for genes with negative left log_2_ FC (fold change) values (reflecting lower expression in the left mPFC) included ensheathment of neurons (*Cldn11, Fa2h, Gal3st1, Mal, Mbp, Mtmr2, Plp1, Trf, Ugt8a*) (Fig. 2a), myelination (*Fa2h, Gal3st1, Mal, Mbp, Mtmr2, Plp1, Trf, Ugt8a*) (Fig. 2b), action potentials (*Cldn11, Drd1a, Gal3st1, Mal, Mbp, Plp1, Scn4b, Tac1, Ugt8a*) (Fig. 2c), and gliogenesis (*Drd1a, Fa2h, Fgf10, Nfib, Plp1, Sox5, Trf*) (Fig. 2d). The GO terms for genes with positive log_2_ FC values (reflecting increased expression in the left or decreased expression in the right mPFC) included neuronal synaptic plasticity (*Bdnf, Camk2a, Egr2, Rasgrf1, Vgf*) (Fig. 2e), cognition (*Bdnf, Casp1, Igf2, Mef2c, Pde4d, Reln, Serpinf1, Vip*) (Fig. 2f), response to metal ions (*Anxa11, Fos, Fosb, Junb, Mef2c, Mt3, Tnnc1*) (Fig. 2g), and synaptic transmission (*Adcyap1, Bdnf, Camk2a, Egr2, Itpka, Mef2c, Rasgrf1, Reln, Rims3, Slc1a3, Sphk1, Vgf*) (Fig. 3h). These functional clusters were prominent in susceptible mice and have previously been associated with various neuronal responses to stress, including neural activity depression [26, 27]_,_ neuronal degeneration with gliosis [28], and cognitive alterations [29]. Our results are also consistent with the previous finding that depression of neural activity in the left mPFC contributes to social depression in susceptible mice [5, 9].

### Differential expression of CTGF in social defeat stress

CTGF is a key growth factor involved in wound healing at the periphery [30, 31] and in the brain [32]. It is known to mediate interneuron degeneration [33], demyelination, and gliosis [28, 34, 35], and thereby contributes to effects similar to those induced by chronic stress in the mPFC. We thus, selected CTGF as a functional case study. A recent study of postmortem samples showed that CTGF is increased in the hippocampus and amygdala of patients with major depressive disorder (MDD) [36]. The most highly lateralized gene on our microarray results was *Ctgf* (Fig. 1). We validated this finding by examining *Ctgf* expression in susceptible mice, as assessed by laterality ratio (L/R) in susceptible mice compared to those in control and resilient mice (Fig. 3a). We found that the three groups show different asymmetry patterns of *Ctgf* gene expression, and that the transcript of C*tgf* recapitulated the microarray results. We observed a significant difference in RNA expression as laterality score (Left/Right) (Fig. 3a; H = 9.620, df = 2, **p = 0.008, Kruskal–Wallis One Way Analysis of Variance on Ranks; between control and susceptible, *p < 0.05, between control and resilient, *p* > 0.05. between resilient and susceptible, *p < 0.05, Turkey test post-hoc). We checked whether the functional protein level was consistent with the transcript profile. Importantly, we found that the CTGF protein level increased in the right hemisphere of susceptible mice and both hemispheres of resilient mice under stress (Fig. 3b). Our analysis revealed that CTGF proteins showed a significant right-oriented expression in susceptible mice (Fig. 3b, upper panel) and a significant difference in expression between the left and right hemispheres (Fig. 3b, lower panel: For group F(2, 38) = 14.445, **p < 0.001, laterality F(1, 38) = 3.634, *p* = 0.064, group $\times$ laterality interaction F(2, 38) = 4.489, *p = 0.018, laterality within susceptible **p = 0.002, two-way ANOVA with Holm-sidak post-hoc analysis). In the resilient mice, we did not detect any significant between-hemisphere difference in CTGF expression (Fig. 3b, lower panel; laterality within resilient *p* = 0.357; two-way ANOVA). The CTGF expression in resilient and susceptible mice showed a significant difference compare to control (within left mPFC, control and resilient, **p < 0.001, control and susceptible, *p* = 0.120, resilient and susceptible, *p = 0.016; within right mPFC, control and resilient, *p = 0.025, control and susceptible, **p < 0.001, resilient and susceptible, *p* = 0.166, two-way ANOVA with Holm-sidak post-hoc analysis). These findings indicate that the RNA and protein expression levels of CTGF are lateralized in susceptible mice but balanced in resilient and control mice, suggesting that the laterality score could be a relevant biomarker for stress susceptibility.

### CTGF is functionally lateralized in the mPFC hemispheres

To induce hemisphere-specific knockdown of CTGF, we injected a viral vector expressing an *sh*RNA targeting CTGF (AAV2/9-GFP-U6-mCTGF-shRNA) into the left or right mPFC. As a control for the effects of viral transfection, we injected a non-targeting *sh*RNA-expression vector (AAV2/5-Scramble *sh*RNA-CMV-mCherry-hGH) into the contralateral mPFC (Fig. 3c). After 3 weeks, we subjected the same mice to 10 days of chronic social defeat stress followed by social interaction tests (Fig. 3c, lower panel). Using immunostaining and Western blotting, we found that the CTGF *sh*RNA effectively reduced the CTGF protein level in the mPFC (Fig. 3c, d; *p = 0.0464, t_4_ = 2.850, t-test). After the 10-day social defeat protocol, control mice with bilateral mPFC injections of scrambled *sh*RNA showed a significant increase in social avoidance of CD-1 mice (Fig. 3e; For stress, F(1, 28) = 1.731, *p* = 0.199, for target F(1, 28) = 7.721, *p = 0.010, stress $\times$ target interaction, F(1, 28) = 5.126, *p = 0.032, two-way RM ANOVA; within non-stressed, effect of target, **p = 0.002; within stressed, effect of target, *p* = 0.709; within non-target, effect of stress, *p* = 0.739, within target, effect of stress, *p = 0.019, Post-hoc analysis with Holm-Sidak method). Mice with left mPFC CTGF depletion showed levels of social avoidance similar to those of control mice (Fig. 3f; For stress, F(1, 28) = 2.514, *p* = 0.124, for target F(1, 28) = 20.319, **p < 0.001, stress $\times$ target interaction, F(1, 28) = 4.478, *p = 0.043, two-way RM ANOVA; within non-stressed, effect of target, **p < 0.001; within stressed, effect of target, *p* = 0.080; within non-target, effect of stress, *p* = 0.949, within target, effect of stress, *p = 0.014, Post-hoc analysis with Holm-Sidak method). Mice with right mPFC CTGF depletion showed no social avoidance (Fig. 3g; For stress, F(1, 24) = 1.968, *p* = 0.173, for main target F(1, 24) = 58.986, **p < 0.001, stress $\times$ target interaction, F(1, 24) = 2.480, *p* = 0.128, two-way RM ANOVA; within non-stressed, effect of target, **p < 0.001; within stressed, effect of target, **p < 0.001; Post-hoc analysis with Holm-Sidak method). Analysis of representative heatmaps showed that mice with right mPFC CTGF knockdown exhibited reduced social avoidance when the CD-1 target was present (Fig. 3h). These results are highly consistent with a previous report that a lesion in the right mPFC leads to stress resistance [9, 10] and suggest that the upregulation of CTGF in the right mPFC plays an important role in stress perception of all stressed mice.

### CTGF overexpression in the right mPFC facilitates social avoidance

**Fig. 4:**
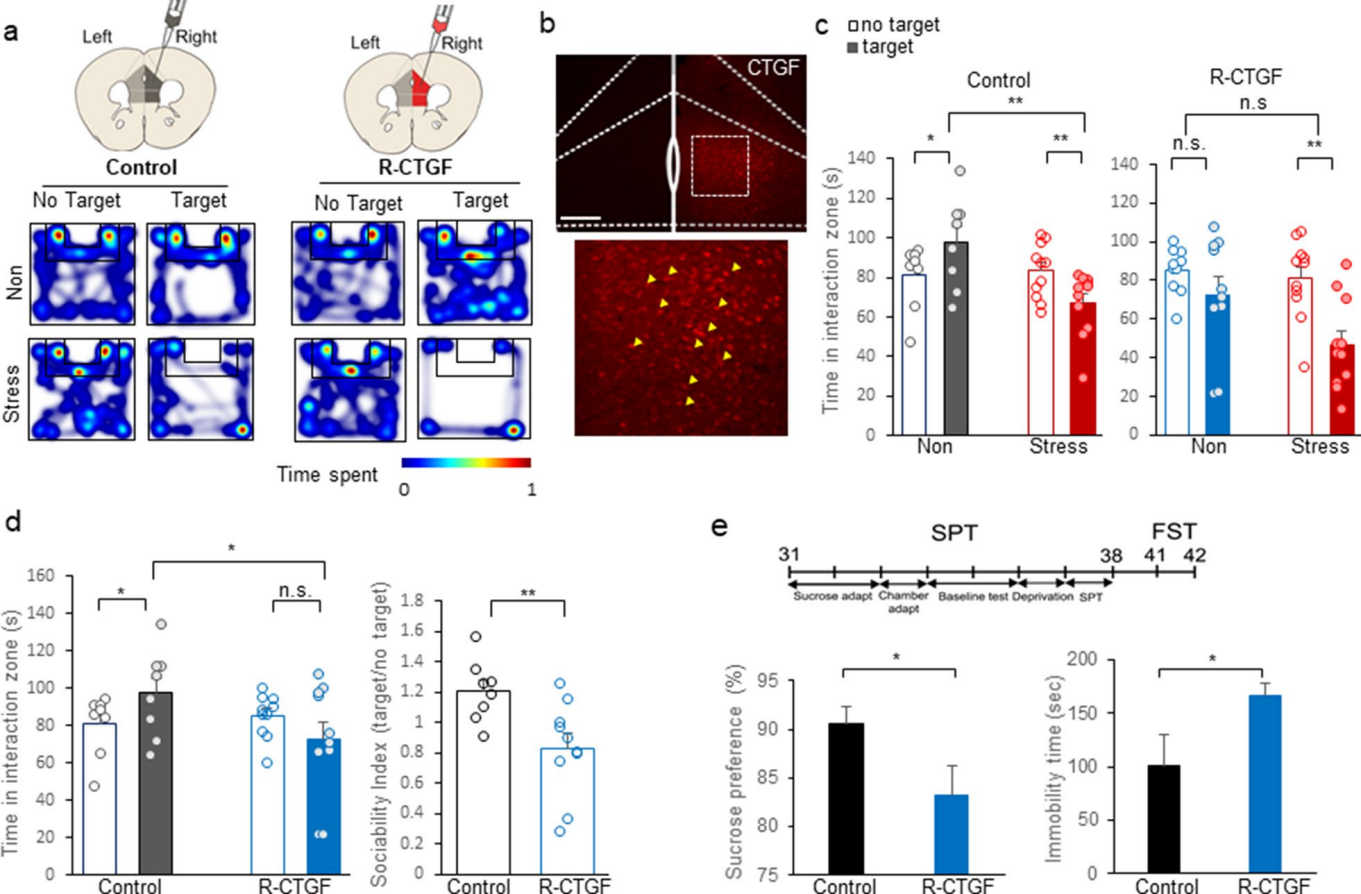
CTGF overexpression in the right mPFC facilitates stress sensitivity. **a** Left panel: Representative heatmap of normalized time spent by control mice in the indicated locations without (No Target) and with (Target) a CD-1 target mouse. The upper (Non) and lower (Stress) rows represent mice injected with AAV-mCherry in the right hemisphere of the mPFC (Control). Right panel: Representative heatmap of normalized time spent by CTGF mice in the indicated locations without (No Target) and with (Target) a CD-1 target mouse. The upper (Non) and lower (Stress) rows represent mice injected with AAV-mCTGF in right hemisphere of the mPFC (R-CTGF). **b** Representative immunohistochemistry image showing AAV-mediated CTGF expression. Scale bar = 200 μm. **c** Interaction times with CD-1 mice under non-stressed and socially stressed conditions are presented as means ± standard deviation. Left panel: Comparison of time spent in the interaction zone by non-stressed (black bar; Non) and stressed mice (red bar; Stress) injected in right hemispheres of the mPFC with AAV-mCherry (Control). Open bar, time spent in the interaction zone without a target; closed bar, time spent in the interaction zone with a target present (For target, F(1, 17) = 0.00147, *p* = 0.970, stress, F(1, 17) = 4.008, *p* = 0.061, target $\times$ stress F(1, 17) = 17.763, **p < 0.001, two-way RM ANOVA; within target absence, effect of stress, *p* = 0.780, n = 11; within target presence, effect of stress, **p < 0.001, n = 11; within non-stress, effect of target, *p = 0.012, within stress the effect of target, **p = 0.005, Post-hoc analysis with Holm-Sidak method). Right panel: Comparison of time spent in the interaction zone by non-stressed (blue bar; Non) and stressed mice (red bar; Stress) injected in right hemispheres of the mPFC with AAV-mCTGF (R-CTGF). (For main target effect, F (1, 19) = 18.637, **p < 0.001, stress, F(1, 19) = 3.371, *p* = 0.082, target $\times$ stress F(1, 19) = 3.965, *p* = 0.061, two-way RM ANOVA; within non-stress, effect of target *p* = 0.125, within stress, effect of target, **p < 0.001; Post-hoc analysis with Holm-Sidak method). **d** Left panel: Comparison of time spent in the interaction zone between control (black bar) and R-CTGF (blue bar) mice (For group, F(1, 16) = 1.266, *p* = 0.277, target, F(1, 16) = 0.143, *p* = 0.710, group $\times$ target, F(1, 16) = 8.921, **p = 0.009, two-way RM ANOVA; within control, effect of target, *p = 0.038, within R-CTGF, effect of target, *p* = 0.068, within target absence, the effect of R-CTGF, *p* = 0.685, within target presence, the effect of R-CTGF, *p = 0.025; Post-hoc analysis with Holm-Sidak method). Right panel: Comparison of sociability index (SI; time spent in interaction zone with a target CD-1 mouse divided by the time without a target) between control (black bar; Control) and right-mPFC^CTGF^ mice (blue bar; R-CTGF) in non-stressed conditions (t_16_ = 3.020, **p = 0.00814, t-test). **e** Upper panel: Experimental timescale of CTGF expression and behavioral analyses (sucrose preference test and forced swim test). Left panel: Comparison of sucrose preference rates (sucrose intake divided by total intake*100 (%)) between control (black bar, Control) and right-mPFC^CTGF^ (blue bar, R-CTGF) mice under the non-stressed condition (*p = 0.033, T = 54, control n = 5, right-mPFC^CTGF^ n = 9, Mann–Whitney Rank Sum Test). *Right panel*: Comparison of helplessness (time spent immobile during the last 4 min of the forced swim test) between control (black bar) and right-mPFC^CTGF^ (blue bar) mice under the non-stressed condition (*p = 0.0256, t_12_ = -2.547, control n = 5, right-mPFC^CTGF^ n = 9, t-test)

Our analysis of CTGF gene expression revealed that CTGF protein increased in the right mPFC of all socially stressed mice, while the corresponding changes in the left mPFC differed by the stress susceptibility of the mice (Fig. 3b). To test the role of increased CTGF in the right mPFC, we expressed AAV2/9-CamKIIα-mCTGF in the right mPFC (Fig. 4a). To generate control mice, we injected the virus, AAV2/5-CamKIIα-mCherry, into the ipsilateral right mPFC. In this experiment, the left mPFC was left intact to check the right hemisphere-specific role of CTGF when social stress is experienced. The expression of CTGF was confirmed by immunohistochemistry (Fig. 4b).

After 3 weeks of expression and 10 consecutive days of social defeat stress, we measured the sociability value by measuring each test mouse’s interaction time with a CD-1 mouse within the interaction zone (Fig. 4c). Heatmaps were generated for the sociability of control mice and those with CTGF expressed in the right-side mPFC (Right-mPFC^CTGF^; R-CTGF) in the absence and presence of a target (for representative maps, see Fig. 4a). Reflecting their normal perception of social stress, control mice showed increased social avoidance when the target CD-1 mouse was present (Fig. 4c left panel; For target, F(1, 17) = 0.00147, *p* = 0.970, stress, F(1, 17) = 4.008, *p* = 0.061, target $\times$ stress interaction, F(1, 17) = 17.763, **p < 0.001, two-way RM ANOVA; within non-stress, effect of target, *p = 0.012, within stress the effect of target, **p = 0.005, within target presence, effect of stress, **p < 0.001; Post-hoc analysis with Holm-Sidak method). However, right-mPFC^CTGF^ mice showed no more social avoidance when socially defeated (Fig. 4c right panel; main target effect, F (1, 19) = 18.637, **p < 0.001, stress, F(1, 19) = 3.371, *p* = 0.082, target $\times$ stress interaction, F(1, 19) = 3.965, *p* = 0.061, two-way RM ANOVA; within non-stress, effect of target *p* = 0.125, within stress, effect of target, **p < 0.001; Post-hoc analysis with Holm-Sidak method).

Furthermore, right-mPFC^CTGF^ mice showed social avoidance under the non-stressed condition, to the point that there was a significant difference in sociability between control and right-mPFC^CTGF^ mice that had not been subjected to stress (Fig. 4d, left panel; For group, F(1, 16) = 1.266, *p* = 0.277, for target, F(1, 16) = 0.143, *p* = 0.710, group $\times$ target interaction, F(1, 16) = 8.921, **p = 0.009, two-way RM ANOVA; within control, effect of target, *p = 0.038, within R-CTGF, effect of target, *p* = 0.068, within target presence, the effect of R-CTGF, *p = 0.025; Post-hoc analysis with Holm-Sidak method). This increase of social avoidance under the non-stressed condition indicates that CTGF expression in the right mPFC led mice to exhibit a socioemotionally depressive state. Our analysis of sociability index (SI) values clearly showed that the right-mPFC^CTGF^ mice were socially avoidant when non-stressed probably due to increased stress sensitivity (Fig. 4d, right panel; t_16_ = 3.020, **p = 0.00814, t-test).

To confirm that the depressive phenotype of right-mPFC^CTGF^ mice is relevant to other depression measures, we used the sucrose preference and forced swim tests as measures of anhedonia and despair, respectively (Fig. 4e). As expected from the results of our social interaction test (Fig. 4d), we observed a significant difference in anhedonia between control and right-mPFC^CTGF^ mice (Fig. 4e left panel; *p = 0.033, T = 54, Mann–Whitney Rank Sum Test). Similarly, the forced swim test showed that right-mPFC^CTGF^ mice exhibited more despair during a forced swim than control mice, as represented by their immobility time (Fig. 4e right panel, *p = 0.0256, t_12_ = -2.547, t-test). The representative video shows that right-mPFC^CTGF^ mice exhibited dramatically more immobile behavior compared to control mice under the stress-naïve condition (Additional file 10: Video 1). These results suggest that CTGF mediates the stress-induced emotional changes in the right mPFC (Fig. 6).

### CTGF overexpression in the left mPFC facilitates social resilience

**Fig. 5:**
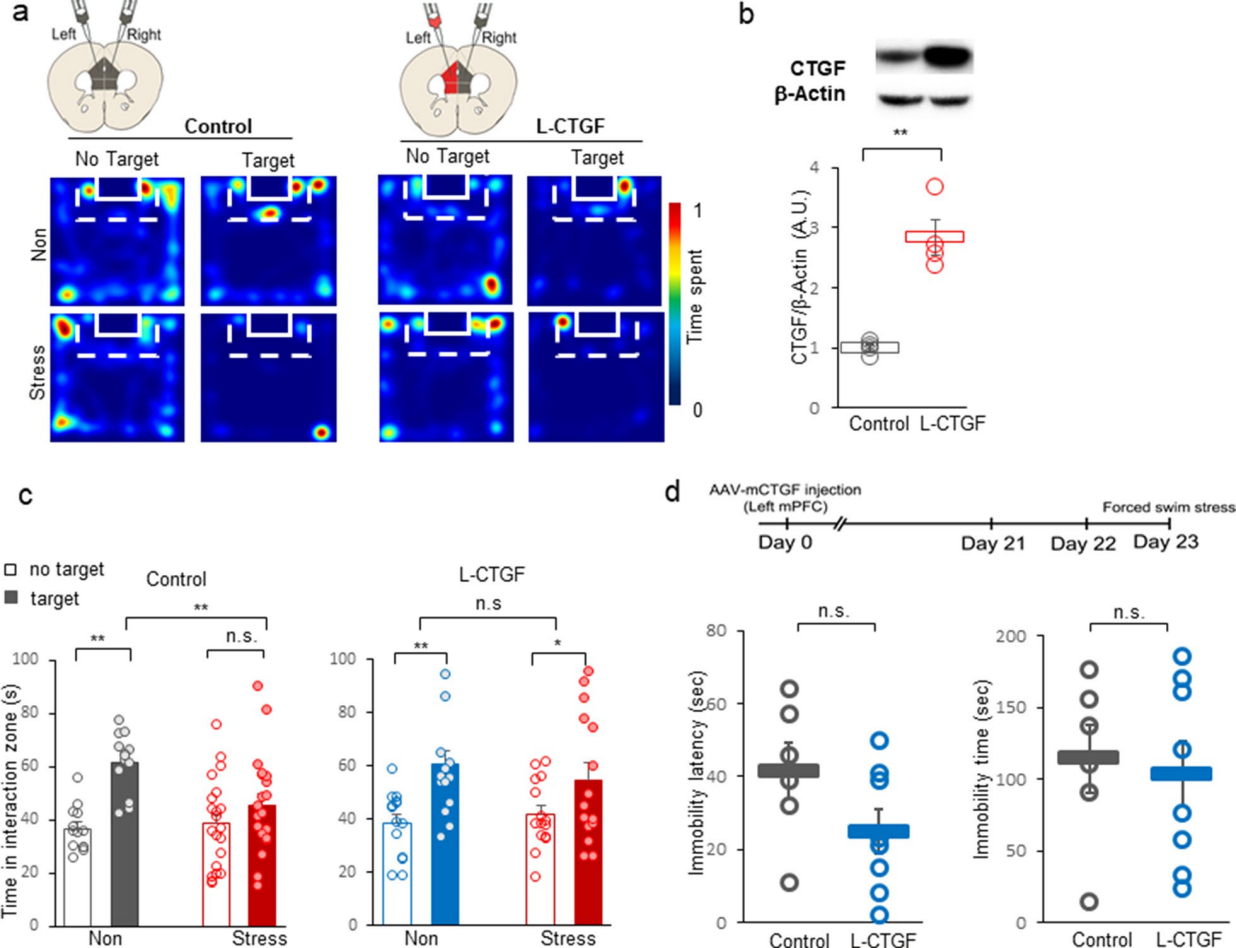
CTGF overexpression in the left mPFC facilitates resilience. **a** Left panel: Representative heatmaps of normalized time spent by mice in the indicated locations without (No Target) and with (Target) a CD-1 target mouse. The upper (Non) and lower (Stress) rows represent mice injected with AAV-mCherry in the left hemisphere of the mPFC (Control). Right panel: Representative heatmaps of normalized time spent by mice in the indicated locations without (No Target) and with (Target) a CD-1 target mouse. The upper (Non) and lower (Stress) rows represent mice injected with AAV-mCTGF in the left hemisphere of the mPFC (L-CTGF). **b** Upper panel: Confirmation of CTGF overexpression by Western blot analysis. Lower panel: Quantitative analysis of CTGF overexpression, presented as the ratio of CTGF/β-actin, as expressed in arbitrary units. Image quantification and analysis were done using ImageJ (**p < 0.01; Control, n = 4; Overexpression, n = 4, t_6_ = -6.142, t-test). **c** Left panel: Interaction times of control mice (Control) with CD-1 mice under non-stressed (Non) and stressed (Stress) conditions, presented as means ± standard deviation. Open bar, time spent in the interaction zone without a target (No target); closed bar, time spent with a target present (Target) (For stress, F(1, 32) = 2.517, *p* = 0.122, target, F(1, 32) = 29.886, **p < 0.001, For stress $\times$ target, F(1, 32) = 9.537, **p = 0.004, two-way RM ANOVA; within non-stressed, the effect of target, **p < 0.001; within stressed, the effect of target, *p* = 0.063; within target presence, effect of stress, **p = 0.004; Post-hoc analysis with Holm-Sidak method). Right panel: Comparison of time spent in the interaction zone by non-stressed (Non) and stressed (Stress) mice with CTGF overexpression in the left mPFC. Interaction times of left-mPFC^CTGF^ mice (L-CTGF) with CD-1 mice under non-stressed and stressed conditions, presented as means ± standard deviation. Open bar, time spent in the interaction zone without a target (No target); closed bar, time spent in the interaction zone when a target is present (Target) (For stress, F(1, 27) = 0.0540, *p* = 0.818, main target effect, F(1, 27) = 20.560, **p < 0.001, stress $\times$ target interaction, F(1, 27) = 0.266, two-way RM ANOVA, within non-stressed, the effect of target,**p < 0.001; within stressed, the effect of target, *p = 0.021; Post-hoc analysis with Holm-Sidak method). **d** Upper: Experimental scheme for investigating the effect of CTGF overexpression in the left mPFC on changes in behavior in response to acute stress (forced swim stress). Lower left panel: Comparison of immobility latency in the forced swim stress (acute stress) between mice with CTGF overexpression in the left mPFC (L-CTGF) and those with injection of control virus in the left mPFC (control). Black circles, control; blue circles, L-CTGF (*p* = 0.110 for Control vs. L-CTGF, t_12_ = 1.728, t-test). Lower right panel: Comparison of immobility time during the last 4 min of the forced swim stress between control and L-CTGF mice (*p* = 0.758 for Control vs. L-CTGF, t_12_ = 0.315, t-test)

After social defeat, resilient mice showed no laterality in CTGF expression, but instead exhibited balanced and increased expression in both mPFC hemispheres (Fig. 3a, b). Considering that the knockout of CTGF in the left mPFC shows stress avoidance to social defeats (Fig. 3f), the increased CTGF in the left mPFC of resilient mice may contribute to enhancing resilience. To test the hypothesis that increased CTGF in the left mPFC made mice socially resilient under social stress, we injected a CTGF-overexpressing viral vector (AAV2/9-CamKIIα-mCTGF) into the left mPFC (Left-mPFC^CTGF^; L-CTGF, Fig. 5). To control for stress-induced social response- and lesion-related effects, we injected a control viral vector (AAV2/5-CamKIIα-mCherry) to the right mPFC (Fig. 5a). As control mice, we injected a control viral vector (AAV2/5-CamKIIα-mCherry) bilaterally into the left and right mPFC. Western blotting confirmed that the CTGF protein level was increased in the left mPFC of L-CTGF mice compared with control mice (Fig. 5b; **p < 0.01, t_6_ = − 6.142, t-test).

After a 10-day social defeat protocol, we measured the sociability value by monitoring the time spent within the interaction zone of a CD-1 target mouse (Fig. 5c). We assessed the sociability of control mice and left-mPFC^CTGF^ mice in the absence (left) and presence (right) of a target mouse (for representative heatmaps, see Fig. 5a, upper panel; non-stressed, lower panel; stressed). Whereas control mice showed increased social avoidance of CD-1 mice (Fig. 5c, left panel; For stress, F(1, 32) = 2.517, *p* = 0.122, target, F(1, 32) = 29.886, **p < 0.001, stress $\times$ target interaction, F(1, 32) = 9.537, **p = 0.004, two-way RM ANOVA; within non-stressed, the effect of target, **p < 0.001; within stressed, the effect of target, *p* = 0.063; within target presence, effect of stress, **p = 0.004; Post-hoc analysis with Holm-Sidak method), Left-mPFC^CTGF^ mice showed no social avoidance (Fig. 5c, right panel; For stress, F(1, 27) = 0.0540, *p* = 0.818, main target effect, F(1, 27) = 20.560, **p < 0.001, stress $\times$ target interaction, F(1, 27) = 0.266, two-way RM ANOVA, within L-CTGF non-stressed, the effect of target,**p < 0.001; within L-CTGF stressed, the effect of target, *p = 0.021; Post-hoc analysis with Holm-Sidak method).

To evaluate the stress coping role of CTGF in acute stress, we measured the latency to immobility after forced swim stress as an acute stress paradigm. Under these conditions, we failed to find any difference in the latency (Fig. 5d left panel, *p* = 0.110, t_12_ = 1.728, t-test) or duration (Fig. 5d right panel, *p* = 0.758, t_12_ = 0.315, t-test) of immobility in Left-mPFC^CTGF^ mice. These results suggest that CTGF expression is specifically involved in the adaptation to chronic stress, which typically leads to emotional and behavioral changes.

These results show the increase of CTGF expression in the left mPFC can critically prevent the development of stress-induced social avoidance (Fig. 5). Without the increase of CTGF in the left mPFC, stressed mice may show depression-like phenotypes as shown in CTGF knockout experiments (Fig. 3).

## Discussion

**Fig. 6:**
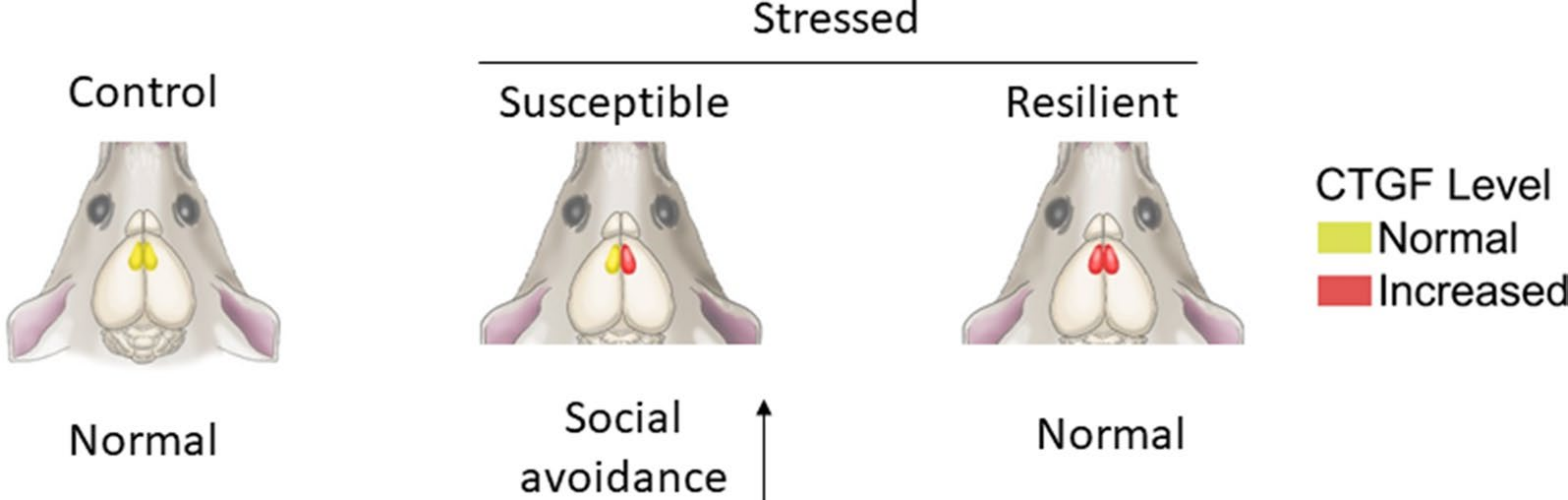
A graphical summary on the hemisphere-specific roles of CTGF. A graphical summary of the hemisphere-specific roles of CTGF in stress adaptation. Non-stressed mice show similar CTGF levels in both mPFC hemispheres. When stressed, mice become susceptible or resilient depending on their CTGF level in each hemisphere: In susceptible mice, CTGF increases only in the right mPFC, while resilient mice show a corresponding increase of CTGF in the left mPFC. Mice with overexpression of CTGF in the right mPFC exhibit an enhanced perception of stress, while mice with overexpression of CTGF in the left mPFC exhibit an increase of the resilient phenotype

Hemispheric lateralization has been implicated in emotional disorders. Transcranial magnetic stimulation (TMS) of the left PFC, which is known to reduce anxiety by retrieving positive memory [37], has been used empirically to treat depression [38–40]. However, the mechanism of PFC lateralization has been poorly studied because of the lack of robust animal models. Our study reveals that chronic social defeat stress induces hemisphere-specific gene expression in the mPFC, opening a new avenue for studying the molecular mechanisms of hemispheric lateralization and stress-induced mental illness (Fig. 6).

### Screening for stress adaptation-related genes by hemisphere-specific analysis

Consistent with previous reports that patients with emotional disorders show lateralization of neuronal activity in the PFC [9, 10], we herein report that the two mPFC hemispheres undergo differential molecular changes under chronic stress conditions (Fig. 1). We identified key molecules that modulate various neuronal functions of stress adaptation, including neuronal degeneration, excitability, synaptic transmission, and cognition (Fig. 2), all of which have been associated with changes induced by chronic stress. In addition, we found that mice exhibiting stress-induced behavioral changes show greater molecular laterality than resilient or control mice, suggesting that laterality score may represent a relevant biomarker for screening stress-modulating genes (Fig. 1).

We identified three groups of stress-related genes. The genes of the first group were altered by stress but did not show any evidence of lateralization. These include *Hba-a1* (hemoglobin alpha, adult chain 1) and *Hbb-b1* (hemoglobin, beta adult major chain), which were increased in both hemispheres of mice exposed to chronic social defeat stress, but not in those exposed to acute stress [12]. Also included in this group is *Arc* (activity-regulated cytoskeleton-associated protein), an immediate early gene that is induced by neural activity and was herein found to be reduced in both hemispheres of stressed mice (Additional file 3: Table S1). This finding is consistent with changes that have been found in postmortem brain tissue of depressed patients and mice exposed to social defeat stress [41]. The second group of genes showed a strong left dominance, as measured by log_2_ L/R values, in response to chronic stress (Fig. 1e, Additional file 6: Table S4). This group included *Adcyap1* (adenylate cyclase activating polypeptide 1, also known as PACAP), which is associated with post-traumatic stress disorder (PTSD) [42]. Notably, suppression of PACAP expression has been shown to reduce corticosterone secretion and depression-like behaviors [43]. The third group of genes showed right dominance, characterized by negative log_2_ L/R values. The genes in this group include *Adra2a* (alpha-2A adrenergic receptor), which was reduced in the left mPFC (Additional file 6: Table S4). Consistent with this, loss of ADRA2A is associated with socially withdrawn behaviors [44], depression and autism [45, 46]; moreover, an increase in ADRA2A has been shown to prevent the withdrawal phenotype and increase novelty-seeking behavior, as observed in ADHD [47]. Increased right mPFC expression of *Ctgf* (Fig. 1e, Additional file 6: Table S4), another group 3, right-dominant gene, was recently observed in the amygdala of an MDD patient [36]. In addition, CTGF levels are reportedly increased in the hippocampus of rats exposed to chronic social defeat stress [48] and in the hippocampus and amygdala of rats exposed to predator-scent stress [49].

Studies have shown that there appear to be gender differences in the functional laterality of the brain [50–52]. While the right vmPFC is involved in socioemotional decision making in males, the left vmPFC is involved in females [51]. Furthermore, females are known to be much more vulnerable to depression and PTSD [53] and show a higher sensitivity in their CRF response [54] than males. Thus, it is plausible that the molecular laterality revealed in our study may provide new insights into gender differences in stress perception and adaptation. Female mice may show more laterality between the two mPFC hemispheres compared to males. As a new social defeat paradigm was recently developed for females [55–57], we could test this hypothesis in a future study.

There have been numerous reports regarding the lateralization of hippocampus under stress conditions [58–61]. Here in this report, we focused on the medial prefrontal cortex, however, there are possibilities of molecular lateralization in other subcortical area such as the hippocampus. With network analysis of regions connected to the mPFC, we may obtain more information about the mechanism of functional laterality induced by emotional stress. Further efforts to study brain regions that are systemically connected to the mPFC will be a future target to consolidate the mechanism of laterality induced by emotional stress.

### CTGF modulates the hemisphere-specific roles of mPFC

In line with the previous finding that right mPFC activity induces stress recognition and left mPFC activity facilitates resilience to chronic stress [9], CTGF was increased in the right mPFC of all stressed mice and in the left mPFC of resilient mice (Fig. 3b). This all-or-none stress information-coding role in the right hemisphere was demonstrated by knockdown and overexpression of CTGF in the mPFC (Fig. 3, 4, 5). Our results showed that right KD mice exhibited increased sociability (Fig. 3g) and right-mPFC^CTGF^ mice exhibited increased stress vulnerability (Fig. 4). The role of the left hemisphere in detecting stress was supported by the decreased sociability of left KD mice (Fig. 3f) and the increased resilience of left-mPFC^CTGF^ mice (Fig. 5c).

The stress sensitivity increment under non-stressed conditions is supported by the increased social avoidance, anhedonia and despair responses in right-mPFC^CTGF^ mice (R-CTGF). (Fig. 4d, e) We can further test the stress sensitivity of R-CTGF mice by measuring corticosterone because this stress hormone is a reliable indicator of stress awareness [62, 63]. Actually, in a pilot study with R-CTGF mice, we found a meaningful stress hormonal increment in the R-CTGF mice under non-stressed conditions indicating that R-CTGF mice were more vulnerable to stress (unpublished data). Further efforts to solve the mechanism of social avoidance of R-CTGF mice might provide answers to this question. We conclude that right-mPFC^CTGF^ mice became depressive even during stress-naïve conditions and are more vulnerable to social stress.

CTGF is known to activate tumor growth factor (TGF)-β signaling through Smad2/3, which in turn, stimulates CTGF expression [64, 65], creating a positive-feedback loop that may contribute to long-term adaptation to chronic stress. Additional studies will be necessary to elucidate the details of this mechanism. Thus, the data from studies on the role of the PFC in emotional control should be interpreted with caution, and any plans for their application must consider the issues of cortical asymmetry and functional divergence. For example, blocking CTGF may alleviate stress perception through a right mPFC-dependent mechanism, but it could harmfully attenuate the pro-resilience effects mediated by the left mPFC. In sum, we herein provide evidence supporting the idea that molecular laterality is a critical mechanism of stress adaptation that encodes information key to the development of stress-related emotional disorders, providing a framework for the potential development of new strategies for treating stress-associated mental illnesses.

## Supplementary Information

**Additional file 1: Figure S1.** Sample preparation for microarrays. a. The distribution of control and socially defeated mice depending on their sociability index (SI). Resilient mice (defined as those exhibiting SI greater than 1) were highly sociable, whereas susceptible mice (defined as those exhibiting SI scores less than 1) were social avoidant. Non-stressed, control mice from social interaction tests showed similar distribution of SI with resilient as previously described [17]. The average SI values for non-defeat control, resilient, and susceptible mice were 1.5, 1.5, and 0.8, respectively. b. The SI values of mice that were selected for the microarray analysis. The average SI of the selected mice were 1.76 (n = 8), 1.61 (n = 8), and 0.5 (n = 7) for the control, resilient, and susceptible groups, respectively. c. Full list of the SI values of the mice used in the microarray analysis.

**Additional file 2: Figure S2.** RT-qPCR confirmation of lateralized genes, categorized according to hemispheric dominance. Changes in the expression levels of left- and right-dominant genes were confirmed by RT-qPCR analysis. a. The *Gapdh*-normalized expression of the left-dominant genes, *Cux2* and *Wfs1*, in the mPFC, presented as a bar graph. The expression levels of *Cux2* and *Wfs1* were higher in the left mPFC of susceptible mice, while the levels of these genes were similar in the left and right mPFC of non-stressed and resilient mice. b. The *Gapdh*-normalized expression of the right-dominant genes, *Ctgf*, *Mbp*, and *Rprm*, in the mPFC. The expression levels of *Ctgf*, *Mbp*, and *Rprm* were higher in the right mPFC of susceptible mice, while the levels of these genes were similar between the left and right mPFC of non-stressed and resilient mice. c. The left/right (L/R) expression ratios of the left-dominant genes, *Cux2* and *Wfs1*, in the mPFC, presented as a bar graph. The ratios of *Cux2* and *Wfs1* were higher in susceptible mice than in non-stressed and resilient mice, whose ratios were similar. d. The L/R expression ratios of the right-dominant genes, *Ctgf*, *Mbp*, and *Rprm*, in the mPFC. The ratios of *Ctgf*, *Mbp*, and *Rprm* were lower in susceptible mice than non-stressed and resilient mice, whose ratios were similar.

**Additional file 3: Table S1.** Analysis of DEGs in the two hemispheres of the mPFC in mice with social defeat stress versus non-stressed mice. Significant DEGs with a FDR adjusted *p*-value cutoff of 0.05 are shown. AveExpr, averaged expression of microarray genes; t, moderated t-statistic; B, B-statistic.

**Additional file 4: Table S2.** List of 526 DEGs with FDR adjusted *p*-value cutoffs of 0.05 corresponding to genes presented in heatmap format in Fig. 1b according to their log2L/R values.

**Additional file 5: Table S3.** Comparison of averaged expression values of DEGs between the two mPFC hemispheres, presented in dot plot format in Fig. 1c.

**Additional file 6: Table S4.** The 526 genes exhibiting laterality listed, according to their log2L/R values. Laterality genes with positive values are expressed more highly in the left mPFC, and those with negative values are expressed more highly in the right mPFC. The genes used in rank order analysis in Fig. 1e.

**Additional file 7: Table S5.** Differences in the expression of the housekeeping genes, *Actb*(b-actin), *Gapdh(*glyceraldehyde-3-phosphate dehydrogenase), *B2m* ((b2microglobinbetween control and resilient/susceptible mice, summarized as log2FC values and *p*-values for each gene.

**Additional file 8: Table S6.** GO analysis using GeneMANIA. Genes with leftward or rightward laterality were used as input to GeneMANIA, and the associated GO terms were reported.

**Additional file 9: Table S7.** Summary of statistics

**Additional file 10: Video S1.** Comparison of immobility time between control and right-mPFC^CTGF^ mice. Representative video showing the difference of immobility time between control and right- mPFC^CTGF^ mice. Mouse behavior was recorded using a video camera, and the total duration of immobility during the last 4 min was analyzed using EthoVision XT (Noldus).

## Data Availability

The raw microarray data are available in NCBI Gene Expression Omnibus at accession number GSE114224.
