# Supplementary figures and images for: Molecular laterality encodes stress susceptibility in the medial prefrontal cortex
Source: Mol Brain. 2021 Jun 14;14:92. doi: 10.1186/s13041-021-00802-w (PMC8201740; doi:10.1186/s13041-021-00802-w)

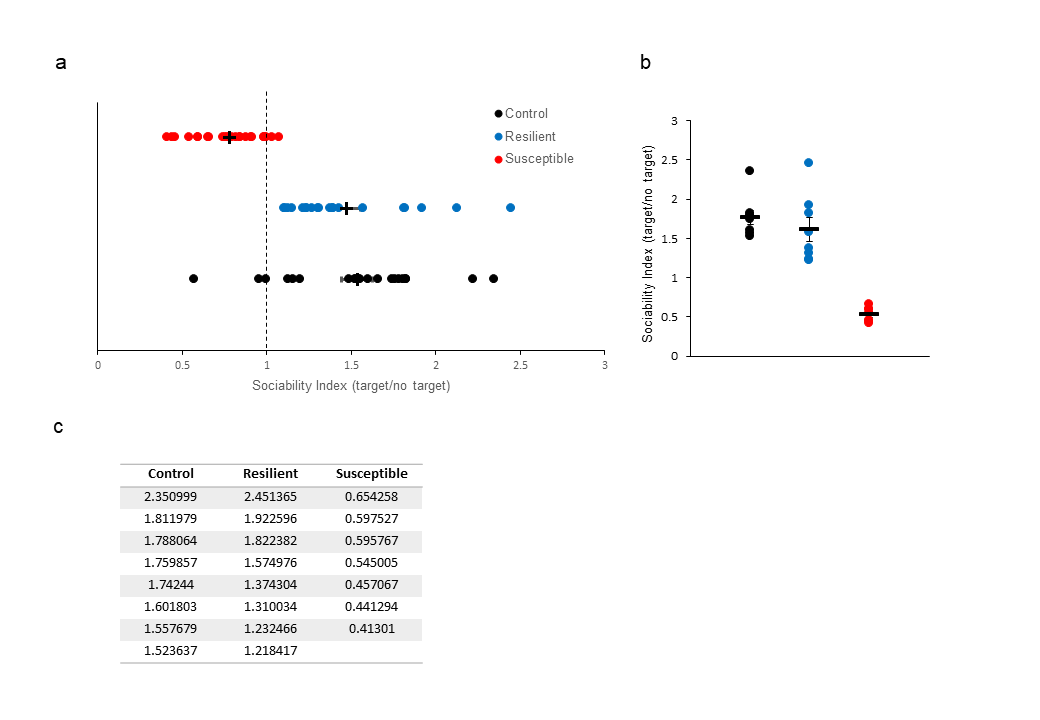

Supplement: Supplementary file 1 — Additional file 1: Figure S1. Sample preparation for microarrays. a. The distribution of control and socially defeated mice depending on their sociability index (SI). Resilient mice (defined as those exhibiting SI greater than 1) were highly sociable, whereas susceptible mice (defined as those exhibiting SI scores less than 1) were social avoidant. Non-stressed, control mice from social interaction tests showed similar distribution of SI with resilient as previously described [17]. The average SI values for non-defeat control, resilient, and susceptible mice were 1.5, 1.5, and 0.8, respectively. b. The SI values of mice that were selected for the microarray analysis. The average SI of the selected mice were 1.76 (n = 8), 1.61 (n = 8), and 0.5 (n = 7) for the control, resilient, and susceptible groups, respectively. c. Full list of the SI values of the mice used in the microarray analysis. [file 13041_2021_802_MOESM1_ESM.tif]

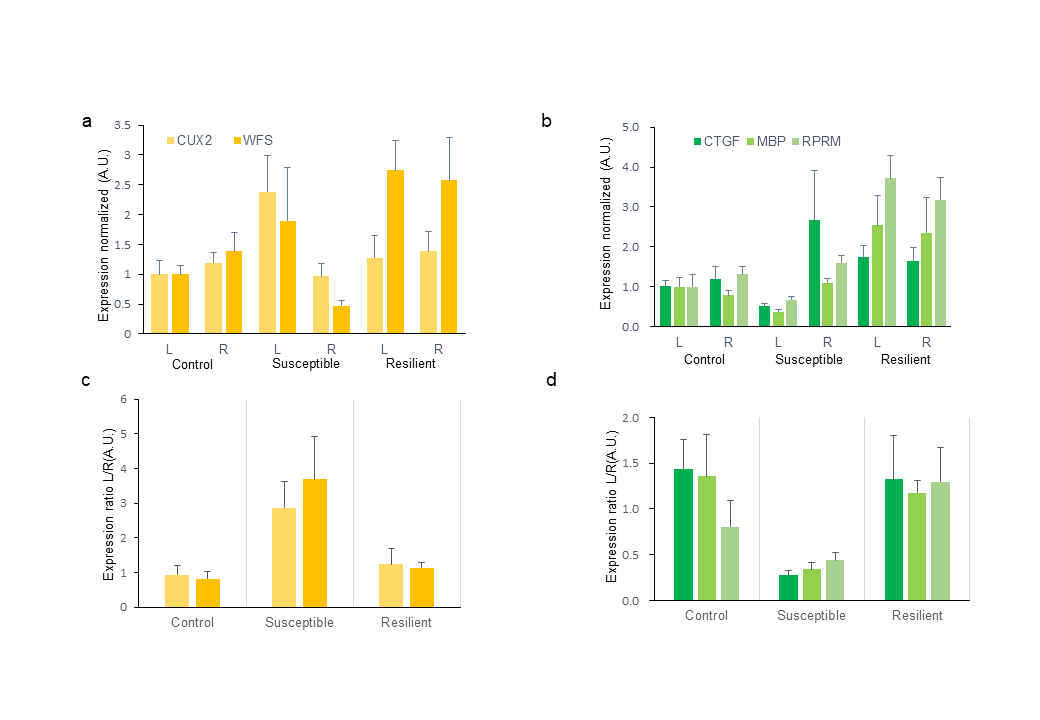

Supplement: Supplementary file 2 — Additional file 2: Figure S2. RT-qPCR confirmation of lateralized genes, categorized according to hemispheric dominance. Changes in the expression levels of left- and right-dominant genes were confirmed by RT-qPCR analysis. a. The Gapdh-normalized expression of the left-dominant genes, Cux2 and Wfs1, in the mPFC, presented as a bar graph. The expression levels of Cux2 and Wfs1 were higher in the left mPFC of susceptible mice, while the levels of these genes were similar in the left and right mPFC of non-stressed and resilient mice. b. The Gapdh-normalized expression of the right-dominant genes, Ctgf, Mbp, and Rprm, in the mPFC. The expression levels of Ctgf, Mbp, and Rprm were higher in the right mPFC of susceptible mice, while the levels of these genes were similar between the left and right mPFC of non-stressed and resilient mice. c. The left/right (L/R) expression ratios of the left-dominant genes, Cux2 and Wfs1, in the mPFC, presented as a bar graph. The ratios of Cux2 and Wfs1 were higher in susceptible mice than in non-stressed and resilient mice, whose ratios were similar. d. The L/R expression ratios of the right-dominant genes, Ctgf, Mbp, and Rprm, in the mPFC. The ratios of Ctgf, Mbp, and Rprm were lower in susceptible mice than non-stressed and resilient mice, whose ratios were similar. [file 13041_2021_802_MOESM2_ESM.tif]
