# Supplementary material for: Molecular laterality encodes stress susceptibility in the medial prefrontal cortex
Source: Mol Brain. 2021 Jun 14;14:92. doi: 10.1186/s13041-021-00802-w (PMC8201740; doi:10.1186/s13041-021-00802-w)
Supplement: Supplementary file 3 — Additional file 3: Table S1. Analysis of DEGs in the two hemispheres of the mPFC in mice with social defeat stress versus non-stressed mice. Significant DEGs with a FDR adjusted p-value cutoff of 0.05 are shown. AveExpr, averaged expression of microarray genes; t, moderated t-statistic; B, B-statistic. [file 13041_2021_802_MOESM3_ESM.pdf]

Supplementary table1
Analysis of DEGs in the two hemispheres of the mPFC in mice with social defeat stress versus non-stressed mice. Significant DEGs with a FDR adjusted *p*-value cutoff of 0.05 are shown. AveExpr, averaged expression of microarray genes; t, moderated t-statistic; B, B-statistic.

DEGs in Stressed mice

adj.P.Value	< 0.05
DEGup	265
DEGdown	314

Gene	logFC	AveExpr	t	P.Value	adj.P.Val	B
HBA-A1	0.84707041	12.603948	8.7445231	7.36E-08	3.79E-04	8.15152952
HBB-B1	0.46623188	8.799639	5.4034457	4.06E-05	0.00541842	2.3301883
SLA	0.45443905	8.89092299	4.5322105	0.00026452	0.01484816	0.55489898
COL6A1	0.35226339	9.22545339	4.45290944	0.00031477	0.0158282	0.39003821
AHI1	0.33521924	10.5770826	8.36220121	1.41E-07	0.00037903	7.57222957
FDPS	0.31547909	10.8318761	6.49685384	4.36E-06	2.42E-03	4.42350064
SGK1	0.31406315	11.4479198	7.80398581	3.74E-07	6.59E-04	6.68677525
PFDN2	0.29616089	11.0416885	4.55956876	0.00024914	0.01437426	0.61168241
NTSR1	0.28754149	8.08729358	4.66064059	0.00019977	0.01269972	0.82100826
CCDC85B	0.28441768	10.2665489	4.81746377	0.00014204	0.0106406	1.14421177
CRYM	0.27706991	11.5469814	5.04613119	8.67E-05	0.00874856	1.61144447
CDH13	0.2708336	8.43969677	8.34963357	1.44E-07	0.00037903	7.55281646
RASL10A	0.26866073	9.58820196	7.19824142	1.14E-06	1.27E-03	5.67128602
FEZF2	0.26701969	9.72472105	6.27530904	6.77E-06	0.00259952	4.01348915
GRP	0.26655555	8.92596053	4.29526966	0.00044531	0.01910341	0.0613068
COL5A1	0.26414787	8.05294259	6.75563963	2.63E-06	1.84E-03	4.89284883
STK32C	0.25945005	9.13738682	4.48840763	0.00029118	0.01546967	0.46388353
FUS	0.25826366	8.26822029	4.379662	0.00036977	0.01695854	0.23744331
RAMP3	0.25743935	8.3924463	6.23155596	7.39E-06	0.00259952	3.93163287
SEZ6	0.25585236	9.33471543	3.83934395	0.00122152	0.0317664	-0.8931805
DLG4	0.25270207	9.49692496	5.70235163	2.17E-05	0.00437931	2.91925099
ALAS2	0.25225822	7.583163	3.73460994	0.00154087	0.0363793	-1.1122626
PTPRU	0.25137647	8.32085233	4.60333318	0.00022639	0.01381007	0.70241164
KLF16	0.2510609	8.78199347	5.37421553	4.31E-05	0.00562063	2.27196278
LY6A	0.24998295	9.56547165	6.20196164	7.84E-06	2.67E-03	3.87610236
STXBP2	0.23797724	9.00951879	4.43217412	0.00032944	0.01602116	0.34686994
COTL1	0.23749955	9.64508114	5.11537348	7.48E-05	0.00821991	1.75186859
RPS26	0.23700543	11.8522756	5.50287672	3.29E-05	4.82E-03	2.52744135
DPYSL5	0.23180537	7.95106553	4.40045113	0.00035324	0.01656753	0.28078151
DGKG	0.22768583	10.633969	6.37649876	5.54E-06	2.60E-03	4.20169067

SLC35E3	0.22445198	9.52768232	4.77129674	0.00015701	0.01069161	1.04928006
SUMO3	0.22403948	9.24110329	4.36564893	0.00038135	0.01734638	0.20821882
SAMD14	0.22184603	9.19400368	4.51858052	0.00027254	0.01500795	0.526591
SYT5	0.22182225	10.3186048	5.52716269	3.13E-05	4.82E-03	2.57542667
PRR7	0.22168804	9.3840363	7.06847588	1.45E-06	1.27E-03	5.44624908
STMN4	0.22083269	9.66088678	4.77805956	0.00015472	0.01069161	1.06319789
ENSMUSG00	0.21900688	7.88298176	3.89094426	0.00108944	0.03017556	-0.7851365
NRBP2	0.218318	10.2750614	4.85832667	0.00013001	0.01035803	1.22807708
KLF5	0.21546766	8.79053829	4.61367067	0.00022134	1.36E-02	0.72382281
HRMT1L2	0.21533176	10.4844888	6.07342188	1.02E-05	0.00291503	3.63339325
RPS4Y2	0.21380394	7.95516996	5.70384181	2.17E-05	0.00437931	2.92215777
DDAH1	0.21379253	11.2813149	3.65080334	0.00185541	0.04009013	-1.2872544
PADI6	0.21296523	7.45104214	3.98994433	0.00087477	0.02660348	-0.5777475
VPS25	0.21160752	8.98146554	5.82681103	1.68E-05	0.00374295	3.16096366
ARHGEF15	0.21079644	8.08675003	6.29914158	6.46E-06	0.00259952	4.05795499
NME2	0.21061356	10.329742	4.29698705	0.00044363	1.91E-02	0.06489424
P140	0.21041299	8.30535316	6.01550195	1.14E-05	3.04E-03	3.52322973
ACTR1A	0.21020477	10.8298495	3.73502912	0.00153944	0.0363793	-1.1113865
HNRNPK	0.20511571	8.5206578	5.59277992	2.73E-05	0.00453347	2.70468832
KCTD10	0.20283557	9.086462	5.08430976	7.99E-05	8.51E-03	1.68893531
HSPA8	0.20017484	12.0776926	3.6713111	0.00177298	0.03946587	-1.2444654
CASC3	0.19892698	10.0445341	4.05915943	0.00075042	0.02525567	-0.4327417
GPN3	0.19885025	8.40650612	5.08116857	8.05E-05	0.00851403	1.68256558
MED6	0.19817141	8.93803399	6.7257443	2.79E-06	1.84E-03	4.83916156
ASAH3L	0.19808508	7.96930731	3.58369733	0.00215272	0.04335436	-1.4270973
DDR1	0.19561391	8.7460174	4.41669929	0.00034084	0.01632144	0.31463796
5430437P03I	0.19478618	9.35244513	4.61662588	0.00021991	0.01357163	0.72994225
SLC38A5	0.19406949	8.13011731	4.55257755	0.00025298	0.01450913	0.59717655
CXX1C	0.19204047	10.4289732	5.13589761	7.16E-05	8.03E-03	1.79339095
ERP29	0.19081977	11.3256306	4.45423017	0.00031386	0.0158282	0.39278699
FXYD6	0.19034592	12.8022387	3.60980779	0.00203179	0.04195989	-1.3727188
ZYX	0.1874176	11.0047353	3.76447944	0.00144212	0.03559497	-1.049819
ANAPC5	0.18696912	10.0035277	4.05071732	0.00076458	0.02525567	-0.4504265
TSC22D1	0.18617797	9.09787686	4.34373051	0.0004002	0.01789556	0.16248902
EIF3D	0.18573016	10.4730926	4.97884225	0.00010023	0.00936197	1.47449411
PDE1B	0.18546282	10.8763249	4.44890056	0.00031756	0.0158282	0.38169413
RASAL1	0.18483579	8.86119083	4.31763245	0.00042389	0.01879399	0.10801077
ZER1	0.18444604	12.3539291	4.34602411	0.00039819	0.01788115	0.16727533
2610208M17	0.18365454	9.26142735	4.40864945	0.00034692	0.01634416	0.29786614
D430041B17	0.18326537	9.12323543	5.63343155	2.51E-05	0.00453347	2.78448168
LOC1000450	0.18307323	11.2231454	4.44499351	0.00032029	0.01586875	0.37356111
PPARGC1B	0.18173284	9.53128162	4.68937558	0.00018764	0.01214815	0.88038075
TCEAL3	0.18108658	8.92495809	4.49482976	0.0002871	0.01545824	0.47723517

MMP24	0.18031522	9.0457707	5.00856376	9.40E-05	0.00902024	1.53504303
LMO3	0.18017455	8.37342408	4.18809319	0.00056418	0.02165034	-0.1627787
ARL6IP1	0.17875161	12.5945866	4.05260063	0.00076139	0.02525567	-0.4464812
LOC1000476	0.1780045	10.4896882	3.90158311	0.00106404	0.02986396	-0.7628545
WSCD1	0.1779186	8.90817941	3.58746022	0.00213486	0.0430769	-1.4192634
1500011H22	0.17748554	10.1429446	4.04425878	0.00077559	0.02526478	-0.4639564
TATDN2	0.17665222	8.41013248	5.22501194	5.92E-05	7.18E-03	1.97312615
SCAMP2	0.17556517	8.77843666	3.98613375	0.00088218	0.02675199	-0.5857311
EG434858	0.17550985	8.69900991	4.37788541	0.00037121	0.01695854	0.23373872
EXTL1	0.17547483	8.80908867	5.18747553	6.41E-05	7.52E-03	1.89752944
DYNC1H1	0.17521284	10.2921665	3.83003193	0.00124701	0.03217518	-0.9126725
LMAN2L	0.17513689	9.21099533	4.48755041	0.00029173	0.01546967	0.46210117
6430598A04	0.17501123	8.39333158	4.89579904	0.00011989	0.00980784	1.3048473
LOC1000465	0.1748415	11.7565652	3.67584536	0.00175525	0.03938727	-1.2350018
TMSB10	0.17310779	11.0622247	3.65311846	0.00184592	0.03999989	-1.2824251
ARL2	0.17218214	9.19495199	3.80673413	0.00131313	0.03357103	-0.96143
LOR	0.17210107	9.26875914	4.78143542	0.00015359	0.01069161	1.07014392
GABBR1	0.17201197	8.46303816	4.87135621	0.0001264	0.0102605	1.25478592
RPS27L	0.1713086	10.4015661	3.52660898	0.00244259	4.70E-02	-1.5458248
CLPP	0.17119424	8.90672552	4.62275276	0.00021699	0.01353358	0.74262735
DBR1	0.17066401	8.70835369	3.99233752	0.00087014	0.02660348	-0.5727336
HSF1	0.17062884	9.89883323	4.91013159	0.00011624	0.0096586	1.33417525
SLC25A44	0.16956409	9.87377288	4.26102381	0.00048026	0.01979244	-0.0102524
MED30	0.16923612	10.0565877	4.74351789	0.00016678	0.01113939	0.9920702
MAGI2	0.1691031	7.58313211	4.92148768	0.00011342	9.58E-03	1.3573985
COG1	0.16909895	8.32080877	5.48986172	3.38E-05	4.82E-03	2.50169414
BEX4	0.16890352	9.10806379	4.79049512	0.0001506	0.01069161	1.08877983
FAM116B	0.16876119	9.60426378	4.39231913	0.00035961	1.67E-02	0.26383168
STARD3NL	0.16860838	8.99338753	4.74831137	0.00016505	0.01109412	1.00194694
ERH	0.16736157	10.7233644	3.54521086	0.00234412	0.04574792	-1.5071648
PRMT2	0.16687455	11.1476029	3.77210678	0.00141794	3.52E-02	-1.0338684
METTL3	0.1664318	9.36895529	4.51377378	0.00027542	0.01500795	0.5166051
2700094K13I	0.16634583	10.635795	4.78878655	0.00015116	0.01069161	1.08526583
PSME2	0.1636671	7.75933121	4.72746289	0.00017271	0.0113203	0.95897565
SLC9A3R2	0.16340613	8.86538615	3.51717751	0.00249407	0.04765765	-1.5654158
ARPC5	0.16295467	11.1296861	3.92184969	0.00101729	0.02925189	-0.7204039
DRCTNNB1A	0.16293861	8.36220791	4.2572087	0.00048432	0.01979244	-0.018227
1700027N10	0.16283907	8.01199439	3.99408059	0.00086678	0.02660348	-0.5690816
CBLN1	0.16219685	8.54147333	3.51164589	0.00252477	0.04774887	-1.5769026
LSM8	0.16203568	9.56242039	3.88765397	0.00109742	0.0302378	-0.7920274
MID1	0.16179284	7.85526935	3.88519426	0.00110342	0.03024524	-0.7971787
WDR61	0.16166385	9.83441411	3.75252231	0.00148087	0.03590904	-1.07482
CLTA	0.16087618	10.2301261	3.64775913	0.00186797	4.01E-02	-1.2936041

LASS5	0.16067632	9.45211093	4.25136442	0.0004906	0.01991287	-0.0304441
EG633640	0.16063123	7.72314209	5.21084281	6.10E-05	7.23E-03	1.9446093
ATP2B1	0.15946217	9.34355751	4.02743305	0.00080504	0.0258163	-0.4992058
LOC1000461	0.15905697	8.01197679	4.28744387	0.00045306	0.01930163	0.04495817
EEF2	0.15875319	8.35025708	3.67769564	0.00174807	3.93E-02	-1.2311397
VTI1B	0.15870814	12.2250299	4.18961211	0.00056229	0.02165034	-0.1596004
UHRF2	0.1586985	9.13106673	4.9426383	0.00010836	0.00954633	1.4006179
G3BP1	0.15838604	8.93058992	4.1420367	0.00062466	0.02265307	-0.2591763
RCL1	0.15806718	8.42388567	4.83714759	0.00013611	0.01047438	1.18462908
CALN1	0.15614126	12.0283157	4.08694837	0.00070564	0.0244688	-0.3745338
DUS4L	0.15590533	8.41962591	5.51861634	3.18E-05	0.00482368	2.55854919
LSM3	0.1550127	9.23406093	4.0081901	0.0008401	0.02626501	-0.5395208
MAPK1	0.15488021	11.321404	4.92662293	0.00011217	0.00954633	1.36789597
B9D1	0.15457714	8.63456699	4.16480825	0.00059398	0.02207153	-0.2115085
SYVN1	0.1545448	10.5735392	4.81347911	0.00014328	0.0106406	1.1360257
DENND2A	0.15444509	8.29597454	3.83541975	0.00123219	0.03187095	-0.9013949
GSPT1	0.15355112	10.8230872	4.77377318	0.00015617	0.01069161	1.05437701
LOC1000482	0.15320556	9.89374789	3.75029931	0.00148819	0.03593784	-1.0794675
VWF	0.15313558	7.58027007	4.28691072	0.0004536	0.01930163	0.04384429
KLF13	0.15303162	10.3038807	3.49255542	0.0026336	4.87E-02	-1.6165261
UQCRC2	0.15298275	12.555152	3.9499884	0.00095577	0.0283173	-0.6614573
ATG16L1	0.15290626	10.1351094	4.73286764	0.00017069	0.0113203	0.97011898
4931406P16I	0.15271542	9.15732291	3.89820759	0.00107204	0.03000851	-0.7699244
ZFP672	0.15246462	9.711252	4.30823505	0.00043276	0.01879399	0.08838709
SMN1	0.15240586	10.0363525	3.66839605	0.00178447	3.95E-02	-1.250549
SUHW4	0.1520486	9.01608947	4.0607486	0.00074778	0.02525567	-0.4294127
ABHD14B	0.15182283	9.13086514	3.54994954	0.00231967	0.04544954	-1.4973122
EXOSC7	0.1515962	10.0604989	4.51424	0.00027514	0.01500795	0.51757373
SLC8A2	0.15132136	8.4344837	3.51416753	0.00251073	0.04774005	-1.5716666
H3F3A	0.15114808	10.167176	4.19453755	0.00055621	0.02165034	-0.1492947
ORC6L	0.15081333	9.61310736	4.45654432	0.00031227	0.0158282	0.39760307
MAS1	0.15080326	8.79785389	4.18398836	0.00056933	0.02173783	-0.1713681
GM1821	0.15080022	14.5101826	4.52208117	0.00027045	1.50E-02	0.5338626
LOC1000483	0.1502078	8.75074611	3.78367923	0.00138201	3.47E-02	-1.0096638
PACRG	0.14966189	9.41480311	4.07544106	0.00072384	0.02470159	-0.3986364
DMAP1	0.14915744	8.75628093	4.11055807	0.00066971	0.02387662	-0.3250878
MRPL10	0.14898598	10.1693607	4.10754522	0.00067419	0.02390011	-0.3313971
TDRD7	0.14880619	9.56353556	4.78873204	0.00015118	1.07E-02	1.08515372
COPZ1	0.1482163	9.98079258	3.671027	0.0017741	0.03946587	-1.2450584
ZFP282	0.14702639	8.66786284	4.35688025	0.00038878	0.01753335	0.18992684
PIGK	0.1469043	7.89787302	4.85643928	0.00013054	0.01035803	1.22420685
POLD2	0.14683051	8.16933594	3.80906568	0.00130636	0.03354252	-0.9565512
LOC1000462	0.1465748	11.8201642	3.50230234	0.00257747	4.82E-02	-1.5962996

ING4	0.14646713	8.67002446	4.43601875	0.00032667	0.01595994	0.35487579
TMEM147	0.145731	11.1240551	4.01030226	0.00083618	0.02626261	-0.5350957
TUBB5	0.1455329	13.4749551	4.07889141	0.00071834	0.02469253	-0.3914093
HDGF	0.14508715	10.1173034	3.66018831	0.00181722	0.03970426	-1.2676757
PRDM4	0.14378747	9.50469255	5.28865781	5.17E-05	0.00650479	2.10092893
THOC4	0.14338703	9.26818632	4.81636825	0.00014238	0.0106406	1.14196127
CACNB3	0.14319773	8.97196061	3.66554292	0.00179579	3.95E-02	-1.2565029
ZFP316	0.14160263	7.80156754	4.57357662	0.00024162	0.01437426	0.64073697
DPYSL3	0.14158218	8.24719702	3.62441838	0.0019671	0.0409443	-1.3422713
WBP2	0.14157056	14.1376787	3.88072114	0.00111442	0.0303973	-0.8065463
RBBP7	0.14134727	8.38107256	5.55405614	2.96E-05	0.00472836	2.62847383
EXOC4	0.13968842	8.78500308	4.13825186	0.00062991	0.02276527	-0.2671002
XAB1	0.13935095	9.11144955	4.06205906	0.00074561	0.02525567	-0.4266676
BC002163	0.13903927	11.4785126	3.51658141	0.00249736	0.04765765	-1.5666537
GRIA1	0.13860623	10.182572	4.1190012	0.00065732	0.02351413	-0.3074074
PHF5A	0.13831598	10.4358163	4.8354479	0.00013661	0.01047438	1.18114047
A130092J06F	0.13819943	8.71973404	3.48722922	0.00266478	4.90E-02	-1.6275755
PYCR2	0.13789917	9.02437896	3.65727996	0.00182897	3.98E-02	-1.2737435
JTV1	0.13789657	8.56783898	4.38242365	0.00036753	0.01693666	0.24320164
RPS27A	0.13709563	9.83155915	4.15212919	0.00061087	0.0223838	-0.2380482
ILK	0.13705113	9.88498295	4.2952632	0.00044532	0.01910341	0.06129331
MUM1	0.13653245	9.76793586	4.31230437	0.0004289	0.01879399	0.09688512
DHRS7B	0.13641495	8.68033067	4.93295395	0.00011065	0.00954633	1.38083426
ZCCHC18	0.13633356	12.8984495	4.51849634	0.00027259	0.01500795	0.52641613
EGFL7	0.13588784	8.76865683	3.63598862	0.00191732	0.04036789	-1.3181505
CASKIN1	0.1357915	9.5293451	4.44118308	0.00032298	0.01592736	0.36562839
KHDRBS1	0.13508343	8.73762982	3.7841345	0.00138062	0.03472466	-1.0087114
CHMP4B	0.13478102	9.64887237	3.99911639	0.00085717	0.02652691	-0.5585311
GDPD1	0.13434244	10.0910966	3.98005613	0.00089415	0.0268174	-0.5984643
2010003O18	0.13426418	9.81555909	3.59665254	0.00209185	0.04261645	-1.4001219
IFITM2	0.13408053	7.77998072	3.53223649	0.00241238	0.04671158	-1.5341321
ZNRD1	0.13379869	9.2295584	4.41541492	0.0003418	0.01632144	0.3119622
CBFA2T3H	0.13351573	8.34434919	4.27906552	0.00046151	0.01955849	0.02745254
ORF61	0.13284415	9.49859862	3.94895808	0.00095795	0.0283173	-0.6636158
RPS4X	0.13261574	12.7098057	3.54415484	0.0023496	0.04574792	-1.5093602
D1BWG0212	0.13206621	8.26317149	3.93340914	0.00099155	0.02882594	-0.6961893
NT5C3	0.13192345	9.61698733	3.69706555	0.00167461	3.81E-02	-1.1906986
MYCBP2	0.13175871	9.80120915	4.0142973	0.00082881	0.02626261	-0.5267258
EDNRA	0.13153306	7.80287202	3.78719539	0.00137128	0.03461981	-1.0023086
ATP5C1	0.131482	12.6317191	4.090587	0.00069998	0.02445981	-0.3669128
SETD1A	0.12976273	8.68510078	3.53406829	0.00240262	0.04660821	-1.5303255
SNRP70	0.12905868	8.89491785	3.648184	0.00186621	0.04009013	-1.2927179
SPRN	0.12881875	8.00073114	3.84059845	0.00121812	0.0317664	-0.8905544

RANBP1	0.1287717	10.3455873	4.1733601	0.00058286	0.0219656	-0.19361
INTS4	0.12812206	9.30587442	3.53828477	0.00238032	4.63E-02	-1.5215623
THOC5	0.12752478	8.78609979	4.05114278	0.00076386	0.02525567	-0.4495352
MORF4L2	0.12689124	13.1272204	3.73004229	0.00155655	3.66E-02	-1.1218084
TSTA3	0.12676568	9.79831986	3.79814357	0.00133838	0.03403366	-0.9794044
SMARCD2	0.12676168	8.1412462	3.90227816	0.0010624	0.02986396	-0.7613987
EMD	0.12628669	8.15997895	5.04701708	8.66E-05	0.00874856	1.61324433
AMZ2	0.12620645	9.30685158	3.74075962	0.00152	0.03633807	-1.0994092
FBXL6	0.12550764	8.62592373	4.23613961	0.00050737	0.02020496	-0.0622761
UBE2L3	0.12546883	11.2301617	3.54962099	0.00232136	0.04544954	-1.4979954
ZFX	0.12541529	7.98917047	4.53359055	0.00026372	0.01484816	0.55776453
PNKP	0.12478613	8.65915579	4.48909354	0.00029074	0.01546967	0.46530966
GSTP1	0.12433132	10.3660904	3.89254989	0.00108557	0.03014745	-0.7817738
MAPK11	0.1236623	8.74313207	3.52765468	0.00243695	0.04695976	-1.5436523
ST5	0.12331556	8.93081243	4.25906266	0.00048234	0.01979244	-0.0143517
ANKRD39	0.12329439	8.27174011	4.17356684	0.00058259	0.0219656	-0.1931773
SERBP1	0.1226573	10.5685327	3.92468474	0.00101091	0.02923188	-0.7144652
SYF2	0.12262014	9.39961167	4.53896673	0.00026063	0.01478734	0.56892647
NARG1L	0.12257279	7.90714917	4.5637677	0.00024686	0.01437426	0.62039309
LOC1000467	0.12247182	8.63062001	3.49243003	0.00263433	4.87E-02	-1.6167863
BRD9	0.12238066	10.2081159	3.79409862	0.00135044	0.03425777	-0.9878671
VAPB	0.12152226	9.06547337	3.93647998	0.00098482	0.02878278	-0.6897564
HDGFRP2	0.12151996	10.8939024	3.63663135	0.00191459	0.04036789	-1.3168103
KCNIP4	0.1211028	7.68505453	4.02727199	0.00080533	0.0258163	-0.4995432
RAB9B	0.12034952	9.8051588	3.70123039	0.00165922	0.037818	-1.1820007
MTX1	0.11980733	9.24712805	4.10209419	0.00068238	0.02392393	-0.3428128
PTK2	0.1177899	11.1997155	4.35765996	0.00038811	0.01753335	0.19155351
TOMM34	0.11767693	9.34356139	3.59995624	0.00207661	0.04247652	-1.3932412
RALY	0.1167667	9.37994488	3.51911561	0.00248341	0.04756826	-1.5613906
WRB	0.11669248	9.79492227	4.1611704	0.00059878	0.02216078	-0.2191229
NUMA1	0.1165107	7.73571399	3.50291871	0.00257396	0.04822693	-1.5950202
CYB5R3	0.11619442	8.69717134	3.57680197	0.00218583	0.04360513	-1.4414502
RASSF1	0.11578866	8.30912044	3.64935852	0.00186136	0.04009013	-1.2902681
SSBP2	0.11575743	9.14415591	3.48675503	0.00266758	0.04904346	-1.628559
MRPL46	0.11394568	9.52579684	4.27360288	0.00046711	0.01962085	0.01603757
RUVBL1	0.11391366	8.72750126	4.81904183	0.00014156	0.0106406	1.14745334
ATG9B	0.11348257	7.99402137	3.63256099	0.00193193	0.04037166	-1.325297
FOXK1	0.11298991	7.4618419	3.59315846	0.0021081	0.04278225	-1.4073984
ZFP295	0.11185828	8.17988314	4.60030772	0.0002279	0.0138218	0.6961438
RPA3	0.10951004	9.12019417	3.87767554	0.00112197	0.03051595	-0.8129242
TDRKH	0.10906305	8.44437328	3.47832844	0.00271772	0.04961946	-1.6460348
ACTB	0.10845888	12.1140291	3.63929549	0.00190332	4.03E-02	-1.3112551
TMED4	0.10825167	9.12854136	4.39547476	0.00035712	0.01666395	0.27040947

SLC2A3	0.10673457	11.3323739	3.72964287	0.00155793	0.03656519	-1.1226431
MBD3L2	0.10654811	7.39180782	3.49408431	0.00262471	0.04867947	-1.6133539
TSC22D2	0.10649417	9.54903723	4.15980907	0.00060059	0.02216078	-0.2219723
RPS6KA5	0.10636275	7.95177987	4.05052646	0.0007649	0.02525567	-0.4508263
NFRKB	0.10627811	8.21998627	3.59099432	0.00211822	4.28E-02	-1.4119049
IL18BP	0.10540903	7.45261636	3.54467464	0.0023469	4.57E-02	-1.5082796
5133400G04	0.10364604	7.86018462	4.1928654	0.00055827	0.02165034	-0.1527933
LONRF2	0.10325084	7.96812964	4.14238597	0.00062418	0.02265307	-0.2584451
LOC1000480	0.10252092	8.84783397	3.66081519	0.0018147	3.97E-02	-1.2663677
MADD	0.10251097	7.55477999	3.87341774	0.00113262	0.03064746	-0.8218403
DCTN4	0.10127044	10.0855676	3.68302901	0.00172753	0.03903767	-1.2200064
CEBPB	0.1005204	9.00095948	4.00179884	0.00085209	0.02647864	-0.5529111
GMDS	0.10011521	7.77673895	3.77224146	0.00141751	0.03520817	-1.0335867
SLC35B3	0.10003218	8.70036831	3.7688044	0.00142836	0.0353837	-1.0407747
SIN3A	0.09401916	8.71878523	3.63927799	0.0019034	0.04032348	-1.3112916
MED26	0.09379981	8.2156832	3.74286936	0.00151291	0.0362857	-1.0949994
KCNC1	0.09348525	9.31369903	3.57714707	0.00218417	0.04360513	-1.4407319
LOC1000483	0.09264571	8.77680998	3.64295749	0.00188794	0.04024431	-1.3036184
GPR27	0.09084794	7.52805571	3.6474913	0.00186907	0.04009013	-1.2941627
LOC1000454	0.08998439	7.75949744	3.75355716	0.00147748	0.03590904	-1.0726565
6430526O11	-0.0803317	7.32928631	-3.5599261	0.00226902	0.04475701	-1.4765636
LOC1000460	-0.0939378	8.06551448	-3.6443909	0.00188196	0.04020305	-1.3006291
GJD3	-0.0950662	7.21793288	-3.5775897	0.00218203	0.04360513	-1.4398106
BBS2	-0.0991047	8.23760203	-3.5640847	0.00224824	0.04451339	-1.4679127
MAPK10	-0.100847	8.0639822	-3.6957951	0.00167933	0.03811177	-1.1933517
PCMTD1	-0.1037211	7.85396725	-3.6751581	0.00175793	0.03938727	-1.2364362
KCNG4	-0.1043834	7.6080946	-3.5605001	0.00226614	0.04475701	-1.4753697
AFAP1L1	-0.1062162	7.4500965	-3.6719233	0.00177058	0.03946587	-1.2431878
LOC1000424	-0.1063231	13.8043571	-3.7104017	0.00162583	0.03737982	-1.1628444
9130213B05I	-0.1069077	9.48811239	-3.5818103	0.00216173	4.34E-02	-1.4310256
SNF8	-0.1070575	7.81359008	-3.6038092	0.00205896	0.04235524	-1.3852155
NDUFS4	-0.108737	10.9825778	-3.7183094	0.00159757	0.03703067	-1.1463246
TPRKB	-0.1087425	8.81131795	-3.4847138	0.00267964	4.92E-02	-1.6327929
SUPT5H	-0.1098681	9.74623783	-3.7640075	0.00144363	0.03559497	-1.050806
RIC8B	-0.1117321	9.07147682	-3.6107304	0.00202765	0.04195989	-1.3707965
SNX25	-0.1119011	8.23030215	-4.4115401	0.00034473	0.01634416	0.30388912
PREP	-0.1122768	8.26468362	-3.9402871	0.00097654	0.02862632	-0.6817809
EDNRB	-0.1127842	9.17500696	-3.5998849	0.00207693	0.04247652	-1.3933899
KIFAP3	-0.1128841	11.2770488	-4.0476034	0.00076987	0.02526478	-0.4569498
SPINK10	-0.1139919	7.61191093	-3.7051227	0.00164496	3.76E-02	-1.1738713
MTERFD3	-0.114198	7.85938397	-3.7175929	0.00160011	0.03703067	-1.1478215
ZCCHC17	-0.1142747	9.24662207	-3.4967327	0.00260939	0.04848053	-1.6078585
5730410I19R	-0.1143842	9.27546424	-3.751694	0.00148359	0.03590904	-1.0765517

NOL14	-0.1146011	8.14815321	-3.5100292	0.00253381	0.04783414	-1.5802593
TMEM209	-0.1147492	8.83965484	-3.6694938	0.00178013	0.03946587	-1.2482582
ABHD6	-0.1150124	7.7221962	-3.7477096	0.00149676	0.03602087	-1.0848815
POU3F1	-0.1154865	9.00795574	-4.0420599	0.00077938	0.02530701	-0.4685629
CLP1	-0.1172426	7.53894072	-4.0859554	0.00070719	0.0244688	-0.3766135
DEXI	-0.1174009	8.69257547	-3.6700362	0.001778	0.03946587	-1.2471261
LIAS	-0.1191533	10.056471	-3.610018	0.00203085	0.04195989	-1.3722808
TMEM85	-0.1203639	11.6230742	-4.7921497	0.00015006	0.01069161	1.0921825
NFKBIL2	-0.1231155	7.27318972	-3.7379893	0.00152937	0.0363793	-1.1051996
YLPM1	-0.1245177	8.48186849	-3.6295398	0.00194491	0.04056243	-1.3315955
PCDHB20	-0.1247237	7.57096906	-3.5822565	0.0021596	0.04337032	-1.4300966
CSNRP3	-0.1257049	9.54251392	-3.7802832	0.00139246	0.03482135	-1.0167673
CLCN3	-0.1276245	10.1249894	-4.0890771	0.00070232	0.02446071	-0.3700753
FKBP7	-0.1278957	7.41293178	-4.1650797	0.00059363	0.02207153	-0.2109403
ZFR	-0.1284916	7.3675072	-4.2558588	0.00048576	0.01979244	-0.0210489
TCERG1	-0.1285572	7.94280238	-3.7054727	0.00164369	0.03757425	-1.1731402
NUDT18	-0.1295314	10.7081417	-3.7471992	0.00149845	0.03602087	-1.0859483
CHFR	-0.1296175	9.26186758	-3.605056	0.00205329	0.04232096	-1.3826184
ZFP161	-0.1302061	7.62611164	-4.6935265	0.00018595	0.0121131	0.88895208
HIVEP2	-0.130601	7.4323918	-4.1212031	0.00065412	0.02347945	-0.3027967
CADM2	-0.1306986	8.6894753	-4.1717878	0.00058489	0.0219656	-0.1969005
CSDA	-0.1307009	7.88704555	-3.8469579	0.00120106	0.0317664	-0.8772413
DLGAP2	-0.1308983	9.560601	-3.5944502	0.00210208	0.04274226	-1.4047085
PRPF38A	-0.1317356	8.10887284	-3.9903167	0.00087404	0.02660348	-0.5769674
UBE2D3	-0.1333185	9.42536592	-3.9411236	0.00097474	0.02862632	-0.6800285
CPSF2	-0.1342043	7.55813994	-3.6665022	0.00179197	3.95E-02	-1.254501
ABCA5	-0.1344657	7.51106804	-4.0012636	0.0008531	0.02647864	-0.5540325
NAT5	-0.1351429	9.48699766	-3.7197853	0.00159235	0.03701346	-1.143241
H1F0	-0.1351502	8.07874811	-3.5007648	0.00258624	0.04830549	-1.5994908
GLRB	-0.1352244	7.55868085	-3.7232765	0.00158008	0.03689059	-1.1359464
2310047O13	-0.1352341	7.66377073	-3.5298152	0.00242533	0.04687641	-1.5391632
C130074G19	-0.1354887	7.88717846	-3.6460368	0.00187511	0.04013795	-1.2971963
1700023B02I	-0.1365221	8.22573752	-3.5122443	0.00252143	4.77E-02	-1.5756601
ST13	-0.1379178	7.74137869	-3.9246231	0.00101105	0.02923188	-0.7145944
HISPPD1	-0.1381504	7.83575908	-4.8018035	0.00014695	0.01069161	1.11203127
CAPN2	-0.1383722	8.27107947	-4.5664679	0.0002454	1.44E-02	0.62599409
EIF4G2	-0.1387461	7.92252007	-4.4684063	0.00030424	0.01567591	0.42228476
MGLL	-0.1390224	9.94378225	-3.9107853	0.00104255	0.02957543	-0.7435801
PTPRS	-0.1390886	11.4334218	-3.6330777	0.00192972	4.04E-02	-1.3242196
CST3	-0.1390921	14.0045232	-3.5155128	0.00250327	0.04768417	-1.568873
GNAS	-0.1402617	10.0597654	-3.5550888	0.00229344	4.52E-02	-1.4866249
5730494N06	-0.1402758	9.41623351	-3.7148672	0.00160981	3.72E-02	-1.153516
2810410P22I	-0.140333	8.57764437	-3.6656241	0.00179547	0.03948115	-1.2563336

ACOT11	-0.141302	8.36026983	-4.2772759	0.00046334	0.01955849	0.02371306
GLRX	-0.1414744	10.9404864	-3.7242947	0.00157651	3.69E-02	-1.1338188
SLC40A1	-0.1416018	8.85927315	-3.5915524	0.00211561	4.28E-02	-1.4107428
6430548M08	-0.1427289	10.9664556	-3.6144432	0.00201104	0.0417766	-1.3630604
2410015M20	-0.1433144	8.7773327	-4.035274	0.00079118	0.02561145	-0.4827791
UHRF1BP1L	-0.1434568	9.53967134	-3.9353293	0.00098734	0.02878278	-0.6921668
HSPA4L	-0.1435737	7.7108802	-4.0442053	0.00077568	0.02526478	-0.4640685
ASH1L	-0.1442621	7.909971	-3.8475401	0.00119952	0.0317664	-0.8760225
TMEM218	-0.1442813	8.53966465	-4.1916354	0.00055979	0.02165034	-0.1553668
DERL2	-0.1456675	7.45903064	-4.0326078	0.00079587	0.02568437	-0.4883647
TUBA3B	-0.1465997	7.70010774	-3.8659023	0.00115165	0.03081715	-0.8375774
LOC1000457	-0.1466427	7.93095203	-3.6606905	0.0018152	3.97E-02	-1.2666279
THY1	-0.1467277	13.5455885	-4.4102668	0.00034569	0.01634416	0.30123607
FUT8	-0.1475569	8.65968238	-5.0356684	8.87E-05	0.00874856	1.59018114
LOC216963	-0.1488169	8.02440682	-4.0075824	0.00084124	0.02626501	-0.5407941
RG9MTD1	-0.1494071	8.48755627	-4.9585734	0.0001047	9.53E-03	1.43315063
LOC545013	-0.1494416	7.66484905	-4.9652888	0.0001032	0.00947015	1.44685299
FIP1L1	-0.1495388	7.72388406	-5.282379	5.24E-05	0.00650479	2.08834228
RRN3	-0.1501706	8.70795332	-3.9625496	0.00092952	0.02778825	-0.6351416
LOC1000446	-0.1507001	12.0216155	-3.6352927	0.00192028	0.04036789	-1.3196015
ABHD12	-0.1508616	9.44510909	-3.8053452	0.00131718	0.03357534	-0.9643363
APH1B	-0.151971	9.266371	-3.8391171	0.00122213	0.0317664	-0.8936553
ALDH6A1	-0.1528466	9.7845189	-3.4793173	0.00271178	0.04959697	-1.6439844
GRSF1	-0.1544344	10.1115142	-4.2646659	0.00047641	0.01979244	-0.00264
LRRC4C	-0.1545145	7.88670858	-3.8758845	0.00112644	0.03055863	-0.8166748
WDR23	-0.1550338	7.93818618	-3.7401735	0.00152198	0.03633807	-1.1006343
LMO2	-0.1557949	9.3821128	-4.1454766	0.00061993	0.02263695	-0.2519747
WDR17	-0.1558571	8.68696574	-3.8681837	0.00114584	0.03081715	-0.8328004
ZFP451	-0.1564559	8.40970226	-4.0517777	0.00076278	0.02525567	-0.4482051
2610044O15	-0.1569155	8.10324211	-3.6923398	0.00169224	0.03832237	-1.2005669
GRIA4	-0.1574046	7.42719461	-3.918042	0.00102591	0.02934	-0.72838
PCDHA6	-0.1575513	10.6803259	-3.6015035	0.0020695	0.04247652	-1.3900185
AMIGO1	-0.1575703	8.66731713	-4.7712192	0.00015704	0.01069161	1.04912051
STAM2	-0.1596207	7.86622367	-4.3095549	0.0004315	0.01879399	0.09114336
LOC433801	-0.1604121	7.84272998	-4.0499773	0.00076583	0.02525567	-0.4519768
ALDOC	-0.1604724	13.2028104	-3.4884208	0.00265778	0.0490341	-1.6251036
KCNA2	-0.1605114	9.17664985	-3.6384953	0.0019067	0.04032348	-1.3129237
CDV3	-0.1607756	10.7200533	-3.6808284	0.00173597	3.91E-02	-1.2246003
ROCK2	-0.1610946	8.32769383	-3.566618	0.00223567	0.04443124	-1.4626423
SGTB	-0.1612221	10.2994308	-4.2722212	0.00046854	0.01962085	0.01315011
EG622339	-0.1627042	13.5035662	-3.4818024	0.00269693	4.94E-02	-1.638831
TGFB2	-0.1630293	7.60003332	-4.1882669	0.00056397	0.02165034	-0.1624151
IL6ST	-0.1630837	7.65963169	-4.4734564	0.00030089	0.01567591	0.43279025

D0H4S114	-0.1634354	11.5110928	-3.6421041	0.00189152	0.04024431	-1.3053981
PHTF2	-0.1650936	8.13225559	-4.4665934	0.00030546	0.01567591	0.41851307
ABCD2	-0.1652055	8.00728519	-3.8363388	0.00122969	0.03187095	-0.8994711
FHOD3	-0.1652565	8.22154908	-3.5265346	0.00244299	4.70E-02	-1.5459793
SEMA3A	-0.1663517	9.01915107	-3.4771938	0.00272454	4.97E-02	-1.6483875
CETN2	-0.1669167	8.11308925	-3.8706023	0.00113971	0.03076053	-0.8277359
PDZD8	-0.1676796	7.98245209	-3.8171841	0.00128305	0.03302437	-0.9395622
UBE3A	-0.167905	7.89953635	-5.5889646	2.75E-05	0.00453347	2.6971881
UBE2Q2	-0.1683549	8.63428642	-3.5122574	0.00252136	4.77E-02	-1.575633
GPR177	-0.1689461	8.29973345	-3.729273	0.00155921	0.03656519	-1.1234161
NFIC	-0.1699004	8.22564978	-5.0798825	8.07E-05	0.00851403	1.67995744
9130422G05	-0.1703669	8.95589999	-5.0087298	9.40E-05	0.00902024	1.53538103
TCFE3	-0.1703949	7.80417078	-3.9933466	0.0008682	0.02660348	-0.5706194
ASB8	-0.1707015	10.7008508	-3.7563714	0.00146828	0.03590904	-1.0667726
TRAK1	-0.1708425	9.6253053	-3.564189	0.00224772	4.45E-02	-1.4676958
MYO6	-0.1718104	10.0087718	-3.9492823	0.00095726	0.0283173	-0.6629365
AGXT2L1	-0.1727755	7.87128236	-4.5129896	0.0002759	0.01500795	0.5149758
ZNRF1	-0.1731732	8.79042262	-3.7345886	0.00154094	3.64E-02	-1.1123072
C1GALT1C1	-0.1744278	9.03711403	-3.89556	0.00107835	0.03010535	-0.7754696
STMN3	-0.1750526	11.1287635	-4.2618669	0.00047936	0.01979244	-0.0084901
CYR61	-0.1757619	7.22230875	-4.7760849	0.00015539	0.01069161	1.05913445
SLC30A4	-0.1758508	7.70362856	-4.8975028	0.00011945	9.81E-03	1.30833457
ARHGAP20	-0.1760157	10.0221737	-4.1061779	0.00067624	0.02390011	-0.3342605
MAPRE3	-0.176068	8.08865264	-4.9494895	0.00010677	0.00954633	1.41460823
ATP2B2	-0.1760819	9.75118756	-4.6205961	0.00021801	0.01353358	0.73816249
SH3BGRL	-0.1765134	8.91192655	-3.5190688	0.00248366	0.04756826	-1.5614878
EMB	-0.1777382	9.94787478	-3.5694627	0.00222163	4.42E-02	-1.4567234
ENPP2	-0.177869	11.0111666	-4.1829896	0.00057058	0.02173783	-0.1734582
TIMP4	-0.1780647	8.30503367	-5.6290631	2.53E-05	4.53E-03	2.7759177
SLC39A12	-0.1790096	9.77355053	-6.0114878	1.15E-05	0.00303926	3.5155767
SMARCC2	-0.180558	9.05350431	-4.0760214	0.00072292	0.02470159	-0.3974209
TSPYL4	-0.1806403	7.58542523	-4.4382088	0.0003251	0.0159572	0.35943587
LOC329575	-0.182228	9.20120536	-4.1782841	0.00057655	0.02188608	-0.1833052
1110008P14I	-0.1823471	12.1151011	-4.7275242	0.00017268	0.0113203	0.95910201
AADACL1	-0.1826915	11.8767463	-3.880596	0.00111473	0.0303973	-0.8068083
GPC5	-0.1839766	8.91443967	-5.5092457	3.25E-05	0.00482368	2.5400329
KLHL7	-0.1839865	10.1140051	-6.2993531	6.45E-06	0.00259952	4.05834925
ANK3	-0.1845228	8.75934654	-3.7118843	0.00162049	0.03733852	-1.1597473
ZMAT3	-0.1856144	10.5869218	-4.0258403	0.00080789	0.0258163	-0.5025427
NR1D2	-0.1864507	8.71471308	-3.5037399	0.00256929	0.04822693	-1.5933158
ZEB2	-0.1874349	9.56213709	-4.3262908	0.00041588	0.01851802	0.1260879
OSBPL6	-0.1876967	9.33250385	-5.879496	1.51E-05	0.00345963	3.2626273
SORL1	-0.1883491	11.3785844	-3.5974313	0.00208825	4.26E-02	-1.3985001

HSP105	-0.1890076	11.5101573	-4.9278588	0.00011187	0.00954633	1.37042202
ABAT	-0.1893487	10.5606014	-3.9928117	0.00086923	0.02660348	-0.5717401
MDH1	-0.1904976	8.88696055	-3.4972817	0.00260623	4.85E-02	-1.6067193
PTEN	-0.1906967	8.29604428	-3.9070208	0.00105129	0.02973106	-0.7514652
SKIV2L	-0.1915418	9.10145632	-5.6181754	2.59E-05	0.00453347	2.75456207
LUZP2	-0.19157	10.7919992	-3.9440169	0.0009685	0.02854921	-0.6739673
CRIPT	-0.1919416	8.34696681	-7.0878948	1.40E-06	0.00127448	5.48009359
PMP22	-0.1925593	8.6723516	-4.3140628	0.00042724	0.01879399	0.10055698
FAM84A	-0.1928733	7.91803284	-4.9123111	0.00011569	0.0096586	1.33863341
LRRTM3	-0.1937815	8.17919309	-6.2576691	7.01E-06	2.60E-03	3.98052184
NRN1	-0.1941367	12.5845376	-4.247495	0.00049481	0.02000672	-0.0385335
CBLN2	-0.1952227	8.07849599	-5.4237716	3.89E-05	0.00525794	2.37061362
GPR37L1	-0.1955676	9.99328467	-5.6922158	2.22E-05	4.38E-03	2.89947171
OPN3	-0.1959794	7.70812162	-4.8582963	0.00013002	1.04E-02	1.22801484
KCTD3	-0.1960987	9.9574261	-3.8447048	0.00120708	0.0317664	-0.8819581
NOL4	-0.1960989	9.80834094	-3.9802748	0.00089371	0.0268174	-0.5980062
HR	-0.1971504	8.82807208	-5.2857638	5.20E-05	0.00650479	2.09512812
MANSC1	-0.1971735	9.07762709	-4.0812297	0.00071463	0.02464532	-0.3865116
NFU1	-0.1971866	8.01526895	-3.7541902	0.0014754	0.03590904	-1.0713331
ACSBG1	-0.1973576	8.4924128	-6.1708591	8.35E-06	2.67E-03	3.81760026
TMEM128	-0.1979096	9.91595865	-3.7774696	0.00140117	0.03495645	-1.0226522
ARCN1	-0.1990303	7.92460342	-5.5456297	3.01E-05	4.74E-03	2.61186295
PIK3R4	-0.1992865	8.10399151	-4.0743332	0.00072562	0.02470159	-0.400957
RAD23B	-0.2002297	10.355068	-3.5072082	0.00254966	0.04804746	-1.586116
SNX3	-0.200539	8.93641844	-3.9123697	0.0010389	0.02955115	-0.7402613
FGF1	-0.2017156	9.16304371	-4.8390269	0.00013556	0.01047438	1.18848617
PKIG	-0.2048577	8.56107276	-3.9798748	0.00089451	0.0268174	-0.5988443
UCHL5	-0.2052889	9.55696917	-4.5953993	0.00023036	0.01389107	0.68597366
RGS4	-0.2056235	12.4503255	-4.9279685	0.00011185	9.55E-03	1.37064609
CCK	-0.2060288	12.1736661	-3.98368	0.00088699	0.0268174	-0.590872
PAFAH1B1	-0.2069343	9.10718414	-5.217294	6.02E-05	0.00721472	1.9575959
LIN7C	-0.2075546	8.14118018	-3.7532667	0.00147843	0.03590904	-1.0732638
CASC4	-0.2083389	8.50957512	-4.7499668	0.00016446	0.01109412	1.00535749
ABHD3	-0.2092438	8.45255252	-4.4239935	0.00033542	0.01616282	0.32983238
SYNGR1	-0.2092784	9.28063683	-5.0127026	9.32E-05	0.00902024	1.5434676
PHF14	-0.2096679	7.97948142	-4.5593334	0.00024926	0.01437426	0.61119423
CAMKK2	-0.2109883	9.58144867	-4.5673969	0.0002449	0.01437426	0.62792083
MRPL20	-0.2112007	10.2726085	-3.7583026	0.00146201	0.03588045	-1.0627347
PRKAR1B	-0.2117456	10.7502053	-4.4483009	0.00031797	0.0158282	0.38044601
TJP1	-0.2117841	8.39715144	-3.5033697	0.00257139	4.82E-02	-1.5940842
TOP2B	-0.2120287	7.58370864	-5.6673521	2.34E-05	0.00448154	2.85089227
TMEM32	-0.2141034	8.91632432	-3.8878006	0.00109706	0.0302378	-0.7917203
PTN	-0.2154757	11.8384157	-5.4720654	3.51E-05	0.00489116	2.46645274

CYB5	-0.2162946	9.14013625	-4.0110535	0.00083479	0.02626261	-0.5335219
LOC1000441	-0.2163858	8.50961321	-4.3091162	0.00043192	0.01879399	0.09022731
4931406C07I	-0.217141	8.32967179	-5.6873232	2.24E-05	4.38E-03	2.88991893
NECAB1	-0.2182387	8.96109015	-4.4490534	0.00031745	0.0158282	0.38201224
ITGB5	-0.2187978	8.32544989	-5.4282311	3.85E-05	5.26E-03	2.37947584
ALDOA	-0.2196041	12.5394842	-4.4852829	0.00029318	0.01546967	0.45738625
PPP1R3C	-0.2199865	10.5177547	-4.9724692	0.00010162	0.00940662	1.46149919
PPP2R2C	-0.2200551	10.2792603	-4.020888	0.0008168	0.02596293	-0.5129179
VCL	-0.2213409	9.080144	-3.6582041	0.00182523	0.03979618	-1.2718156
SEC11C	-0.2228565	9.80396357	-4.0105957	0.00083564	0.02626261	-0.534481
RWDD4A	-0.2230987	8.63784731	-4.5624506	0.00024757	0.01437426	0.61766087
KIRREL3	-0.2237499	8.25142211	-4.8073395	0.0001452	0.0106406	1.1234098
SEPP1	-0.22404	8.72668434	-5.1405969	7.09E-05	0.00803443	1.80289145
DUSP6	-0.22407	10.1133136	-3.9149815	0.0010329	0.02945992	-0.7347906
LOC1000467	-0.2245433	8.17221881	-5.3862149	4.21E-05	5.55E-03	2.29587811
NAP1L3	-0.224871	9.23351286	-3.8854541	0.00110279	0.03024524	-0.7966346
PPP2R2B	-0.2257091	9.40838835	-3.866222	0.00115084	0.03081715	-0.836908
ATAD1	-0.2257358	9.88655588	-5.1092714	7.58E-05	0.00824206	1.73951447
PI4KA	-0.2257782	10.1724236	-4.4657791	0.000306	0.01567591	0.41681884
PTPRA	-0.2262692	8.98679885	-4.238765	0.00050444	0.02016841	-0.0567864
MXI1	-0.2267802	8.45878813	-3.6534073	0.00184473	0.03999989	-1.2818225
SELK	-0.227769	10.2966912	-5.0428531	8.73E-05	0.00874856	1.6047837
OSBP2	-0.2300977	9.0516124	-3.6337937	0.00192666	0.04037166	-1.322727
ARNT2	-0.2309333	8.71759867	-4.1344409	0.00063524	0.02287963	-0.275079
SLC24A3	-0.23207	11.6875339	-4.2013866	0.00054785	0.02149254	-0.1349651
RFWD2	-0.2321419	8.86329785	-4.5401206	0.00025997	0.01478734	0.57132196
PRMT8	-0.2323778	9.22871897	-5.1684126	6.68E-05	0.00765854	1.85907552
HSF2	-0.2325131	7.9405795	-4.1653342	0.00059329	0.02207153	-0.2104077
NDUFB2	-0.2344496	8.82695992	-3.922792	0.00101517	0.02925189	-0.71843
TBC1D19	-0.2345255	8.62820636	-4.9787711	0.00010025	0.00936197	1.47434911
MDH2	-0.2351998	10.058944	-4.4769589	0.00029858	0.01567591	0.44007544
SEL1L	-0.2355171	8.58912388	-3.8631922	0.00115859	0.03087535	-0.843252
GAD1	-0.2358886	12.3212895	-5.6029221	2.67E-05	0.00453347	2.7246168
DLD	-0.2362858	9.03552759	-3.8651833	0.00115349	0.03081715	-0.839083
CDH22	-0.2362956	7.89125551	-4.2318	0.00051226	2.03E-02	-0.0713507
CLSTN1	-0.2371662	10.0844727	-6.0928238	9.77E-06	2.92E-03	3.67018526
CACYBP	-0.2380646	8.2375256	-5.1303269	7.24E-05	0.00804507	1.78212549
PPM1L	-0.2381145	10.4327393	-5.0367073	8.85E-05	0.00874856	1.59229293
PTGES3	-0.2381442	9.39721669	-6.5800424	3.71E-06	2.17E-03	4.57551004
COX7B	-0.2384971	9.23467748	-4.0248064	0.00080974	0.0258163	-0.5047088
RASSF3	-0.2386374	8.62985941	-4.5716562	0.00024263	1.44E-02	0.63675458
LOC1000468	-0.239122	8.8553679	-4.238672	0.00050454	0.02016841	-0.0569808
ZFML	-0.2397068	9.66136903	-3.9190192	0.00102369	0.02934	-0.7263329

PMPCB	-0.2397606	8.51325408	-5.0650934	8.33E-05	0.00870122	1.64995148
IAP	-0.2417125	11.1988469	-3.8450705	0.0012061	0.0317664	-0.8811926
GSTM5	-0.2417395	11.4669217	-4.4273463	0.00033295	1.61E-02	0.33681563
TYKI	-0.244149	8.79212827	-6.6413004	3.29E-06	2.04E-03	4.6867605
CLCN4-2	-0.2456994	8.23215412	-5.4705299	3.52E-05	0.00489116	2.46341004
EVL	-0.2461018	10.0572227	-6.0204068	1.13E-05	3.04E-03	3.5325778
NDRG2	-0.2463635	9.48164458	-5.4998138	3.31E-05	4.82E-03	2.52138396
FGF12	-0.2468643	9.61837415	-5.928322	1.36E-05	3.33E-03	3.35649054
LOC1000477	-0.247057	9.24341442	-4.2256189	0.0005193	0.02052494	-0.0842772
TOMM70A	-0.2508782	9.46477595	-4.3917911	0.00036003	0.01666395	0.26273105
LOC1000417	-0.2515943	7.83273804	-4.4983327	0.00028491	0.0154186	0.48451675
IDH2	-0.2517951	9.18773007	-4.0574508	0.00075326	0.02525567	-0.4363208
RGS10	-0.2521589	9.90840422	-6.4029468	5.25E-06	2.60E-03	4.2506238
FAM134A	-0.2556575	10.5122235	-4.1055569	0.00067717	0.02390011	-0.3355611
GJB6	-0.2560492	10.7561347	-3.8064935	0.00131383	0.03357103	-0.9619334
LOC1000444	-0.2562376	11.0557537	-4.8443139	0.00013402	0.01047438	1.19933507
NLGN1	-0.25734	8.14182766	-4.8075099	0.00014514	1.06E-02	1.12375995
SNAP91	-0.2586765	8.62884003	-3.8420524	0.0012142	0.0317664	-0.8875108
FOXG1	-0.2590973	10.7068772	-3.4981954	0.00260097	0.04848053	-1.6048232
A530082C11	-0.2602531	8.56951605	-5.6542509	2.40E-05	4.52E-03	2.82526088
ALDH1A1	-0.2606003	7.92979	-5.789918	1.81E-05	3.90E-03	3.08953921
RELL2	-0.261073	10.5292788	-4.8342377	0.00013697	0.01047438	1.17865626
GPM6A	-0.2624029	10.3294071	-5.820415	1.70E-05	3.74E-03	3.14859485
PAIP2	-0.2631944	10.3312977	-3.5511907	0.00231331	0.04544954	-1.4947313
ADAM9	-0.263297	8.77264722	-6.4002743	5.28E-06	2.60E-03	4.24568411
KCNK2	-0.2666458	8.96448927	-4.98485	9.89E-05	9.36E-03	1.48674055
SMPD4	-0.2668337	8.12718492	-3.761587	0.0014514	0.03570311	-1.0558673
PIK3CA	-0.2692312	7.84708796	-5.038058	8.83E-05	8.75E-03	1.59503844
P2RY12	-0.2692981	8.52596033	-6.0705098	1.02E-05	2.92E-03	3.62786627
DYNLT3	-0.2697386	11.6152294	-6.0743402	1.01E-05	0.00291503	3.63513596
NPY	-0.2701757	11.0205184	-7.1147226	1.33E-06	1.27E-03	5.52675332
LOC668837	-0.2715451	9.74082912	-4.1023243	0.00068203	0.02392393	-0.3423308
GPM6B	-0.2843532	8.30149019	-5.2486402	5.63E-05	6.91E-03	2.02062838
CCNG2	-0.2849152	9.25756986	-5.8930387	1.47E-05	0.00343977	3.28869608
STMN1	-0.2858392	10.8422583	-3.8929183	0.00108468	0.03014745	-0.7810023
ABCC10	-0.2858719	7.64549569	-4.2402412	0.0005028	0.02016841	-0.0536998
S100B	-0.2866502	7.76645434	-6.754684	2.64E-06	1.84E-03	4.89113481
LOC1000484	-0.2879513	11.0847746	-3.8452786	0.00120555	0.0317664	-0.8807568
6330577E15I	-0.2880153	8.56399213	-4.6824194	0.0001905	0.01225849	0.86601382
FAM107A	-0.2886275	11.5640312	-4.0447713	0.00077471	0.02526478	-0.4628826
CYFIP2	-0.2933438	10.3946373	-3.7810543	0.00139008	0.03482135	-1.0151543
GLRX3	-0.293824	9.16640062	-4.951497	0.00010631	0.00954633	1.41870675
KPNB1	-0.295192	10.2243301	-5.1783618	6.54E-05	0.00758014	1.87915031

SLC25A4	-0.2976257	7.74603557	-3.720055	0.0015914	0.03701346	-1.1426775
UBE2J1	-0.3014377	8.76611148	-6.3368741	5.99E-06	0.00259952	4.12817879
GLRX2	-0.301634	8.89396468	-5.6025249	2.67E-05	4.53E-03	2.72383663
PLA2G7	-0.3025829	8.28695336	-4.2180155	0.00052809	0.02079464	-0.1001798
HSD11B1	-0.3056531	9.32102244	-5.7480954	1.98E-05	0.00417076	3.00834058
SEPT7	-0.306195	8.20691273	-5.6269663	2.54E-05	0.00453347	2.77180628
RORA	-0.3063278	8.98348808	-6.1755063	8.27E-06	2.67E-03	3.82635052
SLC7A10	-0.3170964	8.63636723	-9.2966724	2.98E-08	3.14E-04	8.95052775
TRIM37	-0.3224632	11.0173666	-4.2695826	0.00047127	0.0196574	0.00763596
SLC12A5	-0.3303709	8.21189792	-5.4918668	3.37E-05	4.82E-03	2.50566211
MAP1LC3B	-0.3332635	9.9737923	-3.7873352	0.00137085	0.03461981	-1.002016
DDX3X	-0.3413857	9.46155714	-3.7052833	0.00164438	0.03757425	-1.1735358
SCN1A	-0.3450592	10.2520814	-5.9635112	1.27E-05	0.00326922	3.42392571
A030009H04	-0.3517367	9.0145	-4.6531849	0.00020304	1.28E-02	0.80559285
FOS	-0.3549308	9.76647721	-6.2361678	7.32E-06	0.00259952	3.94027465
ID2	-0.3554898	9.26615316	-4.4661966	0.00030572	0.01567591	0.41768756
GLUL	-0.3688954	8.42803552	-5.5678209	2.87E-05	0.00466505	2.65558787
SETD3	-0.3691924	9.38776346	-3.8397178	0.00122051	0.0317664	-0.8923978
NDRG4	-0.3746447	12.0568899	-4.6209895	0.00021783	0.01353358	0.73897696
LOC1000485	-0.3805732	8.46021607	-7.8761877	3.29E-07	6.59E-04	6.80399782
PDZRN3	-0.3813116	9.21724556	-3.7361858	0.00153549	0.0363793	-1.108969
LPGAT1	-0.3814508	8.4351187	-4.6712827	0.00019519	0.0124836	0.84300466
ATP5A1	-0.3816518	10.8903379	-3.9059999	0.00105367	0.02973106	-0.7536034
NDUFB5	-0.387066	10.2090556	-3.9492794	0.00095727	0.0283173	-0.6629427
JAK1	-0.3873114	8.62220612	-5.9200405	1.39E-05	3.33E-03	3.3405942
PTGDS	-0.3907285	9.92990529	-5.2980302	5.07E-05	6.50E-03	2.11970822
MAP2K1	-0.4182157	9.56538826	-6.9581913	1.79E-06	0.00145025	5.25291244
SPARCL1	-0.419559	10.7264921	-4.9297217	0.00011142	0.00954633	1.37422912
HUWE1	-0.4261975	9.57806109	-6.3016232	6.42E-06	2.60E-03	4.06258023
ARC	-0.4269303	10.6445515	-5.9424939	1.33E-05	3.33E-03	3.38367059
DUSP1	-0.4665203	10.5911814	-7.411806	7.64E-07	0.0010072	6.03587649
NPAS4	-0.4786863	8.18395513	-7.4810389	6.72E-07	1.01E-03	6.15253048
NEFM	-0.4893661	10.0428796	-4.1546125	0.00060753	0.02233879	-0.23285

DEGs in susceptible mPFC

adj.P.Value	< 0.05
DEGup	246
DEGdown	262

Gene	logFC	AveExpr	t	P.Value	adj.P.Val	B
HBA-A1	0.96897	12.60395	12.27137	7.12E-10	7.51E-06	1.21E+01
HBB-B1	0.521643	8.799639	5.777767	2.23E-05	4.67E-03	2.9152003
SLA	0.375319	8.890923	3.710512	0.001737	0.0422373	-1.2241965
SGK1	0.353696	11.44792	9.320597	4.29E-08	1.51E-04	8.66E+00
FDPS	0.343828	10.83188	6.688559	3.81E-06	1.99E-03	4.5755552
ALAS2	0.340133	7.583163	6.373514	6.92E-06	0.002353	4.0155208
STK32C	0.335002	9.137387	7.370428	1.09E-06	0.0009948	5.7345498
FUS	0.331593	8.26822	6.830087	2.92E-06	1.71E-03	4.8221066
PFDN2	0.330036	11.04169	4.745774	0.000187	1.31E-02	0.894604
CCDC85B	0.325464	10.26655	5.333016	5.49E-05	7.33E-03	2.0605731
GABARAPL1	0.312178	11.87212	5.840394	1.97E-05	0.0044123	3.0333366
AHI1	0.305951	10.57708	7.449293	9.49E-07	9.95E-04	5.863904
STMN4	0.287774	9.660887	9.03581	6.68E-08	1.61E-04	8.2656348
DLG4	0.284037	9.496925	6.219446	9.32E-06	2.46E-03	3.7360727
COL6A1	0.277368	9.225453	3.661475	0.0019322	0.0440791	-1.3249574
SUMO3	0.270987	9.241103	5.450956	4.31E-05	6.32E-03	2.2897973
NME2	0.267915	10.32974	6.338699	7.40E-06	2.35E-03	3.9526914
DYNLL2	0.263819	8.9233	7.685176	6.27E-07	0.0008018	6.2450346
RASL10A	0.259366	9.588202	6.284711	8.22E-06	2.35E-03	3.854893
KLF16	0.25796	8.781993	4.963057	0.0001183	1.08E-02	1.3304385
CRYM	0.255724	11.54698	4.268398	0.0005188	0.0223847	-0.0768859
FEZF2	0.254711	9.724721	5.430714	4.49E-05	0.0063204	2.2505848
VPS25	0.254683	8.981466	8.217767	2.52E-07	3.80E-04	7.0741714
RAMP3	0.253779	8.392446	5.510355	3.82E-05	6.20E-03	2.4045524
NTSR1	0.250128	8.087294	3.846251	0.0012936	3.63E-02	-0.944939
ACTR1A	0.244134	10.82985	4.112645	0.0007262	2.70E-02	-0.3966898
SLC38A5	0.2425	8.130117	6.477345	5.68E-06	2.22E-03	4.2017943
CDH13	0.242017	8.439697	7.635214	6.84E-07	8.02E-04	6.1650265
RPS26	0.240258	11.85228	5.002186	0.000109	0.0105524	1.4084067
ARHGEF15	0.23718	8.08675	7.029222	2.02E-06	1.42E-03	5.1636991
SLC35E3	0.235561	9.527682	4.523534	0.0003001	1.62E-02	0.4443605
COL5A1	0.235437	8.052943	5.883601	1.80E-05	4.14E-03	3.1145111
HRMT1L2	0.235143	10.48449	6.237518	9.00E-06	2.44E-03	3.7690386
P140	0.234813	8.305353	6.500707	5.43E-06	2.22E-03	4.2434774
TCEAL3	0.233308	8.924958	7.307184	1.22E-06	9.95E-04	5.6301154
LY6A	0.231051	9.565472	5.311914	5.73E-05	7.47E-03	2.0193709
RPS4Y2	0.230432	7.95517	5.675745	2.73E-05	5.32E-03	2.7215548

STXBP2	0.225695	9.009519	3.794967	0.001446	0.0382441	-1.05049
PRR7	0.224276	9.384036	6.415535	6.39E-06	2.25E-03	4.0911074
PTPRU	0.222252	8.320852	3.8093	0.0014017	0.0379276	-1.0209927
ST3GAL5	0.217225	9.919604	4.919403	0.0001297	0.010859	1.2432604
ANAPC5	0.213799	10.00353	4.373534	0.0004138	1.96E-02	0.138378
DGKG	0.213623	10.63397	5.488186	3.99E-05	6.30E-03	2.3617781
BLMH	0.213411	8.428104	4.728392	0.000194	1.31E-02	0.8595418
COTL1	0.211301	9.645081	4.283494	0.0005022	0.022131	-0.045943
TSC22D1	0.210839	9.097877	4.639701	0.0002342	0.0142703	0.680217
SNRPD3	0.210506	10.62739	3.800386	0.0014291	0.038148	-1.0393382
HNRNPK	0.210168	8.520658	5.149467	8.02E-05	8.81E-03	1.7003466
GABBR1	0.210083	8.463038	6.577975	4.69E-06	2.06E-03	4.3807329
LOC100045019	0.209417	11.22315	4.858408	0.0001474	1.18E-02	1.1211183
CCL21A	0.208936	9.212146	5.740611	2.40E-05	4.78E-03	2.8448463
EEF2	0.207607	8.350257	5.463251	4.20E-05	6.30E-03	2.3135897
5430437P03RIK	0.207007	9.352445	4.451291	0.0003502	0.0178553	0.2971767
GPN3	0.202006	8.406506	4.629542	0.0002394	0.014362	0.6596341
EG434858	0.200346	8.69901	4.755006	0.0001834	1.30E-02	0.9132145
YRDC	0.199725	8.128247	5.070903	9.44E-05	9.67E-03	1.5449252
LSM8	0.198161	9.56242	4.828431	0.000157	1.20E-02	1.060952
6430598A04RIK	0.195497	8.393332	5.156959	7.89E-05	0.008814	1.7151298
DBR1	0.195252	8.708354	4.307957	0.0004765	0.0213636	0.0041774
SYT5	0.193934	10.3186	4.675625	0.000217	0.0137081	0.7529338
WDR61	0.193291	9.834414	4.383418	0.0004051	1.93E-02	0.1585836
LOC100043257	0.192503	9.951471	4.839015	0.0001536	0.0118285	1.0822052
SFRS5	0.191888	10.07632	4.939203	0.0001244	0.0107935	1.2828262
ARL6IP1	0.190973	12.59459	3.923816	0.0010932	0.0331516	-0.7852561
STARD3NL	0.190548	8.993388	5.104881	8.80E-05	9.27E-03	1.6122309
PPARGC1B	0.190331	9.531282	4.432922	0.0003643	0.0181667	0.2596963
GPS1	0.18938	10.67956	3.784986	0.0014777	0.0389665	-1.0710273
SLC8A2	0.188883	8.434484	4.462843	0.0003417	0.0175889	0.3207354
TATDN2	0.18849	8.410132	5.089178	9.09E-05	0.0094037	1.5811418
EIF3D	0.187682	10.47309	4.510055	0.0003088	0.0164608	0.4169253
MAGI2	0.186419	7.583132	5.055147	9.76E-05	9.81E-03	1.5136706
D430041B17RIK	0.185478	9.123235	5.113147	8.65E-05	0.0092572	1.6285846
KCNQ2	0.185358	7.858577	5.93669	1.62E-05	0.0038929	3.2138782
EXTL1	0.185254	8.809089	4.969555	0.0001167	0.0107092	1.343398
IVNS1ABP	0.184364	8.896972	4.820059	0.0001598	0.0121341	1.0441338
VTI1B	0.184284	12.22503	4.685603	0.0002125	0.0135885	0.7731115
KCTD10	0.184139	9.086462	4.276568	0.0005098	0.0222307	-0.0601382
ERP29	0.183951	11.32563	3.861814	0.0012507	0.0355744	-0.9129027
DYNC1H1	0.182527	10.29217	3.588179	0.0022655	0.0480161	-1.4753765
MED6	0.181982	8.938034	5.800617	2.13E-05	4.67E-03	2.9583697

COG1	0.181903	8.320809	5.433429	4.47E-05	0.0063204	2.2558479
MRPL10	0.181203	10.16936	5.111063	8.68E-05	0.0092572	1.6244625
NME5	0.181176	9.103259	3.884113	0.0011916	0.0346408	-0.8669952
CXX1C	0.180428	10.42897	4.386679	0.0004023	1.92E-02	0.1652488
LOC100046136	0.180234	8.011977	4.579075	0.0002665	0.0152033	0.557257
DUS4L	0.179894	8.419626	6.434088	6.17E-06	2.24E-03	4.1243929
EG633640	0.178736	7.723142	5.483442	4.03E-05	6.30E-03	2.3526179
CSTB	0.177749	11.04008	3.966763	0.000996	3.19E-02	-0.6968424
0610006I08RIK	0.177621	10.29162	4.57205	0.0002705	1.53E-02	0.5429912
DYNC1I1	0.17761	9.345363	4.98926	0.000112	0.0105759	1.3826696
EGFL7	0.177327	8.768657	5.326308	5.56E-05	7.34E-03	2.0474829
CLPP	0.177278	8.906726	4.306705	0.0004778	0.0213636	0.0016131
LMO3	0.177237	8.373424	3.69369	0.0018017	0.0428221	-1.2587713
ABHD14B	0.177129	9.130865	3.918679	0.0011055	3.32E-02	-0.7958329
KLF5	0.176321	8.790538	3.798994	0.0014334	0.038148	-1.0422022
NRBP2	0.175352	10.27506	4.091024	0.000761	2.77E-02	-0.441149
ATP2B1	0.174848	9.343558	4.048046	0.0008352	0.0288505	-0.5295543
EXOC4	0.174501	8.785003	5.593306	3.23E-05	5.77E-03	2.5640136
DCTN1	0.174233	9.172176	4.481226	0.0003285	1.72E-02	0.3582078
EXOSC7	0.174096	10.0605	4.988152	0.0001122	0.0105759	1.3804623
RAB3GAP2	0.171332	9.081676	3.718223	0.0017082	0.0420782	-1.2083428
2610208M17RIK	0.170598	9.261427	3.723584	0.0016884	4.19E-02	-1.197321
TMEM132D	0.170478	8.122685	4.192687	0.0006109	0.0242902	-0.2322284
ORC6L	0.170426	9.613107	4.755003	0.0001834	1.30E-02	0.9132086
RASAL1	0.170138	8.861191	3.624383	0.0020943	4.59E-02	-1.4011091
LOR	0.170118	9.268759	4.237152	0.000555	0.0232414	-0.1409663
PSME2	0.170043	7.759331	4.423677	0.0003716	1.83E-02	0.2408252
MED30	0.167486	10.05659	4.20822	0.0005907	0.023977	-0.2003383
NRBF2	0.167154	8.808702	4.081057	0.0007776	0.028006	-0.4616478
JTV1	0.16656	8.567839	5.45987	4.23E-05	0.0062998	2.3070494
SLC25A44	0.165965	9.873773	3.74139	0.0016244	0.0411081	-1.1607075
BEX4	0.165344	9.108064	4.208711	0.0005901	0.023977	-0.1993319
TDRD7	0.164357	9.563536	4.926076	0.0001278	1.08E-02	1.2565981
POLD2	0.163387	8.169336	3.905699	0.001137	0.0336113	-0.8225565
BC002163	0.163137	11.47851	3.917963	0.0011072	0.0331738	-0.7973062
SAMD14	0.163113	9.194004	3.915457	0.0011132	0.0331738	-0.8024653
PIGK	0.161865	7.897873	4.979266	0.0001144	0.0105855	1.362757
SUHW4	0.161747	9.016089	3.909671	0.0011273	0.0334165	-0.8143785
ZFP282	0.161625	8.667863	4.415859	0.0003779	0.0184617	0.2248618
TCTEX1D2	0.161564	10.06042	3.633916	0.0020514	0.045575	-1.3815429
2700094K13RIK	0.161077	10.63579	4.169651	0.000642	2.50E-02	-0.2795383
ARL2BP	0.160048	8.993257	5.554768	3.49E-05	0.0060366	2.4900464
G3BP1	0.159936	8.93059	3.748665	0.0015989	0.0408557	-1.145746

MAPK1	0.159414	11.3214	4.558754	0.0002783	0.0153781	0.5159785
B9D1	0.158822	8.634567	3.841866	0.001306	3.64E-02	-0.9539657
GM1821	0.158647	14.51018	4.298726	0.000486	2.16E-02	-0.0147312
DPYSL5	0.157953	7.951066	4.143668	0.0006791	2.60E-02	-0.3329226
LOC100047707	0.157641	10.11424	3.708051	0.0017463	0.0422478	-1.2292545
VWF	0.156394	7.58027	3.928077	0.0010832	3.29E-02	-0.7764846
CLPTM1L	0.155821	10.78891	3.936663	0.0010632	3.29E-02	-0.7588084
XAB1	0.155755	9.11145	4.213384	0.0005842	0.023977	-0.1897391
UQCRC2	0.155417	12.55515	3.597734	0.002219	0.0473959	-1.455784
DDR1	0.155245	8.746017	3.614508	0.0021397	4.64E-02	-1.4213743
TUBB5	0.15446	13.47496	3.91566	0.0011127	3.32E-02	-0.8020471
MMP24	0.154416	9.045771	4.175613	0.0006338	0.0249577	-0.2672918
CHMP4B	0.154031	9.648872	4.308419	0.000476	0.0213636	0.0051225
RAP1GAP	0.153988	7.960334	3.756793	0.0015709	0.0403809	-1.1290281
SNRPD1	0.15382	10.48105	3.62394	0.0020963	0.0458891	-1.4020197
ZFP316	0.153078	7.801568	4.524038	0.0002997	0.0161733	0.4453853
RANBP1	0.152317	10.34559	4.862056	0.0001463	0.0118285	1.1284332
TOMM34	0.152252	9.343561	5.100615	8.88E-05	9.27E-03	1.6037867
D11MOH35	0.152062	7.827309	5.242949	6.61E-05	0.0080139	1.8843282
CASKIN1	0.151806	9.529345	4.641981	0.0002331	0.0142703	0.6848358
HSF1	0.151512	9.898833	4.094285	0.0007556	2.76E-02	-0.4344433
TMEM147	0.150976	11.12406	3.734405	0.0016492	0.0414383	-1.1750706
D1BWG0212E	0.150937	8.263171	4.23078	0.0005627	0.0234699	-0.1540405
DPM1	0.15081	8.642563	4.840004	0.0001532	0.0118285	1.0841913
ORF61	0.150677	9.498599	4.19159	0.0006123	2.43E-02	-0.234481
L7RN6	0.150631	10.17926	4.940003	0.0001242	0.0107935	1.2844237
PSMD4	0.150562	11.68837	4.068033	0.0007998	2.84E-02	-0.488435
ATG16L1	0.149851	10.13511	4.162404	0.0006522	2.52E-02	-0.2944254
2610507B11RIK	0.14958	9.766797	5.062891	9.60E-05	0.0097424	1.529034
SPRN	0.149176	8.000731	4.221884	0.0005736	0.0237992	-0.1722947
EIF2B5	0.148548	9.632521	4.170794	0.0006404	2.50E-02	-0.2771915
EXOSC2	0.147479	8.369204	4.089912	0.0007628	2.77E-02	-0.443436
HAX1	0.147245	8.016897	3.714377	0.0017225	4.21E-02	-1.2162505
ZNRD1	0.147203	9.229558	4.485498	0.0003255	1.72E-02	0.3669132
ILK	0.146557	9.884983	4.171545	0.0006394	0.0250013	-0.2756489
YIF1B	0.14623	9.341596	3.997346	0.0009321	0.03036	-0.6338885
KHDRBS1	0.145987	8.73763	3.716203	0.0017157	4.21E-02	-1.2124975
JTB	0.14583	11.00039	4.22067	0.0005751	0.0237992	-0.1747858
PDE1B	0.145247	10.87632	3.665288	0.0019163	4.40E-02	-1.317125
UHRF2	0.145179	9.131067	4.162732	0.0006517	0.0252097	-0.2937517
ANKRD39	0.14504	8.27174	4.801524	0.0001662	0.0124395	1.0068733
1600014C10RIK	0.14352	8.808375	3.719214	0.0017045	0.0420782	-1.2063058
BC031353	0.14319	9.414805	3.816844	0.0013789	0.0374075	-1.0054679

FAM116B	0.143188	9.604264	3.581605	0.0022981	4.84E-02	-1.4888568
PNKP	0.143153	8.659156	4.946329	0.0001225	0.0107935	1.2970562
GRIA1	0.143123	10.18257	3.821763	0.0013642	0.037219	-0.9953431
SYVN1	0.142927	10.57354	4.069063	0.000798	2.84E-02	-0.4863166
KCNIP4	0.142649	7.685055	4.616144	0.0002463	1.45E-02	0.6324748
HDGFRP2	0.142384	10.8939	4.059298	0.0008151	2.86E-02	-0.5064043
ING4	0.142228	8.670024	3.869869	0.001229	0.0353389	-0.896321
ZCCHC18	0.142209	12.89845	4.248317	0.0005418	2.29E-02	-0.1180636
VAPB	0.142164	9.065473	4.430168	0.0003665	0.0181667	0.2540762
CXX1A	0.142075	8.073588	3.658833	0.0019433	4.41E-02	-1.3303827
UBE2L3	0.141729	11.23016	3.710858	0.0017357	0.0422373	-1.2234837
1110002N22RIK	0.141105	7.999189	4.067294	0.0008011	0.0283686	-0.4899556
SMARCD2	0.139967	8.141246	3.959369	0.0010121	0.0321708	-0.7120641
ZFP672	0.139931	9.711252	3.61077	0.0021571	0.0465516	-1.4290428
NUDT2	0.139819	8.030182	3.598642	0.0022146	0.0473959	-1.4539203
SIAT7F	0.139795	7.657323	4.637583	0.0002353	1.43E-02	0.6759273
LMAN2L	0.139435	9.210995	3.689018	0.00182	0.0430189	-1.2683727
2410004L22RIK	0.139043	7.654476	3.797536	0.0014379	3.81E-02	-1.0452022
5730410E15RIK	0.138958	10.59782	3.621597	0.002107	4.59E-02	-1.4068272
PRDM4	0.138835	9.504693	4.600367	0.0002547	1.46E-02	0.6004748
SSBP2	0.138831	9.144156	4.047276	0.0008366	2.89E-02	-0.5311384
RCL1	0.138223	8.423886	4.015698	0.0008958	0.0298208	-0.5961193
RALY	0.138174	9.379945	3.984811	0.0009578	0.0309099	-0.6596899
ATP5C1	0.137801	12.63172	3.864364	0.0012437	0.0355648	-0.9076535
GDPD1	0.136915	10.0911	3.638206	0.0020324	0.0453434	-1.3727366
SERBP1	0.136809	10.56853	4.047786	0.0008356	0.0288505	-0.5300885
CYB5R3	0.136582	8.697171	4.008239	0.0009104	0.0301166	-0.6114701
SYF2	0.135763	9.399612	4.671434	0.000219	0.0137081	0.7444567
TSTA3	0.135428	9.79832	3.673085	0.0018841	0.0435078	-1.3011087
GSPT1	0.134745	10.82309	3.959454	0.0010119	3.22E-02	-0.7118882
TUBA1B	0.134351	14.65323	3.732142	0.0016573	0.0415436	-1.1797256
MYCBP2	0.134087	9.801209	3.663878	0.0019222	4.40E-02	-1.3200218
9030607L17RIK	0.133891	8.32339	3.59919	0.002212	0.0473959	-1.452796
CDS2	0.133199	7.920417	3.625394	0.0020897	4.59E-02	-1.3990357
CBFA2T3H	0.132913	8.344349	3.818029	0.0013754	0.0374075	-1.0030287
DHRS7B	0.132653	8.680331	4.311909	0.0004724	0.0213636	0.0122713
ACTB	0.132099	12.11403	4.403028	0.0003884	0.018717	0.1986563
0610010K14RIK	0.131356	8.158006	3.870976	0.001226	0.0353389	-0.8940413
MTX1	0.130671	9.247128	4.094821	0.0007548	2.76E-02	-0.4333412
RBBP7	0.130079	8.381073	4.721453	0.0001969	1.32E-02	0.845536
PHF5A	0.129925	10.43582	4.124193	0.0007083	0.0266828	-0.3729514
BRD9	0.129672	10.20812	3.6304	0.0020671	4.57E-02	-1.3887604
EMD	0.129489	8.159979	4.651248	0.0002286	1.42E-02	0.7036036

FOXK1	0.12937	7.461842	3.84439	0.0012989	3.63E-02	-0.9487699
PKIA	0.12924	12.39856	3.702452	0.0017677	4.24E-02	-1.2407629
METTL3	0.12871	9.368955	3.762573	0.0015513	4.02E-02	-1.1171368
SPINK8	0.128279	7.749771	3.704247	0.0017608	0.042397	-1.2370731
FBXL6	0.12826	8.625924	3.885182	0.0011888	3.46E-02	-0.8647952
VPS16	0.128226	9.014455	4.139139	0.0006858	0.0261266	-0.3422303
KCTD5	0.127828	7.933331	4.523046	0.0003004	1.62E-02	0.4433671
THOC4	0.12761	9.268186	4.011957	0.0009031	3.00E-02	-0.6038168
BLVRB	0.127497	8.8192	3.834749	0.0013263	3.67E-02	-0.9686156
PCGF1	0.127042	9.412433	4.025675	0.0008766	2.95E-02	-0.5755864
RASSF1	0.126531	8.30912	3.636178	0.0020413	0.0454474	-1.3768999
PTK2	0.126208	11.19972	4.246995	0.0005433	2.29E-02	-0.1207754
4933403G14RIK	0.126073	7.826789	3.93406	0.0010692	0.0328595	-0.7641661
WRB	0.124858	9.794922	4.042061	0.0008461	2.90E-02	-0.5418695
TMED4	0.122656	9.128541	4.709969	0.0002018	0.0131817	0.822348
UBE2E1	0.122032	8.225489	4.267647	0.0005197	2.24E-02	-0.078427
MBD3L2	0.121001	7.391808	3.680664	0.0018534	0.0431884	-1.2855374
IL18BP	0.119739	7.452616	3.739589	0.0016307	0.0411705	-1.1644107
KAT5	0.117979	8.077403	3.744553	0.0016133	4.10E-02	-1.1542025
NARG1L	0.117764	7.907149	3.949565	0.0010338	0.0322229	-0.7322453
ZFX	0.117032	7.98917	3.844906	0.0012974	3.63E-02	-0.947708
RUVBL1	0.116693	8.727501	4.435915	0.000362	0.0181667	0.2658044
ESAM	0.113503	8.745956	3.77751	0.0015018	0.0392299	-1.0864077
GMDS	0.112856	7.776739	3.949	0.0010351	3.22E-02	-0.73341
LSM14B	0.111337	7.492098	3.56886	0.0023625	0.0493104	-1.5149805
MRPL46	0.110875	9.525797	3.735325	0.0016459	0.0414383	-1.1731792
RPS6KA5	0.110164	7.95178	3.772839	0.0015171	0.0394346	-1.0960176
SLC35B3	0.108427	8.700368	3.717621	0.0017104	0.0420782	-1.2095806
TSC22D2	0.107861	9.549037	3.779019	0.0014969	0.0392299	-1.0833029
SLITRK1	0.107371	7.75003	3.920162	0.0011019	3.32E-02	-0.7927785
LOC100048384	0.10262	8.77681	3.702962	0.0017658	4.24E-02	-1.2397159
LONRF2	0.10077	7.96813	3.630222	0.0020679	4.57E-02	-1.3891268
PPT1	0.100729	8.384394	3.708816	0.0017434	0.0422478	-1.2276814
GPR27	0.09941	7.528056	3.642811	0.0020121	0.0449874	-1.3632827
5133400G04RIK	0.098492	7.860185	3.595705	0.0022288	0.0474203	-1.4599443
ZFP295	0.097602	8.179883	3.797281	0.0014387	0.038148	-1.0457285
CSNK1D	0.090062	8.112241	3.577346	0.0023194	0.0487582	-1.4975874
SNX25	-0.10074	8.230302	-3.66727	0.0019081	4.39E-02	-1.3130609
NDFIP1	-0.11011	12.47241	-3.67371	0.0018815	4.35E-02	-1.2998167
OS9	-0.11161	9.776048	-3.64767	0.001991	0.0447271	-1.3533095
2810470D21RIK	-0.11326	7.521325	-3.58232	0.0022945	0.0484061	-1.487397
POU3F1	-0.11379	9.007956	-3.57177	0.0023476	0.0492539	-1.5090201
PACSIN2	-0.11422	7.673129	-3.93095	0.0010764	3.29E-02	-0.7705723

ZFP161	-0.11827	7.626112	-3.93071	0.001077	3.29E-02	-0.7710695
PTPRD	-0.11857	12.09508	-3.83201	0.0013342	0.0366727	-0.974249
TMEM85	-0.11889	11.62307	-4.24611	0.0005444	0.0228879	-0.1225992
CLP1	-0.11975	7.538941	-3.74515	0.0016112	0.0410235	-1.1529648
HIST1H4H	-0.12053	7.700087	-3.86318	0.0012469	0.0355648	-0.9100871
KIFAP3	-0.12203	11.27705	-3.98625	0.0009548	3.09E-02	-0.6567186
TPRKB	-0.12374	8.811318	-3.68152	0.0018499	0.0431884	-1.2837892
EDNRB	-0.12397	9.175007	-3.61501	0.0021373	0.0463651	-1.4203334
CAPN2	-0.12446	8.271079	-3.80164	0.0014252	3.81E-02	-1.0367638
SNAPC1	-0.12501	7.985758	-3.5881	0.0022659	4.80E-02	-1.4755423
HISPPD1	-0.12528	7.835759	-4.02386	0.0008801	0.0294843	-0.5793265
MTERFD3	-0.12531	7.859384	-3.72912	0.0016682	4.16E-02	-1.1859366
PREP	-0.1262	8.264684	-4.11758	0.0007185	2.68E-02	-0.3865473
TMEM47	-0.12877	7.878606	-3.60567	0.0021811	4.70E-02	-1.4395138
CLCN3	-0.12886	10.12499	-3.7013	0.0017721	0.0424069	-1.2431345
SPINK10	-0.12902	7.611911	-3.89829	0.0011555	0.0338716	-0.8378173
ABHD6	-0.12902	7.722196	-3.88959	0.0011775	3.44E-02	-0.8557137
TMEM209	-0.12931	8.839655	-3.83074	0.0013379	0.0366727	-0.9768611
ATP2B2	-0.13267	9.751188	-3.97116	0.0009866	3.17E-02	-0.6877925
RG9MTD1	-0.1333	8.487556	-4.14392	0.0006787	0.0259659	-0.3324036
NOL14	-0.13453	8.148153	-3.91433	0.001116	3.32E-02	-0.8047906
THY1	-0.13514	13.54559	-3.70723	0.0017495	4.22E-02	-1.2309506
RIC8B	-0.13621	9.071477	-4.36924	0.0004177	0.0196763	0.1295919
4930455C21RIK	-0.13652	8.093514	-3.59687	0.0022232	4.74E-02	-1.4575509
5730410I19RIK	-0.13741	9.275464	-4.42989	0.0003667	1.82E-02	0.2535134
AGXT2L1	-0.1376	7.871282	-3.71662	0.0017142	4.21E-02	-1.2116412
LAMP2	-0.13998	9.228855	-4.46841	0.0003376	0.0175515	0.3320941
ATP13A2	-0.14187	8.678051	-4.20388	0.0005963	2.40E-02	-0.2092578
EIF4G2	-0.14256	7.92252	-4.124	0.0007086	0.0266828	-0.3733403
LOC545013	-0.14327	7.664849	-4.29334	0.0004917	0.0218025	-0.0257699
ST13	-0.14345	7.741379	-3.6714	0.001891	0.0435716	-1.3045615
YLPM1	-0.14366	8.481868	-3.93811	0.0010599	0.0328595	-0.7558345
ZFR	-0.1442	7.367507	-4.46288	0.0003417	1.76E-02	0.3208083
KLHL5	-0.14458	7.651154	-3.75628	0.0015727	0.0403809	-1.1300774
TRIP12	-0.14485	9.477423	-3.72606	0.0016794	0.0417977	-1.1922231
TCERG1	-0.14694	7.942802	-3.96305	0.0010041	0.0321086	-0.7044908
DERL2	-0.14696	7.459031	-3.64646	0.0019963	4.47E-02	-1.3557899
AMIGO1	-0.14709	8.667317	-4.05212	0.0008278	2.89E-02	-0.5211687
SGTB	-0.14731	10.29943	-3.57015	0.0023559	0.0493104	-1.5123298
1110008P14RIK	-0.14774	12.1151	-3.93055	0.0010774	0.0328595	-0.7713837
UBE3A	-0.14794	7.899536	-4.73316	0.0001921	0.0131252	0.8691535
UHRF1BP1L	-0.14896	9.539671	-3.67409	0.00188	0.0435078	-1.2990402
LOC216963	-0.15043	8.024407	-3.63095	0.0020646	0.0456537	-1.3876382

STAM2	-0.15119	7.866224	-3.68604	0.0018319	0.0430548	-1.2744961
ACOT11	-0.15203	8.36027	-4.1899	0.0006146	0.0242902	-0.2379526
HIST1H2BN	-0.15303	7.967064	-4.73609	0.0001909	0.0131252	0.8750789
LMO2	-0.15336	9.382113	-3.65875	0.0019437	0.0440806	-1.3305509
EIF2C2	-0.15399	7.580781	-4.21038	0.000588	2.40E-02	-0.1958997
FIP1L1	-0.15481	7.723884	-4.92736	0.0001275	0.0107935	1.2591642
NOTCH1	-0.15571	8.00586	-4.12276	0.0007105	0.0266828	-0.3758884
GPC5	-0.15572	8.91444	-4.70567	0.0002036	1.32E-02	0.8136652
PRPF38A	-0.15602	8.108873	-4.62013	0.0002442	1.45E-02	0.6405472
2810405J04RIK	-0.15666	9.856258	-4.07348	0.0007904	0.0282758	-0.4772287
TGFB2	-0.15685	7.600033	-3.62408	0.0020956	0.0458891	-1.4017263
6430548M08RIK	-0.15839	10.96646	-3.68053	0.0018539	4.32E-02	-1.2858201
ASH1L	-0.15929	7.909971	-3.90036	0.0011503	0.0338716	-0.8335529
CHFR	-0.16045	9.261868	-4.51924	0.0003028	0.0162228	0.4356127
SLC39A12	-0.16046	9.773551	-5.13289	8.30E-05	0.0090287	1.6676177
CAMKK2	-0.16112	9.581449	-3.85745	0.0012625	0.0357202	-0.9218792
RRN3	-0.16118	8.707953	-3.85942	0.0012572	0.035664	-0.9178397
2410015M20RIK	-0.16215	8.777333	-4.27265	0.0005141	0.0223267	-0.0681598
WDR17	-0.1636	8.686966	-3.65808	0.0019465	0.0440806	-1.3319284
9130422G05RIK	-0.16393	8.9559	-4.34205	0.0004428	2.05E-02	0.0739861
TUBA3B	-0.16459	7.700108	-4.02529	0.0008774	2.95E-02	-0.5763884
IL6ST	-0.1652	7.659632	-4.06276	0.000809	0.0284574	-0.4992828
WNK1	-0.166	8.723069	-4.09934	0.0007474	0.0275785	-0.4240442
GPR17	-0.16648	8.459968	-4.43055	0.0003662	1.82E-02	0.2548465
FUT8	-0.16676	8.659682	-5.45916	4.24E-05	0.0062998	2.3056735
UCHL5	-0.16853	9.556969	-3.77832	0.0014992	0.0392299	-1.0847476
LOC433801	-0.16865	7.84273	-3.83972	0.0013121	0.0364379	-0.9583774
SLC40A1	-0.17149	8.859273	-4.27699	0.0005093	2.22E-02	-0.0592762
MAPRE3	-0.17152	8.088653	-4.3312	0.0004532	0.0208862	0.0517787
OPN3	-0.17282	7.708122	-4.04011	0.0008497	2.90E-02	-0.5458898
CENPB	-0.17322	10.12591	-3.91946	0.0011036	0.0331738	-0.7942286
DEDD2	-0.17334	8.140133	-4.00613	0.0009146	3.02E-02	-0.6158175
NRXN3	-0.17422	10.13802	-3.94982	0.0010333	3.22E-02	-0.7317259
FAM84A	-0.1746	7.918033	-4.11833	0.0007173	0.0267929	-0.385006
TIMP4	-0.17468	8.305034	-4.95759	0.0001197	1.08E-02	1.3195338
CTSB	-0.17506	13.04462	-3.56147	0.0024007	0.0498711	-1.5301199
TSPYL4	-0.17512	7.585425	-3.86561	0.0012404	0.0355648	-0.9050893
SLCO1C1	-0.1755	9.342314	-4.25902	0.0005294	0.0226201	-0.0961182
NFIC	-0.17706	8.22565	-4.77678	0.0001751	0.012746	0.9570706
RGS4	-0.17736	12.45033	-4.09574	0.0007533	0.0275924	-0.43144
NARS	-0.17746	10.32798	-3.62159	0.002107	0.0458891	-1.4068518
ENPP2	-0.17829	11.01117	-3.7567	0.0015713	0.0403809	-1.1292176
HR	-0.17908	8.828072	-4.45946	0.0003442	1.76E-02	0.3138301

SLC30A4	-0.17947	7.703629	-4.48578	0.0003253	1.72E-02	0.3674846
GNAS	-0.17948	10.05977	-4.84276	0.0001524	0.0118285	1.0897291
NCAPD2	-0.18245	8.053598	-4.67424	0.0002177	1.37E-02	0.7501303
LOC329575	-0.18285	9.201205	-3.75633	0.0015725	4.04E-02	-1.1299724
LOC100045780	-0.18303	7.930952	-4.7066	0.0002032	0.0131817	0.8155534
WDR23	-0.18308	7.938186	-4.25502	0.000534	0.0227241	-0.104325
OSBPL6	-0.18406	9.332504	-5.1773	7.57E-05	8.81E-03	1.7552247
2610044O15RIK	-0.18444	8.103242	-4.15411	0.000664	2.56E-02	-0.3114715
ALDH6A1	-0.18479	9.784519	-4.10223	0.0007428	2.75E-02	-0.4181125
CASC4	-0.18573	8.509575	-3.95192	0.0010286	3.22E-02	-0.7273919
LOC100046796	-0.18732	8.172219	-4.60364	0.0002529	0.0145855	0.6071083
TCFE3	-0.18838	7.804171	-4.06346	0.0008078	0.0284574	-0.4978415
PHTF2	-0.18839	8.132256	-4.86122	0.0001465	0.0118285	1.126759
LRRTM3	-0.18854	8.179193	-5.48004	4.06E-05	0.0062998	2.3460516
CBLN2	-0.18928	8.078496	-4.73127	0.0001928	0.0131252	0.8653565
AADACL1	-0.18929	11.87675	-3.61261	0.0021485	0.0464613	-1.4252732
PDZD8	-0.18971	7.982452	-4.02129	0.000885	0.0295547	-0.5846035
SKIV2L	-0.18984	9.101456	-4.99182	0.0001114	1.06E-02	1.3877662
PPM1L	-0.19004	10.43274	-4.31997	0.0004643	0.0212119	0.0287745
CRIPT	-0.19024	8.346967	-6.30148	7.95E-06	2.35E-03	3.8853216
TXNL4	-0.1905	9.157446	-3.60158	0.0022005	0.0472958	-1.4478879
CYR61	-0.19129	7.222309	-4.78526	0.000172	1.27E-02	0.974155
PTN	-0.19226	11.83842	-4.61804	0.0002453	0.0145194	0.6363232
ARCN1	-0.19386	7.924603	-4.85524	0.0001484	0.0118285	1.1147564
YTHDF1	-0.19509	9.730857	-3.98897	0.0009492	0.0308213	-0.6511362
1810035L17RIK	-0.19659	10.27342	-4.60511	0.0002521	0.0145855	0.6100953
KLHL7	-0.19772	10.11401	-6.27652	8.35E-06	2.35E-03	3.8400081
HSP105	-0.20031	11.51016	-4.7407	0.000189	1.31E-02	0.8843754
TPPP	-0.20043	10.8174	-3.68649	0.00183	0.0430548	-1.2735587
ALKBH8	-0.20129	8.305834	-3.56189	0.0023985	0.0498711	-1.5292702
TOP2B	-0.20356	7.583709	-4.91279	0.0001315	0.0109242	1.2300363
PRMT8	-0.20447	9.228719	-4.32778	0.0004566	0.0209491	0.0447725
BCAS2	-0.20459	9.930868	-3.85349	0.0012735	0.0358366	-0.9300381
FGF1	-0.20547	9.163044	-4.42155	0.0003733	1.83E-02	0.2364772
ABHD3	-0.20567	8.452553	-3.89907	0.0011535	3.39E-02	-0.8362093
ZEB2	-0.20682	9.562137	-4.40921	0.0003833	0.0186406	0.211284
SEPT9	-0.20816	8.339702	-4.40372	0.0003878	0.018717	0.200074
ZMAT3	-0.20863	10.58692	-4.21184	0.0005861	2.40E-02	-0.1929023
RASSF3	-0.21014	8.629859	-3.77624	0.001506	0.0392414	-1.0890268
ACSBG1	-0.21048	8.492413	-6.05039	1.30E-05	0.0031835	3.4252878
MYO6	-0.21149	10.00877	-5.01405	0.0001063	0.0103889	1.4320228
HIST1H2BC	-0.21242	10.16474	-3.5685	0.0023644	0.0493104	-1.5157184
CCK	-0.21249	12.17367	-3.68921	0.0018193	4.30E-02	-1.2679694

ANK3	-0.21263	8.759347	-4.02654	0.000875	0.0294843	-0.5738069
SYNGR1	-0.21272	9.280637	-4.56965	0.0002719	1.53E-02	0.538114
TM9SF2	-0.21281	8.777746	-4.35461	0.000431	0.0201247	0.0996795
PI4KA	-0.21391	10.17242	-3.82154	0.0013649	3.72E-02	-0.9957929
CACYBP	-0.21484	8.237526	-4.3102	0.0004742	2.14E-02	0.0087758
PAFAH1B1	-0.2163	9.107184	-4.92709	0.0001276	0.0107935	1.2586269
NECAB1	-0.21651	8.96109	-3.95495	0.0010218	0.0322229	-0.7211596
ID4	-0.21749	8.860939	-3.69595	0.0017929	4.27E-02	-1.254129
SMARCC2	-0.21829	9.053504	-4.97986	0.0001142	1.06E-02	1.3639485
PTPRA	-0.22001	8.986799	-3.69976	0.0017781	0.042453	-1.2463062
MDH1	-0.22043	8.886961	-3.79823	0.0014358	0.038148	-1.0437762
GPR37L1	-0.22064	9.993285	-6.26886	8.47E-06	2.35E-03	3.8260906
ZNRF1	-0.22095	8.790423	-5.15128	7.99E-05	0.008814	1.7039228
PPP2R2C	-0.22121	10.27926	-3.62116	0.002109	4.59E-02	-1.4077325
PPP1R3C	-0.22135	10.51775	-4.48327	0.0003271	0.0171612	0.3623723
NOL4	-0.22153	9.808341	-4.19657	0.0006058	2.42E-02	-0.2242635
FGF12	-0.22166	9.618374	-5.05006	9.86E-05	9.82E-03	1.5035791
PTGES3	-0.22276	9.397217	-5.66861	2.77E-05	5.32E-03	2.7079649
RELL2	-0.22289	10.52928	-4.00425	0.0009183	0.0301886	-0.6196741
ZFP36L1	-0.22334	9.402585	-4.69833	0.0002068	0.013307	0.7988327
OLIG1	-0.22351	12.08359	-4.56012	0.0002775	0.0153781	0.5187607
CLCN4-2	-0.2237	8.232154	-4.6292	0.0002395	0.014362	0.6589501
NR1D2	-0.22391	8.714713	-4.07918	0.0007807	0.0280241	-0.465503
RFWD2	-0.2247	8.863298	-3.94926	0.0010345	3.22E-02	-0.7328782
PTPRB	-0.2261	8.660836	-4.08625	0.0007689	0.027788	-0.4509738
ALDH1A1	-0.22645	7.92979	-4.94723	0.0001223	0.0107935	1.2988628
PMPCB	-0.2269	8.513254	-4.34191	0.0004429	0.0205002	0.0736985
HSF2	-0.22717	7.94058	-3.6506	0.0019784	4.47E-02	-1.3472898
HIST1H2BJ	-0.22728	8.773123	-4.43713	0.000361	0.0181667	0.2682866
4931406C07RIK	-0.22746	8.329672	-5.39686	4.81E-05	0.0065977	2.1848783
CLSTN1	-0.22848	10.08447	-5.29847	5.89E-05	7.49E-03	1.9930972
PRKAR1B	-0.22906	10.75021	-4.39788	0.0003927	1.88E-02	0.1881381
PIK3CA	-0.22995	7.847088	-4.20432	0.0005957	2.40E-02	-0.2083352
SEC11C	-0.23069	9.803964	-3.73005	0.0016649	0.0416193	-1.1840186
GAD1	-0.23138	12.32129	-4.93249	0.0001261	0.0107935	1.2694124
PTEN	-0.23151	8.296044	-4.76968	0.0001778	1.29E-02	0.9427776
P2RY12	-0.23327	8.52596	-5.24954	6.52E-05	8.00E-03	1.8972582
ATAD1	-0.23347	9.886556	-4.75587	0.0001831	1.30E-02	0.9149489
EVL	-0.23424	10.05722	-5.19917	7.23E-05	0.0085762	1.7983017
NDRG2	-0.23447	9.481645	-4.73915	0.0001897	0.0131252	0.8812355
HSD11B1	-0.2346	9.321022	-5.74456	2.38E-05	4.78E-03	2.8523374
TOMM70A	-0.2363	9.464776	-3.74169	0.0016233	0.0411081	-1.1600816
ARNT2	-0.23669	8.717599	-3.80193	0.0014243	0.038148	-1.0361519

LOC100047935	-0.23718	12.50334	-3.64889	0.0019858	0.0447271	-1.3508061
SNX1	-0.23848	8.491607	-3.95628	0.0010189	3.22E-02	-0.7184235
TBC1D19	-0.23871	8.628206	-4.54541	0.0002864	0.0157401	0.4888538
RGS10	-0.23872	9.908404	-5.52722	3.69E-05	6.08E-03	2.4370415
KCTD3	-0.23901	9.957426	-4.71968	0.0001976	0.0131817	0.8419622
SELK	-0.23936	10.29669	-4.79476	0.0001686	1.25E-02	0.9932747
SCG3	-0.23951	11.00394	-4.02795	0.0008723	2.95E-02	-0.5709095
D14ERTD171E	-0.23971	11.00192	-4.56329	0.0002756	1.54E-02	0.5252039
NPY	-0.23972	11.02052	-6.28426	8.22E-06	0.002353	3.8540794
PMP22	-0.23996	8.672352	-5.8964	1.76E-05	4.12E-03	3.138509
PPP2R2B	-0.23996	9.408388	-3.71488	0.0017206	0.0420782	-1.215218
TESK1	-0.24003	9.737184	-4.60787	0.0002507	1.46E-02	0.6156928
SEPP1	-0.24341	8.726684	-5.15626	7.91E-05	0.008814	1.7137436
KIRREL3	-0.24347	8.251422	-4.81584	0.0001613	0.0121551	1.0356649
GLRX2	-0.25017	8.893965	-4.87034	0.0001437	1.18E-02	1.145037
NLGN1	-0.25036	8.141828	-4.2005	0.0006007	0.0241015	-0.2161772
PHF14	-0.25092	7.979481	-5.59778	3.20E-05	5.77E-03	2.5725855
GSTM5	-0.25106	11.46692	-4.13677	0.0006893	0.0261664	-0.3471033
LOC100044468	-0.2512	11.05575	-4.26011	0.0005282	0.0226201	-0.0938708
RWDD4A	-0.25139	8.637847	-4.85142	0.0001496	0.0118285	1.10711
LOC100047794	-0.25142	9.243414	-3.85526	0.0012686	0.0357945	-0.9263898
TJP1	-0.25191	8.397151	-4.0019	0.000923	0.0302488	-0.6245195
ITGB5	-0.25239	8.32545	-6.29233	8.10E-06	0.002353	3.8687185
AK1	-0.25505	9.469627	-3.58456	0.0022834	0.0482896	-1.4828039
RORA	-0.26033	8.983488	-5.44226	4.39E-05	0.0063204	2.2729668
MDH2	-0.26061	10.05894	-4.60578	0.0002518	1.46E-02	0.6114502
NDUFB2	-0.26183	8.82696	-4.05168	0.0008286	0.0288505	-0.5220875
TYKI	-0.26287	8.792128	-6.66794	3.96E-06	1.99E-03	4.5393793
CCNG2	-0.26317	9.25757	-5.02891	0.0001031	0.0101654	1.4615605
RAD23B	-0.26343	10.35507	-5.17238	7.65E-05	0.008814	1.745544
LOC100041703	-0.26508	7.832738	-4.28251	0.0005033	0.022131	-0.0479632
RHBDL2	-0.26528	10.20887	-4.02472	0.0008784	0.0294843	-0.5775547
IDH2	-0.26607	9.18773	-3.87099	0.001226	3.53E-02	-0.8940052
ALDOA	-0.2674	12.53948	-5.77088	2.26E-05	0.0046716	2.9021697
DYNLT3	-0.26858	11.61523	-5.4194	4.60E-05	6.38E-03	2.2286402
A530082C11RIK	-0.27033	8.569516	-5.29941	5.88E-05	0.0074912	1.9949395
VCL	-0.27214	9.080144	-4.52691	0.0002979	1.62E-02	0.4512231
ADAM9	-0.27539	8.772647	-6.07587	1.23E-05	3.10E-03	3.4724016
GPM6B	-0.27571	8.30149	-4.57623	0.0002681	0.0152134	0.5514867
LOC100044177	-0.27742	8.509613	-6.58919	4.59E-06	0.0020618	4.4005792
SNAP91	-0.28482	8.62884	-3.87655	0.0012113	3.51E-02	-0.8825673
ACTR3	-0.28506	11.12468	-3.93403	0.0010693	3.29E-02	-0.7642283
SMPD4	-0.28575	8.127185	-3.64671	0.0019952	0.0447271	-1.3552777

SCN1A	-0.28904	10.25208	-5.26079	6.37E-05	0.0079058	1.9193187
UBE2J1	-0.29177	8.766111	-5.52885	3.68E-05	6.08E-03	2.4401853
DST	-0.29372	9.687143	-3.75176	0.0015882	4.07E-02	-1.1393895
C1QL3	-0.29422	8.877394	-5.78193	2.21E-05	4.67E-03	2.9230802
KPNB1	-0.29714	10.22433	-4.6702	0.0002195	0.0137081	0.7419639
SLC7A10	-0.29735	8.636367	-8.23012	2.47E-07	3.80E-04	7.092891
6330577E15RIK	-0.29796	8.563992	-4.35598	0.0004297	2.01E-02	0.1024866
GPM6A	-0.29976	10.32941	-6.67068	3.94E-06	0.0019885	4.5441809
STMN1	-0.30016	10.84226	-3.68246	0.0018461	0.0431884	-1.2818468
KCNK2	-0.3005	8.964489	-5.35921	5.20E-05	7.04E-03	2.1116404
SEL1L	-0.30286	8.589124	-5.56193	3.44E-05	6.04E-03	2.5038057
PLA2G7	-0.30305	8.286953	-3.78405	0.0014807	0.0389665	-1.0729616
GLRX3	-0.30559	9.166401	-4.64004	0.0002341	0.0142703	0.6809137
ID2	-0.31113	9.266153	-3.6735	0.0018824	0.0435078	-1.3002596
S100B	-0.31587	7.766454	-7.19091	1.51E-06	1.14E-03	5.436483
SEPT7	-0.31839	8.206913	-5.2808	6.11E-05	7.68E-03	1.9585222
AI314180	-0.31899	9.368344	-4.71678	0.0001989	1.32E-02	0.8361058
SLC12A5	-0.31938	8.211898	-4.77945	0.0001741	1.27E-02	0.9624529
IAP	-0.32293	11.19885	-6.45788	5.89E-06	2.22E-03	4.1670076
TRIM37	-0.32304	11.01737	-3.83119	0.0013366	3.67E-02	-0.9759428
GLUL	-0.32372	8.428036	-4.71155	0.0002011	1.32E-02	0.8255373
NDRG4	-0.33294	12.05689	-3.83081	0.0013377	3.67E-02	-0.9767267
FAM134A	-0.33294	10.51222	-6.48357	5.61E-06	2.22E-03	4.2129138
MEGF9	-0.33433	9.484822	-4.04453	0.0008415	2.89E-02	-0.5367853
A030009H04RIK	-0.33485	9.0145	-3.99737	0.0009321	3.04E-02	-0.633838
ABCC10	-0.34543	7.645496	-5.23083	6.77E-05	8.12E-03	1.8605362
PGRMC1	-0.34944	10.40578	-3.66107	0.0019339	4.41E-02	-1.3257865
FOS	-0.35902	9.766477	-5.655	2.85E-05	5.37E-03	2.6820011
MAP1LC3B	-0.36605	9.973792	-3.80467	0.0014158	3.81E-02	-1.0305294
LOC100048530	-0.37395	8.460216	-6.95259	2.33E-06	1.54E-03	5.0329778
NPAS4	-0.37975	8.183955	-9.78878	2.11E-08	1.11E-04	9.2791836
SETD3	-0.39251	9.387763	-3.68848	0.0018222	4.30E-02	-1.2694829
MAP2K1	-0.40863	9.565388	-6.11314	1.15E-05	2.95E-03	3.5411557
LPGAT1	-0.42389	8.435119	-4.84656	0.0001511	1.18E-02	1.0973414
HUWE1	-0.42614	9.578061	-5.64392	2.91E-05	0.0053943	2.6608438
XLR4A	-0.43142	8.818481	-8.95086	7.64E-08	1.61E-04	8.1463969
PTGDS	-0.43295	9.929905	-5.5326	3.65E-05	6.08E-03	2.4474026
JAK1	-0.44352	8.622206	-6.86547	2.74E-06	1.70E-03	4.883258
DUSP1	-0.46451	10.59118	-6.61164	4.40E-06	2.06E-03	4.4402496
SPARCL1	-0.46679	10.72649	-5.15217	7.97E-05	0.008814	1.7056765
ARC	-0.49701	10.64455	-7.30596	1.23E-06	9.95E-04	5.6280925

DEGs in resilient mPFC

adj.P.Value < 0.05

DEGup 204

DEGdown 214

Gene	logFC	AveExpr	t	P.Value	adj.P.Val	B
CTGF	0.754879	10.55685	3.879257	0.001204	0.037829	-0.87661
HBA-A1	0.664221	12.60395	7.678997	6.33E-07	5.57E-04	6.23465
GBP2	0.588043	7.660565	9.766155	2.18E-08	1.15E-04	9.248671
SLA	0.573119	8.890923	5.172347	7.65E-05	8.77E-03	1.74561
COL6A1	0.464607	9.225453	5.598795	3.19E-05	5.52E-03	2.574572
RGS9	0.435953	8.980841	5.266522	6.29E-05	0.007938	1.930668
HBB-B1	0.383116	8.799639	3.873705	0.001219	3.78E-02	-0.88804
AHI1	0.379121	10.57708	8.426566	1.78E-07	2.09E-04	7.386834
IGTP	0.36929	7.656577	9.389765	3.86E-08	1.28E-04	8.75E+00
RAI14	0.367945	8.747604	4.236513	0.000556	0.025171	-0.14195
SEZ6	0.354107	9.334715	5.138415	8.20E-05	9.03E-03	1.678669
IIGP2	0.353915	7.653213	8.983659	7.26E-08	0.000128	8.191642
GRP	0.350456	8.925961	5.29567	5.93E-05	0.007721	1.987725
NTSR1	0.343662	8.087294	4.8241	0.000158	1.23E-02	1.052461
DPYSL5	0.342583	7.951066	8.204122	2.58E-07	2.72E-04	7.052798
IFITM3	0.335355	8.690011	5.007851	0.000108	1.03E-02	1.419851
LOC10004	0.32722	9.874884	4.522108	0.000301	1.71E-02	0.441727
CDH13	0.314059	8.439697	9.044727	6.59E-08	1.28E-04	8.277166
SAMD14	0.309946	9.194004	6.79189	3.14E-06	0.001743	4.755613
CRYM	0.309089	11.54698	4.70963	0.000202	0.014204	0.821896
COL5A1	0.307215	8.052943	7.008434	2.10E-06	1.31E-03	5.128011
ASAH3L	0.306114	7.969307	6.409574	6.46E-06	2.53E-03	4.080242
ENSMUSG	0.297461	7.882982	5.005886	0.000108	1.03E-02	1.415943
PTPRU	0.295064	8.320852	4.616639	0.000246	0.015119	0.633731
FEZF2	0.285482	9.724721	5.556439	3.48E-05	5.73E-03	2.493307
BCL11B	0.283545	10.63047	3.690625	0.001814	4.79E-02	-1.26465
NRBP2	0.282766	10.27506	6.022231	1.37E-05	3.62E-03	3.373052
RASL10A	0.282602	9.588202	6.251112	8.77E-06	2.93E-03	3.793685
LY6A	0.27838	9.565472	5.842394	1.96E-05	4.56E-03	3.037082
COTL1	0.276797	9.645081	5.122318	8.48E-05	9.13E-03	1.646866
DRD1A	0.276574	8.898292	3.759078	0.001563	4.40E-02	-1.12392
KLF5	0.274188	8.790538	5.392917	4.85E-05	0.006921	2.177307
FDPS	0.272956	10.83188	4.847234	0.000151	0.011868	1.098906
SMAD3	0.269576	8.05612	5.125047	8.44E-05	0.009132	1.652259
FXYD6	0.268861	12.80224	4.950833	0.000121	0.010676	1.30623
D12ERTD6	0.26779	9.969361	4.113313	0.000725	0.029809	-0.39497
HIST1H2AI	0.267699	7.875641	8.649114	1.24E-07	0.000163	7.713722

PADI6	0.263685	7.451042	4.302671	0.000482	2.29E-02	-0.00634
SYT5	0.263654	10.3186	5.802692	2.12E-05	4.59E-03	2.962275
RAMP3	0.26293	8.392446	5.211627	7.05E-05	8.27E-03	1.822927
DDAH1	0.256774	11.28131	3.71937	0.001704	0.04656	-1.20557
STXBP2	0.2564	9.009519	3.935616	0.001066	0.035927	-0.76058
DDR1	0.256168	8.746017	5.444596	4.37E-05	6.49E-03	2.277557
SGK1	0.254614	11.44792	6.124988	1.12E-05	0.003379	3.562872
HIST1H2AI	0.253116	8.040344	4.566683	0.000274	0.016134	0.532349
HIST1H2A(	0.249375	10.24161	4.301683	0.000483	0.022855	-0.00836
PLOD3	0.249374	9.058599	5.104438	8.80E-05	0.009141	1.611504
DGKG	0.24878	10.63397	5.83452	1.99E-05	0.004561	3.022265
PDE1B	0.245786	10.87632	5.661972	2.81E-05	0.004978	2.695324
WSCD1	0.245043	8.908179	4.635572	0.000236	0.015113	0.672099
KLF16	0.240713	8.781993	4.227722	0.000566	2.55E-02	-0.15999
TFG	0.238861	9.941635	3.806442	0.00141	0.04123	-1.02648
SCAMP2	0.237762	8.778437	5.137632	8.22E-05	9.03E-03	1.677123
SLC9A3R2	0.237439	8.865386	5.117592	8.57E-05	0.009132	1.637523
DBI	0.234461	11.25175	5.100167	8.88E-05	0.009141	1.603052
PRMT2	0.234318	11.1476	5.195364	7.29E-05	8.45E-03	1.790938
ZER1	0.233769	12.35393	4.966356	0.000117	0.010676	1.337197
AGPAT4	0.232422	9.419419	3.653838	0.001965	0.049597	-1.34022
JOSD2	0.232159	9.284611	4.643253	0.000232	0.015028	0.687658
RPS26	0.232126	11.85228	4.4118	0.000381	0.020113	0.216864
XRCC1	0.23129	8.464533	5.731443	2.44E-05	4.86E-03	2.827463
KCTD10	0.23088	9.086462	4.894912	0.000137	0.011343	1.194456
1700027N	0.229076	8.011994	5.683394	2.69E-05	0.004956	2.736143
CASC3	0.228791	10.04453	3.914493	0.001116	3.66E-02	-0.80407
LMAN2L	0.22869	9.210995	5.523274	3.72E-05	5.95E-03	2.429505
LOC10004	0.227385	7.312192	6.805442	3.06E-06	1.74E-03	4.779134
SETD1B	0.226028	10.51562	3.749723	0.001595	0.044654	-1.14316
ARPC5	0.224685	11.12969	5.24693	6.55E-05	8.00E-03	1.892259
SPRED1	0.223292	11.63264	5.717807	2.51E-05	0.004907	2.801582
DRCTNNB	0.223107	8.362208	5.79994	2.13E-05	4.59E-03	2.957083
METTL3	0.223015	9.368955	5.951378	1.58E-05	0.004058	3.241251
MED6	0.222456	8.938034	6.472913	5.73E-06	2.42E-03	4.193701
DNAJB2	0.221406	9.228935	4.12038	0.000714	0.029809	-0.38044
MMP24	0.219163	9.045771	5.410081	4.69E-05	0.006773	2.210642
PRR7	0.217806	9.384036	5.687593	2.67E-05	0.004956	2.744136
FAM171A2	0.216884	10.38884	3.946216	0.001041	0.035683	-0.73877
HMGN2	0.215461	11.39921	3.670704	0.001894	0.04851	-1.30558
CCDC3	0.21541	9.109321	4.425504	0.00037	0.019728	0.244844
TRA2A	0.214269	10.53561	3.734398	0.001649	0.045561	-1.17468
FIGF	0.214219	7.855367	6.194621	9.79E-06	3.04E-03	3.690601

LOC10004(	0.211929	11.75657	3.798446	0.001435	4.17E-02	-1.04293
GBP3	0.211678	7.725706	6.604028	4.46E-06	2.01E-03	4.426593
AMIGO2	0.211462	8.551371	4.045602	0.00084	0.032337	-0.53422
LASS5	0.209702	9.452111	5.141274	8.16E-05	0.009033	1.684314
CXX1C	0.209459	10.42897	4.648798	0.00023	0.015028	0.698886
LOC10004!	0.208053	9.222131	3.912703	0.00112	0.03659	-0.80775
RFX1	0.207857	8.273137	4.865507	0.000145	0.01161	1.135554
FAM116B	0.20712	9.604264	4.729358	0.000194	0.013854	0.861718
RASAL1	0.206882	8.861191	4.023144	0.000881	3.33E-02	-0.58043
DLG4	0.205699	9.496925	4.11167	0.000728	0.029809	-0.39835
CBLN1	0.204016	8.541473	3.823809	0.001358	0.040262	-0.99074
SRP54	0.203745	8.205825	4.376794	0.000411	0.020949	0.14534
2610208M	0.203239	9.261427	4.049511	0.000833	0.032232	-0.52618
ERP29	0.201123	11.32563	3.854424	0.001271	3.88E-02	-0.92772
HSF1	0.199304	9.898833	4.916512	0.00013	0.010934	1.237669
HNRNPK	0.197537	8.520658	4.418271	0.000376	0.019935	0.230077
DENND2A	0.197443	8.295975	4.348777	0.000436	0.021322	0.088044
1500011H;	0.197184	10.14294	3.724352	0.001686	0.046323	-1.19533
KLF13	0.196669	10.30388	3.935873	0.001065	0.035927	-0.76006
HPCAL4	0.195933	13.09549	5.677306	2.72E-05	4.96E-03	2.724548
PACRG	0.194773	9.414803	4.844402	0.000152	0.011868	1.093223
GPN3	0.194117	8.406506	4.061117	0.000812	3.16E-02	-0.50231
SEMA4A	0.193774	10.29253	3.874244	0.001217	3.78E-02	-0.88693
DCLK2	0.191712	9.040562	3.718352	0.001708	0.04656	-1.20766
MID1	0.190901	7.855269	3.87565	0.001214	3.78E-02	-0.88403
C4B	0.190897	7.499789	4.950745	0.000121	0.010676	1.306054
RPS4Y2	0.188862	7.95517	4.246524	0.000544	0.02474	-0.12142
LOC10004(	0.188612	11.82016	3.957154	0.001017	0.035303	-0.71625
PYCR2	0.188531	9.024379	4.6808	0.000215	1.47E-02	0.763638
RCL1	0.187833	8.423886	4.981527	0.000114	0.010444	1.367438
ZFHX2	0.187236	10.14144	4.129729	0.0007	0.029539	-0.36123
HIST1H2AI	0.18693	7.994154	4.483322	0.000327	0.018068	0.362757
LSM3	0.186498	9.234061	4.127948	0.000703	0.029539	-0.36489
HRMT1L2	0.185616	10.48449	4.494736	0.000319	0.017777	0.386008
THSD4	0.183608	7.619463	4.882537	0.00014	0.011502	1.169679
LZTS2	0.183385	9.066074	3.659976	0.001939	0.049086	-1.32761
PSMB3	0.182978	11.29022	3.66235	0.001929	4.90E-02	-1.32274
EIF3D	0.182803	10.47309	4.010082	0.000907	0.033693	-0.60731
MAP3K3	0.18226	8.555405	3.662736	0.001927	0.049041	-1.32194
H3F3A	0.182258	10.16718	4.355855	0.00043	0.021197	0.102522
4931406P1	0.181975	9.157323	3.94658	0.001041	3.57E-02	-0.73801
STYX	0.181766	9.168237	4.186619	0.000619	0.026665	-0.24436
GSPT1	0.18176	10.82309	4.875625	0.000142	0.011502	1.155831

D430041B1	0.179947	9.123235	4.528459	0.000297	0.017077	0.454649
IFITM2	0.179023	7.779981	4.270236	0.000517	0.02392	-0.0728
UHRF2	0.178978	9.131067	4.684725	0.000213	0.014736	0.771575
MUM1	0.178381	9.767936	5.250449	6.51E-05	0.008005	1.899161
SETD1A	0.176067	8.685101	4.409208	0.000383	0.020125	0.211571
DDIT4	0.176011	8.683473	3.878703	0.001206	0.037829	-0.87775
TPR	0.175645	9.902227	3.665913	0.001914	0.048899	-1.31542
LOR	0.175076	9.268759	3.98071	0.000966	0.034588	-0.66776
RUNX1T1	0.174816	9.781986	3.840155	0.001311	3.96E-02	-0.95709
DMAP1	0.174714	8.756281	4.073384	0.000791	0.031015	-0.47708
2700094K1	0.174249	10.63579	4.117596	0.000718	0.029809	-0.38617
BEX4	0.174243	9.108064	4.048791	0.000834	3.22E-02	-0.52766
P140	0.173812	8.305353	4.392659	0.000397	0.020647	0.177764
RPS27A	0.173153	9.831559	4.671271	0.000219	0.014817	0.744368
GNB4	0.172854	7.85434	4.605403	0.000252	0.015371	0.610945
ST6GALNA	0.172481	9.782116	3.901703	0.001147	3.69E-02	-0.8304
SYVN1	0.171971	10.57354	4.469361	0.000337	0.018519	0.334304
CTNNAL1	0.17191	8.952682	4.813432	0.000162	0.012485	1.031028
MED30	0.171862	10.05659	3.94194	0.001051	0.035781	-0.74757
ZFP672	0.171264	9.711252	4.034234	0.000861	0.033022	-0.55761
ARHGEF15	0.171221	8.08675	4.632276	0.000238	0.015119	0.665423
CALN1	0.170225	12.02832	3.677461	0.001866	0.048272	-1.2917
D16ERTD4	0.170173	8.663599	4.803787	0.000165	0.012649	1.011638
MAS1	0.169246	8.797854	3.908857	0.001129	0.036669	-0.81567
TPST1	0.168802	9.578391	3.708994	0.001743	0.046798	-1.2269
THOC4	0.167052	9.268186	4.794388	0.000169	0.012719	0.992735
ACVR2B	0.163689	8.799062	4.752311	0.000184	0.013804	0.908006
RPS4X	0.163195	12.70981	3.734893	0.001647	0.045561	-1.17366
ST5	0.162886	8.930812	5.298388	5.89E-05	7.72E-03	1.99304
IFIT3	0.161767	7.826133	4.523045	0.0003	0.017077	0.443635
EXTL1	0.160807	8.809089	3.937899	0.00106	0.035927	-0.75589
RAB9B	0.159126	9.805159	4.439213	0.000359	0.019356	0.272822
IPPK	0.159102	8.711257	3.994015	0.000939	0.034092	-0.64038
TATDN2	0.158895	8.410132	3.916323	0.001111	3.66E-02	-0.8003
NUMA1	0.158411	7.735714	4.378718	0.000409	0.020949	0.149273
RBBP7	0.15825	8.381073	5.243497	6.60E-05	0.008005	1.885522
ATG16L1	0.157489	10.13511	3.993415	0.00094	0.034092	-0.64161
TSPAN33	0.155817	8.140744	3.930612	0.001077	0.036089	-0.77089
ETS2	0.155493	10.83293	3.952496	0.001027	0.035544	-0.72584
ALOXE3	0.154447	7.650103	4.255955	0.000533	2.45E-02	-0.10208
PSME2	0.154104	7.759331	3.659718	0.00194	4.91E-02	-1.32814
GSTP1	0.153725	10.36609	4.176425	0.000633	0.027142	-0.26529
ING4	0.152826	8.670024	3.795931	0.001443	0.041834	-1.04811

TPPP3	0.152722	7.8509	4.101369	0.000744	0.029973	-0.41953
PRDM4	0.151217	9.504693	4.574073	0.000269	0.016061	0.54736
IRF1	0.150983	7.651147	4.274084	0.000513	0.023827	-0.06491
PHF5A	0.150902	10.43582	4.372688	0.000415	2.10E-02	0.136946
RGS12	0.150282	8.360341	3.911268	0.001123	3.66E-02	-0.81071
COG1	0.149892	8.320809	4.087157	0.000767	0.030559	-0.44875
CACNG5	0.149089	7.778077	4.58217	0.000265	0.015965	0.563801
MAPK1	0.148079	11.3214	3.865642	0.00124	0.038154	-0.90463
GBL	0.147824	9.860339	3.683022	0.001844	4.79E-02	-1.28027
ZC3H3	0.147549	8.206165	3.880082	0.001202	0.037829	-0.87491
VPS25	0.146994	8.981466	4.329761	0.000455	0.021908	0.04913
MOXD1	0.146	7.645678	3.998847	0.000929	0.034092	-0.63043
NEGR1	0.144958	8.151532	3.753037	0.001584	0.044452	-1.13634
RFC5	0.143688	7.758241	4.497066	0.000318	0.017777	0.390752
B230373P(	0.143326	8.126521	4.000389	0.000926	0.034092	-0.62726
DHRS7B	0.142058	8.680331	4.215287	0.000582	0.025906	-0.18551
USE1	0.139822	10.1733	3.704687	0.001759	0.047118	-1.23575
SLC2A3	0.139046	11.33237	4.356945	0.000429	0.021197	0.104753
ZFX	0.13799	7.98917	4.138426	0.000687	0.029109	-0.34336
ANKS3	0.135533	8.460922	3.729339	0.001667	4.59E-02	-1.18508
2400006N(	0.134595	7.582371	3.713344	0.001726	4.66E-02	-1.21796
EG633640	0.133474	7.723142	3.738082	0.001636	0.045556	-1.1671
ZFP295	0.133243	8.179883	4.732229	0.000192	1.39E-02	0.867511
NFRKB	0.131299	8.219986	3.813281	0.00139	0.040962	-1.0124
PCDH21	0.130833	7.413735	3.830125	0.00134	0.040051	-0.97774
NARG1L	0.129786	7.907149	3.973501	0.000982	0.034756	-0.6826
PTBP1	0.12817	8.3364	3.789017	0.001465	0.042234	-1.06233
1200016B1	0.126183	8.376874	3.73615	0.001643	0.045561	-1.17107
LOC10004:	0.124322	8.847834	3.786439	0.001473	4.24E-02	-1.06763
POU2F1	0.123087	7.895745	3.764478	0.001545	0.04371	-1.11281
DHX38	0.122661	9.170918	3.972296	0.000984	0.034756	-0.68508
EMD	0.121483	8.159979	3.983455	0.000961	0.034588	-0.66211
DUS4L	0.119922	8.419626	3.915404	0.001113	0.03659	-0.80219
TMEM2	0.11867	7.896467	3.856223	0.001266	0.038722	-0.92402
CEBPB	0.116222	9.000959	3.888086	0.001181	0.03755	-0.85843
FBLN2	0.114698	7.533667	3.765901	0.00154	0.043692	-1.10989
5133400G(	0.111377	7.860185	3.711818	0.001732	0.04663	-1.2211
RUVBL1	0.109744	8.727501	3.808277	0.001405	0.04123	-1.0227
TMEM85	-0.12258	11.62307	-3.99655	0.000934	0.034092	-0.63516
SNX25	-0.12864	8.230302	-4.27491	0.000512	0.023827	-0.06322
AKR1C18	-0.13346	7.264306	-3.69117	0.001812	0.04785	-1.26352
HTRA1	-0.1355	9.349731	-3.67381	0.001881	0.048488	-1.2992
AGPAT9	-0.13619	7.277887	-4.10351	0.000741	0.029973	-0.41512

KCNG4	-0.13738	7.608095	-4.19945	0.000602	0.026471	-0.21801
CCDC111	-0.14064	7.508426	-3.6908	0.001813	4.79E-02	-1.26429
FIP1L1	-0.14163	7.723884	-4.11505	0.000722	0.029809	-0.39141
ZFP161	-0.14811	7.626112	-4.49337	0.00032	0.017777	0.383233
1200003C(	-0.15031	10.71943	-3.98046	0.000967	0.034588	-0.66828
9130213B(	-0.15136	9.488112	-4.92837	0.000127	0.010933	1.261364
LAPTM4B	-0.15276	7.557326	-3.89015	0.001176	0.03755	-0.85419
APLP2	-0.15398	12.43777	-3.78318	0.001483	0.042541	-1.07434
COX7A1	-0.15406	7.769965	-3.68334	0.001843	0.047873	-1.27961
LRRC14	-0.1541	7.257886	-3.86438	0.001244	0.038154	-0.90724
CADM2	-0.15425	8.689475	-4.18652	0.000619	0.026665	-0.24457
CSNRP3	-0.15484	9.542514	-4.01723	0.000893	0.033582	-0.59259
HISPPD1	-0.15746	7.835759	-4.61706	0.000246	0.015119	0.634591
MGLL	-0.15776	9.943782	-3.6983	0.001784	4.74E-02	-1.24888
GPR37L1	-0.15796	9.993285	-4.09686	0.000751	0.030038	-0.4288
LOC54501:	-0.1587	7.664849	-4.34139	0.000443	0.021465	0.072927
ABCA5	-0.15908	7.511068	-4.01652	0.000894	0.033582	-0.59407
NFIC	-0.15916	8.22565	-3.91966	0.001103	3.66E-02	-0.79342
NUDT18	-0.15916	10.70814	-3.96232	0.001006	0.035025	-0.70561
CAPN2	-0.15924	8.271079	-4.43999	0.000359	0.019356	0.274416
CPSF2	-0.15924	7.55814	-3.67316	0.001884	4.85E-02	-1.30054
HIVEP2	-0.15942	7.432392	-4.3593	0.000427	2.12E-02	0.10956
VAMP1	-0.15944	7.902481	-4.14944	0.000671	0.028655	-0.32073
NTNG1	-0.16146	8.583457	-3.96822	0.000993	0.034756	-0.69347
C130074G	-0.16287	7.887178	-3.72017	0.001701	4.66E-02	-1.20393
KLHL7	-0.16338	10.11401	-4.7344	0.000192	0.013854	0.871885
D4BWG09!	-0.16339	9.087012	-3.70221	0.001769	0.047156	-1.24085
TOB1	-0.16389	8.347271	-4.55574	0.00028	0.016423	0.510125
THY1	-0.16411	13.54559	-4.10972	0.000731	0.029809	-0.40236
TYROBP	-0.16641	7.65399	-3.71202	0.001731	0.04663	-1.22069
MARE	-0.16661	7.847496	-4.14705	0.000674	0.028687	-0.32563
ITGB5	-0.16841	8.32545	-3.83265	0.001332	0.039945	-0.97254
SLC30A4	-0.17042	7.703629	-3.88834	0.001181	3.76E-02	-0.8579
HSP105	-0.17206	11.51016	-3.71724	0.001712	0.04656	-1.20996
STAM2	-0.17226	7.866224	-3.8338	0.001329	0.039945	-0.97017
AMIGO1	-0.1733	8.667317	-4.35829	0.000428	0.021197	0.107494
VPS29	-0.1734	10.97247	-4.24761	0.000543	0.02474	-0.1192
RG9MTD1	-0.17357	8.487556	-4.92571	0.000128	0.010933	1.256046
FKBP7	-0.17449	7.412932	-5.56587	3.41E-05	5.73E-03	2.511431
FAM125A	-0.17535	8.708298	-3.87785	0.001208	3.78E-02	-0.8795
ACSBG1	-0.17768	8.492413	-4.66246	0.000223	0.014944	0.726547
TFRC	-0.17805	10.65107	-4.67842	0.000216	1.47E-02	0.758826
GJD2	-0.1791	8.00752	-5.0052	0.000108	0.010267	1.414585

5730469M	-0.17965	9.365089	-4.06336	0.000808	0.031579	-0.4977
9130422G(	-0.18002	8.9559	-4.35271	0.000433	0.021241	0.096099
1110006G(	-0.18171	8.167631	-3.91201	0.001122	0.03659	-0.80918
PLXDC1	-0.18174	8.814225	-4.1184	0.000717	0.029809	-0.38452
SGTB	-0.18209	10.29943	-4.02847	0.000871	0.033077	-0.56948
LRRC4C	-0.18257	7.886709	-3.87378	0.001219	0.037829	-0.88788
MAPRE3	-0.18289	8.088653	-4.21584	0.000581	0.025906	-0.18438
TIMP4	-0.18313	8.305034	-4.74454	0.000187	1.39E-02	0.892347
TMEM218	-0.18373	8.539665	-4.79955	0.000167	1.27E-02	1.003117
PSG23	-0.18376	9.27572	-3.89508	0.001164	0.037321	-0.84402
4930403O(	-0.18405	10.30541	-4.19011	0.000614	0.026665	-0.2372
GRSF1	-0.18488	10.11151	-4.38444	0.000404	0.020809	0.160959
TSPYL4	-0.18892	7.585425	-3.80682	0.001409	0.04123	-1.0257
CAMK2G	-0.18919	10.06756	-4.65296	0.000228	0.01502	0.70731
GLRX	-0.19052	10.94049	-4.64108	0.000234	0.015028	0.683265
GABRG2	-0.19208	9.855456	-6.3027	7.94E-06	2.93E-03	3.887397
DIAP3	-0.19223	7.760914	-4.20474	0.000595	0.02628	-0.20715
PAFAH1B1	-0.19288	9.107184	-4.01083	0.000905	0.033693	-0.60578
LOC10004	-0.19297	12.02162	-4.09699	0.000751	0.030038	-0.42852
OSBPL6	-0.19315	9.332504	-4.95938	0.000119	0.010676	1.323277
GRIN1	-0.19335	8.440663	-4.22739	0.000567	0.025453	-0.16067
CDV3	-0.19366	10.72005	-3.76903	0.00153	4.35E-02	-1.10345
SKIV2L	-0.1941	9.101456	-4.65907	0.000225	1.49E-02	0.719688
CRIPT	-0.19449	8.346967	-5.88087	1.81E-05	0.004349	3.109367
MRPS18C	-0.19459	10.45204	-3.83923	0.001313	0.039604	-0.959
SEPP1	-0.19499	8.726684	-3.77073	0.001524	4.35E-02	-1.09995
LOC10004	-0.19602	12.44281	-3.94618	0.001041	0.035683	-0.73883
FGF1	-0.19609	9.163044	-3.8521	0.001277	0.038846	-0.93252
UBE3A	-0.19785	7.899536	-5.77814	2.23E-05	0.004647	2.915909
RGS7BP	-0.19793	13.05924	-5.28005	6.12E-05	7.88E-03	1.957163
JDP2	-0.19891	9.195855	-4.4484	0.000352	0.019269	0.291571
STMN3	-0.19989	11.12876	-4.07957	0.00078	0.030812	-0.46435
PCDHA6	-0.20002	10.68033	-3.9944	0.000938	0.034092	-0.63959
ANXA5	-0.20008	9.07179	-4.64668	0.000231	0.015028	0.69459
C1GALT1C	-0.2005	9.037114	-3.74647	0.001607	4.49E-02	-1.14984
LRRTM3	-0.20165	8.179193	-5.35032	5.30E-05	0.0074	2.094408
4931406C(	-0.20166	8.329672	-4.36772	0.000419	0.021157	0.126788
SYNGR1	-0.20412	9.280637	-4.00286	0.000921	0.034092	-0.62218
CBLN2	-0.20414	8.078496	-4.65828	0.000225	0.014944	0.718087
ZRANB1	-0.20487	8.718941	-3.87151	0.001225	0.037898	-0.89255
HNT	-0.20575	10.53084	-3.7599	0.00156	0.04399	-1.12223
DEPDC6	-0.20611	8.29381	-3.96823	0.000993	0.034756	-0.69344
GPM6A	-0.20637	10.32941	-4.19237	0.000611	0.026657	-0.23254

ARCN1	-0.20679	7.924603	-4.72773	0.000194	0.013854	0.858428
SLC39A12	-0.20684	9.773551	-6.04007	1.32E-05	0.003582	3.406123
ABCD2	-0.20878	8.007285	-4.26182	0.000526	0.024251	-0.09005
SELK	-0.21038	10.29669	-3.84705	0.001291	0.039161	-0.9429
PPP2R2D	-0.21068	10.08132	-3.68445	0.001838	4.79E-02	-1.27733
ZFP365	-0.2109	12.8063	-4.91611	0.000131	0.010934	1.236872
COX11	-0.21225	8.117175	-4.20503	0.000595	0.02628	-0.20656
NUDT4	-0.21397	10.55454	-4.30984	0.000475	0.02266	0.008336
ATAD1	-0.21414	9.886556	-3.9821	0.000963	0.034588	-0.6649
ABHD3	-0.2146	8.452553	-3.71385	0.001724	0.04663	-1.21692
TYKI	-0.21606	8.792128	-5.00296	0.000109	0.010267	1.410115
D0H4S114	-0.21731	11.51109	-4.39813	0.000393	0.020507	0.188947
ABHD12	-0.21753	9.445109	-5.65827	2.83E-05	4.98E-03	2.688272
PPP1R3C	-0.21794	10.51775	-4.02957	0.000869	0.033077	-0.56721
PIK3R4	-0.21821	8.103992	-3.68483	0.001837	0.047873	-1.27656
FAM84A	-0.22028	7.918033	-4.74298	0.000188	0.013854	0.889201
NECAB1	-0.22083	8.96109	-3.68242	0.001846	0.047873	-1.28152
TRPC3	-0.22376	8.770999	-4.98848	0.000112	0.010383	1.381295
KCNA2	-0.22386	9.17665	-4.85885	0.000147	0.011685	1.122212
HR	-0.22426	8.828072	-5.09812	8.92E-05	0.009141	1.59899
TOP2B	-0.22473	7.583709	-4.95102	0.000121	0.010676	1.30661
AGXT2L1	-0.22554	7.871282	-5.56138	3.44E-05	0.005734	2.502806
GPC5	-0.22636	8.91444	-6.2442	8.89E-06	0.002931	3.7811
TBC1D19	-0.22824	8.628206	-3.9674	0.000995	0.034756	-0.69516
OPN3	-0.23072	7.708122	-4.92378	0.000128	0.010933	1.252193
SLC23A2	-0.23149	8.969781	-4.0319	0.000865	0.03307	-0.56243
ARHGAP2(	-0.23354	10.02217	-5.09852	8.91E-05	0.009141	1.599795
GOPC	-0.23363	7.672498	-3.93317	0.001071	0.036003	-0.76561
1110008P1	-0.23426	12.1151	-5.68931	2.66E-05	4.96E-03	2.747398
SLC2A13	-0.2351	11.19407	-3.77444	0.001512	0.043238	-1.09231
EMB	-0.23589	9.947875	-4.27522	0.000511	0.023827	-0.06259
AI836003	-0.23615	8.946058	-5.7735	2.25E-05	4.65E-03	2.907131
CYB5	-0.23919	9.140136	-3.67104	0.001893	4.85E-02	-1.3049
PKIG	-0.23939	8.561073	-3.92078	0.0011	0.03659	-0.79113
ATP2B2	-0.24119	9.751188	-6.59026	4.58E-06	2.01E-03	4.402271
CASC4	-0.24225	8.509575	-4.70544	0.000204	0.014236	0.813426
GAD1	-0.24265	12.32129	-4.72193	0.000197	0.013931	0.846732
S100B	-0.24282	7.766454	-5.04611	9.94E-05	0.009994	1.495888
RFWD2	-0.2433	8.863298	-3.90366	0.001142	0.036858	-0.82637
PI4KA	-0.24359	10.17242	-3.97264	0.000983	0.034756	-0.68436
A530082C1	-0.24514	8.569516	-4.3869	0.000402	0.020801	0.165993
ADAM9	-0.24516	8.772647	-4.93761	0.000125	0.010883	1.279832
BC048546	-0.24741	9.075915	-7.95148	3.96E-07	0.00038	6.664412

RGS4	-0.24802	12.45033	-5.22856	6.81E-05	8.16E-03	1.85621
ASB13	-0.24817	11.1178	-4.6267	0.000241	0.015119	0.654123
KCNV1	-0.24855	9.38054	-4.34614	0.000439	0.021344	0.082658
5730409E0	-0.24888	8.124448	-4.01185	0.000903	0.033693	-0.60366
CLSTN1	-0.25019	10.08447	-5.29648	5.92E-05	0.007721	1.989316
PTN	-0.2503	11.83842	-5.48844	3.99E-05	0.006289	2.36233
KCNAB3	-0.25529	7.994942	-4.4391	0.00036	0.019356	0.272598
MANSC1	-0.25716	9.077627	-4.87354	0.000143	1.15E-02	1.151653
PMPCB	-0.25905	8.513254	-4.52525	0.000299	0.017077	0.448127
MRPL20	-0.25926	10.27261	-3.96781	0.000994	0.034756	-0.69431
NRN1	-0.26008	12.58454	-5.4577	4.25E-05	6.41E-03	2.302931
1110012J1	-0.26028	10.40025	-4.62154	0.000243	1.51E-02	0.643667
UCHL5	-0.26043	9.556969	-5.33008	5.52E-05	0.007567	2.05494
NFU1	-0.26099	8.015269	-4.5246	0.000299	0.017077	0.44679
PTGES3	-0.26123	9.397217	-6.06841	1.25E-05	3.56E-03	3.458552
LOC10004	-0.26379	11.05575	-4.08389	0.000773	0.03066	-0.45547
EVL	-0.2639	10.05722	-5.34725	5.33E-05	0.0074	2.088426
NDRG2	-0.2642	9.481645	-4.87463	0.000142	1.15E-02	1.153829
NLGN1	-0.26781	8.141828	-4.10165	0.000744	0.029973	-0.41894
DLD	-0.27088	9.035528	-3.70198	0.00177	4.72E-02	-1.24132
DYNLT3	-0.27147	11.61523	-5.00048	0.000109	1.03E-02	1.405183
COX7B	-0.2716	9.234677	-3.82714	0.001348	0.040084	-0.98388
FGFR1OP2	-0.27202	9.83493	-3.79947	0.001432	0.04172	-1.04083
RGS10	-0.27231	9.908404	-5.75555	2.33E-05	0.004725	2.87315
TOMM70A	-0.27275	9.464776	-3.94251	0.00105	0.035781	-0.7464
CACYBP	-0.2729	8.237526	-4.99806	0.00011	0.010267	1.400371
KRT12	-0.27342	9.487761	-3.90525	0.001138	0.036843	-0.8231
PRMT8	-0.27423	9.228719	-5.29854	5.89E-05	7.72E-03	1.993332
GLRX3	-0.27618	9.166401	-3.82816	0.001345	0.040084	-0.98179
CLCN4-2	-0.27869	8.232154	-5.26458	6.32E-05	7.94E-03	1.926869
ANK1	-0.27894	8.847987	-4.57955	0.000266	0.015965	0.558473
LOC10004	-0.28038	8.172219	-6.29037	8.13E-06	0.002931	3.865028
RASSF3	-0.28138	8.629859	-4.61587	0.000246	0.015119	0.632171
HAPLN4	-0.28255	9.550447	-7.14502	1.64E-06	1.11E-03	5.359115
SLC24A3	-0.28262	11.68753	-4.43476	0.000363	0.019438	0.263739
FGF12	-0.28466	9.618374	-5.92029	1.68E-05	4.21E-03	3.183181
CAMKK2	-0.28579	9.581449	-6.24587	8.86E-06	2.93E-03	3.784143
SORL1	-0.286	11.37858	-6.05671	1.28E-05	3.56E-03	3.436912
SEPT7	-0.2879	8.206913	-4.35909	0.000427	0.021197	0.109144
KPNB1	-0.29227	10.22433	-4.19348	0.00061	0.026657	-0.23027
GPM6B	-0.29731	8.30149	-4.50472	0.000312	0.017629	0.40633
GJB6	-0.30207	10.75613	-3.79013	0.001461	0.042234	-1.06004
JAK1	-0.303	8.622206	-4.2817	0.000504	0.023753	-0.0493

CDH22	-0.3059	7.891256	-5.02044	0.000105	1.03E-02	1.444899
YWHAE	-0.30668	8.61883	-3.86641	0.001238	0.038154	-0.90305
LOC10004(	-0.31017	8.855368	-5.052	9.82E-05	9.97E-03	1.507586
PPM1L	-0.31022	10.43274	-6.4373	6.13E-06	2.49E-03	4.129985
ALDH1A1	-0.31183	7.92979	-6.21909	9.33E-06	2.98E-03	3.735303
OSBP2	-0.31423	9.051612	-4.62867	0.00024	0.015119	0.658111
NPY	-0.31586	11.02052	-7.55872	7.82E-07	6.35E-04	6.041317
UBE2J1	-0.31594	8.766111	-5.46525	4.19E-05	0.006402	2.317532
CCNG2	-0.31753	9.25757	-5.53883	3.60E-05	0.005851	2.459448
RELL2	-0.31835	10.52928	-5.22105	6.91E-05	8.20E-03	1.841457
ARC	-0.32181	10.64455	-4.31834	0.000466	0.022351	0.025745
P2RY12	-0.32334	8.52596	-6.64262	4.15E-06	2.01E-03	4.494641
PTGDS	-0.32739	9.929905	-3.81919	0.001372	0.040553	-1.00024
PIK3CA	-0.32815	7.847088	-5.47689	4.09E-05	0.006344	2.340021
FAM107A	-0.34239	11.56403	-4.07815	0.000782	0.030812	-0.46727
LOC66883'	-0.34588	9.740829	-4.67688	0.000216	0.014736	0.755705
SLC7A10	-0.34672	8.636367	-8.76033	1.04E-07	1.56E-04	7.874378
SLC12A5	-0.34686	8.211898	-4.73845	0.00019	0.013854	0.880055
FOS	-0.3488	9.766477	-5.01544	0.000106	0.010267	1.434955
SLC25A4	-0.35011	7.746036	-3.68353	0.001842	0.047873	-1.27923
PVALB	-0.3536	11.5717	-5.42351	4.56E-05	0.006682	2.236689
RORA	-0.37532	8.983488	-7.16237	1.59E-06	1.11E-03	5.388273
A030009H(	-0.37707	9.0145	-4.10923	0.000732	0.029809	-0.40337
GLRX2	-0.37883	8.893965	-6.73253	3.51E-06	1.85E-03	4.652253
LOC10004ℇ	-0.39051	8.460216	-6.628	4.27E-06	0.002014	4.468888
HSD11B1	-0.41223	9.321022	-9.21458	5.05E-08	1.28E-04	8.5123
ID2	-0.42203	9.266153	-4.54884	0.000284	0.016575	0.496087
HUWE1	-0.42629	9.578061	-5.15393	7.94E-05	9.01E-03	1.7093
SCN1A	-0.42909	10.25208	-7.12948	1.69E-06	0.001112	5.332971
MAP2K1	-0.4326	9.565388	-5.90789	1.72E-05	0.004217	3.159982
NDUFB5	-0.43488	10.20906	-3.68588	0.001832	4.79E-02	-1.27441
GLUL	-0.43666	8.428036	-5.8015	2.12E-05	0.004588	2.960036
NDRG4	-0.4372	12.05689	-4.59213	0.000259	0.015721	0.58401
ATP5A1	-0.45175	10.89034	-3.9146	0.001115	3.66E-02	-0.80384
DUSP1	-0.46954	10.59118	-6.10097	1.17E-05	3.44E-03	3.518644
NEFM	-0.61056	10.04288	-4.56676	0.000274	0.016134	0.532502
NPAS4	-0.62709	8.183955	-14.7559	4.00E-11	4.22E-07	14.34863

DEGs in susceptible left mPFC

adj.P.Value	< 0.05
DEGup	234
DEGdown	237

Gene	logFC	AveExpr	t	P.Value	adj.P.Val	B
HBA-A1	1.10867	12.60395	16.25222	5.34E-11	5.63E-07	1.43E+01
HBB-B1	0.590954	8.799639	6.131656	1.84E-05	2.73E-03	3.1448868
WFS1	0.453204	8.924659	8.213079	5.79E-07	3.79E-04	6.426525
ALAS2	0.419706	7.583163	6.580325	8.30E-06	0.001835	3.9078195
MGP	0.404839	8.405792	8.620245	3.14E-07	3.21E-04	6.9940087
PFDN2	0.399404	11.04169	4.439209	0.000467	0.0221843	0.0107246
IGF2	0.395417	9.499371	7.013057	3.96E-06	1.13E-03	4.6144622
CPNE6	0.375195	11.49593	6.72518	6.46E-06	1.55E-03	4.1475604
KLF16	0.367043	8.781993	6.057422	2.10E-05	2.86E-03	3.0157068
CCDC85B	0.346065	10.26655	4.010052	0.0011152	0.0352762	-0.8328261
DLG4	0.340462	9.496925	5.713583	3.95E-05	0.0042512	2.4066542
FAM148C	0.334552	8.862439	5.53744	5.49E-05	0.0054007	2.0879668
TSC22D1	0.333725	9.097877	6.529165	9.08E-06	0.0019157	3.8223805
TMEM198	0.331863	9.405492	4.882984	0.0001933	0.0119274	0.8670723
LY6A	0.328519	9.565472	6.93404	4.53E-06	1.20E-03	4.4875679
CDKN1C	0.326102	8.447607	7.172867	3.03E-06	9.26E-04	4.868194
DACT2	0.324963	8.467647	4.732067	0.0002603	0.0143057	0.5780989
PROSAPIP1	0.323047	10.43257	6.320412	1.31E-05	0.0022561	3.4695961
CAR4	0.315639	8.937582	5.050089	0.0001394	0.0096805	1.1839982
LPL	0.3155	9.145933	7.218152	2.81E-06	0.0009102	4.9393883
MFGE8	0.310553	11.42435	5.980808	2.41E-05	0.0030698	2.8815185
LYZ	0.309988	8.308757	6.637315	7.52E-06	1.73E-03	4.0025236
SLC38A5	0.309208	8.130117	7.471316	1.86E-06	0.0006543	5.3316943
STK32C	0.306801	9.137387	5.321725	8.26E-05	0.0069188	1.6917524
CACNA1H	0.304843	9.677641	5.36814	7.56E-05	0.0064872	1.7775431
COTL1	0.301485	9.645081	6.07048	2.05E-05	0.0028596	3.0384914
NME2	0.30083	10.32974	6.112609	1.90E-05	2.79E-03	3.1118212
AHI1	0.300192	10.57708	4.981479	0.0001594	0.0102239	1.0542769
ECE1	0.298461	8.707794	5.705022	4.01E-05	4.27E-03	2.391269
SLC13A4	0.297359	7.609667	6.249755	1.49E-05	0.0024134	3.3486814
HAP1	0.296352	9.718132	5.785578	3.45E-05	4.01E-03	2.5356245
C1QTNF4	0.295476	10.68126	4.843151	0.000209	0.0124617	0.7910461
CPNE4	0.295428	10.03049	5.522666	5.64E-05	0.0054631	2.0610364
HPCA	0.290398	12.20839	5.650305	4.44E-05	0.0045507	2.2926797
ABHD14B	0.28895	9.130865	5.393313	7.21E-05	0.0064327	1.8239501
NRXN2	0.288636	10.91045	4.628631	0.0003196	0.0166164	0.3786448
NR2F6	0.288494	8.718306	6.136421	1.82E-05	2.73E-03	3.1531502

ANXA2	0.288297	8.2587	6.968733	4.27E-06	0.0011854	4.5434006
SLC8A2	0.286977	8.434484	6.789719	5.79E-06	1.47E-03	4.2533385
TIAM1	0.286631	10.38899	5.422717	6.82E-05	0.006148	1.8780468
IFITM3	0.28368	8.690011	5.874812	2.93E-05	0.0035858	2.6944218
GRASP	0.282582	9.44881	5.384685	7.33E-05	6.43E-03	1.8080533
FOSB	0.281432	8.376133	4.683923	0.0002864	0.0153395	0.4853984
GRP	0.280555	8.925961	3.911247	0.0013651	3.90E-02	-1.0283601
IGSF3	0.279533	8.42896	6.229192	1.54E-05	2.47E-03	3.3133502
SGK1	0.278326	11.44792	6.255713	1.47E-05	2.41E-03	3.358907
ANAPC5	0.277793	10.00353	4.136233	0.0008621	0.0306319	-0.5836722
PNCK	0.277513	9.398202	5.571474	5.15E-05	0.0051251	2.1498868
VPS25	0.27487	8.981466	6.479644	9.90E-06	0.0019864	3.7392951
ECHDC2	0.274129	8.450159	5.694837	4.09E-05	0.0042712	2.3729503
PDE2A	0.274091	9.818303	4.385343	0.0005205	0.0234715	-0.0944699
RILPL1	0.273343	9.735163	5.747448	3.71E-05	0.0040842	2.4674122
PPARGC1B	0.273311	9.531282	4.87587	0.000196	0.0119554	0.853509
ELTD1	0.266945	8.236378	5.718586	3.91E-05	0.0042512	2.4156402
DCN	0.266474	7.854345	6.31416	1.33E-05	0.0022561	3.4589274
GNB2	0.265266	10.99294	5.619258	4.71E-05	0.0047762	2.2365477
FUS	0.264729	8.26822	4.127318	0.0008779	0.030881	-0.6012495
DYNC1H1	0.264323	10.29217	3.847951	0.0015544	0.0413887	-1.1537614
FDPS	0.26422	10.83188	4.368009	0.000539	0.0236003	-0.1283694
LOC100047619	0.263621	10.48969	3.924676	0.0013281	0.0385467	-1.0017676
S100A11	0.26329	7.936864	6.028579	2.21E-05	2.88E-03	2.965293
ACTR1A	0.263104	10.82985	6.165118	1.73E-05	0.0026456	3.2028445
CACNB3	0.262536	8.971961	6.028577	2.21E-05	0.0028839	2.9652895
AI662250	0.261129	8.286352	4.690801	0.0002825	0.0152866	0.4986571
ITPKA	0.260705	11.18222	4.877195	0.0001955	0.0119554	0.8560357
HNRNPK	0.259455	8.520658	5.325329	8.20E-05	0.0069188	1.6984243
LOC100048301	0.258286	8.750746	5.140221	0.0001171	0.0086843	1.3535274
RAB3GAP2	0.258041	9.081676	4.371225	0.0005355	0.0236003	-0.1220777
FCHO1	0.257287	9.802169	4.130274	0.0008726	0.0307984	-0.5954207
PLD3	0.256961	10.79672	4.240735	0.0006972	0.0276592	-0.3779557
LOC100045782	0.25485	9.439505	4.180305	0.0007882	0.0298767	-0.4968359
PALMD	0.254095	8.656403	5.845888	3.09E-05	0.0036651	2.643078
DPP10	0.251812	10.53745	5.314477	8.38E-05	0.0069601	1.6783287
DYNLL2	0.251663	8.9233	5.118607	0.0001221	0.0088641	1.3129668
KCTD10	0.250777	9.086462	4.319116	0.0005949	0.0248134	-0.2241124
PSME2	0.250322	7.759331	5.34083	7.97E-05	6.78E-03	1.7271005
PTPRK	0.250319	8.592393	4.976834	0.0001608	0.0102239	1.0454739
SLC2A1	0.24952	9.947545	4.774086	0.0002395	0.0135892	0.6588075
RPS4Y2	0.247763	7.95517	4.172338	0.000801	0.0299766	-0.5125256
PDGFB	0.247513	9.157739	4.43728	0.0004688	0.0221843	0.0069616

FLT1	0.246893	10.00623	6.320386	1.31E-05	2.26E-03	3.4695523
TCEAL3	0.246051	8.924958	5.134806	0.0001183	0.0086843	1.3433712
ARL6IP1	0.245685	12.59459	4.848875	0.0002067	0.0123922	0.8019821
YRDC	0.245408	8.128247	4.550463	0.0003736	0.0189468	0.2272131
1300013J15RIK	0.243151	7.416783	5.447551	6.50E-05	0.0060197	1.9236425
LOC100045019	0.241891	11.22315	3.806117	0.0016938	0.0430495	-1.2366827
BAI2	0.241786	10.29107	4.888415	0.0001912	1.19E-02	0.8774248
STMN4	0.240105	9.660887	5.4559	6.40E-05	0.0060197	1.9389533
CXX1C	0.239158	10.42897	4.598759	0.0003392	0.0175494	0.3208434
KCTD4	0.238774	8.452961	4.801307	0.000227	0.0131611	0.7109898
HRMT1L2	0.237397	10.48449	4.335405	0.0005756	0.0245933	-0.1921952
TMEM121	0.23699	8.855934	5.613004	4.76E-05	0.0047864	2.2252238
ARHGEF15	0.236958	8.08675	5.307947	8.48E-05	6.99E-03	1.6662301
DGKG	0.235553	10.63397	4.396613	0.0005088	0.023207	-0.0724417
MID1	0.233447	7.855269	5.900405	2.80E-05	3.47E-03	2.7397491
D8ERTD82E	0.233143	7.809519	4.577768	0.0003538	0.0180771	0.2801769
EVC2	0.231504	8.157568	4.548278	0.0003752	0.0189468	0.2229731
AKAP8L	0.230903	10.04768	3.920483	0.0013395	0.038729	-1.0100698
PRMT2	0.228779	11.1476	4.833685	0.0002129	0.0126248	0.7729525
ERP29	0.228555	11.32563	4.03534	0.001059	0.0337638	-0.7828371
NBL1	0.228089	8.887872	3.848153	0.0015537	0.0413887	-1.1533627
COL6A1	0.227131	9.225453	5.014508	0.0001494	0.0099806	1.1167969
PKNOX2	0.227106	8.955686	4.989543	0.0001569	0.0102197	1.0695528
CACNG3	0.225866	8.812613	4.347013	0.0005623	0.0242196	-0.1694615
SNHG11	0.225844	13.09614	3.686952	0.0021642	0.0493936	-1.4729496
SNRPD1	0.224622	10.48105	4.325643	0.0005871	0.0246919	-0.2113211
FXYD5	0.224501	7.826416	5.247223	9.53E-05	0.0075597	1.5534432
LOC100046207	0.224191	11.82016	5.115811	0.0001227	0.0088641	1.3077153
MAPK1	0.222536	11.3214	5.2817	8.92E-05	0.0072698	1.6175401
MORN4	0.2221	8.545751	4.297823	0.0006211	0.0257019	-0.2658628
PRR7	0.221835	9.384036	4.401859	0.0005034	0.023199	-0.0621896
COL5A1	0.221131	8.052943	5.779622	3.49E-05	4.01E-03	2.5249838
FHL1	0.220897	10.33251	4.642925	0.0003107	0.0162302	0.4062721
OGN	0.219502	7.387425	4.451214	0.0004558	0.0220553	0.0341371
SF3B2	0.219315	11.17369	4.859247	0.0002025	0.0122113	0.8217895
EGFL7	0.219107	8.768657	5.082039	0.000131	0.0092349	1.2442084
RAMP3	0.218814	8.392446	5.152324	0.0001144	0.0085603	1.3762131
WBP7	0.218496	8.504333	3.813857	0.0016671	0.0427012	-1.2213384
UACA	0.218276	7.967921	5.226543	9.91E-05	0.0077505	1.5149219
ACSL5	0.218068	11.33073	4.100462	0.0009273	0.0313176	-0.6542286
BEX4	0.217383	9.108064	4.78983	0.0002322	0.0133728	0.6889984
G3BP1	0.216513	8.93059	3.806201	0.0016935	4.30E-02	-1.2365165
VWF	0.215803	7.58027	4.787684	0.0002332	0.0133728	0.6848849

RLBP1	0.215552	7.800433	4.439618	0.0004666	0.0221843	0.011523
RPS2	0.215535	12.2098	3.940707	0.0012852	0.0380789	-0.9700267
PPM1M	0.213725	8.633396	3.782055	0.0017797	0.0445044	-1.2843849
ARD1	0.212775	8.805313	4.576603	0.0003546	0.0180771	0.2779179
EXOSC2	0.212768	8.369204	4.423553	0.0004819	0.0224362	-0.0198243
5430437P03RIK	0.212472	9.352445	4.978953	0.0001602	0.0102239	1.0494903
PPM1G	0.21219	7.909943	4.329751	0.0005822	0.024676	-0.2032709
ATG16L1	0.211326	10.13511	5.659254	4.37E-05	0.0045192	2.3088324
C79267	0.211185	7.870549	4.516081	0.0004002	0.019922	0.1604268
2310067B10RIK	0.210724	8.900398	4.579273	0.0003527	0.0180771	0.2830926
GTPBP6	0.210354	8.148405	4.132072	0.0008694	0.0307888	-0.591876
LOC100045403	0.210303	12.55801	4.21567	0.0007335	0.028586	-0.4272372
MED6	0.207714	8.938034	4.464517	0.0004438	0.0215835	0.0600655
IMPDH2	0.20718	9.440955	4.686385	0.000285	0.0153395	0.4901459
UBQLN4	0.206136	9.872981	4.677907	0.0002898	0.0154455	0.4737988
ARL2BP	0.20535	8.993257	5.133991	0.0001185	0.0086843	1.3418414
PER2	0.205161	9.974818	3.927527	0.0013203	0.0384905	-0.9961223
SPAG5	0.20433	8.134456	4.241632	0.0006959	0.0276592	-0.3761911
ERGIC3	0.204214	9.800817	3.776102	0.0018016	4.46E-02	-1.2961878
VIP	0.204022	10.46507	3.710816	0.0020605	0.0477897	-1.4256352
LOC100046744	0.203418	8.63062	3.969435	0.0012118	0.0366412	-0.9131678
ORF61	0.201986	9.498599	4.048449	0.001031	0.033274	-0.756932
ACCN2	0.201765	9.589577	3.691213	0.0021453	4.91E-02	-1.464502
1600002K03RIK	0.201738	7.945538	4.211458	0.0007398	0.0287044	-0.4355217
CDH13	0.201347	8.439697	4.981093	0.0001595	0.0102239	1.0535458
SNAPC4	0.200158	9.161214	4.082347	0.0009621	0.0320824	-0.6899843
HDGF	0.199805	10.1173	4.167164	0.0008095	0.0300803	-0.5227162
9030607L17RIK	0.198879	8.32339	3.964173	0.0012249	0.0368195	-0.9235816
EG633640	0.19859	7.723142	4.07784	0.000971	0.0321229	-0.6988831
FKBP1A	0.198046	11.0073	3.787659	0.0017593	0.0440993	-1.2732749
SPARC	0.197772	8.820703	4.43092	0.0004748	0.0223256	-0.0054471
HSF1	0.197749	9.898833	3.96288	0.0012281	0.0368195	-0.9261392
CITED4	0.197618	7.933663	4.181225	0.0007867	0.0298767	-0.4950253
EIF2B5	0.197133	9.632521	3.880731	0.0014533	0.0401833	-1.0888087
STARD8	0.196558	8.988055	4.781667	0.000236	0.0134595	0.6733473
DGCR6	0.196363	8.539485	3.6838	0.0021783	4.94E-02	-1.4791996
ICAM5	0.195766	9.429693	3.840948	0.0015769	0.0413953	-1.1676419
PSME1	0.195076	8.940949	4.074839	0.000977	0.0322191	-0.7048071
MED30	0.193679	10.05659	4.147225	0.000843	0.0303393	-0.5620041
KCNQ2	0.193458	7.858577	4.347927	0.0005612	0.0242196	-0.167672
2410004L22RIK	0.193341	7.654476	3.842845	0.0015708	4.14E-02	-1.1638825
LOC100047651	0.193003	10.44043	4.536356	0.0003843	0.0192202	0.1998247
RAB35	0.192714	10.63935	3.682146	0.0021857	0.0494369	-1.4824773

CH25H	0.191093	7.330832	3.951737	0.0012565	0.0374563	-0.9481934
DUS4L	0.190795	8.419626	4.813677	0.0002215	0.0129149	0.7346763
BRP16	0.189409	8.724896	3.854166	0.0015347	0.0411052	-1.1414446
FBXO21	0.189249	10.62954	4.255054	0.0006772	0.0273142	-0.3498191
EIF6	0.187552	9.743348	3.90909	0.0013712	0.0389669	-1.0326323
TUT1	0.187351	8.435789	4.104504	0.0009196	0.0312596	-0.6462537
1600014C10RIK	0.18637	8.808375	4.376867	0.0005294	0.0236003	-0.1110431
METTL3	0.185593	9.368955	4.177643	0.0007925	0.0298767	-0.5020777
CLPTM1L	0.18539	10.78891	3.726173	0.0019964	0.0470804	-1.3951854
SFRS5	0.183728	10.07632	4.039858	0.0010493	0.033555	-0.7739076
CDC42BPB	0.182365	10.16177	3.880076	0.0014552	0.0401833	-1.0901066
JTV1	0.182021	8.567839	4.060615	0.0010058	0.0328598	-0.7328986
P140	0.181598	8.305353	3.780027	0.0017871	0.0445297	-1.2884073
EXDL2	0.180415	10.34811	3.924125	0.0013296	0.0385467	-1.0028587
TOMM34	0.179828	9.343561	4.371422	0.0005353	0.0236003	-0.1216932
CACNA1A	0.178846	7.746361	3.8856	0.0014388	4.01E-02	-1.0791624
GJB2	0.178833	7.55037	4.395226	0.0005102	0.023207	-0.0751508
WNT4	0.178766	7.771687	4.413055	0.0004922	0.0227814	-0.0403207
ALDH4A1	0.178645	9.816682	3.91401	0.0013574	0.0389669	-1.0228881
CENPT	0.178212	8.876303	3.844847	0.0015643	0.0413887	-1.1599148
SCN3B	0.177785	8.282374	4.160599	0.0008204	0.0303104	-0.5356494
MYST1	0.177414	8.634195	3.685869	0.002169	0.0493936	-1.4750959
UBE2L3	0.1774	11.23016	3.743374	0.001927	0.0462172	-1.36108
9030624J02RIK	0.177345	9.357109	4.044905	0.0010385	0.0334135	-0.7639353
GLP2R	0.177155	7.644971	3.810616	0.0016782	0.0428824	-1.2277642
SERPINF1	0.176394	8.022009	4.118185	0.0008944	0.0309516	-0.6192629
SPRN	0.175936	8.000731	3.825298	0.0016284	0.0421581	-1.198661
2610507B11RIK	0.175418	9.766797	4.008442	0.0011188	0.0352762	-0.8360096
DDIT4L	0.17504	8.776336	4.016431	0.0011007	0.0349879	-0.8202129
CBFA2T3H	0.174824	8.344349	3.790532	0.0017489	0.044049	-1.2675793
ATG9B	0.174356	7.994021	3.854319	0.0015342	0.0411052	-1.1411418
PDE1B	0.174308	10.87632	3.857475	0.0015243	0.0410354	-1.1348889
LOC100044829	0.173753	9.425801	3.863104	0.0015068	4.09E-02	-1.1237346
CYBA	0.173664	7.991633	3.754018	0.0018853	0.0455269	-1.339975
THOC4	0.172658	9.268186	3.876483	0.001466	4.02E-02	-1.0972236
RAC3	0.172125	8.019743	3.730609	0.0019783	0.0469987	-1.3863892
FBXL6	0.172028	8.625924	3.724534	0.0020031	0.0470804	-1.3984343
NNMT	0.171715	7.510133	3.869055	0.0014885	4.06E-02	-1.1119428
MBD3L2	0.170582	7.391808	3.747729	0.0019098	0.0460142	-1.3524445
ARSJ	0.169815	7.545497	4.148073	0.0008416	0.0303393	-0.5603324
PLD4	0.168975	7.462028	4.054818	0.0010177	0.0331483	-0.74435
CENTG1	0.168897	8.552411	4.119972	0.0008911	0.0309516	-0.6157381
NAPEPLD	0.168592	7.945383	4.264824	0.000664	0.0269491	-0.3306298

APBA3	0.168484	8.358946	3.830147	0.0016122	0.0420422	-1.1890481
BOK	0.16711	9.008271	3.729728	0.0019818	4.70E-02	-1.3881365
GABBR1	0.166092	8.463038	3.766133	0.0018389	0.0452348	-1.3159532
HDGFRP2	0.165088	10.8939	3.779374	0.0017895	0.0445297	-1.2897012
SIAT7F	0.164264	7.657323	3.70238	0.0020966	0.048308	-1.4423616
CIB2	0.163965	8.988893	3.986992	0.001169	0.0359229	-0.878433
PVRL3	0.163359	8.039421	4.114824	0.0009005	0.0309547	-0.6258922
2610110G12RIK	0.161943	8.67709	3.828135	0.0016189	4.21E-02	-1.1930373
MAPRE2	0.160067	12.07198	3.74468	0.0019218	0.0461982	-1.3584896
PNKP	0.159952	8.659156	3.915088	0.0013544	0.0389669	-1.0207543
RBBP7	0.159227	8.381073	3.985653	0.0011722	0.0359229	-0.8810806
ZBTB8B	0.159184	7.919013	3.759053	0.0018658	0.0453377	-1.3299911
EID2	0.157595	8.482277	4.238763	0.0007	0.027666	-0.3818309
ALOXE3	0.156637	7.650103	3.818682	0.0016507	0.042383	-1.2117749
SPATA7	0.15609	7.817523	4.158273	0.0008243	0.0303104	-0.5402321
ZFP810	0.154599	8.241748	3.876874	0.0014648	0.0401833	-1.0964507
TIMM50	0.153621	8.208673	3.685259	0.0021718	4.94E-02	-1.4763054
BMP1	0.15326	8.137301	3.985514	0.0011726	0.0359229	-0.881356
CORO1A	0.152183	10.06149	3.681304	0.0021895	4.94E-02	-1.4841468
TXNIP	0.150438	7.469305	3.77557	0.0018036	0.0445736	-1.2972436
LYPD1	0.149015	10.54637	3.680664	0.0021924	4.94E-02	-1.4854169
ZFP532	0.148163	8.803466	3.731392	0.0019751	4.70E-02	-1.3848362
2510009E07RIK	0.142052	9.425649	3.788069	0.0017578	4.41E-02	-1.2724622
THSD4	0.139483	7.619463	3.763974	0.0018471	0.0452731	-1.3202345
GSK3A	0.137415	7.501465	3.724974	0.0020013	4.71E-02	-1.397562
TMEM175	-0.15078	7.359128	-3.77833	0.0017933	0.0445297	-1.2917663
INSC	-0.15377	7.563494	-4.19286	0.0007683	0.0293773	-0.4721229
LOC545013	-0.1541	7.664849	-3.71374	0.0020481	0.047708	-1.419846
EPHA7	-0.15803	8.387731	-3.95999	0.0012354	0.0369329	-0.931854
ZFP161	-0.1599	7.626112	-3.84684	0.0015579	0.0413887	-1.1559652
PRSS23	-0.16006	7.439347	-3.86567	0.0014989	0.0407672	-1.1186525
PDE7B	-0.16021	7.287927	-3.70324	0.0020928	4.83E-02	-1.440649
PCMTD1	-0.16174	7.853967	-3.79058	0.0017488	0.044049	-1.2674911
CARHSP1	-0.1618	8.668882	-3.8869	0.001435	0.0400756	-1.0765854
PLXNB3	-0.16524	7.579603	-3.80373	0.0017021	0.0430762	-1.2414177
GORASP1	-0.16946	8.485574	-3.90155	0.0013925	3.93E-02	-1.0475718
PTPRT	-0.16993	9.194145	-3.83697	0.0015898	4.16E-02	-1.1755247
DEXI	-0.16997	8.692575	-3.67354	0.0022248	0.0498482	-1.4995489
NCAM2	-0.1737	8.052647	-4.19448	0.0007658	0.0293773	-0.468924
ABHD5	-0.17443	8.313656	-3.9854	0.0011728	0.0359229	-0.8815746
KLHL7	-0.17612	10.11401	-4.15649	0.0008273	0.0303135	-0.5437461
SLC24A2	-0.17709	7.750117	-4.12253	0.0008865	0.0309516	-0.6106896
NOL14	-0.17766	8.148153	-4.14261	0.000851	0.0303393	-0.5711085

DDX6	-0.17855	9.159642	-4.47611	0.0004336	0.0212444	0.0826576
ZCCHC17	-0.17996	9.246622	-3.98651	0.0011702	0.0359229	-0.8793787
HSD11B1	-0.18039	9.321022	-4.6483	0.0003074	0.0161374	0.4166542
SNAPC1	-0.18053	7.985758	-3.99138	0.0011586	0.0359229	-0.8697609
CDR2	-0.18231	8.471707	-3.73173	0.0019737	0.0469987	-1.384167
PALM2	-0.18298	8.030058	-4.47471	0.0004348	0.0212444	0.079915
ABCA5	-0.18539	7.511068	-3.99251	0.0011559	3.59E-02	-0.8675181
CDH2	-0.18603	7.553857	-4.06686	0.000993	0.0326457	-0.7205577
ATP13A2	-0.18684	8.678051	-4.16913	0.0008063	0.030066	-0.5188396
FAM108B	-0.18868	7.79897	-3.72132	0.0020164	0.0472876	-1.4048099
SNX7	-0.18876	8.082012	-3.76806	0.0018316	0.045161	-1.3121269
CRIPT	-0.1888	8.346967	-4.14343	0.0008496	0.0303393	-0.5694897
GRIN1	-0.18899	8.440663	-4.34779	0.0005614	0.0242196	-0.1679474
2010305A19RIK	-0.18921	7.845689	-4.15452	0.0008306	0.0303299	-0.5476289
ACSBG1	-0.19145	8.492413	-3.93762	0.0012933	0.0381245	-0.976148
SKIV2L	-0.19198	9.101456	-3.71804	0.0020301	0.0475023	-1.4113146
GRB14	-0.19202	8.169003	-4.75789	0.0002473	0.0138087	0.6277181
CAMKK2	-0.19289	9.581449	-3.99374	0.001153	0.0359229	-0.8650885
ZFP36L1	-0.19308	9.402585	-3.75635	0.0018763	0.045413	-1.3353521
LSM12	-0.19379	8.266302	-4.26495	0.0006638	0.0269491	-0.3303878
FIP1L1	-0.19568	7.723884	-4.37445	0.000532	0.0236003	-0.1157659
LITAF	-0.19712	9.402568	-3.93444	0.0013018	0.0382663	-0.9824332
MDGA2	-0.19745	10.00648	-3.82977	0.0016135	0.0420422	-1.1897883
LRRTM3	-0.19796	8.179193	-4.33257	0.0005789	0.024635	-0.1977576
ZFP457	-0.1983	7.854082	-3.98192	0.0011812	0.0359229	-0.8884656
OPN3	-0.19841	7.708122	-3.90823	0.0013736	0.0389669	-1.0343436
ELAVL2	-0.19852	10.69863	-4.90657	0.0001845	0.0115755	0.9120142
ANKRD35	-0.19902	7.595472	-3.69857	0.0021131	0.0485825	-1.4499206
SLC35F3	-0.19925	10.5571	-4.11756	0.0008955	0.0309516	-0.6204897
CCNL1	-0.20097	8.02895	-3.90923	0.0013708	0.0389669	-1.0323484
ZFP106	-0.20112	7.875586	-4.44919	0.0004577	0.0220553	0.0301819
OLA1	-0.20192	9.979465	-4.10954	0.0009103	0.0311464	-0.6363145
DCBLD1	-0.20334	8.228777	-4.21531	0.0007341	0.028586	-0.4279476
TLE4	-0.20583	10.51575	-4.73621	0.0002581	0.014263	0.5860707
DEGS1	-0.20641	11.37378	-4.10366	0.0009212	0.0312596	-0.6479206
6330503K22RIK	-0.20698	9.631736	-4.10861	0.000912	0.0311464	-0.6381608
RGS10	-0.20761	9.908404	-3.76233	0.0018533	4.53E-02	-1.3234896
HR	-0.20788	8.828072	-4.82941	0.0002147	0.0126604	0.7647834
KLHL5	-0.20837	7.651154	-4.06204	0.0010028	0.0328598	-0.7300883
PPP1R14A	-0.21035	7.848177	-3.98301	0.0011786	0.0359229	-0.8863143
CPLX2	-0.21041	10.7641	-3.85845	0.0015212	4.10E-02	-1.1329571
TCERG1	-0.2112	7.942802	-4.50636	0.0004081	0.0202186	0.1415186
PLLP	-0.21199	8.43167	-3.73187	0.0019731	0.0469987	-1.3838848

UBE3A	-0.21244	7.899536	-5.5065	5.82E-05	0.0055306	2.0315367
SEMA3C	-0.21299	7.329269	-3.7595	0.0018641	4.53E-02	-1.329111
EDNRB	-0.21305	9.175007	-5.38339	7.34E-05	0.0064327	1.8056751
CST3	-0.21376	14.00452	-3.91124	0.0013652	3.90E-02	-1.0283786
TMED9	-0.21404	11.00351	-4.0922	0.000943	0.0316926	-0.6705345
NCAPD2	-0.2166	8.053598	-3.93165	0.0013092	0.0382721	-0.9879491
ARSG	-0.21759	7.788665	-3.90053	0.0013955	3.93E-02	-1.0495911
PEBP1	-0.21763	9.613663	-3.93957	0.0012882	0.0380789	-0.9722879
PIGZ	-0.21795	8.331971	-4.67011	0.0002943	0.0156079	0.4587523
ANLN	-0.21797	8.280857	-4.20235	0.0007536	0.0291328	-0.4534331
PHTF2	-0.21948	8.132256	-4.2668	0.0006613	0.0269491	-0.3267428
RORA	-0.2206	8.983488	-3.71272	0.0020524	0.047708	-1.4218669
HSPA4	-0.221	7.682145	-3.93227	0.0013076	0.0382721	-0.9867218
FLRT2	-0.22145	9.381509	-4.24558	0.0006904	0.0275965	-0.3684394
CAT	-0.22329	7.897932	-4.39081	0.0005148	0.023314	-0.0837836
GPR17	-0.22329	8.459968	-4.2224	0.0007236	0.0284932	-0.4140063
EVL	-0.22348	10.05722	-4.28957	0.0006315	0.0260327	-0.2820556
PTGES3	-0.22472	9.397217	-3.8052	0.001697	0.0430495	-1.2385031
UCHL5	-0.22623	9.556969	-3.84285	0.0015707	0.0413887	-1.163869
ZFP292	-0.22676	8.929222	-4.19901	0.0007588	0.0292243	-0.4600163
GRIT	-0.22724	9.460596	-3.87391	0.0014738	0.0402915	-1.1023255
MAP3K1	-0.2288	7.624211	-4.42968	0.000476	0.0223256	-0.007861
A530082C11RIK	-0.23068	8.569516	-3.98225	0.0011804	0.0359229	-0.8878143
CADPS2	-0.23087	11.12074	-5.74628	3.72E-05	0.0040842	2.4653149
CNOT4	-0.2309	9.116484	-6.04804	2.14E-05	0.0028596	2.9993171
ITGB5	-0.23156	8.32545	-5.00435	0.0001524	0.0100529	1.0975822
CBLN2	-0.2327	8.078496	-4.75983	0.0002464	0.0138087	0.63145
RHOG	-0.23305	7.999174	-5.37686	7.44E-05	0.0064327	1.793625
SMEK2	-0.23563	9.418866	-4.54169	0.0003802	0.0191068	0.2101804
ZMAT3	-0.23569	10.58692	-4.11647	0.0008975	0.0309516	-0.6226369
9130422G05RIK	-0.23865	8.9559	-5.11274	0.0001235	0.0088641	1.3019502
TJP1	-0.24025	8.397151	-3.70847	0.0020705	0.047916	-1.4302941
PTN	-0.24175	11.83842	-4.72597	0.0002634	0.0144044	0.5663759
ENPP2	-0.24341	11.01117	-3.71685	0.002035	4.75E-02	-1.413664
RELL1	-0.24467	8.501901	-3.88545	0.0014393	0.0400756	-1.0794592
RAB6B	-0.24646	12.47246	-4.48288	0.0004278	0.0210942	0.0958291
TYKI	-0.24703	8.792128	-4.32265	0.0005906	0.0247346	-0.2171855
SCD1	-0.24734	10.50984	-4.82125	0.0002182	0.0127942	0.7491711
1700023B02RIK	-0.24811	8.225738	-5.22648	9.91E-05	0.0077505	1.5147955
CLCN4-2	-0.24913	8.232154	-5.03658	0.0001431	0.0097458	1.1585032
LOC100047749	-0.25012	8.076691	-4.3647	0.0005426	0.0236603	-0.1348485
PDCD4	-0.25095	8.358616	-3.81938	0.0016483	0.042383	-1.2103841
SLC7A10	-0.25199	8.636367	-4.9924	0.000156	0.0102197	1.0749593

ZNRF1	-0.25362	8.790423	-5.03962	0.0001423	0.0097458	1.1642334
SLC7A14	-0.25388	9.001413	-4.42283	0.0004826	0.0224362	-0.0212428
GPM6B	-0.2546	8.30149	-3.67438	0.002221	4.98E-02	-1.4978764
S100B	-0.2547	7.766454	-5.21758	0.0001009	0.0078262	1.4982129
ZBTB38	-0.2594	7.687606	-6.40703	1.12E-05	0.0020465	3.616783
OSBPL6	-0.25954	9.332504	-5.69973	4.05E-05	0.0042712	2.38176
LOC329575	-0.26211	9.201205	-3.88912	0.0014285	0.0400756	-1.0721931
SEMA3E	-0.26234	8.285762	-4.44514	0.0004614	0.0221343	0.0222942
FOS	-0.26245	9.766477	-5.42842	6.74E-05	0.0061343	1.8885193
CTSB	-0.2632	13.04462	-4.00802	0.0011198	0.0352762	-0.8368531
PGM2L1	-0.26616	10.91507	-3.87754	0.0014628	0.0401833	-1.0951398
PBRM1	-0.26704	9.111144	-4.17748	0.0007927	0.0298767	-0.5023952
FGF1	-0.26717	9.163044	-4.1744	0.0007977	0.0299573	-0.5084582
ALDOA	-0.26736	12.53948	-4.12113	0.000889	0.0309516	-0.6134632
SEPP1	-0.27065	8.726684	-4.04193	0.0010449	0.0335151	-0.7698191
INA	-0.27312	10.56001	-3.98679	0.0011695	0.0359229	-0.878831
SLC44A1	-0.27381	8.392265	-7.14815	3.16E-06	9.27E-04	4.8292086
ABCD2	-0.27395	8.007285	-6.46451	1.02E-05	0.0019864	3.7138338
ZFP91-CNTF	-0.27524	8.334652	-5.08117	0.0001313	0.0092349	1.2425765
SULF1	-0.27566	8.768357	-5.98343	2.40E-05	0.0030698	2.8861218
NR1D2	-0.27592	8.714713	-3.82294	0.0016363	0.0422195	-1.203336
BUD31	-0.27659	9.647988	-4.39879	0.0005065	0.023207	-0.0681896
PCBP2	-0.27727	10.78411	-3.72701	0.0019929	0.0470804	-1.3935164
PPP1R3C	-0.27743	10.51775	-3.88358	0.0014448	0.0401236	-1.0831615
PPM1L	-0.27804	10.43274	-5.87014	2.96E-05	0.0035858	2.6861407
PTEN	-0.27819	8.296044	-4.05039	0.001027	0.033244	-0.7530953
ATP6V0A2	-0.2784	8.836953	-5.0625	0.0001361	0.0095124	1.2074032
LOC100045304	-0.27843	8.674278	-5.00876	0.0001511	1.00E-02	1.1059258
R3HDM1	-0.27915	8.718493	-5.7642	3.59E-05	0.0040609	2.4974131
NFIC	-0.27981	8.22565	-7.16545	3.07E-06	9.26E-04	4.8565061
NPY	-0.28003	11.02052	-5.50928	5.79E-05	0.0055306	2.0366025
ASH1L	-0.28142	7.909971	-6.57728	8.35E-06	1.83E-03	3.9027389
2610044O15Rik	-0.28231	8.103242	-5.81658	3.26E-05	0.0038255	2.5909303
IMPAD1	-0.28263	8.443051	-3.90446	0.0013842	0.0391632	-1.0417931
ANKRD17	-0.28295	8.146667	-4.14885	0.0008402	0.0303393	-0.5588101
KPNB1	-0.28297	10.22433	-3.75827	0.0018688	0.0453377	-1.3315376
DYNLT3	-0.28541	11.61523	-3.94478	0.0012745	0.0378868	-0.9619694
LOC100046320	-0.28636	9.399475	-5.03019	0.0001449	0.0098046	1.1464296
NPAS4	-0.28719	8.183955	-6.05277	2.12E-05	0.0028596	3.0075776
KCNK13	-0.28941	8.208999	-4.25337	0.0006795	0.0273142	-0.3531207
TXNL4	-0.28956	9.157446	-4.0996	0.0009289	0.0313176	-0.655922
SKP1A	-0.29175	11.02576	-3.97003	0.0012103	0.0366412	-0.9119966
EVI2A	-0.29342	8.523316	-5.37719	7.43E-05	0.0064327	1.7942371

NETO1	-0.29425	8.955297	-6.0717	2.05E-05	2.86E-03	3.0406205
C1QL3	-0.29453	8.877394	-4.08154	0.0009637	0.0320824	-0.6915718
PMP22	-0.29505	8.672352	-5.19039	0.0001063	0.0080687	1.4474467
KIF5A	-0.29787	11.55376	-4.90424	0.0001854	0.0115755	0.9075643
LOC100041703	-0.29917	7.832738	-3.76254	0.0018525	0.0452731	-1.3230743
FAM134A	-0.29967	10.51222	-3.96796	0.0012154	0.0366472	-0.9160946
KCNK2	-0.29971	8.964489	-4.15825	0.0008243	0.0303104	-0.5402781
ANK3	-0.30099	8.759347	-4.70168	0.0002764	0.0150371	0.519626
TCFE3	-0.302	7.804171	-7.48071	1.83E-06	6.54E-04	5.3460652
MYO6	-0.30452	10.00877	-5.91217	2.74E-05	3.44E-03	2.7605502
FAM134B	-0.30595	11.08409	-4.36908	0.0005378	0.0236003	-0.1262777
NDUFB4	-0.30745	9.025854	-3.82484	0.0016299	4.22E-02	-1.1995697
TBC1D19	-0.31029	8.628206	-4.30807	0.0006083	0.025274	-0.2457728
ADAMTS4	-0.31185	7.889195	-4.94136	0.0001724	0.0108925	0.9781618
PHF14	-0.31209	7.979481	-5.01883	0.0001482	0.0099599	1.1249725
MYL4	-0.31315	10.89938	-7.49564	1.79E-06	6.54E-04	5.3688787
ROCK2	-0.31328	8.327694	-6.42065	1.10E-05	0.0020465	3.6398252
TOMM70A	-0.31654	9.464776	-3.84224	0.0015727	0.0413887	-1.1650828
GPM6A	-0.31727	10.32941	-5.25406	9.40E-05	7.52E-03	1.5661735
ATAD1	-0.3178	9.886556	-5.20974	0.0001024	0.0078871	1.4835797
MOG	-0.32021	8.901772	-7.21139	2.85E-06	0.0009102	4.9287788
HSF2	-0.32468	7.94058	-4.74618	0.0002531	0.0140579	0.6052271
LASS2	-0.32604	9.522891	-6.40694	1.12E-05	2.05E-03	3.6166375
SEL1L	-0.32655	8.589124	-4.07786	0.000971	0.0321229	-0.6988466
VCL	-0.32701	9.080144	-5.44995	6.47E-05	0.0060197	1.9280503
ADSSL1	-0.32921	9.236767	-8.18694	6.03E-07	3.79E-04	6.3892979
TBL1X	-0.33069	9.068043	-4.66605	0.0002967	0.0156554	0.4509336
LOC100044177	-0.33478	8.509613	-6.46461	1.02E-05	1.99E-03	3.7139938
MDH1	-0.33486	8.886961	-5.19485	0.0001054	0.0080577	1.4557696
IAP	-0.33699	11.19885	-5.25438	9.40E-05	0.0075176	1.5667574
TMEM184C	-0.3401	8.991811	-6.10303	1.93E-05	0.0027964	3.0951731
OLIG1	-0.34093	12.08359	-6.78277	5.85E-06	1.47E-03	4.241981
ADAM9	-0.34608	8.772647	-6.04701	2.14E-05	0.0028596	2.9975165
GLTP	-0.34848	9.439177	-6.5152	9.30E-06	1.92E-03	3.7989901
NDUFB2	-0.34906	8.82696	-5.27951	8.96E-05	0.0072698	1.6134787
ZEB2	-0.35005	9.562137	-7.84422	1.03E-06	5.70E-04	5.8921339
BZW1	-0.35249	9.998774	-4.25252	0.0006807	0.0273142	-0.354793
MAP2K1	-0.35345	9.565388	-3.67939	0.0021982	4.95E-02	-1.4879367
DLD	-0.35378	9.035528	-4.14341	0.0008496	0.0303393	-0.569521
GSTM5	-0.3546	11.46692	-5.09206	0.0001285	0.0091642	1.2630662
LOC100048530	-0.35492	8.460216	-4.76355	0.0002446	0.013801	0.6385839
CNP	-0.36463	9.184679	-6.21189	1.59E-05	2.51E-03	3.2835701
XLR4A	-0.36541	8.818481	-7.42552	2.00E-06	6.82E-04	5.2614378

DST	-0.36941	9.687143	-3.69137	0.0021446	0.0491095	-1.4641943
FUT9	-0.37216	7.660745	-7.61997	1.46E-06	6.18E-04	5.5575823
TRIM59	-0.37302	7.856267	-7.48695	1.81E-06	6.54E-04	5.3556014
SC4MOL	-0.37357	10.7044	-7.64124	1.42E-06	6.18E-04	5.5896201
EDG2	-0.37368	8.274282	-5.76073	3.62E-05	0.0040609	2.4911981
HTR1F	-0.37436	8.201666	-7.64301	1.41E-06	6.18E-04	5.5922929
SST	-0.3792	12.73845	-4.37035	0.0005364	0.0236003	-0.1237931
SELK	-0.38075	10.29669	-7.51984	1.72E-06	6.54E-04	5.4057862
SEPT7	-0.38371	8.206913	-5.15488	0.0001138	0.0085603	1.3810082
SCN1A	-0.38571	10.25208	-5.85211	3.06E-05	0.0036646	2.6541327
TMEM178	-0.39797	11.20272	-6.1983	1.63E-05	0.0025293	3.2601447
ARC	-0.40271	10.64455	-7.89118	9.53E-07	0.0005587	5.9612627
LOC668837	-0.40361	9.740829	-6.56374	8.55E-06	1.84E-03	3.8801685
STMN1	-0.4068	10.84226	-3.85967	0.0015174	0.0410354	-1.1305346
MOBP	-0.40846	9.052363	-9.32387	1.14E-07	1.72E-04	7.9216387
GLUL	-0.40974	8.428036	-4.21784	0.0007303	0.028586	-0.4229752
PLEKHB1	-0.41037	11.22758	-4.33829	0.0005723	0.0245498	-0.1865439
IL33	-0.41584	8.923349	-7.62493	1.45E-06	0.000618	5.5650589
ID2	-0.41817	9.266153	-4.26804	0.0006596	0.0269491	-0.3243119
ENPP6	-0.41951	8.235505	-8.50862	3.71E-07	3.21E-04	6.8407252
DUSP1	-0.4298	10.59118	-7.62685	1.45E-06	6.18E-04	5.5679467
FA2H	-0.43244	10.11226	-8.32953	4.86E-07	3.66E-04	6.5911891
OPALIN	-0.43297	8.913391	-5.53366	5.53E-05	0.0054007	2.081085
MEGF9	-0.43593	9.484822	-8.46627	3.95E-07	3.21E-04	6.7821216
QDPR	-0.43797	9.731883	-6.93256	4.54E-06	0.0011971	4.4851774
KCNA1	-0.44247	9.983153	-8.17899	6.10E-07	3.79E-04	6.3779502
TRF	-0.44854	10.38794	-6.29694	1.37E-05	2.29E-03	3.429505
2510003E04RIK	-0.46587	9.671275	-4.05067	0.0010264	0.033244	-0.7525349
NDRG1	-0.46847	8.982476	-4.86861	0.0001988	0.0120575	0.8396589
A030009H04RII	-0.46939	9.0145	-4.08391	0.0009591	0.0320824	-0.6868901
PLP1	-0.48567	13.35939	-6.41866	1.10E-05	2.05E-03	3.6364611
TSPAN2	-0.48869	9.588803	-9.03785	1.71E-07	2.01E-04	7.5524577
PGRMC1	-0.48921	10.40578	-5.04385	0.0001411	0.0097346	1.1722221
PRR18	-0.49336	10.29879	-6.72115	6.51E-06	1.55E-03	4.1409339
CLDN11	-0.49684	12.82258	-9.21003	1.34E-07	0.0001771	7.7759682
MBP	-0.49737	12.4868	-11.6569	5.75E-09	2.02E-05	10.56913
SCN4B	-0.49789	9.653842	-8.54697	3.51E-07	3.21E-04	6.8935855
MAG	-0.50234	11.07011	-6.33115	1.29E-05	2.26E-03	3.4879113
MAP1LC3B	-0.51673	9.973792	-4.14895	0.0008401	0.0303393	-0.5585962
UGT8A	-0.52601	9.291783	-11.2144	9.75E-09	2.57E-05	10.112992
GJC2	-0.52934	8.416071	-7.6904	1.31E-06	6.18E-04	5.6634441
JAK1	-0.54443	8.622206	-6.71242	6.61E-06	1.55E-03	4.1265677
SPARCL1	-0.55035	10.72649	-4.32547	0.0005873	0.0246919	-0.2116571

MAL	-0.64099	10.16366	-11.8869	4.39E-09	2.02E-05	10.798476
BCAS1	-0.69346	12.16648	-9.93673	4.96E-08	1.05E-04	8.6778423
NEFM	-0.69662	10.04288	-5.42959	6.73E-05	0.0061343	1.8906705
LPGAT1	-0.70101	8.435119	-9.42478	9.94E-08	1.72E-04	8.0493752

DEGs in resilient left mPFC

adj.P.Value	< 0.05
DEGup	207
DEGdown	187

Gene	logFC	AveExpr	t	P.Value	adj.P.Val	B
GBP2	0.72731639	7.66056502	16.2623439	5.29E-11	5.58E-07	1.39E+01
CTGF	0.71655944	10.5568533	13.7035409	6.06E-10	3.20E-06	1.21E+01
HBA-A1	0.65360581	12.603948	8.74652926	2.61E-07	3.45E-04	7.07E+00
COL6A1	0.54431168	9.22545339	10.9700686	1.32E-08	3.47E-05	9.66E+00
RGS9	0.53004388	8.98084078	8.37394695	4.54E-07	4.79E-04	6.57374009
SLA	0.5244151	8.89092299	7.99390871	8.12E-07	6.59E-04	6.05E+00
IFITM3	0.4813154	8.69001105	9.09923747	1.57E-07	0.00033148	7.5228103
CMTM5	0.4248932	9.72152111	5.20476514	0.00010338	1.11E-02	1.50576151
RPRM	0.41761493	11.0341216	4.33000093	0.00058194	0.02624452	-0.1479201
IIGP2	0.41663311	7.65321297	8.11615961	6.72E-07	5.91E-04	6.22E+00
AHI1	0.41227444	10.5770826	6.24532814	1.50E-05	4.53E-03	3.34E+00
SEZ6	0.4043393	9.33471543	4.52843242	0.00039044	0.02239287	0.23430928
IGTP	0.39408004	7.65657693	8.56641087	3.41E-07	3.99E-04	6.83E+00
PTPRU	0.38374163	8.32085233	4.63031509	0.00031857	0.0195456	0.42914405
NTSR1	0.37083086	8.08729358	5.39818881	7.14E-05	9.30E-03	1.85873991
COL5A1	0.36728953	8.05294259	8.76330913	2.55E-07	3.45E-04	7.09E+00
PEA15	0.36580947	11.0826732	4.25962229	0.00067099	2.79E-02	-0.2842629
GRP	0.3632124	8.92596053	4.62239903	0.00032363	0.01963387	0.41404314
SAMD14	0.35427398	9.19400368	5.46122427	6.34E-05	0.00868457	1.97264348
LYZ	0.35174883	8.30875705	6.8752732	5.00E-06	2.42E-03	4.3699962
COTL1	0.35053458	9.64508114	6.44313398	1.06E-05	4.29E-03	3.67E+00
FXYD6	0.34990777	12.8022387	6.02435375	2.23E-05	5.23E-03	2.96427826
6330503C03I	0.34432699	10.5691509	3.96184233	0.00123074	0.03971879	-0.8646061
SPSB1	0.34340759	9.26422672	5.37862353	7.41E-05	9.49E-03	1.82327143
ADRA2A	0.34288886	8.96545121	5.87674504	2.92E-05	5.60E-03	2.70896103
CASC3	0.34170751	10.0445341	4.43596882	0.00047002	0.02455524	0.05662849
PLOD3	0.34132678	9.05859914	5.31193765	8.42E-05	1.02E-02	1.70197863
DPYSL5	0.33934644	7.95106553	6.39517027	1.15E-05	4.37E-03	3.59E+00
LY6A	0.33164142	9.56547165	6.39004267	1.16E-05	4.37E-03	3.5822898
ERP29	0.32847867	11.3256306	5.29427462	8.71E-05	1.03E-02	1.67E+00
HAP1	0.32591353	9.71813153	5.80832411	3.31E-05	0.00602717	2.58948742
PRMT2	0.32489834	11.1476029	6.26640409	1.44E-05	4.53E-03	3.38E+00
FAM171A2	0.32398854	10.388839	5.35986453	7.68E-05	0.00965003	1.78921407
HIST1H2AO	0.3238989	10.2416106	6.2569516	1.47E-05	4.53E-03	3.36E+00
PDE2A	0.32251091	9.81830252	4.71045772	0.00027166	0.01837739	0.58165689
CDKN1C	0.32085638	8.44760745	6.44256475	1.06E-05	4.29E-03	3.67E+00
RAMP3	0.31961971	8.3924463	6.87024045	5.04E-06	2.42E-03	4.36E+00

RAI14	0.31790188	8.74760376	6.18530823	1.67E-05	0.00452793	3.23885429
CDH13	0.31748061	8.43969677	7.16979108	3.05E-06	2.01E-03	4.83E+00
SYT5	0.31424091	10.3186048	5.72344439	3.88E-05	0.00668212	2.44029267
S100A8	0.31320713	7.55956522	7.01641868	3.94E-06	2.21E-03	4.59E+00
FAM148C	0.31249882	8.86243934	4.7217542	0.00026565	0.0182038	0.60309902
CBLN1	0.31203372	8.54147333	5.58480611	5.02E-05	7.72E-03	2.19429658
ARL2	0.31202058	9.19495199	5.01860138	0.00014824	1.35E-02	1.16128428
GNG7	0.31109509	10.2406808	4.17226017	0.00080117	3.03E-02	-0.4539944
KCTD10	0.31055034	9.086462	4.88256447	0.00019343	0.01588044	0.90678009
MFGE8	0.30838402	11.4243492	5.42156782	6.83E-05	0.00912512	1.9010511
MAP3K3	0.308255	8.55540534	5.53565999	5.51E-05	7.75E-03	2.10641445
RLBP1	0.30478053	7.80043288	5.73047099	3.83E-05	6.68E-03	2.4526845
DLG4	0.30397391	9.49692496	4.65678191	0.00030222	0.01899364	0.47958585
XRCC1	0.29993652	8.46453332	6.3434893	1.26E-05	4.53E-03	3.50477363
FEZF2	0.29985094	9.72472105	4.1798653	0.00078888	3.03E-02	-0.4391989
METTL3	0.2994042	9.36895529	6.15230395	1.77E-05	4.67E-03	3.18E+00
FDPS	0.29729403	10.8318761	4.48655791	0.00042461	0.02383487	0.15393914
SEMA4A	0.29689014	10.2925298	5.0254238	0.00014629	0.01345885	1.17398752
SCAMP2	0.29686507	8.77843666	4.71210726	0.00027078	0.01837739	0.5847888
STATIP1	0.29603642	10.5913701	4.31141254	0.00060422	0.02679127	-0.1838947
NRBP2	0.2921339	10.2750614	4.24911893	0.00068543	0.02823293	-0.3046419
5430437P03I	0.29176821	9.35244513	6.24141925	1.51E-05	4.53E-03	3.33362983
DDR1	0.28913609	8.7460174	5.11866308	0.00012207	1.22E-02	1.35E+00
ASAH3L	0.28720016	7.96930731	4.2967786	0.00062237	0.0268076	-0.2122347
LOC1000475	0.28573189	9.87488445	4.64019596	0.00031236	1.94E-02	0.44798403
DGKG	0.28495	10.633969	4.85520124	0.00020412	0.01619583	0.85531698
ACTR1A	0.28491105	10.8298495	6.09442808	1.96E-05	5.06E-03	3.08431274
HNRNPK	0.28472949	8.5206578	5.33490194	8.06E-05	1.00E-02	1.74381762
ICAM5	0.2845324	9.42969298	5.09615536	0.00012751	1.23E-02	1.30533871
ZER1	0.28418472	12.3539291	4.19360008	0.00076717	0.0298743	-0.4124876
DBI	0.28305936	11.2517493	4.67304709	0.00029261	0.01882851	0.51054848
GH	0.28248431	7.23640633	6.30058769	1.36E-05	4.53E-03	3.43E+00
PADI6	0.28131167	7.45104214	5.24940419	9.49E-05	1.05E-02	1.58767932
PDE1B	0.280005	10.8763249	5.65666533	4.39E-05	0.00712685	2.32215671
ANLN	0.27997655	8.2808568	4.92747592	0.00017712	1.52E-02	0.99105296
PPP1R1B	0.2799175	8.97316049	5.18847032	0.00010667	1.11E-02	1.47579171
ALDH4A1	0.27872472	9.81668194	5.57462351	5.12E-05	7.72E-03	2.17611719
ZMYM3	0.27796566	10.5506796	4.78169359	0.00023594	1.72E-02	0.71663573
LMAN2L	0.27722551	9.21099533	5.07153165	0.00013374	0.01271534	1.25968445
MED30	0.2766862	10.0565877	5.40844691	7.00E-05	9.24E-03	1.88E+00
FANCD2	0.27579874	8.1102285	5.89584134	2.82E-05	5.60E-03	2.74E+00
HSF1	0.27571457	9.89883323	5.04389686	0.00014112	0.01329695	1.20835447
CACNA1H	0.2745926	9.67764093	4.41414228	0.00049112	0.02478703	0.01457406

HIST1H2AH	0.27445863	7.87564113	5.88640142	2.87E-05	5.60E-03	2.72576536
RPRML	0.27207069	10.6658441	4.50295216	0.00041088	0.0234381	0.18542432
C4B	0.27164169	7.49978945	5.57412841	5.12E-05	0.00772225	2.17523288
AMIGO2	0.2695825	8.55137098	3.98928478	0.00116354	0.03833166	-0.8109363
1700027N10	0.26530942	8.01199439	5.03903271	0.00014246	1.33E-02	1.19930951
TXNDC5	0.26514533	8.78931254	3.94988925	0.00126123	4.02E-02	-0.8879919
LOR	0.26500952	9.26875914	5.9917608	2.37E-05	5.31E-03	2.91E+00
MGP	0.26417314	8.40579191	5.13494365	0.00011828	1.20E-02	1.3770953
RASL10A	0.2635879	9.58820196	4.08002884	0.0009667	3.45E-02	-6.34E-01
SLC9A3R2	0.26328908	8.86538615	3.82414574	0.00163224	4.63E-02	-1.1342712
LOC1000485	0.26237269	7.3121917	5.92774699	2.66E-05	5.60E-03	2.79755554
LOC1000483	0.2617378	8.75074611	4.75505943	0.0002487	0.01769331	0.6662349
ARPC5	0.26006417	11.1296861	4.42445711	0.00048103	0.02472631	0.03445339
DENND2A	0.25974376	8.29597454	4.53842029	0.00038271	2.22E-02	0.25345461
LASS5	0.25890909	9.45211093	4.53545883	0.00038499	0.02220081	0.24777891
BEX4	0.25862425	9.10806379	5.20204089	0.00010392	1.11E-02	1.50075348
OASL2	0.25804734	7.46063322	4.91998815	0.00017974	1.52E-02	0.97701964
ENSMUSG00	0.25748333	7.88298176	5.54802011	5.38E-05	7.75E-03	2.12854969
WSCD1	0.2566169	8.90817941	4.14180257	0.00085238	0.03161488	-0.5132842
SEMA5A	0.25644608	9.76898032	4.69316012	0.00028115	0.01842835	0.54879708
CRMP1	0.25628835	9.35140786	3.84201726	0.00157343	0.04536723	-1.0992439
AGPAT4	0.25606981	9.41941948	4.22091109	0.00072579	0.02926014	-0.3594099
ZFYVE21	0.25581775	9.79064244	4.57812595	0.00035351	0.02119653	0.32946915
9030624J02F	0.25530015	9.35710945	5.31555444	8.36E-05	0.0102095	1.70857297
ADAM15	0.25477133	9.51510423	4.49065282	0.00042114	0.02376633	0.16180576
CXX1C	0.25390587	10.4289732	4.45696004	0.00045061	0.0245493	0.09703403
WBP5	0.2538873	10.7542197	3.8583403	0.00152159	4.49E-02	-1.0672574
ST6GALNAC5	0.25166391	9.7821164	4.39040978	0.00051516	0.02482434	-0.031199
HDGF	0.25147816	10.1173034	4.78789929	0.00023307	0.01708026	0.72836751
IGF2	0.24960236	9.49937059	4.04119845	0.00104642	3.62E-02	-7.09E-01
DRCTNNB1A	0.24683752	8.36220791	5.27352695	9.06E-05	0.01039103	1.63183471
TSC22D1	0.24666292	9.09787686	4.40536996	0.00049987	0.02482434	-0.0023396
IDE	0.24563783	9.57247495	3.86758014	0.00149301	4.43E-02	-1.05E+00
AI894139	0.2456207	8.09544081	4.39141607	0.00051412	0.02482434	-0.0292572
TRIM41	0.24558318	9.41411425	4.44586106	0.00046077	0.0245493	0.07567472
FA2H	0.24544011	10.1122559	4.31568435	0.00059902	0.02667296	-0.175625
SAMD9L	0.24458682	8.21459994	4.26843352	0.00065912	0.02760212	-0.2671731
2610208M17	0.2436618	9.26142735	3.90486782	0.0013831	4.22E-02	-0.9761189
NME2	0.24255588	10.329742	4.49910574	0.00041406	0.02349252	0.17803952
MAS1	0.24145756	8.79785389	4.41796645	0.00048736	0.02472631	0.0219453
MORN4	0.24093044	8.54575067	4.25599878	0.00067594	2.80E-02	-0.2912925
RFX1	0.24085165	8.27313652	3.95815295	0.00124007	3.99E-02	-0.8718236
DRD1A	0.23741997	8.89829189	3.97605026	0.00119547	3.89E-02	-0.8368156

ARL6IP1	0.23714749	12.5945866	4.27257859	0.00065361	0.02760212	-0.2591354
CYBA	0.23700991	7.9916332	4.67695874	0.00029034	0.01879736	0.51799058
DDAH1	0.23680293	11.2813149	3.79668103	0.00172698	4.77E-02	-1.188112
RYR3	0.23640937	8.07447006	4.70549726	0.00027435	0.01842835	0.57223693
JOSD2	0.23559235	9.28461091	3.98657474	0.00117001	0.03834512	-0.8162351
G3BP1	0.23520052	8.93058992	3.77446689	0.00180765	0.04929229	-1.231668
RPS26	0.23514436	11.8522756	3.88407379	0.00144334	0.04327156	-1.0168433
MIDN	0.23358263	10.1683838	4.33784966	0.00057279	0.02614914	-0.1327384
HIST1H2AK	0.23328321	7.99415354	4.36045588	0.00054724	0.02571531	-0.0890386
LOC1000482	0.23296584	9.89374789	4.3037171	0.00061369	2.68E-02	-0.1987957
D12ERTD647	0.23192765	9.96936057	5.27980866	8.95E-05	1.04E-02	1.64331997
SPRED1	0.2319093	11.6326393	4.35731866	0.00055071	0.02571531	-0.0951007
ARD1	0.23126028	8.80531302	4.54081213	0.00038088	0.0221909	0.25803802
WFS1	0.23021222	8.92465889	3.80846964	0.00168565	0.04705997	-1.1650005
NR2F6	0.22953673	8.71830637	4.45697771	0.00045059	0.0245493	0.09706804
ECE1	0.22891649	8.70779445	3.99443971	0.00115134	0.0382079	-0.8008581
GM347	0.22710281	9.25337727	3.83045775	0.00161122	4.60E-02	-1.12E+00
ARHGEF15	0.22645484	8.08675003	4.63069484	0.00031833	0.0195456	0.42986831
HIST2H2AC	0.22639309	10.2980401	4.06026944	0.00100646	3.54E-02	-0.6722591
ARD1A	0.22555531	9.6175107	4.01197044	0.00111081	3.80E-02	-0.7665926
ACVR2B	0.22517906	8.79906189	4.9220095	0.00017903	0.01517738	0.98080865
BAT1A	0.22491457	10.9653133	4.58269953	0.0003503	0.02112387	0.33821507
GAB1	0.22481247	9.00369265	4.80563762	0.00022505	0.01684358	0.76187742
MED6	0.22423229	8.93803399	4.39963273	0.00050568	0.02482434	-0.013405
INTS4	0.22326807	9.30587442	3.91209951	0.00136275	4.19E-02	-0.9619589
FIGF	0.22220842	7.85536692	4.41929907	0.00048605	0.02472631	0.02451367
ITGB4	0.22197859	7.81661978	4.55483844	0.00037035	0.02195661	0.28490501
ETS2	0.22177192	10.8329305	4.21089503	0.00074069	2.96E-02	-0.3788699
H3F3A	0.22090498	10.167176	3.85044786	0.00154644	0.04510091	-1.0827226
VPS37C	0.21897736	7.66261498	3.91067489	0.00136673	0.04192766	-0.9647482
LOC1000462	0.21806354	11.8201642	4.54244498	0.00037964	0.0221909	0.26116666
THOC4	0.21797631	9.26818632	4.46754232	0.00044113	0.02445159	0.11738874
ARPC4	0.2151017	9.41185601	4.12101962	0.00088922	0.03247026	-0.5537724
PSME2	0.21486211	7.75933121	4.18483846	0.00078095	3.01E-02	-0.4295258
GNB2	0.21413954	10.9929425	4.14097764	0.00085381	0.03161488	-0.5148908
THSD4	0.21381142	7.619463	5.2670094	9.17E-05	1.04E-02	1.62E+00
DDX27	0.21358971	8.34350716	3.80402704	0.00170111	4.71E-02	-1.17E+00
MOBP	0.21091835	9.05236343	4.39515786	0.00051026	0.02482434	-0.0220376
NPM3-PS1	0.21029489	9.32619546	3.85354186	0.00153665	0.04504507	-1.0766598
CELSR3	0.20875692	10.1994232	3.94349839	0.00127785	0.04049581	-0.9004975
IFIT3	0.2086082	7.82613328	4.69725417	0.00027887	0.01842835	0.55657736
CEP120	0.20776642	10.0179769	3.89783512	0.00140318	0.04255109	-0.9898908
SMPD3	0.20699083	9.09406722	3.92548104	0.00132589	0.04103248	-0.9357614

VPS25	0.20635303	8.98146554	4.44061665	0.00046565	0.02455524	0.06557836
ITFG3	0.20490279	7.7505897	4.30700976	0.00060962	0.0268076	-0.1924194
CSRP2	0.2039182	7.8909085	3.81410125	0.00166626	4.68E-02	-1.1539605
GBP3	0.20378764	7.72570631	4.19749031	0.00076113	2.98E-02	-0.4049242
CACNB3	0.20373972	8.97196061	4.27081993	0.00065595	2.76E-02	-0.2625455
ILK	0.20313357	9.88498295	4.28947125	0.00063164	0.02709631	-0.2263921
HPCAL4	0.20253292	13.0954874	4.18419107	0.00078198	0.03011748	-0.4307849
LMO3	0.20112918	8.37342408	3.95701794	0.00124296	0.03986912	-0.8740442
APBA3	0.19963427	8.35894646	4.14288152	0.00085051	0.03161488	-0.5111829
NUMA1	0.1984612	7.73571399	3.87619776	0.00146685	4.37E-02	-1.0322713
UBE2L3	0.19803463	11.2301617	3.81469845	0.00166422	4.68E-02	-1.15E+00
SF3B2	0.19654197	11.1736923	3.97525306	0.00119743	0.03888136	-0.8383747
ATG16L1	0.19380728	10.1351094	4.73790001	0.00025729	0.01793268	0.63372152
SDK1	0.19378217	7.8105597	3.92029517	0.00134005	4.13E-02	-0.9459133
UPF3A	0.19262864	7.99018957	3.83745855	0.00158822	0.0454215	-1.1081781
CBFA2T3H	0.19250204	8.34434919	3.81015917	0.00167981	0.04702132	-1.1616884
SGK1	0.19211816	11.4479198	3.94185587	0.00128215	0.0405106	-0.9037118
D430041B17	0.19081431	9.12323543	3.7808291	0.00178416	4.88E-02	-1.2191928
FBXO21	0.18998335	10.6295379	3.89938568	0.00139873	4.25E-02	-0.9868543
CD74	0.18962902	7.23198446	4.06780335	0.00099111	0.03521608	-0.6575545
MAPK1	0.18713731	11.321404	4.05455438	0.00101827	0.03558222	-0.6834155
RBBP7	0.18646251	8.38107256	4.26072321	0.0006695	0.02787798	-0.2821273
S100A11	0.18417631	7.93686446	3.84966637	0.00154892	4.51E-02	-1.084254
SLC2A3	0.18320907	11.3323739	4.17051557	0.00080402	0.03030283	-0.457389
SCRIB	0.18125157	8.24136484	4.1230547	0.00088554	0.03244824	-0.5498067
SLC9A1	0.18057772	9.01189405	4.39829426	0.00050705	0.02482434	-0.0159869
ANXA2	0.18054056	8.25869956	3.9837975	0.00117668	0.03844418	-0.8216655
CTXN1	0.17825284	10.2981216	3.77223069	0.00181598	4.94E-02	-1.24E+00
IRF1	0.17772877	7.6511468	4.16153415	0.00081884	3.06E-02	-0.4748676
JOSD1	0.17738489	10.9458483	3.94848681	0.00126486	0.04020499	-0.890736
COX4NB	0.17709742	7.93215351	3.90471743	0.00138352	4.22E-02	-0.9764134
FXYD5	0.17637022	7.82641592	3.76310374	0.00185037	0.04994117	-1.2539504
TPST1	0.17621868	9.57839146	4.11094411	0.00090766	3.28E-02	-0.5734099
1600014C10I	0.17533789	8.80837478	3.75899847	0.00186606	5.00E-02	-1.2620009
MUM1	0.17519167	9.76793586	3.88670774	0.00143557	0.04316106	-1.0116841
ST5	0.1749312	8.93081243	3.99866623	0.00114143	0.0382079	-0.7925957
UACA	0.17410982	7.96792062	3.8057603	0.00169506	4.71E-02	-1.170312
ALOXE3	0.17248358	7.65010303	3.83863355	0.0015844	0.0454215	-1.1058753
LOC1000480	0.17199985	8.84783397	3.82899514	0.00161607	4.60E-02	-1.12E+00
DUS4L	0.16522382	8.41962591	3.80532819	0.00169656	4.71E-02	-1.1711591
5133400G04	0.15824766	7.86018462	3.75996113	0.00186237	5.00E-02	-1.2601131
CDH8	-0.1547624	8.21145266	-3.8480973	0.00155391	0.04510091	-1.0873289
D11WSU47E	-0.1697061	8.09100643	-3.8555044	0.00153047	4.50E-02	-1.0728142

9130213B05I	-0.1721488	9.48811239	-4.0007711	0.00113653	3.82E-02	-0.7884812
PALM2	-0.1742581	8.03005812	-3.8901694	0.00142541	0.04297819	-1.0049039
ZFP161	-0.1756964	7.62611164	-3.8586357	0.00152067	4.49E-02	-1.0666786
6330406I15R	-0.1792514	8.81559798	-3.9985133	0.00114179	0.0382079	-0.7928946
FIP1L1	-0.1846194	7.72388406	-3.767689	0.00183301	0.04970301	-1.2449589
NFIC	-0.1850112	8.22564978	-4.3249625	0.00058789	0.02638208	-0.1576685
1110032E23I	-0.1897337	7.82790267	-3.8475547	0.00155565	4.51E-02	-1.09E+00
FKBP7	-0.18996	7.41293178	-4.1732535	0.00079955	3.03E-02	-0.4520618
ZBTB38	-0.1910065	7.68760601	-4.306686	0.00061002	0.0268076	-0.1930463
DIAP3	-0.1927974	7.76091449	-3.933452	0.00130441	0.04067682	-0.9201592
SLMAP	-0.1963016	8.9465056	-3.8404783	0.00157841	4.54E-02	-1.10E+00
ZCCHC17	-0.1982558	9.24662207	-4.0090809	0.00111739	3.80E-02	-0.7722397
PLXDC1	-0.1987521	8.81422529	-3.9326019	0.00130668	4.07E-02	-9.22E-01
DDIT4L	-0.199271	8.77633627	-4.1740494	0.00079826	0.03030283	-0.4505132
KLHL7	-0.1995562	10.1140051	-4.2991862	0.00061935	0.0268076	-0.207571
SLC39A12	-0.2008061	9.77355053	-4.0114508	0.00111199	3.80E-02	-0.7676082
ASH1L	-0.2013968	7.909971	-4.2968205	0.00062232	0.0268076	-0.2121536
VAMP1	-0.2025936	7.90248061	-4.4435318	0.00046293	0.0245493	0.07119077
HR	-0.2060784	8.82807208	-4.3704457	0.00053632	0.02549451	-0.0697405
PREI4	-0.2064273	9.13433251	-4.0620283	0.00100286	0.03536492	-0.6688258
TMEM136	-0.2084623	8.29878741	-3.7936607	0.00173773	4.77E-02	-1.19E+00
TMEM218	-0.2089637	8.53966465	-4.0871128	0.00095283	0.03408558	-0.61988
CCNC	-0.2092644	8.18052726	-4.3232016	0.00058999	2.64E-02	-0.1610759
CRIPT	-0.2096734	8.34696681	-4.2005422	0.00075643	0.0298302	-0.3989913
5730472N09	-0.2115982	10.1393743	-4.8074731	0.00022424	1.68E-02	0.76534275
LOC1000441	-0.2151606	8.50961321	-3.7927929	0.00174083	4.77E-02	-1.1957351
GRAMD4	-0.2157626	8.01404579	-4.0138305	0.00110659	3.80E-02	-0.7629577
CLCN4-2	-0.2163337	8.23215412	-3.9925031	0.00115591	3.82E-02	-0.8046441
LOC545013	-0.2167426	7.66484905	-4.7684319	0.00024221	0.01750681	0.69155002
MRPS18C	-0.2168136	10.4520367	-3.7666743	0.00183684	0.04970301	-1.2469487
4931406C07I	-0.2178242	8.32967179	-4.0008093	0.00113644	0.0382079	-0.7884065
ITGB5	-0.2178824	8.32544989	-4.2985707	0.00062012	2.68E-02	-0.2087633
NRN1	-0.2182582	12.5845376	-4.1108798	0.00090778	3.28E-02	-0.5735353
CCNE1	-0.2189054	7.45288275	-4.7527879	0.00024982	0.01769331	0.66193274
MAGOHB	-0.2191514	7.76901814	-4.0097804	0.00111579	0.03803807	-0.7708725
NAT5	-0.219714	9.48699766	-3.8713643	0.00148146	0.04403915	-1.0417402
UBE3A	-0.2202259	7.89953635	-5.2109248	0.00010216	1.11E-02	1.51708115
GABRG2	-0.2217854	9.85545614	-4.9340077	0.00017487	0.01517738	1.00328895
SNX7	-0.2219048	8.08201153	-4.0437518	0.00104098	0.03613636	-0.7045076
2610044O15	-0.2219845	8.10324211	-4.1752049	0.00079639	0.03030283	-0.4482651
RG9MTD1	-0.2222002	8.48755627	-4.6621049	0.00029904	0.01899364	0.48972185
STARD8	-0.2254364	8.98805521	-5.0063512	0.00015182	0.01369401	1.13845986
RAB10	-0.2281382	8.5178054	-4.3360527	0.00057487	0.02614914	-0.1362138

OSBPL6	-0.2315367	9.33250385	-4.641749	0.0003114	0.01939031	0.45094431
HAPLN4	-0.2329205	9.55044711	-4.2759869	0.00064912	0.02760212	-0.2525274
SPAG9	-0.2337413	9.15645284	-4.269354	0.0006579	0.02760212	-0.265388
ZFP367	-0.233966	7.81244926	-4.2295991	0.00071311	0.02916823	-0.3425355
2310047O13	-0.2357469	7.66377073	-4.4105579	0.00049468	0.02478703	0.00766389
NTNG1	-0.2381477	8.58345708	-4.5584897	0.00036765	0.02192008	0.29189573
SELK	-0.2409211	10.2966912	-4.3436195	0.00056615	0.02597654	-0.121581
CAMK2G	-0.241596	10.0675616	-4.2204658	0.00072644	0.02926014	-0.3602748
EGR2	-0.2422923	8.30567915	-5.6566048	4.39E-05	7.13E-03	2.32E+00
GRIN1	-0.2429456	8.44066288	-5.1019948	0.00012607	0.0123188	1.31615397
ARCN1	-0.2464699	7.92460342	-4.465941	0.00044255	0.02445159	0.11430923
GADD45G	-0.248077	8.98114632	-4.3544577	0.0005539	0.02575027	-0.1006297
SKIV2L	-0.2486019	9.10145632	-4.3950167	0.0005104	0.02482434	-0.0223099
SLITRK3	-0.2486429	8.17547314	-5.0825063	0.00013093	1.26E-02	1.28004193
GRIA3	-0.2486916	8.8106862	-4.4264518	0.00047911	0.02472631	0.03829661
PDHB	-0.2489454	9.28756032	-4.1981444	0.00076012	0.0298302	-0.4036525
SCAMP3	-0.2495979	7.88973311	-4.221437	0.00072501	0.02926014	-0.3583882
PTN	-0.2511145	11.8384157	-4.4813704	0.00042906	0.02395679	0.14397133
ARNTL	-0.2515586	8.82486104	-3.9367048	0.00129575	4.07E-02	-0.9137927
SLC24A3	-0.2523753	11.6875339	-3.8448497	0.00156431	0.04522778	-1.093693
STMN3	-0.2537282	11.1287635	-3.8195019	0.00164788	0.04649757	-1.143374
EIF3A	-0.2540049	9.90564264	-3.9882671	0.00116597	0.03833166	-0.812926
BMPER	-0.2550192	8.15340116	-4.8297755	0.00021459	0.01652986	0.8074189
1110008P14I	-0.2552825	12.1151011	-4.4546481	0.0004527	0.0245493	0.09258578
5730469M10	-0.2562916	9.36508929	-4.3582525	0.00054967	0.02571531	-0.0932961
GPM6A	-0.2576491	10.3294071	-3.8949656	0.00141146	0.04267953	-0.9955105
LOC1000463	-0.2579211	9.39947452	-4.1358872	0.0008627	0.03177215	-0.5248057
ABHD12	-0.2581881	9.44510909	-4.9935359	0.00015567	1.39E-02	1.11E+00
TYROBP	-0.2600813	7.65398959	-4.7314549	0.00026059	0.01797398	0.62150108
TBC1D23	-0.2607308	8.74207067	-4.1351042	0.00086408	0.03177215	-0.5263308
EPDR1	-0.2610968	11.3240985	-4.8450655	0.00020823	1.63E-02	0.83623185
SEMA3E	-0.261845	8.28576234	-4.0502552	0.00102725	0.0357774	-0.691809
LRRTM3	-0.2628526	8.17919309	-5.2516259	9.45E-05	1.05E-02	1.59174934
AGXT2L1	-0.2649321	7.87128236	-4.9046558	0.00018522	0.01551316	0.94826317
TSPYL4	-0.2687866	7.58542523	-3.9549128	0.00124833	3.99E-02	-0.8781628
LOC1000446	-0.2703619	12.0216155	-4.1961473	0.00076321	0.0298302	-0.4075351
ZEB2	-0.2713207	9.56213709	-5.5502938	5.36E-05	7.75E-03	2.13261906
KRT12	-0.2730527	9.48776137	-5.2781817	8.98E-05	1.04E-02	1.64034588
ROCK2	-0.2731807	8.32769383	-5.110957	0.0001239	0.01221994	1.3327442
CRHBP	-0.2741212	11.0537709	-3.9402409	0.0012864	4.05E-02	-0.9068723
TCFE3	-0.274433	7.80417078	-6.2054947	1.61E-05	0.00452793	3.27300722
DPM2	-0.2764758	9.4302523	-4.9611136	0.00016584	1.46E-02	1.05401106
VCL	-0.2777903	9.080144	-4.2263295	0.00071785	0.02924899	-0.3488853

DUSP6	-0.2778275	10.1133136	-4.9590286	0.00016652	0.01464378	1.05011267
S100B	-0.2782768	7.76645434	-5.203773	0.00010357	1.11E-02	1.50E+00
ABCD2	-0.2797512	8.00728519	-6.0263116	2.22E-05	5.23E-03	2.97E+00
PTGES3	-0.2801972	9.39721669	-4.33115	0.00058059	0.02624452	-0.1456971
PDE7B	-0.2802485	7.28792668	-5.913423	2.73E-05	5.60E-03	2.77E+00
GJB6	-0.2803689	10.7561347	-4.6565323	0.00030237	0.01899364	0.47911045
ARC	-0.2808685	10.6445515	-5.0240931	0.00014667	1.35E-02	1.17151024
BUD31	-0.2824499	9.64798818	-4.1005395	0.00092711	3.34E-02	-0.5936946
4930452B06I	-0.2849159	7.91131012	-4.3503645	0.0005585	0.02585	-0.108541
ARMCX5	-0.2862827	9.46208071	-4.7430891	0.00025466	0.01791594	0.64355709
NPY	-0.2884273	11.0205184	-5.1801391	0.0001084	1.12E-02	1.46E+00
LOC1000467	-0.2941196	8.17221881	-4.3798307	0.00052627	0.02512981	-0.0516183
ATP2B2	-0.294262	9.75118756	-5.6736801	4.25E-05	7.12E-03	2.3523198
PIK3R4	-0.2981058	8.10399151	-3.7927327	0.00174104	4.77E-02	-1.1958532
ANKRD17	-0.2985232	8.14666661	-3.9958338	0.00114806	0.0382079	-0.7981326
BC048546	-0.3012398	9.075915	-7.0114567	3.97E-06	2.21E-03	4.58E+00
NECAB1	-0.3017738	8.96109015	-4.5388569	0.00038238	0.0221909	0.25429137
GOPC	-0.3037702	7.6724985	-3.8798433	0.00145592	4.35E-02	-1.02513
CBLN2	-0.3061529	8.07849599	-5.7165931	3.93E-05	6.68E-03	2.43E+00
RGS4	-0.3100428	12.4503255	-6.5278693	9.10E-06	4.00E-03	3.80979268
EMB	-0.31112	9.94787478	-4.2136983	0.00073649	0.02955201	-0.3734228
DGKB	-0.3121388	9.55066216	-5.1294122	0.00011955	0.01201577	1.36687435
ALDH1A1	-0.3133309	7.92979	-4.191165	0.00077097	2.99E-02	-0.4172225
TMEM32	-0.3137053	8.91632432	-4.801363	0.00022696	0.0168666	0.75380551
KCNA1	-0.3137182	9.98315271	-5.2937828	8.71E-05	1.03E-02	1.66885031
C1GALT1C1	-0.3148493	9.03711403	-4.9198831	0.00017978	0.01517738	0.97682279
ELAVL4	-0.3153791	9.19286119	-4.6573854	0.00030186	0.01899364	0.48073514
TJP1	-0.3153984	8.39715144	-4.4443447	0.00046218	0.0245493	0.07275579
TBC1D19	-0.3156915	8.62820636	-4.0011287	0.0011357	0.0382079	-0.7877823
GLRX2	-0.3162618	8.89396468	-4.0976545	0.00093257	0.03347432	-0.5993203
OSBP2	-0.3166724	9.0516124	-4.3657071	0.00054147	0.02562392	-0.0788934
NDUFB2	-0.3173516	8.82695992	-4.3816734	0.00052432	0.02512981	-0.0480609
FGF12	-0.3210532	9.61837415	-4.7582117	0.00024715	0.01769331	0.67220425
EVL	-0.3212908	10.0572227	-5.6296568	4.62E-05	0.00738117	2.2741892
ATAD1	-0.3213514	9.88655588	-4.8089294	0.00022359	0.01684358	0.76809201
KCNAB3	-0.3227959	7.99494232	-4.8657798	0.00019991	1.60E-02	0.87522302
FEZ2	-0.3252718	9.51004821	-3.9261198	0.00132415	4.10E-02	-0.9345111
KCNV1	-0.3267426	9.38054021	-4.6831369	0.0002868	0.01868271	0.52974153
A530082C11	-0.3276608	8.56951605	-5.1636508	0.0001119	1.15E-02	1.430075
COCH	-0.3278033	7.74142953	-5.7858544	3.45E-05	6.18E-03	2.55009738
HSBP1	-0.3281009	11.7542797	-4.4096334	0.0004956	0.02478703	0.00588149
MRPS15	-0.3293276	8.49057374	-4.4526901	0.00045449	0.0245493	0.0888181
SLC7A10	-0.3294488	8.63636723	-5.9583629	2.52E-05	5.53E-03	2.85054707

RASSF3	-0.3306074	8.62985941	-4.2727716	0.00065336	2.76E-02	-0.2587612
FUT9	-0.3308313	7.660745	-6.1835946	1.67E-05	4.53E-03	3.23595216
MEGF9	-0.3309573	9.48482227	-5.8676046	2.97E-05	5.60E-03	2.69304151
PRMT8	-0.3311151	9.22871897	-4.6947818	0.00028025	0.01842835	0.55187918
2410129H14	-0.3320526	9.22034873	-4.4203997	0.00048498	0.02472631	0.02663478
RSPRY1	-0.3350399	7.64991849	-4.8844154	0.00019273	0.01588044	0.91025803
UBE2J1	-0.3358892	8.76611148	-4.3939438	0.00051151	0.02482434	-0.0243799
P2RY12	-0.3396272	8.52596033	-4.8701813	0.00019819	0.01596588	0.88350145
PPM1L	-0.3404825	10.4327393	-6.5622083	8.57E-06	0.00393131	3.86601009
OPN3	-0.34423	7.70812162	-6.1897985	1.65E-05	4.53E-03	3.25E+00
HSF2	-0.3463818	7.9405795	-4.6222493	0.00032373	0.01963387	0.41375753
ADAM9	-0.347451	8.77264722	-5.5420006	5.44E-05	0.00774783	2.1177724
NFU1	-0.3480531	8.01526895	-4.4361235	0.00046988	0.02455524	0.05692644
MRPL20	-0.3532792	10.2726085	-4.2475859	0.00068756	0.02823293	-0.307617
UCHL5	-0.354402	9.55696917	-5.4956046	5.94E-05	8.25E-03	2.03452871
TAF13	-0.3568287	9.24950745	-6.0788256	2.02E-05	0.00507774	3.05765198
GSTM5	-0.3580918	11.4669217	-4.6941393	0.0002806	0.01842835	0.55065813
PVALB	-0.3602049	11.5716975	-4.7950565	0.0002298	0.01695839	0.74189277
TOMM70A	-0.3606	9.46477595	-3.9956403	0.00114852	3.82E-02	-0.7985109
COX7B	-0.3606128	9.23467748	-4.1138217	0.00090235	3.28E-02	-0.5678009
LOC1000417	-0.3627094	7.83273804	-4.1641902	0.00081442	0.03058586	-0.4696981
CAMKK2	-0.3662128	9.58144867	-6.9217957	4.62E-06	2.42E-03	4.44E+00
TMEM184C	-0.3682301	8.99181088	-6.0321262	2.20E-05	5.23E-03	2.97762955
MTMR12	-0.3689504	9.87382074	-5.8116403	3.29E-05	0.00602717	2.59529441
HSD11B1	-0.3729202	9.32102244	-8.7721235	2.52E-07	3.45E-04	7.10E+00
R3HDM2	-0.3745464	10.6348487	-4.4235691	0.00048189	0.02472631	0.03274241
NSG1	-0.3752331	8.78126024	-3.9725502	0.00120407	3.90E-02	-0.843661
DLD	-0.3796641	9.03552759	-4.0591807	0.0010087	3.54E-02	-0.6743843
CDH22	-0.383858	7.89125551	-4.8116525	0.0002224	0.01684358	0.77323214
SEPT7	-0.3979808	8.20691273	-4.8807435	0.00019412	1.59E-02	0.90335804
KPNB1	-0.3990332	10.2243301	-4.838045	0.00021113	0.01638243	0.82300559
SMPD4	-0.401809	8.12718492	-3.8220307	0.00163934	4.64E-02	-1.14E+00
RORA	-0.4037972	8.98348808	-6.2037278	1.61E-05	4.53E-03	3.27002027
PDZRN3	-0.4054322	9.21724556	-7.018465	3.93E-06	2.21E-03	4.59539133
FOS	-0.4137438	9.76647721	-7.8119955	1.08E-06	8.14E-04	5.79E+00
SLC12A5	-0.4141472	8.21189792	-4.0657394	0.00099529	3.52E-02	-0.6615827
GPM6B	-0.4221178	8.30149019	-5.5611753	5.25E-05	0.00774783	2.15208443
SCN1A	-0.4257585	10.2520814	-5.8968591	2.81E-05	5.60E-03	2.74E+00
LOC1000485	-0.4387017	8.46021607	-5.3749709	7.46E-05	0.00948938	1.81664389
DUSP1	-0.4567765	10.5911814	-7.3992655	2.09E-06	1.47E-03	5.17928046
CYFIP2	-0.4615227	10.3946373	-4.2063774	0.00074752	0.02976812	-0.3876493
JAK1	-0.4617828	8.62220612	-5.1973966	0.00010485	0.01106516	1.49221358
FOXG1	-0.4819767	10.7068772	-4.3459428	0.0005635	0.02596788	-0.1170889

LPGAT1	-0.4892176	8.4351187	-6.0042563	2.31E-05	5.31E-03	2.92971234
A030009H04	-0.4954271	9.0145	-3.9348605	0.00130065	4.07E-02	-9.17E-01
GLUL	-0.5039776	8.42803552	-4.7359275	0.00025829	0.01793268	0.62998192
PGRMC1	-0.5176179	10.4057758	-4.8717274	0.00019759	1.60E-02	0.88640907
NDUFB4	-0.5197932	9.02585354	-5.903097	2.78E-05	5.60E-03	2.75E+00
SETD3	-0.5198583	9.38776346	-4.1998547	0.00075748	0.0298302	-0.4003278
LOC668837	-0.5489508	9.74082912	-8.1495662	6.39E-07	0.00059115	6.26514536
NDRG4	-0.5698097	12.0568899	-4.4509499	0.00045608	0.0245493	0.08546931
NPAS4	-0.5894663	8.18395513	-11.34106	8.37E-09	2.94E-05	1.00E+01
ID2	-0.6029888	9.26615316	-5.6181355	4.72E-05	7.43E-03	2.25E+00
NDUFB5	-0.6160481	10.2090556	-3.7612511	0.00185744	5.00E-02	-1.26E+00
NEFM	-0.7623033	10.0428796	-5.4238201	6.80E-05	0.00912512	1.90512316
REXO1	-0.8749936	13.4383849	-4.8478587	0.00020709	0.01627727	0.84149261

DEGs in susceptible right mPFC

adj.P.Value	< 0.05	
DEGup		240
DEGdown		240

Gene	logFC	AveExpr	t	P.Value	adj.P.Val	B
CTGF	0.888018	10.55685	18.60342	7.56E-12	7.97E-08	1.61E+01
HBA-A1	0.829271	12.60395	12.15645	3.22E-09	8.51E-06	1.12E+01
RPRM	0.794649	11.03412	9.025641	1.74E-07	1.08E-04	7.59E+00
SLA	0.655077	8.890923	10.93873	1.37E-08	1.44E-05	9.93E+00
SEMA5A	0.570781	9.76898	11.44272	7.41E-09	1.12E-05	1.05E+01
ZFPM2	0.529271	10.0211	10.29118	3.11E-08	2.98E-05	9.18E+00
SULF1	0.514473	8.768357	11.16702	1.03E-08	1.21E-05	1.02E+01
ADRA2A	0.4749	8.965451	8.916121	2.04E-07	1.17E-04	7.44E+00
PRSS35	0.472352	8.502893	7.737558	1.21E-06	3.97E-04	5.754815699
HBB-B1	0.452332	8.799639	4.693332	0.000281	0.014105	0.461461152
SGK1	0.429066	11.44792	9.64377	7.36E-08	5.23E-05	8.393298661
MOG	0.427892	8.901772	9.636345	7.43E-08	5.23E-05	8.38E+00
SPSB1	0.427801	9.264227	7.339956	2.30E-06	6.08E-04	5.140670155
FDPS	0.423435	10.83188	7.000109	4.05E-06	9.00E-04	4.60E+00
GLRA2	0.421272	9.669872	6.291631	1.38E-05	2.08E-03	3.408364162
PRR18	0.41105	10.29879	5.599801	4.88E-05	0.004906	2.175347923
IGSF21	0.40723	9.680149	5.20922	0.000102	0.007671	1.449413529
PPP1R1B	0.401553	8.97316	8.15347	6.35E-07	2.68E-04	6.37E+00
FUS	0.398458	8.26822	6.212252	1.59E-05	0.002207	3.270464157
NOS1AP	0.393522	8.453826	5.112501	0.000124	0.008629	1.266576692
RAI14	0.391675	8.747604	8.348042	4.72E-07	2.17E-04	6.653421325
GABARAPL1	0.391325	11.87212	5.639875	4.53E-05	0.00464	2.248653797
GARNL3	0.366239	11.71781	5.649559	4.45E-05	4.60E-03	2.266333405
STK32C	0.363203	9.137387	6.300064	1.36E-05	2.08E-03	3.422959169
NTSR1	0.360795	8.087294	5.753391	3.67E-05	3.99E-03	2.455073798
FOXN3	0.354447	8.552932	5.794209	3.40E-05	0.003858	2.52884974
DDAH1	0.353899	11.28131	6.215663	1.58E-05	2.21E-03	3.276409062
ANLN	0.352362	8.280857	6.79333	5.75E-06	1.17E-03	4.258026273
FA2H	0.350201	10.11226	6.745467	6.24E-06	0.001203	4.17860405
CPLX2	0.350024	10.7641	6.418799	1.10E-05	1.85E-03	3.627316305
FAM20B	0.341027	9.404528	6.198557	1.63E-05	2.23E-03	3.25E+00
TLE4	0.336238	10.51575	7.736988	1.22E-06	3.97E-04	5.753950923
STMN4	0.335442	9.660887	7.622246	1.46E-06	4.40E-04	5.58E+00
ENSMUSG000	0.331249	7.882982	7.818698	1.07E-06	3.89E-04	5.877312389
COL6A1	0.327605	9.225453	7.23274	2.75E-06	6.74E-04	4.97E+00
TRF	0.326206	10.38794	4.579467	0.000353	0.016318	0.239545765
CNTNAP4	0.325434	9.846148	5.872479	2.94E-05	0.003428	2.669647322

OGFRL1	0.316944	10.94292	4.625934	0.000321	0.015485	0.330255984
D12ERTD647E	0.315275	9.969361	7.862237	9.97E-07	3.76E-04	5.94E+00
RASL10A	0.313269	9.588202	5.311853	8.42E-05	6.95E-03	1.642133018
LITAF	0.312848	9.402568	6.244316	1.50E-05	2.16E-03	3.326281326
AHI1	0.31171	10.57708	5.172624	0.00011	8.00E-03	1.380370424
SNRPD3	0.310925	10.62739	5.022377	0.000147	0.00963	1.095169336
PIK3R3	0.309435	10.28267	5.386326	7.30E-05	0.00637	1.781112573
FEZF2	0.307236	9.724721	4.691593	0.000282	0.014105	0.458080558
FANCD2	0.305371	8.110228	7.151087	3.15E-06	7.54E-04	4.840758879
RGS9	0.303976	8.980841	5.260751	9.28E-05	7.05E-03	1.546346213
LY6G6E	0.301729	7.750259	5.880505	2.90E-05	3.43E-03	2.684035024
EVI2A	0.300796	8.523316	5.512408	5.75E-05	5.52E-03	2.014708722
ST3GAL5	0.30026	9.919604	5.105675	0.000125	0.008634	1.253628759
RPS26	0.298635	11.85228	5.403605	7.07E-05	6.22E-03	1.813252515
ARHGAP25	0.298005	8.662254	6.038876	2.17E-05	0.002796	2.966010853
PADI6	0.297851	7.451042	6.088526	1.99E-05	2.59E-03	3.05365402
ZMIZ1	0.297064	8.750812	6.269359	1.44E-05	2.10E-03	3.369767531
TMEM132D	0.292858	8.122685	7.255845	2.64E-06	6.65E-04	5.00776405
SUMO3	0.291172	9.241103	3.997873	0.001143	0.032088	-0.909649739
BLMH	0.29116	8.428104	5.109351	0.000124	0.008629	1.260601839
LMO3	0.289471	8.373424	6.238627	1.52E-05	2.16E-03	3.316388848
RAMP3	0.288744	8.392446	6.798962	5.69E-06	0.001166	4.267349072
P140	0.288029	8.305353	5.995422	2.35E-05	2.95E-03	2.889007513
LRFN2	0.286158	8.801948	3.934093	0.001303	0.034803	-1.036760072
DBNDD2	0.285931	11.0243	6.438277	1.06E-05	0.001812	3.660638093
LOC10004758	0.282914	9.874884	5.032957	0.000144	0.009568	1.115341196
CDH13	0.282687	8.439697	6.993361	4.09E-06	9.00E-04	4.59E+00
APPBP2	0.282053	10.5446	4.748293	0.000252	0.013502	0.568120633
CRYM	0.281089	11.54698	3.961087	0.001233	0.033592	-0.982947474
ANO4	0.280008	8.816755	4.7326	0.00026	0.013719	0.537698112
TMSB10	0.279349	11.06222	4.918973	0.00018	0.011114	0.897316876
GPN3	0.276842	8.406506	5.155838	0.000114	0.0081	1.348644957
DYNLL2	0.275974	8.9233	5.613077	4.76E-05	4.83E-03	2.20E+00
ZYX	0.275789	11.00474	4.995141	0.000155	0.01	1.043176464
PLEKHA2	0.27473	9.379361	5.159262	0.000113	0.0081	1.355119043
EG434858	0.271693	8.69901	6.714515	6.58E-06	1.22E-03	4.127059045
EEF2	0.27014	8.350257	5.434686	6.66E-05	0.006013	1.87096427
BCL11B	0.269008	10.63047	3.886128	0.001437	0.036515	-1.13242335
TATDN2	0.267212	8.410132	6.08729	1.99E-05	0.002593	3.051476655
GSK3B	0.266202	9.105032	4.272594	0.000654	0.023702	-0.364082683
CBX5	0.265504	8.903005	4.008574	0.001119	3.17E-02	-0.888334255
RASL11B	0.261901	11.25043	6.595553	8.09E-06	1.45E-03	3.93E+00
ALAS2	0.26056	7.583163	4.08517	0.000957	0.029605	-0.735894764

LOC10004150	0.25962	8.657669	4.649746	0.000306	0.014974	0.376662005
FKBP2	0.254815	9.830134	4.548272	0.000375	0.016851	0.178537075
GABBR1	0.254073	8.463038	5.76111	3.61E-05	3.97E-03	2.469043476
LOC10004613	0.253444	8.011977	4.75873	0.000247	0.013293	0.588340533
PSMB10	0.252859	9.812459	4.502734	0.000411	0.01768	0.08932394
DYNC1I1	0.251687	9.345363	5.495535	5.94E-05	5.60E-03	1.98357222
VANGL2	0.251133	8.243392	6.312204	1.33E-05	2.06E-03	3.443949314
ZDHHC9	0.250827	8.576029	4.4158	0.000489	0.019905	-0.081472774
POLD2	0.249868	8.169336	5.27087	9.11E-05	7.00E-03	1.565340272
COL5A1	0.249742	8.052943	6.527431	9.10E-06	1.57E-03	3.812428642
6430598A04R	0.249098	8.393332	4.767681	0.000243	0.013263	0.60567257
GM1821	0.248521	14.51018	5.661422	4.35E-05	4.55E-03	2.287974919
LOC10004612	0.248317	7.99283	4.414862	0.00049	0.019905	-0.083320519
CLDN11	0.248315	12.82258	4.603057	0.000336	0.015779	0.28562152
STAC2	0.246349	10.06809	4.476379	0.000433	0.018148	0.037609116
SLC35F3	0.246099	10.5571	5.085627	0.00013	0.00886	1.215567095
SYT5	0.244676	10.3186	4.881756	0.000194	0.011617	0.825806989
RAP1GAP	0.243312	7.960334	4.662564	0.000299	0.014733	0.401618834
COG1	0.242909	8.320809	5.368852	7.55E-05	0.006532	1.748568143
NUP93	0.242263	8.254299	4.02679	0.001078	0.031278	-0.85206092
KHDRBS1	0.241122	8.73763	5.020027	0.000148	0.00963	1.090685105
EZH1	0.239171	7.714855	4.978902	0.00016	1.02E-02	1.012135808
VTI1B	0.238599	12.22503	4.37756	0.000529	0.020835	-0.156791872
AGPAT4	0.238511	9.419419	4.30672	0.00061	0.02299	-0.296605636
MMP16	0.238449	8.328217	3.686688	0.002165	4.81E-02	-1.53E+00
ARHGEF15	0.237402	8.08675	5.317903	8.32E-05	0.00695	1.653449425
LOC10004325	0.236612	9.951471	4.515598	0.000401	0.017397	0.114543403
PSMB4	0.236517	12.44938	4.145251	0.000846	0.027399	-0.616501887
1500011H22R	0.23562	10.14294	4.020876	0.001091	0.031365	-0.863836527
HSD17B11	0.235383	7.926266	4.937598	0.000174	0.010908	0.93304472
NME2	0.235	10.32974	4.77499	0.000239	0.013141	0.619819608
VPS25	0.234495	8.981466	5.527866	5.59E-05	0.00541	2.043199626
ATP2B1	0.234103	9.343558	4.373457	0.000533	0.020835	-0.164879971
NMRAL1	0.23381	8.267957	5.286857	8.83E-05	0.007001	1.595321274
RCAN3	0.23336	9.859845	3.944845	0.001274	3.44E-02	-1.015323959
MOBP	0.233312	9.052363	5.325843	8.20E-05	6.92E-03	1.668295262
HRMT1L2	0.232888	10.48449	4.253057	0.00068	0.024325	-0.402746217
GAB1	0.232142	9.003693	5.435949	6.65E-05	0.006013	1.873306102
DPYSL5	0.23193	7.951066	4.788025	0.000233	0.012942	0.645034391
ORC6L	0.231907	9.613107	4.707184	0.000273	0.01401	0.488371799
GRIK1	0.231209	8.822511	4.489672	0.000422	0.017956	0.063700669
PDLIM1	0.230388	8.85	4.209783	0.000742	0.025654	-0.488468884
LRRTM2	0.229543	9.535346	4.080829	0.000965	0.029781	-0.744528938

SOX5	0.22812	8.718758	5.18133	0.000108	8.00E-03	1.39681199
COX17	0.22789	11.62163	3.710215	0.002063	4.64E-02	-1.48E+00
DLG4	0.227613	9.496925	3.819769	0.001647	0.040233	-1.264840983
WSCD1	0.227225	8.908179	4.017456	0.001098	3.15E-02	-0.870647092
PRR7	0.226718	9.384036	4.498759	0.000414	0.017703	0.081527417
PLCL2	0.226252	10.44229	4.559549	0.000367	0.016688	0.200602313
ACTR1A	0.225165	10.82985	5.276126	9.01E-05	0.007001	1.57520111
PIGK	0.225097	7.897873	5.179705	0.000108	0.008004	1.393744144
NT5C3	0.224887	9.616987	5.024694	0.000146	0.00963	1.099587659
UNC5B	0.224681	7.855626	5.42573	6.78E-05	6.06E-03	1.854348604
DDR1	0.224077	8.746017	4.345532	0.000564	0.021642	-0.219959442
DCTN1	0.223405	9.172176	4.717037	0.000268	0.013871	0.507501341
CCL21A	0.222402	9.212146	4.093248	0.000941	0.029293	-0.719832428
STARD3NL	0.222166	8.993388	4.220249	0.000727	0.025229	-0.467726521
CASK	0.221946	10.107	4.184861	0.000781	0.026409	-0.53788685
CDS2	0.221907	7.920417	4.903823	0.000186	1.14E-02	0.868226568
MAGI2	0.221738	7.583132	4.400717	0.000505	0.020338	-0.111169267
TCEAL3	0.220565	8.924958	4.602955	0.000336	0.015779	0.285422319
EXTL1	0.219543	8.809089	4.022838	0.001086	0.031325	-0.859928511
D11MOH35	0.217242	7.827309	5.844545	3.10E-05	3.55E-03	2.619499862
DRD1A	0.21604	8.898292	3.963323	0.001227	0.033546	-0.978492079
LOC10004770	0.215854	10.11424	3.894573	0.001413	0.03627	-1.115577756
SSU72	0.215467	11.41431	4.274602	0.000651	0.023688	-0.360109092
ARRDC4	0.214684	8.320606	4.17093	0.000803	0.026621	-0.56552742
RNF113A2	0.214206	9.034929	4.057684	0.001012	0.030257	-0.790570765
LSM8	0.213394	9.56242	3.807777	0.001688	4.09E-02	-1.288775065
IVNS1ABP	0.213157	8.896972	4.069851	0.000987	0.029924	-0.766364269
DPYSL3	0.212903	8.247197	3.803076	0.001704	4.09E-02	-1.30E+00
PSMD4	0.212686	11.68837	4.280246	0.000644	0.023663	-0.348944976
H2-T23	0.212437	8.456817	4.81632	0.00022	0.012484	0.699705007
MBP	0.21204	12.4868	4.969632	0.000163	0.010307	0.994402091
NRP1	0.211727	9.840109	3.889258	0.001428	0.03649	-1.126179927
1190003J15RI	0.210432	7.933184	4.603781	0.000336	0.015779	0.287035692
MECP2	0.210115	9.311704	5.14837	0.000115	8.11E-03	1.334519943
SAMD9L	0.209404	8.2146	4.003226	0.001131	0.031904	-0.898985644
EXOC4	0.209185	8.785003	4.759542	0.000247	0.013293	0.589913094
ZFP282	0.208713	8.667863	4.164224	0.000814	0.026773	-0.578835638
BCLAF1	0.208095	10.99544	4.164488	0.000814	0.026773	-0.578311404
EXOSC4	0.20767	8.799763	3.806394	0.001693	0.040879	-1.291536217
D430041B17R	0.206259	9.123235	4.476915	0.000433	0.018148	0.038663285
D030056L22RI	0.206206	10.90586	3.902502	0.00139	0.03605	-1.099761986
TPBG	0.205956	8.884654	4.073959	0.000979	0.029919	-0.758191315
CLPP	0.204998	8.906726	3.885402	0.001439	0.036515	-1.133871788

PACRG	0.204377	9.414803	4.419204	0.000486	0.019885	-0.074775295
IL11RA1	0.20408	11.04973	4.035309	0.001059	0.031132	-0.835100223
TPM3	0.204064	8.404092	4.620472	0.000325	0.015584	0.319603189
GADD45A	0.203275	8.596068	4.57217	0.000358	0.016486	0.225283495
TPR	0.202675	9.902227	3.915704	0.001353	0.035511	-1.073430714
5430437P03RI	0.201542	9.352445	4.722822	0.000265	0.013852	0.518728396
LOR	0.20074	9.268759	4.971846	0.000162	0.010307	0.998639612
CARHSP1	0.200392	8.668882	4.814037	0.000221	1.25E-02	0.6952965
ITGB4	0.200262	7.81662	4.501433	0.000412	0.01768	0.086771764
SFRS5	0.200049	10.07632	4.398723	0.000507	0.020338	-0.115095358
RPS4X	0.199753	12.70981	4.029469	0.001072	0.031278	-0.846727247
XAB1	0.19931	9.11145	4.564444	0.000363	0.016669	0.210175923
NETO1	0.199006	8.955297	4.106434	0.000916	0.0286	-0.693619496
0610006I08RII	0.198959	10.29162	3.766096	0.001839	0.043479	-1.371975382
JOSD2	0.198718	9.284611	3.683544	0.002179	4.82E-02	-1.536754958
ODZ3	0.198289	7.82894	4.50641	0.000408	0.017648	0.096532366
PCGF5	0.19766	9.205501	4.174493	0.000798	0.026555	-0.558457271
OG9X	0.19743	7.473515	4.046258	0.001036	0.030614	-0.813306921
MAK16	0.196968	8.531099	3.732477	0.001971	0.044917	-1.439084055
F2R	0.196831	8.729049	4.589012	0.000346	0.016081	0.258195217
AU040829	0.196249	12.2194	3.960123	0.001235	0.033592	-0.984868461
UHRF2	0.194994	9.131067	4.040307	0.001048	0.030902	-0.825152075
UPF3A	0.194585	7.99019	4.24641	0.000689	0.024407	-0.415905937
GAL3ST1	0.194094	8.251595	3.834015	0.001599	0.03953	-1.236407086
1810014F10RI	0.193164	8.532179	4.062571	0.001002	0.030118	-0.78084607
2010106G01R	0.193009	7.769611	4.377084	0.000529	0.020835	-0.157730374
EXOSC7	0.19275	10.0605	3.726982	0.001993	0.045113	-1.450052953
SMARCD2	0.191961	8.141246	3.97962	0.001187	0.0327	-0.94601346
TXNRD2	0.191414	7.990596	4.366565	0.000541	0.020972	-0.178468827
OLFML2B	0.190586	8.523727	3.83781	0.001587	0.039316	-1.228833516
POP4	0.190069	10.13204	3.738855	0.001945	0.044677	-1.42635253
MMP24	0.189884	9.045771	3.727095	0.001993	4.51E-02	-1.449826832
GDPD1	0.189746	10.0911	3.902318	0.00139	0.03605	-1.100128501
VAPB	0.187748	9.065473	4.381768	0.000524	0.020835	-0.148499461
MYO5B	0.187476	7.458379	4.174414	0.000798	0.026555	-0.558613729
ELOVL6	0.186535	9.634029	4.160441	0.000821	0.026896	-0.586345159
MYL4	0.186122	10.89938	4.455048	0.000452	0.01872	-0.004287915
DDX5	0.185812	7.667202	4.277918	0.000647	0.023688	-0.353550429
INPP4B	0.185347	7.602162	4.280675	0.000643	0.023663	-0.34809586
TRIT1	0.185294	8.233818	3.980139	0.001186	0.0327	-0.944979857
CHCHD1	0.18521	12.02609	4.452413	0.000455	0.018746	-0.009465692
6720458F09RI	0.184949	9.604516	4.193383	0.000768	0.026297	-0.520985071
C030011O14R	0.179378	9.295485	3.722803	0.00201	4.53E-02	-1.458394177

SEMA3F	0.17886	7.856118	3.938464	0.001291	0.034669	-1.028046258
SFRS12	0.17836	7.526806	4.028112	0.001075	0.031278	-0.849428591
RALY	0.178158	9.379945	3.669676	0.002243	4.93E-02	-1.56E+00
FGF10	0.178013	8.106554	4.520763	0.000396	0.01735	0.124665871
KCNQ2	0.177258	7.858577	3.983831	0.001177	0.032675	-0.93762457
1500031L02RI	0.176826	11.11723	3.860512	0.001515	0.038062	-1.18353258
SLC38A5	0.175792	8.130117	4.247625	0.000688	0.024407	-0.413499145
GRIA1	0.175458	10.18257	3.831397	0.001608	3.97E-02	-1.241632824
USP1	0.174528	8.405441	4.183361	0.000783	0.026409	-0.540863229
BC002163	0.174241	11.47851	4.071408	0.000984	0.029921	-0.76326668
ENPP4	0.173828	8.486283	4.148963	0.00084	0.027277	-0.609130586
CHRM2	0.17319	7.520361	4.37746	0.000529	0.020835	-0.156990155
ABI2	0.172979	12.6459	3.748091	0.001908	0.044262	-1.407917273
YIF1B	0.172919	9.341596	3.80323	0.001704	0.040879	-1.297852115
RANBP1	0.172353	10.34559	3.759548	0.001864	0.043809	-1.38504611
2410066E13RI	0.170714	7.828188	3.738244	0.001947	4.47E-02	-1.43E+00
TUBA1B	0.170311	14.65323	3.743959	0.001925	4.45E-02	-1.416164997
DUS4L	0.168994	8.419626	4.263645	0.000666	0.02389	-0.381789503
D16H22S680E	0.168793	11.92436	3.753033	0.001889	4.40E-02	-1.398051509
DHRS7B	0.165597	8.680331	4.076616	0.000973	0.029863	-0.752906742
C130090K23RI	0.16492	7.740552	4.223541	0.000722	0.025182	-0.461202679
DPM1	0.16478	8.642563	3.736782	0.001953	4.47E-02	-1.430491946
MUM1	0.157581	9.767936	3.829691	0.001614	0.039697	-1.245037776
KCNIP4	0.157462	7.685055	3.751659	0.001894	4.40E-02	-1.400794818
1700021C14RI	0.157105	8.067149	3.809184	0.001683	0.040879	-1.285966826
FADS2	0.154378	7.895403	3.681204	0.00219	0.048248	-1.541425102
RPA3	0.151714	9.120194	3.774735	0.001807	0.042993	-1.35473014
TMED4	0.151416	9.128541	4.138337	0.000858	0.027677	-0.630232223
CACNG5	0.149619	7.778077	4.156818	0.000827	0.027011	-0.593536574
EMD	0.146751	8.159979	3.684152	0.002177	4.82E-02	-1.535541696
SLC44A1	0.143725	8.392265	3.75217	0.001892	0.044035	-1.399773435
RYR1	-0.13433	7.694155	-3.68983	0.002151	4.79E-02	-1.524213018
TLE1	-0.14633	9.008041	-3.6903	0.002149	4.79E-02	-1.523271475
TPD52L1	-0.14809	8.029941	-3.84202	0.001573	0.039254	-1.22044065
MAPK10	-0.15248	8.063982	-3.91709	0.001349	0.035498	-1.07066527
B2M	-0.15586	11.03704	-3.86981	0.001486	0.037432	-1.164979153
RGL1	-0.15697	10.75633	-3.72961	0.001982	4.51E-02	-1.444810238
THBD	-0.16271	7.41851	-3.68158	0.002188	0.048248	-1.54066762
CADM2	-0.16343	8.689475	-3.90639	0.001379	0.036016	-1.092001597
DAB2	-0.16546	7.640299	-3.92563	0.001325	3.52E-02	-1.053644408
TMEM85	-0.166	11.62307	-4.3079	0.000609	0.02299	-0.294281511
EHMT2	-0.16628	9.184744	-3.83881	0.001584	3.93E-02	-1.226842601
5730410I19RII	-0.16686	9.275464	-4.13107	0.000871	0.027776	-0.644671241

EMID2	-0.17024	7.543926	-3.80902	0.001684	0.040879	-1.286299563
SUPT5H	-0.17099	9.746238	-4.00199	0.001134	0.031904	-0.901447036
ANKRD6	-0.17152	7.631742	-4.23526	0.000705	0.024798	-0.437989369
RXFP3	-0.17227	7.422516	-3.91119	0.001365	0.035752	-1.082440429
CAPN2	-0.17424	8.271079	-4.1782	0.000792	0.026518	-0.551095365
ARF4	-0.1759	7.33964	-4.11494	0.0009	0.028446	-0.676720596
HSPA1A	-0.17605	7.75233	-4.29889	0.00062	0.023274	-0.312083876
CHFR	-0.17669	9.261868	-4.37416	0.000532	0.020835	-0.163497525
LRRTM3	-0.17912	8.179193	-3.92028	0.00134	3.54E-02	-1.064306705
CNPY3	-0.18001	7.660573	-3.77249	0.001815	0.043043	-1.359220028
IGFBP2	-0.18049	7.749552	-4.84899	0.000207	1.21E-02	0.762717888
YWHAZ	-0.18072	12.78527	-3.77414	0.001809	4.30E-02	-1.36E+00
RASGRF1	-0.18121	12.43773	-4.61242	0.00033	0.015693	0.303896312
GRSF1	-0.18259	10.11151	-3.80348	0.001703	0.040879	-1.297345833
SLC23A3	-0.1839	7.441943	-4.39848	0.000507	0.020338	-0.1155646
ARPP21	-0.18391	12.3379	-4.5616	0.000365	0.016688	0.204615945
LINGO2	-0.18397	8.577158	-4.05227	0.001023	0.030411	-0.80134453
DLX2	-0.18439	7.380128	-3.948	0.001266	0.03426	-1.009037741
SEC14L1	-0.18478	8.730405	-4.48345	0.000427	0.018108	0.051496988
BMPER	-0.18595	8.153401	-3.85771	0.001524	3.82E-02	-1.189118705
AK3	-0.18726	9.791201	-4.11107	0.000907	0.02855	-0.684413753
BC002230	-0.18757	7.796825	-3.69895	0.002111	4.72E-02	-1.506008131
ZNRF1	-0.18828	8.790423	-3.74127	0.001935	0.044677	-1.421534161
SULT1A1	-0.18888	7.931959	-4.7439	0.000254	0.013551	0.559604613
ABHD6	-0.18925	7.722196	-4.26508	0.000664	0.02389	-0.37894791
TRPC3	-0.18952	8.770999	-3.73408	0.001964	0.044866	-1.435885559
CAMTA2	-0.1915	12.5099	-4.01663	0.0011	0.031467	-0.872292537
CRIPT	-0.19168	8.346967	-4.20665	0.000747	0.025681	-0.494678841
GNG4	-0.19264	8.709831	-4.2901	0.000631	0.023441	-0.329464961
BICD2	-0.19496	9.32708	-3.89066	0.001424	0.036474	-1.123382384
RILPL1	-0.19541	9.735163	-4.10876	0.000912	0.02855	-0.689004707
TTYH1	-0.1955	10.85504	-4.18702	0.000777	0.026409	-0.533595437
CXCL14	-0.19562	8.932043	-3.75525	0.001881	4.40E-02	-1.393631492
CDC42EP3	-0.1958	9.189026	-3.95143	0.001257	0.034107	-1.002193431
TAX1BP3	-0.1973	8.556084	-4.72036	0.000266	0.013852	0.513948956
FUT8	-0.19756	8.659682	-4.69658	0.000279	0.0141	0.46777932
CYR61	-0.19762	7.222309	-3.70232	0.002097	4.70E-02	-1.499282757
CLCN4-2	-0.19828	8.232154	-4.00859	0.001119	0.031731	-0.888301493
NPY	-0.19942	11.02052	-3.92334	0.001332	0.035222	-1.058201346
SCHIP1	-0.20078	11.64477	-3.8479	0.001555	0.038875	-1.208704602
SPAG5	-0.20088	8.134456	-4.17009	0.000805	0.026621	-0.56720004
CDH8	-0.2013	8.211453	-5.48283	6.08E-05	5.63E-03	1.960103674
PRR13	-0.20217	11.09519	-4.52971	0.000389	0.017268	0.142193086

HIST1H2BN	-0.20297	7.967064	-4.85001	0.000206	0.012114	0.76468458
GPC5	-0.20337	8.91444	-4.36883	0.000538	0.020953	-0.17399968
BMP1	-0.20393	8.137301	-5.30309	8.56E-05	0.007001	1.625737133
EG328644	-0.20653	8.233297	-4.0562	0.001015	0.030257	-0.79351307
DKKL1	-0.20786	8.274005	-4.0871	0.000953	0.029575	-0.732060754
AMIGO1	-0.20932	8.667317	-4.29181	0.000629	0.023441	-0.326069095
E430002G05R	-0.21199	8.412399	-4.62999	0.000319	0.015481	0.338166812
C130074G19R	-0.21208	7.887178	-4.5233	0.000394	0.01735	0.129641733
IL6ST	-0.2124	7.659632	-3.82192	0.00164	4.01E-02	-1.26053913
SLC6A13	-0.21313	7.414268	-4.27507	0.00065	0.023688	-0.359183188
GJB2	-0.21435	7.55037	-5.26802	9.15E-05	0.007001	1.559990593
ZFP238	-0.21478	10.43949	-4.20878	0.000744	0.025654	-0.490466349
RRN3	-0.21561	8.707953	-3.8227	0.001637	0.040148	-1.258996916
EFCAB1	-0.21631	7.730321	-4.43773	0.000468	0.019232	-0.038328825
DEDD2	-0.21689	8.140133	-3.76535	0.001842	0.043479	-1.373471917
4930455C21RI	-0.21751	8.093514	-4.59188	0.000344	0.01606	0.26380476
SHFM1	-0.21864	11.52601	-3.72618	0.001996	0.045113	-1.451663105
KLHL7	-0.21933	10.11401	-5.1761	0.000109	0.008004	1.38694102
PDSS1	-0.21943	8.000255	-4.05614	0.001015	0.030257	-0.793642145
LOC10004417	-0.22007	8.509613	-4.24957	0.000685	0.024407	-0.409639138
PTGES3	-0.22079	9.397217	-3.73858	0.001946	4.47E-02	-1.426899088
HSPA2	-0.22192	10.32245	-3.94136	0.001283	3.46E-02	-1.022276437
DAB2IP	-0.22207	10.18955	-4.70175	0.000276	0.014023	0.477814715
INSM1	-0.22364	7.671825	-3.92503	0.001327	0.035189	-1.054839905
ZFP451	-0.22387	8.409702	-4.14998	0.000838	0.027277	-0.607114244
OGN	-0.22452	7.387425	-4.55294	0.000372	0.016781	0.187673857
PAFAH1B1	-0.227	9.107184	-3.89489	0.001412	3.63E-02	-1.114942554
MAPRE3	-0.2277	8.088653	-4.26964	0.000658	0.023763	-0.36992823
FAM84A	-0.22915	7.918033	-4.005	0.001127	3.19E-02	-0.895461588
ACSBG1	-0.2295	8.492413	-4.72023	0.000266	0.013852	0.513701741
CD83	-0.22972	8.422298	-4.07892	0.000969	0.029809	-0.748317874
RGS4	-0.23027	12.45033	-5.31111	8.43E-05	6.95E-03	1.640748418
TOP2B	-0.2321	7.583709	-3.97973	0.001187	0.0327	-0.945793711
MEGF9	-0.23274	9.484822	-4.52008	0.000397	0.01735	0.123327535
DLGAP2	-0.23469	9.560601	-5.43444	6.67E-05	6.01E-03	1.870507212
FAM148C	-0.23562	8.862439	-3.89986	0.001397	0.036143	-1.105022803
HSP105	-0.23709	11.51016	-3.87435	0.001472	0.037263	-1.155925333
SLC2A13	-0.23796	11.19407	-4.32299	0.00059	0.022406	-0.264457207
COCH	-0.23865	7.74143	-4.61437	0.000329	0.015693	0.307703685
ARHGAP20	-0.23903	10.02217	-4.65699	0.000302	0.014828	0.39077304
SLC40A1	-0.23914	8.859273	-4.62837	0.00032	0.015481	0.335002836
CPNE4	-0.23922	10.03049	-4.47194	0.000437	0.018239	0.028886513
TIMP4	-0.23931	8.305034	-5.4183	6.87E-05	0.006095	1.840560961

FGF12	-0.23946	9.618374	-3.88762	0.001433	3.65E-02	-1.129445164
HPCA	-0.24039	12.20839	-4.67738	0.00029	0.014441	0.430445437
PAM	-0.24111	8.054158	-4.1373	0.00086	0.027677	-0.632294553
ARCN1	-0.2418	7.924603	-4.79951	0.000228	0.012719	0.667240617
COBL	-0.24227	8.931042	-3.98497	0.001174	0.032675	-0.935363899
TGFB2	-0.24312	7.600033	-4.35499	0.000553	0.02131	-0.20129331
SCCPDH	-0.24478	11.79798	-4.55247	0.000372	0.016781	0.186751508
EVL	-0.24499	10.05722	-4.70246	0.000276	0.014023	0.479197533
NECAB1	-0.24668	8.96109	-4.06428	0.000998	3.01E-02	-0.777453528
TPCN1	-0.24669	10.05106	-3.90437	0.001385	0.03605	-1.096039192
LOC10004612	-0.24739	12.16889	-4.02706	0.001077	3.13E-02	-0.85152438
VAT1L	-0.25014	10.00021	-3.80016	0.001715	0.041032	-1.303978742
APOE	-0.25216	10.56576	-3.97137	0.001207	0.033083	-0.962445145
ZFP36L1	-0.2536	9.402585	-4.93381	0.000175	1.09E-02	0.925786933
PTPRB	-0.25416	8.660836	-4.2228	0.000723	0.025182	-0.462680943
FHOD3	-0.25427	8.221549	-3.76431	0.001846	4.35E-02	-1.375547658
RELN	-0.25565	10.6203	-4.99442	0.000155	0.01	1.041791457
NRN1	-0.2564	12.58454	-5.2903	8.77E-05	0.007001	1.601780715
SMARCC2	-0.25769	9.053504	-4.04883	0.00103	0.03054	-0.808183339
GPR37L1	-0.25841	9.993285	-5.15187	0.000114	0.008108	1.341144538
PVRL3	-0.25974	8.039421	-6.54251	8.87E-06	1.56E-03	3.84E+00
CDKN1C	-0.26313	8.447607	-5.78779	3.44E-05	0.003863	2.517272502
TJP1	-0.26357	8.397151	-4.06855	0.00099	0.029924	-0.768958387
SYNGR1	-0.26486	9.280637	-4.70708	0.000273	1.40E-02	0.488166546
RREB1	-0.26653	8.059592	-6.73215	6.39E-06	1.20E-03	4.156438497
2510009E07RI	-0.26701	9.425649	-7.1203	3.31E-06	7.76E-04	4.791360819
ALDOA	-0.26745	12.53948	-4.1225	0.000887	0.028179	-0.661683926
RBM28	-0.2696	9.240326	-4.54465	0.000378	0.016901	0.171453115
RGS10	-0.26984	9.908404	-4.89022	0.000191	0.011582	0.84208477
P2RY12	-0.27	8.52596	-4.24122	0.000697	0.024582	-0.426189514
SLC4A3	-0.27051	9.234296	-7.3457	2.28E-06	6.08E-04	5.149706293
KRT12	-0.27062	9.487761	-5.73038	3.83E-05	4.08E-03	2.413384807
LOC10004765	-0.27098	10.44043	-6.36923	1.20E-05	1.98E-03	3.54E+00
LOC10004701	-0.27161	8.987815	-4.78413	0.000235	1.30E-02	0.637507891
OSBP2	-0.27259	9.051612	-4.11666	0.000897	0.028431	-0.67330287
ITGB5	-0.27323	8.32545	-5.90502	2.77E-05	0.003364	2.72791823
RASSF3	-0.27538	8.629859	-3.89865	0.001401	3.61E-02	-1.107448033
LOC10004885	-0.27567	8.947436	-3.8047	0.001699	0.040879	-1.294915469
CLSTN1	-0.27573	10.08447	-4.39437	0.000511	0.020429	-0.123662251
TYKI	-0.27872	8.792128	-4.87733	0.000195	0.011652	0.817292288
SLC1A3	-0.2791	12.60486	-6.34182	1.26E-05	2.05E-03	3.50E+00
6030405A18R	-0.28009	9.172804	-4.88894	0.000191	0.011582	0.839630841
NSFL1C	-0.28024	9.604223	-4.02626	0.001079	0.031278	-0.853120508

RIMS3	-0.28058	11.09785	-4.01023	0.001115	0.031731	-0.885038526
EVC2	-0.28202	8.157568	-5.54067	5.45E-05	0.005369	2.066769937
GPM6A	-0.28224	10.32941	-4.67401	0.000292	0.014469	0.423892618
ATP1B2	-0.28492	10.25857	-4.7621	0.000245	0.013293	0.594871728
CACNG3	-0.28604	8.812613	-5.50522	5.83E-05	0.005544	2.001441654
SEPT9	-0.28753	8.339702	-5.15647	0.000113	0.0081	1.349849707
PECI	-0.2877	8.989299	-4.32431	0.000589	0.022406	-0.261856369
TMEM32	-0.28797	8.916324	-4.82821	0.000215	1.23E-02	0.7226562
RNF144A	-0.28824	8.82717	-4.81162	0.000222	0.012484	0.690635904
GCNT2	-0.2886	9.294167	-5.32648	8.19E-05	6.92E-03	1.67E+00
HSD11B1	-0.28881	9.321022	-7.44208	1.95E-06	5.42E-04	5.300638191
ADCYAP1	-0.28972	8.629429	-4.51902	0.000398	0.01735	0.121241393
RWDD4A	-0.29031	8.637847	-3.99191	0.001157	0.032357	-0.921518154
GLRX2	-0.29107	8.893965	-4.13118	0.000871	0.027776	-0.644449213
PMPCB	-0.29163	8.513254	-4.3575	0.000551	0.02128	-0.196348184
ABAT	-0.29226	10.5606	-5.07079	0.000134	0.009003	1.187370071
EGR2	-0.29254	8.305679	-7.48159	1.83E-06	5.22E-04	5.36E+00
C1QL3	-0.29391	8.877394	-4.07285	0.000981	0.029919	-0.760399551
KIRREL3	-0.29441	8.251422	-4.32533	0.000587	0.022406	-0.259845727
GPM6B	-0.29683	8.30149	-4.2838	0.000639	0.023658	-0.341908147
KCTD3	-0.29746	9.957426	-4.18602	0.000779	0.026409	-0.535590631
EIF3K	-0.29824	9.353701	-3.87165	0.001481	0.037379	-1.161299496
4931406C07RI	-0.29832	8.329672	-6.00227	2.32E-05	2.95E-03	2.90116192
RTN4RL1	-0.29885	9.196549	-4.10875	0.000912	0.02855	-0.689023799
RORA	-0.30007	8.983488	-5.05005	0.000139	9.31E-03	1.147913632
B930076A02	-0.30053	9.14238	-6.10733	1.92E-05	2.56E-03	3.086753128
KCNK2	-0.3013	8.964489	-4.18038	0.000788	0.026485	-0.546774268
2810022L02RI	-0.30394	8.042042	-5.27572	9.02E-05	7.00E-03	1.574447641
LUZP2	-0.30598	10.792	-4.92736	0.000177	0.010998	0.913402319
RAD23B	-0.30648	10.35507	-4.73665	0.000258	0.013678	0.545545439
PIGQ	-0.3068	9.942993	-4.47836	0.000432	0.018148	0.041492792
SCG3	-0.30835	11.00394	-3.70484	0.002086	4.68E-02	-1.49E+00
IAP	-0.30887	11.19885	-4.81598	0.000221	0.012484	0.699041902
A530082C11R	-0.30998	8.569516	-5.35127	7.81E-05	0.006699	1.715791546
KPNB1	-0.31131	10.22433	-4.13469	0.000865	0.02774	-0.637477403
LOC10004446	-0.31246	11.05575	-4.02328	0.001085	0.031325	-0.859058628
CRHR1	-0.31293	7.64078	-5.07098	0.000134	0.009003	1.187737599
C1QTNF4	-0.3217	10.68126	-5.27296	9.07E-05	0.007001	1.569256341
DUSP6	-0.32373	10.11331	-6.32979	1.29E-05	2.05E-03	3.474319925
IGF2	-0.32392	9.499371	-5.74503	3.72E-05	4.01E-03	2.439934501
PDYN	-0.32494	9.007365	-6.73208	6.39E-06	1.20E-03	4.156331647
TMCC2	-0.32627	9.238876	-5.57722	5.09E-05	0.00507	2.133946786
DCN	-0.32709	7.854345	-7.75051	1.19E-06	3.97E-04	5.774428261

PRELP	-0.3277	8.393626	-6.64035	7.48E-06	1.36E-03	4.002957697
VIP	-0.32789	10.46507	-5.96375	2.49E-05	3.06E-03	2.83E+00
PLCH2	-0.32925	9.267879	-4.53326	0.000387	0.017218	0.149139101
DDIT4L	-0.32972	8.776336	-7.5658	1.60E-06	4.68E-04	5.492357417
NDRG2	-0.33295	9.481645	-5.09925	0.000127	0.008685	1.241444071
FSTL4	-0.33309	8.77555	-6.14229	1.80E-05	2.44E-03	3.148138082
NOL4	-0.33933	9.808341	-6.87444	5.01E-06	1.08E-03	4.39E+00
CCK	-0.3394	12.17367	-7.01021	3.98E-06	9.00E-04	4.613577572
SLC24A3	-0.33973	11.68753	-5.6696	4.28E-05	4.52E-03	2.302878381
PRKAR1B	-0.33984	10.75021	-5.87017	2.96E-05	0.003428	2.665505296
JAK1	-0.34261	8.622206	-4.2241	0.000721	0.025182	-0.460098556
SLC7A10	-0.34271	8.636367	-6.78985	5.78E-06	1.17E-03	4.252261818
BHLHB5	-0.34475	7.83986	-3.97658	0.001194	0.032818	-0.952071835
4933439C20RI	-0.34492	10.97684	-5.98912	2.38E-05	2.95E-03	2.87781004
LOC10004604	-0.34954	11.75974	-4.18877	0.000775	0.026409	-0.530131696
SLC12A5	-0.35146	8.211898	-3.77968	0.001788	0.042699	-1.344856969
AI314180	-0.35912	9.368344	-3.9912	0.001159	0.032357	-0.922940996
CAR4	-0.36107	8.937582	-5.77703	3.51E-05	0.003899	2.497832012
PKNOX2	-0.36215	8.955686	-7.95653	8.61E-07	3.36E-04	6.08E+00
LOC10004540	-0.36265	12.55801	-7.26957	2.59E-06	6.65E-04	5.03E+00
GUCY1B3	-0.36436	9.216574	-3.93692	0.001295	0.034691	-1.031131543
ARNTL	-0.36593	8.824861	-6.27313	1.43E-05	2.10E-03	3.376306612
FAM134A	-0.36622	10.51222	-4.8491	0.000207	0.012114	0.762939272
UBE2J1	-0.36768	8.766111	-5.26887	9.14E-05	7.00E-03	1.561583315
VGF	-0.36772	11.799	-8.09483	6.95E-07	2.82E-04	6.287116077
BHLHB2	-0.37512	11.29105	-4.83981	0.00021	0.012267	0.745022998
S100B	-0.37704	7.766454	-7.72363	1.24E-06	3.97E-04	5.73E+00
SLC39A10	-0.38035	9.329431	-3.92528	0.001326	0.035189	-1.054343403
HKDC1	-0.38313	8.32328	-6.78244	5.86E-06	0.001166	4.239991041
EGR4	-0.38704	10.09944	-5.53681	5.49E-05	5.37E-03	2.05967334
NLGN1	-0.38758	8.141828	-5.23886	9.68E-05	0.007298	1.505205019
SLC13A4	-0.3881	7.609667	-8.1569	6.31E-07	2.68E-04	6.38E+00
LOC10004853	-0.39297	8.460216	-5.27421	9.05E-05	7.00E-03	1.571605686
IDH2	-0.41352	9.18773	-4.88619	0.000192	0.011582	0.834340148
GJB6	-0.42057	10.75613	-7.65179	1.39E-06	4.32E-04	5.624296216
TRIM37	-0.43837	11.01737	-3.75484	0.001882	4.40E-02	-1.394449515
1300001I01RII	-0.44782	9.018701	-5.89086	2.85E-05	0.003413	2.702580447
CPNE9	-0.45002	10.14747	-8.49778	3.77E-07	1.81E-04	6.865850547
FOS	-0.45558	9.766477	-9.42288	9.97E-08	6.57E-05	8.11E+00
STARD8	-0.45966	8.988055	-11.1823	1.01E-08	1.21E-05	1.02E+01
ABCC10	-0.46106	7.645496	-5.49058	5.99E-05	5.60E-03	1.97442485
MAP2K1	-0.4638	9.565388	-4.82807	0.000215	0.012349	0.722373419
HUWE1	-0.46833	9.578061	-4.29108	0.00063	0.023441	-0.327520066

NPAS4	-0.47232	8.183955	-9.95446	4.84E-08	3.93E-05	8.78E+00
MARCKSL1	-0.47788	8.354413	-8.71578	2.73E-07	1.37E-04	7.17E+00
R3HDM2	-0.48874	10.63485	-6.3232	1.30E-05	2.05E-03	3.462940906
WFS1	-0.48957	8.924659	-8.8721	2.18E-07	1.17E-04	7.38E+00
XLR4A	-0.49743	8.818481	-10.1084	3.95E-08	3.47E-05	8.965742642
DUSP1	-0.49921	10.59118	-8.85849	2.22E-07	1.17E-04	7.37E+00
SETD3	-0.50505	9.387763	-4.46969	0.000439	0.018249	0.024479836
DDX3X	-0.51656	9.461557	-3.8393	0.001582	3.93E-02	-1.225858492
PTGDS	-0.52181	9.929905	-4.83044	0.000214	0.012349	0.726963173
ARC	-0.59131	10.64455	-11.5867	6.24E-09	1.12E-05	1.06E+01
PDZRN3	-0.70155	9.217246	-13.3037	9.18E-10	4.84E-06	1.23E+01
CUX2	-0.77341	9.049738	-11.5337	6.65E-09	1.12E-05	1.06E+01
TNNC1	-0.78823	9.120732	-12.1935	3.09E-09	8.51E-06	1.12E+01

DEGs in resilient right mPFC

adj.P.Value	< 0.05	
DEGup		64
DEGdown		49

**Gene**	**logFC**	**AveExpr**	**t**	**P.Value**	**adj.P.Val**	**B**
CTGF	0.793198	10.55685	15.16918	1.43E-10	1.51E-06	1.30E+01
HBA-A1	0.674836	12.60395	9.030629	1.73E-07	3.05E-04	7.3706657
SLA	0.621823	8.890923	9.478749	9.23E-08	1.95E-04	7.92E+00
RPRM	0.479928	11.03412	4.976091	0.0001611	2.43E-02	1.0942289
PRSS35	0.459244	8.502893	6.867373	5.07E-06	2.55E-03	4.34E+00
GBP2	0.44877	7.660565	10.03421	4.35E-08	1.15E-04	8.56E+00
ADRA2A	0.432	8.965451	7.40402	2.07E-06	1.56E-03	5.16E+00
SULF1	0.423781	8.768357	8.397036	4.39E-07	6.61E-04	6.55E+00
RAI14	0.417988	8.747604	8.132651	6.55E-07	8.65E-04	6.20E+00
TRF	0.411639	10.38794	5.275327	9.03E-05	1.54E-02	1.64E+00
ZFPM2	0.405182	10.0211	7.191938	2.94E-06	1.82E-03	4.84E+00
COL6A1	0.384902	9.225453	7.757314	1.18E-06	1.13E-03	5.67E+00
PRR18	0.38442	10.29879	4.780717	0.0002364	3.04E-02	7.30E-01
SEMA5A	0.380602	9.76898	6.9653	4.29E-06	2.38E-03	4.4898588
MOG	0.377715	8.901772	7.765192	1.16E-06	1.13E-03	5.68E+00
SPSB1	0.374721	9.264227	5.869069	2.96E-05	8.20E-03	2.69E+00
LOC10004758:	0.368707	9.874884	5.987692	2.38E-05	6.99E-03	2.90E+00
GLRA2	0.353871	9.669872	4.824535	0.0002168	2.82E-02	0.8120262
TLE4	0.352836	10.51575	7.411529	2.05E-06	1.56E-03	5.17E+00
FA2H	0.348365	10.11226	6.125464	1.86E-05	6.13E-03	3.13E+00
AHI1	0.345968	10.57708	5.240892	9.64E-05	1.57E-02	1.580229
DPYSL5	0.345821	7.951066	6.517178	9.27E-06	3.81E-03	3.78E+00
IGTP	0.3445	7.656577	7.488651	1.81E-06	1.56E-03	5.28E+00
RGS9	0.341862	8.980841	5.400942	7.10E-05	1.41E-02	1.87E+00
ENSMUSG000(	0.337438	7.882982	7.270813	2.58E-06	1.81E-03	4.96E+00
D3BWG0562E	0.331868	9.785591	5.572251	5.14E-05	1.18E-02	2.17E+00
ASAH3L	0.325028	7.969307	4.862713	0.0002011	2.76E-02	0.8833785
CNTNAP4	0.319261	9.846148	5.259127	9.31E-05	1.56E-02	1.613348
EVI2A	0.317289	8.523316	5.30804	8.48E-05	1.52E-02	1.7019579
IPO13	0.317218	9.75607	4.920577	0.0001795	2.57E-02	0.9911888
SGK1	0.31711	11.44792	6.506418	9.44E-06	3.81E-03	3.76E+00
NTSR1	0.316493	8.087294	4.607191	0.0003336	3.71E-02	4.03E-01
DRD1A	0.315728	8.898292	5.287474	8.82E-05	1.53E-02	1.6647425
MOBP	0.314054	9.052363	6.54431	8.84E-06	3.81E-03	3.82E+00
CDH13	0.310637	8.439697	7.015229	3.95E-06	2.31E-03	4.57E+00
ANLN	0.306406	8.280857	5.392629	7.22E-05	1.41E-02	1.85E+00
D12ERTD647E	0.303653	9.969361	6.912619	4.69E-06	2.48E-03	4.4078856

RASL10A	0.301617	9.588202	4.668676	0.0002952	3.47E-02	5.19E-01
SMAD3	0.299845	8.05612	4.611293	0.0003309	3.71E-02	0.4104161
PPP1R1B	0.29915	8.97316	5.544954	5.41E-05	1.19E-02	2.1263196
CLDN11	0.293039	12.82258	4.95881	0.0001666	2.48E-02	1.0621944
GAB1	0.291293	9.003693	6.226742	1.55E-05	5.64E-03	3.30E+00
IIGP2	0.291198	7.653213	5.672631	4.26E-05	1.02E-02	2.3516042
LITAF	0.288049	9.402568	5.248401	9.51E-05	1.57E-02	1.593874
RUNX1T1	0.28172	9.781986	5.151164	0.0001146	1.83E-02	1.4165947
TPR	0.278892	9.902227	4.918763	0.0001802	2.57E-02	0.9878152
DDAH1	0.276746	11.28131	4.437087	0.000469	4.66E-02	7.88E-02
RNF113A2	0.265555	9.034929	4.592094	0.0003438	3.71E-02	3.74E-01
HIST1H2AH	0.260939	7.875641	5.596446	4.91E-05	1.15E-02	2.22E+00
PRR7	0.253737	9.384036	4.596214	0.000341	3.71E-02	0.3818286
COL5A1	0.24714	8.052943	5.896618	2.82E-05	8.03E-03	2.7408698
PADI6	0.246058	7.451042	4.591554	0.0003442	3.71E-02	0.3729891
MMP24	0.245668	9.045771	4.401896	0.0005034	4.83E-02	1.14E-02
PACRG	0.239371	9.414803	4.724902	0.000264	3.24E-02	0.6250148
MID1	0.238843	7.855269	5.510788	5.77E-05	1.24E-02	2.0656249
LOC10004172[illegible]	0.228751	11.64068	4.483527	0.0004272	4.33E-02	1.67E-01
NMRAL1	0.22846	8.267957	4.715776	0.0002688	3.26E-02	0.607828
CALCA	0.226201	7.57042	4.539704	0.0003817	4.03E-02	0.2744917
GBP3	0.219568	7.725706	4.522523	0.0003951	4.09E-02	2.42E-01
CARHSP1	0.211707	8.668882	4.642733	0.0003108	3.60E-02	0.4699414
RAMP3	0.20624	8.392446	4.433141	0.0004727	4.66E-02	0.0712353
EG434858	0.204093	8.69901	4.604411	0.0003354	3.71E-02	0.3973715
SLCO2B1	0.2036	8.822654	4.425724	0.0004798	4.69E-02	5.70E-02
CACNG5	0.184057	7.778077	4.668061	0.0002955	3.47E-02	5.18E-01
SLC4A3	-0.18289	9.234296	-4.53358	0.0003864	4.04E-02	2.63E-01
BC048546	-0.19359	9.075915	-4.50581	0.0004085	4.19E-02	2.10E-01
CAPN2	-0.20012	8.271079	-4.38063	0.0005254	4.91E-02	-0.0293031
AI593442	-0.21654	9.778111	-4.39159	0.0005139	4.89E-02	-8.31E-03
LOC100047651	-0.22573	10.44043	-4.84336	0.0002089	2.82E-02	8.47E-01
2510009E07Ri[illegible]	-0.22802	9.425649	-5.55083	5.35E-05	1.19E-02	2.1367459
COX7A1	-0.22956	7.769965	-4.76359	0.0002445	3.07E-02	0.6977648
DDIT4L	-0.23047	8.776336	-4.82748	0.0002156	2.82E-02	0.8175417
HR	-0.24245	8.828072	-5.14171	0.0001167	1.84E-02	1.40E+00
PTN	-0.24949	11.83842	-4.45239	0.0004548	4.57E-02	0.1080362
SYTL2	-0.25312	10.0444	-4.53996	0.0003815	4.03E-02	2.75E-01
KRT12	-0.27379	9.487761	-5.2925	8.74E-05	1.53E-02	1.67E+00
TRPC3	-0.27393	8.770999	-4.92701	0.0001773	2.57E-02	1.0031426
GPC5	-0.27404	8.91444	-5.37398	7.48E-05	1.41E-02	1.8208829
CAMK2A	-0.27575	11.23575	-5.67473	4.24E-05	1.02E-02	2.36E+00
PPM1L	-0.27996	10.43274	-5.39567	7.18E-05	1.41E-02	1.86E+00

GRSF1	-0.28259	10.11151	-5.3737	7.48E-05	1.41E-02	1.8203693
FOS	-0.28386	9.766477	-5.35968	7.68E-05	1.42E-02	1.795152
ATP1B2	-0.28706	10.25857	-4.37991	0.0005262	4.91E-02	-3.07E-02
ARHGAP20	-0.28788	10.02217	-5.12008	0.0001217	1.89E-02	1.359649
STARD8	-0.2989	8.988055	-6.63775	7.51E-06	3.60E-03	3.97E+00
NRN1	-0.3019	12.58454	-5.6862	4.15E-05	1.02E-02	2.38E+00
FAM84A	-0.30251	7.918033	-4.82638	0.000216	2.82E-02	0.8154756
P2RY12	-0.30706	8.52596	-4.40317	0.0005021	4.83E-02	1.39E-02
LOC10004783[illegible]	-0.31183	12.44281	-5.85638	3.03E-05	8.20E-03	2.6715144
SLC24A3	-0.31287	11.68753	-4.7665	0.0002431	3.07E-02	0.7032398
GJB6	-0.32377	10.75613	-5.37736	7.43E-05	1.41E-02	1.8269659
HAPLN4	-0.33218	9.550447	-6.09826	1.95E-05	6.24E-03	3.08E+00
NDRG2	-0.33457	9.481645	-4.67759	0.00029	3.47E-02	0.5358206
FSTL4	-0.33696	8.77555	-5.67222	4.26E-05	1.02E-02	2.35E+00
SORL1	-0.34073	11.37858	-5.04561	0.0001407	2.15E-02	1.22272
CLCN4-2	-0.34105	8.232154	-6.29416	1.37E-05	5.18E-03	3.4125893
NPY	-0.34329	11.02052	-6.16543	1.73E-05	5.89E-03	3.20E+00
SCN4B	-0.34469	9.653842	-5.40156	7.10E-05	1.41E-02	1.87E+00
RORA	-0.34684	8.983488	-5.32865	8.15E-05	1.48E-02	1.7391921
PVALB	-0.347	11.5717	-4.61925	0.0003257	3.71E-02	0.425486
CPNE9	-0.35127	10.14747	-6.05526	2.11E-05	6.55E-03	3.01E+00
ARC	-0.36275	10.64455	-6.48872	9.74E-06	3.81E-03	3.73E+00
SLC7A10	-0.36398	8.636367	-6.58293	8.26E-06	3.79E-03	3.88E+00
RGS10	-0.37352	9.908404	-6.1793	1.69E-05	5.89E-03	3.22E+00
LOC100046841	-0.39754	8.855368	-4.7525	0.00025	3.10E-02	0.6769309
PDZRN3	-0.41786	9.217246	-7.23367	2.74E-06	1.81E-03	4.90E+00
SCN1A	-0.43243	10.25208	-5.98921	2.38E-05	6.99E-03	2.8995337
GLRX2	-0.4414	8.893965	-5.719	3.91E-05	1.02E-02	2.4328132
HSD11B1	-0.45154	9.321022	-10.6215	2.03E-08	7.16E-05	9.19E+00
SRR	-0.45635	9.461644	-4.86224	0.0002013	2.76E-02	0.8824878
DUSP1	-0.4823	10.59118	-7.81277	1.08E-06	1.13E-03	5.75E+00
MAP2K1	-0.51183	9.565388	-4.86385	0.0002007	2.76E-02	0.885508
NPAS4	-0.66471	8.183955	-12.7887	1.59E-09	8.42E-06	1.12E+01
