# Supplementary material for: Molecular laterality encodes stress susceptibility in the medial prefrontal cortex
Source: Mol Brain. 2021 Jun 14;14:92. doi: 10.1186/s13041-021-00802-w (PMC8201740; doi:10.1186/s13041-021-00802-w)
Supplement: Supplementary file 4 — Additional file 4: Table S2. List of 526 DEGs with FDR adjusted p-value cutoffs of 0.05 corresponding to genes presented in heatmap format in Fig. 1b according to their log2L/R values. [file 13041_2021_802_MOESM4_ESM.pdf]

Supplementary table 2
List of 526 DEGs with FDR adjusted *p*-value cutoffs of 0.05 corresponding to genes presented in heatmap format in Figure 1b according to their $\log_2$ L/R values.

Heatmap

Adj.P.Val < 0.05 (n=526)

**Gene**	**Log2(L/R) Sl_vs_Sr**	**Log2(L/R) Cl_vs_Cr**	**Log2(L/R) Rl_vs_Rr**
CUX2	0.93	-0.06	0.17
WFS1	0.83	-0.12	0.24
TNNC1	0.78	-0.09	-0.05
STARD8	0.57	-0.08	-0.01
CAR4	0.56	-0.12	-0.10
PDZRN3	0.54	-0.14	-0.13
VIP	0.52	-0.01	0.01
SLC13A4	0.52	-0.17	0.09
DDIT4L	0.51	0.01	0.04
IGF2	0.51	-0.21	0.09
EGR2	0.50	0.08	-0.11
EVC2	0.49	-0.02	-0.03
CDKN1C	0.49	-0.10	0.10
CPNE6	0.48	-0.02	-0.03
EGR4	0.48	0.01	0.00
CPNE4	0.47	-0.06	0.01
DCN	0.47	-0.13	-0.08
LOC10004	0.45	0.00	-0.08
BHLHB2	0.43	-0.10	-0.15
PVRL3	0.42	-0.01	0.04
TIAM1	0.41	-0.00	-0.05
RILPL1	0.39	-0.08	0.03
BTBD3	0.38	0.17	0.21
JUNB	0.38	0.02	0.01
PALMD	0.38	-0.03	0.06
CPNE9	0.36	-0.18	-0.01
DACT2	0.36	-0.08	-0.02
LPL	0.36	0.01	-0.10
LOC10004	0.36	-0.21	-0.04
C1QTNF4	0.36	-0.26	-0.03
RELN	0.36	-0.04	-0.09

RIMS3	0.36	-0.15	0.18
GRASP	0.35	-0.04	-0.03
SPAG5	0.35	-0.05	-0.09
MARCKSL1	0.35	-0.08	0.05
RREB1	0.35	-0.04	-0.02
MGP	0.35	-0.15	0.14
PRELP	0.35	-0.10	0.09
HPCA	0.34	-0.19	0.04
OGN	0.34	-0.11	-0.01
PDYN	0.34	-0.08	-0.10
D8ERTD82	0.34	0.01	-0.03
IGSF3	0.33	-0.04	0.02
BHLHB5	0.33	-0.07	-0.15
HBA-A1	0.33	0.05	0.03
HKDC1	0.33	-0.09	-0.07
GJB2	0.33	-0.06	-0.04
GCNT2	0.33	-0.03	-0.06
6030405A1	0.33	-0.16	-0.03
EFCAB1	0.33	-0.04	0.01
LYZ	0.33	-0.06	0.25
FOSB	0.32	-0.07	0.02
GJB6	0.32	-0.07	-0.02
SLC39A10	0.32	-0.00	-0.05
SLC6A13	0.32	-0.06	-0.04
CITED4	0.31	-0.01	0.05
FAM148C	0.31	-0.26	0.06
RTN4RL1	0.31	-0.09	-0.07
LOC10004	0.30	-0.16	0.00
COCH	0.30	0.12	-0.14
FSTL4	0.30	-0.15	0.13
OTOF	0.30	0.02	-0.05
PLCH2	0.29	-0.21	-0.00
2510009E0	0.29	-0.12	0.08
KRT12	0.29	0.01	0.01
PKNOX2	0.29	-0.30	-0.21
CACNG3	0.29	-0.22	-0.11
SLC7A11	0.29	-0.04	0.03
BDNF	0.29	-0.01	0.03
ECHDC2	0.29	-0.06	0.03
MEF2C	0.29	0.17	0.03
TLE1	0.28	0.01	0.08
ZIC1	0.28	0.01	-0.05
FOS	0.28	0.08	-0.05

DUSP6	0.28	0.04	-0.01
PER2	0.27	-0.03	-0.11
MFGE8	0.27	-0.05	0.20
PER1	0.27	-0.05	-0.15
CDH8	0.27	-0.02	-0.02
NBL1	0.27	-0.14	0.12
NEURL	0.27	0.03	0.03
ARC	0.27	0.08	0.16
GUCY1A3	0.27	0.00	-0.03
SERPINF1	0.27	-0.00	0.06
TGM2	0.27	0.04	-0.07
1300013J1	0.27	-0.10	-0.03
PTPRK	0.27	-0.07	-0.01
ADCYAP1	0.27	-0.12	0.10
DBP	0.27	0.12	-0.00
SLC2A1	0.27	-0.08	-0.06
FMO1	0.27	-0.05	0.01
LOC10004	0.27	-0.03	-0.19
VGF	0.26	-0.24	-0.03
VASN	0.26	-0.01	-0.07
HAP1	0.26	-0.10	0.07
KCTD4	0.26	-0.04	0.05
2810405K(	0.26	0.02	-0.05
NR2F6	0.26	-0.12	0.07
LINGO2	0.26	0.02	0.04
LOC10004	0.26	-0.07	0.01
HSPA2	0.26	-0.10	0.01
UACA	0.26	-0.02	0.15
PRKG2	0.26	-0.02	0.09
CHST1	0.26	0.03	0.01
S100A11	0.25	-0.08	0.02
TMCC2	0.25	-0.13	-0.04
ODZ4	0.25	0.12	-0.09
RANBP3L	0.25	0.03	0.02
S100A8	0.25	0.14	0.36
SLC4A3	0.25	-0.12	-0.03
CCK	0.25	-0.00	-0.01
ANXA11	0.25	-0.12	0.01
EPHX1	0.24	-0.05	0.09
ACSL5	0.24	-0.10	0.14
BMP1	0.24	-0.12	0.01
CACNA1H	0.24	-0.12	0.05
VAT1L	0.24	-0.06	-0.22

FBF1	0.24	-0.02	0.07
WASF1	0.24	0.08	-0.05
ANKRD6	0.24	-0.05	0.01
DUSP1	0.23	0.16	0.19
ZFP148	0.23	-0.03	0.00
SYNE1	0.23	0.13	-0.03
2810022LC	0.23	-0.03	0.05
CALB2	0.23	-0.05	0.03
FCHO1	0.23	-0.02	0.07
EFNA5	0.23	0.03	0.11
PNCK	0.23	-0.05	-0.01
DARC	0.23	-0.02	0.08
ARNTL	0.23	-0.14	-0.15
CDH7	0.23	0.00	-0.06
OSBP2	0.23	0.03	0.02
AUTS2	0.22	0.01	0.12
C1QC	0.22	-0.01	-0.01
CBLN4	0.22	-0.07	-0.07
NPY1R	0.22	-0.07	-0.04
DOCK4	0.22	-0.02	0.02
DUSP18	0.22	-0.02	0.11
ARSJ	0.22	0.03	-0.02
ACCN2	0.22	-0.06	-0.02
CDC42EP4	0.22	0.04	0.10
KLF2	0.22	-0.08	-0.01
TRIB2	0.22	-0.07	0.13
CLCN2	0.22	-0.08	0.01
DKKL1	0.22	0.01	-0.02
HDAC11	0.22	-0.02	0.17
SCCPDH	0.22	-0.00	0.05
SLC26A4	0.22	0.01	-0.03
ITPKA	0.22	-0.07	0.10
RNF144A	0.22	-0.03	-0.16
ANXA2	0.22	-0.07	-0.06
SLC24A3	0.22	-0.07	-0.01
SLC2A13	0.22	-0.08	-0.04
RNF19A	0.22	0.03	0.04
RBP1	0.21	0.08	0.03
CASP1	0.21	0.01	-0.01
LYZ2	0.21	0.02	0.06
PPM2C	0.21	0.08	-0.07
TNFRSF19	0.21	0.06	0.04
DLK2	0.21	-0.03	-0.01

SCHIP1	0.21	-0.06	-0.03
2310021P1	0.21	-0.14	-0.00
ZDHHC14	0.21	-0.08	0.00
TRPC7	0.21	-0.01	0.02
BC067047	0.21	0.05	0.04
ERDR1	0.21	-0.03	0.04
ZBTB7C	0.21	-0.06	-0.00
COBL	0.21	-0.01	0.10
FHL1	0.21	-0.08	0.04
B930076A(	0.21	-0.26	-0.03
CD83	0.20	-0.12	-0.10
SYT17	0.20	0.07	-0.04
CAMK2A	0.20	-0.09	0.16
MARCH4	0.20	-0.05	-0.01
AKAP8L	0.20	-0.15	0.06
SERGEF	0.20	-0.05	0.07
PSME1	0.20	-0.03	0.03
4933439C2	0.20	-0.24	-0.01
EXTL3	0.20	-0.08	0.04
B3GNT8	0.20	0.00	0.03
NPTX2	0.20	-0.07	0.13
MYOC	0.20	0.02	-0.05
MEGF9	0.20	0.40	0.12
IFITM3	0.20	-0.07	0.22
AW049604	0.20	0.04	0.04
SLC1A3	0.20	-0.03	0.09
5330439J0	0.20	0.03	0.07
DNAJB1	0.20	0.02	0.16
CAMTA2	0.19	-0.06	0.08
ADAMTSL2	0.19	0.02	0.01
MMD	0.19	-0.03	-0.11
ACTA2	0.19	0.00	-0.08
WNT4	0.19	-0.07	0.01
GM129	0.19	0.04	-0.06
HEY1	0.19	0.03	-0.04
LOC10004	0.19	-0.02	-0.03
EXDL2	0.19	-0.07	-0.02
MAPK4	0.19	0.05	-0.02
RHBDL3	0.19	-0.08	0.04
SYTL2	0.19	-0.10	0.09
4930511J1	0.19	-0.04	0.04
BOK	0.19	-0.06	0.06
NAPEPLD	0.19	-0.06	-0.09

CGGBP1	0.19	-0.01	0.02
AGGF1	0.19	0.04	-0.04
RXFP3	0.19	-0.11	-0.08
FKBP1A	0.19	-0.02	-0.08
4930544G2	0.18	-0.04	0.05
EHMT2	0.18	-0.11	0.01
ARPP21	0.18	-0.03	-0.01
GTPBP6	0.18	-0.08	0.02
SEPT5	0.18	-0.02	0.04
IGFBP2	0.18	0.00	0.01
OTOP2	0.18	-0.01	0.04
FAM13C	0.18	-0.07	-0.05
GPR83	0.18	0.05	0.05
AI593442	0.18	-0.08	0.06
BC030499	0.18	0.03	0.08
PCTK1	0.18	-0.00	0.14
SULT1A1	0.18	-0.09	0.01
WDR60	0.18	0.03	-0.13
KHDRBS2	0.18	0.02	-0.01
NAV1	0.18	-0.08	-0.03
TPD52L1	0.17	-0.02	0.04
CAMKK1	0.17	-0.00	-0.07
UBQLN4	0.17	-0.10	0.06
YWHAZ	0.17	-0.03	0.04
NOL4	0.17	-0.06	-0.15
MORN4	0.17	-0.08	0.18
MAPRE2	0.17	-0.14	-0.11
BC064033	0.17	0.02	0.04
ETS1	0.17	-0.02	-0.06
ST8SIA2	0.17	0.03	-0.08
MID1	0.17	-0.01	-0.11
JOSD1	0.17	-0.01	0.17
LOC10004	0.17	0.04	0.08
CSF1R	0.17	-0.04	0.07
RYR1	0.17	-0.07	-0.05
PROSAPIP1	0.17	-0.11	0.10
CYP1B1	0.17	-0.02	-0.03
TCF4	0.17	-0.01	-0.06
LOC10004	0.17	-0.05	0.01
NPM3-PS1	0.17	0.08	0.27
MKX	0.17	-0.02	-0.06
NGFRAP1	0.16	0.09	0.12
RGS6	0.16	0.01	-0.01

BCL9L	0.16	0.02	-0.02
ZBTB8B	0.16	-0.05	0.17
TBC1D13	0.16	-0.05	0.04
H47	0.16	-0.00	0.07
COTL1	0.16	-0.02	0.13
HIST2H3B	0.16	0.06	-0.04
RGL1	0.16	-0.09	-0.03
ZFP810	0.16	-0.07	0.04
HIST1H2A(	0.16	0.04	0.19
EFEMP2	0.16	-0.01	0.00
EMID2	0.16	-0.10	0.04
PDE4D	0.16	-0.03	0.06
CIB2	0.16	-0.04	0.10
CACNB3	0.15	-0.09	0.03
RGS4	0.15	0.05	-0.08
FBXL10	0.15	-0.10	-0.01
AB112350	0.15	0.04	-0.00
AGFG1	0.15	0.06	-0.01
D930015E(	0.15	-0.08	0.10
KCNK6	0.15	-0.01	0.05
ANXA3	0.15	-0.02	0.01
ARHGAP2(	0.15	-0.02	-0.01
OLFML3	0.15	-0.06	0.12
6330406I1	0.15	-0.04	-0.13
MT3	0.15	0.07	0.17
SAP130	0.15	-0.06	-0.05
THBD	0.15	-0.10	-0.07
D930028F'	0.14	0.00	-0.02
PRR13	0.14	-0.08	0.01
TCF19	0.14	0.03	-0.02
RASGRF2	0.14	0.05	-0.06
FLT1	0.14	-0.20	-0.14
CHN2	0.14	0.03	-0.15
B2M	0.14	-0.03	0.10
S100A6	0.14	-0.02	0.15
HRASLS	0.14	0.10	0.08
IGSF9	0.14	-0.06	-0.02
SCN3B	0.14	-0.04	-0.00
HSD11B1	0.14	0.03	0.11
CPNE8	0.14	0.03	-0.08
CAMK1G	0.14	-0.07	0.03
SPHK1	0.14	-0.02	0.07
EPB4.9	0.14	-0.06	-0.03

RASGRF1	0.13	-0.16	-0.04
ATG16L1	0.12	0.00	0.07
FBXO32	-0.13	-0.00	0.02
EG434858	-0.14	0.01	-0.13
COL5A1	-0.14	-0.11	0.01
DCLK1	-0.14	-0.11	-0.05
ADAMTSL4	-0.14	-0.01	-0.13
MIF4GD	-0.14	-0.07	-0.02
PHLDA3	-0.14	-0.03	0.04
DIAP3	-0.15	0.05	0.04
INPPL1	-0.15	0.02	0.04
INPP4B	-0.15	-0.03	-0.03
FIGN	-0.15	0.08	-0.03
FAM132B	-0.15	0.02	0.01
AB182283	-0.15	-0.01	0.02
MTMR2	-0.15	-0.03	-0.04
ATP8B1	-0.15	0.06	-0.03
CHRM2	-0.15	0.10	0.06
RAB5A	-0.15	0.01	-0.19
SEMA3F	-0.15	-0.00	0.00
MYO5B	-0.15	0.13	-0.13
PIGZ	-0.16	0.12	0.02
HS3ST2	-0.16	-0.02	0.10
SDK1	-0.16	0.01	0.08
ABCA8A	-0.16	-0.01	-0.08
ODZ3	-0.16	0.14	0.02
CLCN4-2	-0.16	-0.11	0.01
EPHA7	-0.16	0.01	-0.13
ELOVL6	-0.16	0.10	-0.08
INSC	-0.17	0.08	0.01
WHRN	-0.17	0.00	0.02
HSD17B11	-0.17	-0.01	-0.09
TAC1	-0.17	-0.07	0.02
FUT9	-0.17	0.26	-0.02
LGI2	-0.17	0.14	0.09
THAP3	-0.17	0.09	0.07
STK4	-0.17	0.07	-0.01
RAMP3	-0.17	-0.10	0.01
SLC7A4	-0.17	-0.05	-0.11
CDC42EP2	-0.17	0.09	0.09
6330503K2	-0.18	0.01	-0.06
OG9X	-0.18	-0.02	-0.01
LMO3	-0.18	0.05	0.08

ELAVL2	-0.18	0.07	-0.06
OLIG1	-0.18	0.06	-0.05
RAB3B	-0.18	-0.06	-0.06
RYR3	-0.18	-0.18	-0.10
DYNC1LI2	-0.18	0.02	0.01
NFE2L3	-0.18	0.12	-0.09
DCBLD1	-0.18	0.07	-0.02
CHRNA5	-0.18	-0.02	-0.05
PIGP	-0.18	0.02	-0.14
GRB7	-0.18	-0.06	-0.01
TMEM63A	-0.18	0.04	-0.03
GRIK1	-0.18	0.12	0.04
PRDX3	-0.18	-0.02	0.05
TTC19	-0.18	-0.02	-0.06
AI314976	-0.18	0.02	0.01
WNT2	-0.18	-0.09	-0.00
TRIM36	-0.18	-0.01	-0.06
ASAH2	-0.18	0.02	-0.07
STAC2	-0.18	0.12	-0.06
PRSS23	-0.19	-0.06	-0.06
OPRK1	-0.19	0.11	0.12
GFM1	-0.19	-0.06	0.03
H2AFY	-0.19	-0.04	-0.01
TMEM19	-0.19	-0.01	-0.06
LRRTM2	-0.19	0.08	-0.08
SPOP	-0.19	-0.11	0.05
CADPS2	-0.19	0.06	-0.06
OLFML2B	-0.19	-0.00	-0.07
TMEM38A	-0.20	-0.11	-0.05
TMEM125	-0.20	0.01	0.10
SERINC2	-0.20	-0.08	-0.02
RPH3A	-0.20	-0.10	0.05
THSD7B	-0.20	-0.03	0.00
SLC35F3	-0.20	0.24	0.09
CAR14	-0.20	-0.02	-0.14
IL11RA1	-0.20	0.15	-0.12
NDST1	-0.21	-0.01	0.03
CD47	-0.21	0.05	0.07
PLLP	-0.21	0.12	0.20
SAMD9L	-0.21	-0.00	0.17
1700019D(	-0.21	0.05	-0.02
DBNDD2	-0.21	0.14	0.10
CD82	-0.21	0.03	-0.01

AGAP1	-0.21	0.09	-0.08
ATP5F1	-0.21	0.04	-0.20
FGF10	-0.21	0.03	0.01
HAPLN2	-0.21	-0.05	0.07
RNMT	-0.22	0.06	-0.03
REEP5	-0.22	-0.19	0.14
ZYX	-0.22	-0.02	0.02
ARSG	-0.22	0.11	0.03
AGPAT4	-0.22	0.05	0.10
ADORA1	-0.22	0.02	0.02
H2-T23	-0.22	0.05	-0.02
PLEKHG3	-0.22	0.00	-0.01
TTYH2	-0.22	0.01	0.04
PLXNB3	-0.22	0.04	0.12
SOX5	-0.22	0.14	0.04
EFHD1	-0.22	0.10	0.00
CPLX2	-0.22	0.34	0.06
PHLDB1	-0.22	-0.09	-0.07
PBRM1	-0.22	0.08	-0.13
DOK4	-0.22	0.03	0.05
3110035E1	-0.22	-0.07	-0.07
PRICKLE1	-0.22	-0.06	-0.09
NEU2	-0.23	-0.00	-0.07
NXPH2	-0.23	0.01	-0.02
VSTM2B	-0.23	-0.01	-0.11
NETO1	-0.23	0.27	0.06
NMRAL1	-0.23	0.10	0.02
NIPA1	-0.23	0.10	-0.15
ENSMUSG	-0.23	0.10	0.02
C130090K	-0.23	0.05	-0.10
KLF7	-0.23	-0.05	-0.04
INA	-0.23	-0.03	0.15
TRIM59	-0.23	0.08	-0.13
RAPGEF4	-0.23	-0.07	-0.02
PACS2	-0.23	-0.07	0.03
SCD1	-0.23	-0.06	0.18
GPR21	-0.24	0.00	0.04
TPBG	-0.24	0.08	-0.06
RASL11B	-0.24	0.08	0.12
PADI6	-0.24	0.00	0.04
ANO4	-0.24	0.20	0.07
LY6G6E	-0.24	0.05	-0.05
FOXN3	-0.24	0.07	0.09

LRRN1	-0.24	0.07	-0.12
SLAIN1	-0.24	-0.03	-0.05
PPP1R14A	-0.24	0.10	0.00
SKP1A	-0.24	-0.05	-0.09
PDLIM2	-0.24	0.07	0.11
KIF5A	-0.25	-0.06	-0.01
RELL1	-0.25	-0.04	-0.12
DDAH1	-0.25	0.09	0.05
UAP1	-0.26	-0.09	-0.02
HTR1F	-0.26	0.17	0.02
FAM134B	-0.26	0.02	-0.06
PCBP2	-0.26	-0.10	-0.04
VANGL2	-0.26	0.02	-0.01
OGFRL1	-0.26	0.08	-0.18
NTSR1	-0.26	-0.04	0.02
LRFN2	-0.26	0.02	-0.03
OSBPL9	-0.26	-0.04	-0.07
ADAMTS4	-0.26	0.03	0.08
CARHSP1	-0.26	0.10	0.03
CRTAC1	-0.27	-0.10	0.06
GADD45A	-0.27	0.06	-0.06
GLTP	-0.27	0.11	-0.14
D12ERTD6	-0.27	0.07	0.00
PIK3R3	-0.27	-0.00	-0.05
BCL11B	-0.27	0.04	0.01
LOC10004	-0.28	0.02	-0.09
LOC10004	-0.28	0.08	-0.01
DRD1A	-0.28	0.11	0.03
RIMS1	-0.28	0.08	0.01
RHOG	-0.28	-0.02	0.02
KCNK13	-0.29	0.15	0.03
NFIB	-0.29	0.07	-0.06
NOS1AP	-0.29	0.21	0.04
ITGB4	-0.30	0.02	0.10
NRIP3	-0.30	-0.07	-0.06
PLEKHA2	-0.30	0.15	-0.03
GAB1	-0.30	0.04	-0.03
IL33	-0.31	0.07	0.08
LPGAT1	-0.31	0.25	-0.10
UNC5B	-0.31	0.06	-0.02
PEBP1	-0.31	0.00	-0.04
ADSSL1	-0.31	0.05	0.11
PDLIM1	-0.31	-0.04	0.05

FANCD2	-0.31	0.02	0.13
COL6A1	-0.32	-0.21	-0.06
NRP1	-0.32	0.08	0.03
LOC10004[illegible]	-0.32	0.00	-0.12
RGS9	-0.32	-0.07	0.12
GAL3ST1	-0.33	0.01	0.02
MYL4	-0.33	0.17	0.08
LOC10004[illegible]	-0.33	-0.02	-0.00
SST	-0.33	-0.03	0.04
LASS2	-0.33	0.09	-0.01
TMEM178	-0.34	-0.01	0.05
GARNL3	-0.34	0.06	0.03
SLC44A1	-0.34	0.08	0.13
CNTNAP4	-0.34	-0.02	-0.12
KCNA1	-0.34	0.19	-0.01
OPALIN	-0.34	0.25	0.04
ZDHHC9	-0.34	-0.04	0.11
ARHGAP2[illegible]	-0.35	0.01	0.05
LOC10004[illegible]	-0.36	0.10	-0.08
SC4MOL	-0.36	0.09	-0.10
CNP	-0.37	0.13	0.19
NDRL	-0.37	-0.04	-0.05
PPP1R1B	-0.37	0.07	0.05
RAI14	-0.38	0.04	-0.06
PLP1	-0.38	0.10	-0.02
TLE4	-0.38	0.16	-0.11
EVI2A	-0.38	0.21	-0.04
LITAF	-0.39	0.12	0.02
OTX1	-0.39	-0.04	0.04
IGSF21	-0.41	-0.05	0.00
NEFM	-0.42	0.16	-0.15
ISLR2	-0.42	-0.15	-0.06
EDG2	-0.43	-0.05	0.00
ENPP6	-0.43	-0.06	-0.01
NDRG1	-0.45	0.09	-0.24
CRYAB	-0.46	0.13	0.02
GLRA2	-0.46	0.10	-0.10
SPSB1	-0.46	0.02	-0.01
TSPAN2	-0.46	0.14	-0.12
ANLN	-0.46	0.11	0.08
GJC2	-0.48	0.15	0.03
SLA	-0.48	0.08	-0.02
QDPR	-0.48	0.02	-0.04

PLEKHB1	-0.49	0.15	0.15
ZFPM2	-0.50	0.16	-0.06
SCN4B	-0.51	0.05	0.22
MOBP	-0.52	0.12	0.02
PRSS35	-0.53	0.06	-0.19
UGT8A	-0.53	0.12	-0.07
ADRA2A	-0.53	0.03	-0.06
MAG	-0.55	0.09	0.13
MOG	-0.57	0.18	-0.03
SULF1	-0.57	0.22	-0.05
MBP	-0.58	0.13	0.09
SEMA5A	-0.59	0.13	0.01
MAL	-0.59	0.17	0.14
CLDN11	-0.60	0.15	0.07
FA2H	-0.62	0.16	0.06
BCAS1	-0.68	0.11	-0.01
PRR18	-0.68	0.22	0.09
TRF	-0.73	0.04	-0.08
RPRM	-0.83	0.12	0.06
CTGF	-0.90	0.14	0.06
GBP2	0.01	0.03	0.31
ACTR1A	0.10	0.06	0.31
