# Supplementary material for: Molecular laterality encodes stress susceptibility in the medial prefrontal cortex
Source: Mol Brain. 2021 Jun 14;14:92. doi: 10.1186/s13041-021-00802-w (PMC8201740; doi:10.1186/s13041-021-00802-w)
Supplement: Supplementary file 5 — Additional file 5: Table S3. Comparison of averaged expression values of DEGs between the two mPFC hemispheres, presented in dot plot format in Fig. 1c. [file 13041_2021_802_MOESM5_ESM.pdf]

Supplementary table 3
Comparison of averaged expression values of DEGs between the two mPFC hemispheres, presented in dot plot format in Figure 1c.

Gene	Sl	Sr	Rl	Rr	Cl	Cr
HBA-A1	9004.78	7139.99	6573.75	6429.38	4170.86	4017.78
WFS1	636.88	359.09	544.84	460.72	464.48	503.89
MGP	397.86	312.33	360.79	327.52	300.51	332.58
IGF2	859.29	602.64	776.51	733.07	653.42	753.84
CPNE6	3457.90	2476.79	3022.96	3087.09	2665.37	2700.06
FAM148C	513.18	412.98	505.94	486.39	407.04	486.81
CDKN1C	400.88	286.08	399.14	372.37	319.54	343.45
DACT2	420.47	328.05	337.05	341.63	335.79	354.51
PROSAPIP'	1545.09	1376.90	1424.80	1327.23	1234.86	1333.12
CAR4	611.67	414.98	435.33	469.37	491.73	533.43
LPL	659.81	514.82	562.84	603.48	530.25	525.96
MFGE8	3093.09	2558.61	3089.21	2691.01	2493.19	2575.32
LYZ	357.61	285.58	368.17	309.64	288.45	301.00
CACNA1H	902.57	762.13	882.24	852.92	729.43	792.51
COTL1	871.80	780.38	901.66	824.94	707.31	716.61
SLC13A4	226.70	158.52	210.31	198.08	184.62	207.45
HAP1	921.57	766.71	940.10	895.60	750.22	804.08
C1QTNF4	1867.29	1456.32	1598.88	1627.79	1521.10	1823.07
CPNE4	1235.79	891.01	1065.19	1056.10	1006.05	1050.61
HPCA	5482.02	4333.23	4540.31	4422.58	4483.48	5117.07
NR2F6	466.73	389.97	448.15	425.53	382.37	416.20
ANXA2	337.74	290.59	313.36	326.63	276.52	290.87
TIAM1	1561.88	1181.14	1354.32	1401.35	1280.84	1284.18
IFITM3	439.24	382.89	503.33	432.82	360.61	379.23
GRASP	807.56	632.01	697.29	710.88	663.14	681.49
FOSB	380.44	303.86	334.97	331.33	312.79	329.33
IGSF3	397.65	315.38	346.93	341.26	327.59	335.90
PNCK	747.86	636.53	699.76	705.28	617.07	638.44
ECHDC2	394.91	323.20	359.44	351.50	326.32	341.34
RILPL1	991.23	754.84	852.88	837.43	819.39	864.34
DCN	267.53	193.32	230.77	243.27	222.26	242.52
S100A11	270.78	227.03	256.30	253.60	225.61	237.69
ACTR1A	2008.71	1879.70	2038.58	1646.05	1673.46	1608.71
CACNB3	544.65	489.05	522.69	512.73	453.85	482.20
ITPKA	2665.16	2291.78	2239.04	2090.63	2225.62	2334.75
FCHO1	997.29	846.82	935.30	892.29	832.22	843.72

PALMD	460.44	355.03	424.84	408.31	386.15	393.91
PTPRK	436.86	362.89	376.70	380.17	367.22	385.64
SLC2A1	1081.42	898.07	1026.93	1070.73	908.90	960.17
FLT1	1114.86	1010.05	957.02	1052.85	939.65	1076.36
1300013J1	194.63	161.60	160.71	164.25	164.38	176.04
KCTD4	390.08	324.84	364.29	351.24	330.64	341.00
MID1	250.33	222.37	235.08	253.25	212.91	214.64
D8ERTD82	259.37	205.26	217.53	222.10	220.59	219.11
EVC2	336.35	239.25	282.87	288.92	286.16	290.82
AKAP8L	1131.26	984.73	1132.00	1089.84	963.29	1070.16
NBL1	515.52	427.22	514.90	473.99	440.09	484.65
COL6A1	546.53	679.91	680.68	707.27	466.82	542.01
PKNOX2	535.46	437.44	471.00	543.15	457.19	562.15
CACNG3	498.43	407.63	421.78	455.73	426.16	496.94
LOC10004(	3869.00	3446.71	3851.35	3817.02	3310.83	3415.82
MORN4	403.05	357.88	408.63	360.29	345.78	366.17
COL5A1	261.65	287.66	289.49	287.19	224.44	241.94
FHL1	1398.92	1213.38	1344.78	1308.09	1200.32	1272.11
OGN	189.75	150.01	163.49	164.83	163.05	175.27
RAMP3	332.38	374.43	356.36	353.76	285.57	306.50
UACA	277.72	232.17	269.31	242.34	238.64	242.16
ACSL5	2885.33	2438.19	2597.07	2360.13	2478.86	2662.19
ATG16L1	1207.75	1107.77	1193.42	1133.32	1043.21	1041.95
GTPBP6	307.24	270.66	289.76	286.58	265.44	280.95
LOC10004!	6775.61	5282.22	5764.85	5923.94	5857.14	6789.89
UBQLN4	1016.45	901.18	953.04	915.79	881.15	947.04
PER2	1090.35	901.14	1045.01	1127.26	947.86	964.00
SPAG5	314.14	246.27	280.47	297.88	272.63	282.99
VIP	1681.82	1168.52	1389.54	1379.17	1458.94	1469.57
ACCN2	836.83	716.93	789.30	801.24	726.89	757.42
FKBP1A	2268.29	1995.29	1971.99	2078.19	1978.81	2011.93
CITED4	282.69	227.32	233.70	225.02	246.64	247.76
STARD8	619.35	416.32	462.30	465.39	540.45	572.35
PSME1	532.34	464.04	510.63	500.09	464.89	476.32
LOC10004	1562.29	1268.09	1312.67	1308.64	1366.65	1530.32
EXDL2	1403.19	1230.91	1314.97	1336.57	1238.63	1296.21
GJB2	206.14	163.67	192.04	197.90	182.02	189.89
WNT4	237.90	208.40	216.97	215.09	210.09	220.11
SCN3B	330.87	300.33	320.64	321.26	292.44	300.97
SERPINF1	284.71	236.30	274.03	263.76	251.91	252.71
DDIT4L	530.07	371.60	408.85	398.32	469.42	466.97
ARSJ	202.37	173.43	193.88	197.11	179.86	175.93
NAPEPLD	266.16	233.65	239.94	254.52	236.77	246.73

BOK	571.53	501.61	492.21	472.13	508.89	529.87
CIB2	543.34	487.72	534.59	497.50	485.18	497.04
PVRL3	304.57	228.25	256.54	249.95	271.93	273.23
MAPRE2	4564.66	4051.41	4169.19	4498.23	4086.93	4506.17
ZBTB8B	260.45	232.98	255.35	226.84	233.24	241.20
ZFP810	329.91	295.22	292.35	283.82	296.29	311.88
BMP1	305.92	258.64	278.23	276.64	275.04	297.92
2510009E(	761.94	622.34	674.26	639.35	690.48	748.86
INSC	173.85	195.17	199.02	197.46	193.41	182.50
EPHA7	310.34	347.83	320.34	350.18	346.14	343.67
PRSS23	159.24	181.10	168.13	175.15	177.92	185.15
CARHSP1	361.43	434.25	445.88	437.46	404.25	377.76
PLXNB3	173.31	201.60	206.53	190.09	194.30	188.40
HSD11B1	663.69	602.69	580.99	538.40	752.13	736.22
LITAF	578.49	756.67	751.14	743.03	663.16	608.54
ELAVL2	1512.36	1711.52	1674.79	1748.19	1735.32	1656.58
SLC35F3	1397.83	1606.84	1600.29	1505.68	1605.09	1356.94
DCBLD1	275.50	311.95	298.67	303.36	317.00	302.92
TLE4	1261.39	1642.11	1542.38	1660.51	1454.58	1300.20
6330503K2	724.54	817.55	776.74	810.20	835.32	827.56
PPP1R14A	205.30	242.60	243.11	243.00	237.57	221.89
CPLX2	1683.48	1963.78	1673.02	1602.11	1947.29	1539.37
PLLP	314.27	363.02	379.33	330.44	363.92	334.40
ARSG	198.96	231.25	232.82	227.47	231.20	214.77
PEBP1	704.21	873.29	741.52	759.65	818.22	818.25
PIGZ	300.59	334.86	318.83	314.91	349.88	322.28
ANLN	257.11	353.69	362.79	342.89	298.78	277.12
CADPS2	2026.61	2316.18	2173.82	2273.78	2377.73	2273.52
RHOG	223.52	272.07	263.93	260.65	262.60	265.33
RELL1	323.97	384.58	338.12	366.85	383.52	395.69
SCD1	1289.77	1518.31	1541.48	1357.45	1530.71	1601.19
CLCN4-2	274.95	307.93	281.29	278.90	326.73	352.97
LOC10004	235.68	294.14	259.79	282.04	280.23	280.22
FOS	891.64	735.89	802.21	828.73	1069.21	1008.90
PBRM1	494.48	576.55	535.29	585.97	594.42	560.81
INA	1358.49	1594.56	1514.18	1358.54	1640.97	1673.39
SLC44A1	286.47	362.21	373.04	341.66	346.31	327.84
SULF1	354.50	526.45	476.18	494.61	429.28	368.48
PCBP2	1518.99	1819.35	1764.92	1809.22	1841.92	1980.65
LOC10004	362.56	456.54	379.05	379.78	440.24	446.82
KCNK13	258.44	315.14	313.06	307.59	314.99	283.91
SKP1A	1833.51	2157.53	1996.46	2131.92	2233.09	2311.41
EVI2A	310.35	405.69	399.82	410.03	380.46	328.90

NETO1	455.06	532.60	501.25	480.93	557.84	463.93
KIF5A	2560.76	3037.25	3145.25	3165.43	3143.54	3287.11
FAM134B	1896.91	2262.22	2134.50	2231.58	2338.66	2313.83
ADAMTS4	205.40	246.10	249.68	235.06	254.46	249.55
MYL4	1665.41	2090.86	1995.04	1883.82	2069.09	1837.69
MOG	379.79	563.71	535.19	544.46	474.09	419.21
LASS2	613.94	773.60	799.46	804.42	768.70	722.89
ADSSL1	526.60	653.39	607.32	563.43	661.46	637.50
OLIG1	3788.25	4285.84	4411.12	4559.03	4796.23	4612.18
GLTP	586.45	707.07	717.50	790.80	746.00	689.84
CNP	476.63	614.93	689.64	603.29	613.05	561.08
FUT9	186.37	209.36	191.67	193.86	241.04	201.80
TRIM59	196.50	230.83	235.63	258.71	254.40	240.15
SC4MOL	1416.04	1812.87	1609.18	1728.97	1834.12	1720.20
EDG2	247.87	333.06	329.97	330.12	320.58	331.38
HTR1F	257.31	307.60	298.44	293.46	333.55	295.49
SST	5639.65	7078.31	7273.58	7060.53	7318.46	7443.51
TMEM178	1990.24	2514.92	2326.59	2251.67	2623.36	2642.73
ARC	1540.68	1276.26	1674.46	1495.06	2034.59	1923.85
MOBP	406.82	582.90	625.05	616.31	539.88	495.93
PLEKHB1	1869.85	2605.75	2931.23	2659.69	2473.21	2236.16
IL33	409.98	507.17	498.85	473.65	547.23	520.73
ENPP6	232.18	313.70	337.68	341.00	310.33	322.50
DUSP1	1527.60	1299.03	1500.56	1314.19	2057.41	1841.03
FA2H	829.22	1272.96	1325.89	1271.81	1117.86	998.97
OPALIN	401.24	509.14	526.93	511.40	540.91	455.11
MEGF9	698.69	608.72	750.40	692.27	943.72	715.20
QDPR	683.92	958.86	836.47	862.82	926.45	910.80
KCNA1	876.05	1106.77	956.87	965.99	1189.11	1040.73
TRF	948.66	1574.05	1574.61	1670.56	1296.39	1256.91
NDRG1	393.75	540.13	518.09	611.24	543.77	510.41
PLP1	8450.14	10942.18	11020.21	11229.94	11832.35	10984.42
TSPAN2	601.13	827.53	809.55	881.84	843.12	763.16
PRR18	918.15	1476.38	1542.27	1453.68	1294.13	1108.98
CLDN11	5329.87	8074.15	8736.34	8323.82	7533.85	6794.11
MBP	4321.70	6460.55	6594.92	6205.91	6104.00	5576.93
SCN4B	652.43	928.71	816.18	700.53	920.11	891.47
MAG	1583.86	2329.69	2642.31	2425.40	2240.27	2110.33
UGT8A	474.35	684.72	675.81	707.48	682.65	630.29
GJC2	265.03	368.71	370.38	366.01	383.14	344.61
MAL	826.96	1246.55	1364.64	1242.61	1289.23	1145.85
BCAS1	3152.48	5046.88	5330.38	5369.41	5088.14	4732.15
NEFM	878.94	1175.00	843.35	934.53	1421.93	1277.82

LPGAT1	280.36	347.97	324.80	346.97	455.14	384.33
6030405A1	658.07	524.12	534.86	546.87	568.95	635.73
TBC1D13	536.56	479.70	522.24	509.03	480.66	498.28
LGI2	545.59	613.09	606.22	568.78	618.33	562.41
SERGEF	318.89	277.65	309.39	295.60	281.98	292.13
AB182283	387.07	429.12	418.85	413.91	427.58	429.18
LYZ2	192.44	166.04	186.78	179.24	171.34	168.62
KLF2	221.50	189.91	207.49	209.60	198.68	209.84
LOC10004(	226.59	289.88	248.57	262.41	260.94	243.95
NFIB	1616.99	1982.83	2041.95	2130.23	1908.67	1817.03
PACS2	1841.68	2166.36	2192.98	2147.57	2051.80	2146.02
2310021P1	562.50	486.53	548.52	550.59	482.38	533.45
CRYAB	414.60	570.25	554.41	544.50	532.67	487.44
LRRN1	778.36	915.88	843.79	916.02	915.15	874.09
NDST1	761.56	877.76	888.62	871.09	839.24	842.49
NRP1	812.07	1010.23	964.48	943.10	925.56	872.16
ZBTB7C	226.43	196.30	213.72	214.06	203.21	211.38
VSTM2B	1268.16	1484.47	1342.76	1450.16	1416.02	1424.55
OPRK1	161.60	183.67	181.43	167.34	181.55	167.87
TMEM19	243.95	278.29	264.83	276.08	275.34	276.99
PRKG2	279.98	233.95	282.45	265.32	246.31	250.33
BTBD3	795.83	611.27	660.22	571.77	713.92	634.90
CBLN4	403.24	345.10	334.00	350.12	361.38	377.84
UNC5B	205.92	255.06	243.66	246.55	226.91	218.24
ZDHHC14	833.10	721.15	732.39	729.73	734.99	777.54
SLC6A13	190.04	152.52	166.91	171.23	169.03	176.76
4930511J1	200.98	176.20	188.98	183.89	181.48	186.29
MARCH4	257.22	223.64	241.31	243.45	228.29	236.81
SLAIN1	221.18	261.20	249.38	258.04	257.00	262.55
ATP5F1	1552.15	1798.12	1576.69	1807.40	1780.70	1734.55
INPPL1	444.00	491.22	496.47	483.01	485.56	480.28
PLEKHA2	612.50	754.15	650.22	663.68	690.85	623.16
OTX1	547.41	718.79	603.33	586.35	624.62	642.74
B930076A(	604.20	524.33	537.96	551.00	540.53	645.95
RIMS3	2519.10	1976.33	2206.97	1943.18	2149.75	2391.44
DRD1A	413.18	501.76	550.61	537.63	466.99	433.19
LOC10004	274.56	331.43	294.12	314.58	328.10	323.56
RAB5A	807.86	897.68	827.99	945.98	896.50	889.52
CUX2	677.29	356.68	562.84	499.76	582.78	607.63
KCNK6	236.02	212.35	222.65	215.19	216.24	217.74
CTGF	1097.07	2053.17	2004.13	1923.24	1219.71	1110.06
DLK2	565.92	489.06	561.59	565.28	502.54	514.97
ADAMTSL2	200.77	175.55	189.48	187.89	181.60	178.83

EG434858	417.55	459.16	401.41	438.12	381.80	380.24
FMO1	204.63	170.22	201.41	200.30	182.15	188.00
MTMR2	230.79	256.07	236.84	244.42	252.88	257.36
S100A6	265.31	240.43	260.68	235.37	241.42	244.36
EFCAB1	235.58	187.67	213.94	212.61	211.87	217.93
FBXL10	304.59	273.78	287.67	288.87	275.88	295.38
OTOF	181.64	147.99	174.05	179.42	165.15	162.66
NDRL	414.88	537.00	585.08	604.53	483.36	496.92
EGR2	373.88	265.16	290.74	313.47	343.96	324.76
RREB1	293.53	230.26	268.56	273.06	269.84	276.96
NAV1	1510.42	1336.60	1445.18	1475.30	1361.56	1434.41
ANXA3	418.02	376.20	391.34	387.77	380.51	385.25
TTYH2	176.23	205.04	202.96	197.49	194.46	193.73
VGF	3807.62	3168.51	3477.84	3543.20	3461.53	4088.61
SEMA5A	726.57	1092.01	963.58	956.71	806.17	734.83
SLC26A4	374.46	321.96	351.80	358.19	340.13	337.14
TLE1	572.62	471.37	512.53	485.73	527.05	521.67
EPHX1	307.54	259.82	290.15	273.14	278.30	288.15
VASN	293.87	244.71	242.30	253.75	261.41	264.05
SOX5	391.94	456.21	438.53	425.61	429.25	389.66
RASGRF1	5854.33	5330.52	5269.98	5401.88	5397.43	6044.15
IL11RA1	2002.59	2305.29	2009.94	2177.15	2221.93	2000.44
ANKRD6	211.69	179.57	204.11	202.65	194.83	202.28
SLC7A11	407.83	333.09	400.04	392.46	359.00	368.23
AI314976	163.01	185.01	173.67	171.70	181.26	178.25
FAM13C	346.17	305.61	329.32	340.01	314.79	330.56
CALB2	297.13	253.07	288.83	283.41	266.39	276.49
C130090K2	199.11	233.60	206.01	220.73	215.44	208.37
CDC42EP2	236.69	267.15	272.09	256.38	258.20	242.12
CAMK2A	2651.15	2308.83	2368.00	2124.45	2424.92	2572.00
SYTL2	1163.99	1020.31	1011.30	946.71	1050.51	1128.40
IGSF9	178.10	161.56	164.07	166.36	164.38	171.02
RGS4	5781.32	5197.34	5082.36	5356.50	6300.89	6096.10
EHMT2	614.33	540.75	589.02	586.15	564.10	607.10
REEP5	3056.28	3550.15	3767.85	3430.98	3398.74	3877.83
JOSD1	2080.26	1848.11	2169.01	1923.15	1917.87	1928.92
GAL3ST1	271.08	340.22	315.37	310.68	299.90	297.69
HIST1H2A(	1255.30	1124.75	1430.46	1254.04	1143.05	1110.91
TCF4	10613.21	9451.73	9400.15	9804.75	9605.30	9653.79
FBF1	316.25	268.12	303.02	289.86	286.34	291.02
ADAMTSL4	160.07	176.81	170.60	186.64	174.55	175.43
SLC4A3	652.83	548.70	572.23	583.05	607.49	661.85
DIAP3	211.68	234.31	202.14	195.97	231.02	223.85

GUCY1A3	955.42	793.52	841.13	856.16	854.17	852.45
CAMK1G	216.30	196.82	208.04	203.23	200.95	211.29
PDLIM2	208.85	247.29	253.37	235.29	229.94	218.49
2810405K(	649.48	541.57	580.08	600.33	579.76	571.86
GADD45A	352.41	423.56	391.14	408.52	383.95	367.89
RYR1	218.12	194.16	204.53	211.66	203.28	213.09
RXFP3	183.19	161.04	163.69	173.10	168.22	181.38
FIGN	157.09	174.03	173.34	177.21	170.82	161.75
CLCN2	329.82	283.15	293.42	290.52	301.22	318.66
AB112350	277.48	249.40	257.94	258.39	254.83	248.14
PLEKHG3	248.74	289.38	294.33	296.47	270.75	270.06
RELN	1780.64	1389.91	1470.76	1566.18	1614.53	1658.83
THAP3	153.79	172.94	164.65	156.55	169.50	158.83
TGM2	278.46	231.16	241.67	254.44	253.73	246.20
ZFP148	844.75	717.86	824.35	823.68	777.50	791.13
ACTA2	238.57	209.02	224.38	237.29	220.98	220.62
ANO4	419.17	494.41	472.43	448.80	468.14	406.89
BC030499	222.30	196.42	216.04	204.06	202.94	198.45
ITGB4	199.11	245.28	252.41	235.69	216.41	213.37
NPY1R	392.93	336.64	329.01	339.13	351.89	368.70
DARC	1120.18	954.04	1090.13	1029.18	1029.41	1043.69
ADORA1	588.66	685.14	673.37	663.58	646.40	638.95
RIMS1	514.61	624.73	550.06	545.58	573.60	544.49
TMEM63A	214.55	243.27	251.39	256.02	236.22	229.54
MIF4GD	257.16	284.25	276.57	280.96	276.63	290.28
GAB1	450.16	554.87	567.70	578.06	485.73	472.56
AGAP1	2043.09	2365.64	2418.73	2548.42	2285.70	2144.77
NIPA1	776.71	909.57	832.95	920.70	873.40	818.32
BCL9L	440.23	393.57	405.30	410.96	404.89	399.42
TRIB2	652.88	560.47	641.07	586.46	584.02	613.86
BDNF	445.07	364.05	397.02	390.13	406.96	409.15
NFE2L3	160.27	181.55	163.31	173.89	175.70	162.07
PRELP	367.46	288.60	350.56	329.78	337.36	362.23
WASF1	11317.62	9601.40	10130.59	10498.74	10366.80	9774.86
TMEM125	211.40	242.38	245.66	229.43	227.81	226.15
NEURL	5971.19	4949.24	5594.98	5470.42	5322.78	5214.05
HSPA2	1396.52	1165.77	1262.46	1253.90	1267.46	1360.04
1700019D(	414.03	478.00	444.08	451.49	452.73	435.84
EFHD1	303.00	353.33	356.90	356.09	333.36	310.98
CDH8	328.49	271.96	277.43	281.13	308.82	312.68
PLCH2	665.17	542.21	626.07	625.93	589.20	682.63
ZFPM2	894.12	1261.37	1112.32	1157.32	974.00	873.70
RAPGEF4	1891.66	2219.49	2253.17	2282.20	2111.76	2218.30

STK4	962.81	1083.85	1005.24	1010.95	1047.38	1000.58
DOK4	235.10	274.34	266.02	259.19	261.48	255.92
PDE4D	199.43	179.09	191.76	184.04	185.07	188.42
ODZ3	214.91	240.12	237.67	233.82	230.90	209.23
CYP1B1	167.80	149.52	162.63	165.98	156.79	159.03
TRIM36	186.44	211.93	197.79	206.09	200.46	201.31
ANXA11	855.17	720.09	727.51	720.11	770.81	837.68
TTC19	752.79	854.73	751.82	782.02	812.49	822.00
JUNB	766.44	590.53	688.87	683.77	676.05	668.71
UAP1	794.12	950.09	826.72	840.53	877.89	936.57
EXTL3	1298.53	1132.39	1171.11	1140.84	1200.59	1266.70
RNMT	556.91	646.07	594.02	604.22	612.02	587.83
D930028F1	313.04	283.12	284.82	288.98	293.86	293.34
GM129	360.73	316.29	325.40	340.26	334.06	324.13
C1QC	540.14	462.37	519.81	524.61	498.95	501.68
ATP8B1	171.34	190.38	179.70	183.20	183.85	176.89
NTSR1	256.74	307.02	301.47	298.20	232.82	239.03
PHLDB1	308.10	359.13	362.91	379.85	336.59	357.50
S100A8	200.36	168.40	233.87	182.29	188.23	171.16
RNF19A	1014.98	874.02	1003.75	973.55	929.69	912.48
NPTX2	1680.11	1463.07	1610.33	1473.78	1555.46	1638.59
RHBDL3	278.90	244.98	264.75	256.68	257.40	271.69
H47	1242.18	1110.26	1204.20	1143.34	1157.20	1159.25
MYO5B	169.77	188.88	166.09	181.35	181.67	165.94
RGL1	1773.48	1586.52	1810.62	1842.91	1665.22	1768.86
NGFRAP1	3916.80	3495.32	3835.33	3527.18	3674.71	3448.63
BC067047	439.43	380.43	412.90	401.68	407.11	391.92
KHDRBS2	294.65	260.53	264.67	267.56	273.57	269.59
TPBG	433.83	510.54	484.42	505.83	467.57	442.63
AI593442	949.69	838.92	841.05	807.60	889.03	938.54
4930544G2	2851.14	2507.89	2742.60	2645.72	2654.04	2723.85
DUSP18	203.63	174.38	199.22	184.00	186.26	189.01
NMRAL1	282.12	330.56	334.06	329.11	301.06	281.00
D930015E0	193.37	173.88	196.76	183.99	181.77	191.92
FSTL4	483.61	394.15	429.53	392.78	447.10	496.40
NOL4	899.84	799.19	841.30	932.34	966.98	1009.85
GLRA2	699.73	960.30	857.15	920.17	769.32	717.05
CAR14	206.29	236.91	224.76	247.87	222.98	225.28
5330439J0	895.33	781.95	874.87	832.75	831.40	811.92
CHRM2	176.09	195.76	188.47	180.34	186.32	173.60
SAP130	560.94	506.61	551.85	570.50	531.92	556.05
EFEMP2	216.01	193.79	211.02	210.64	202.42	203.80
OLFML3	373.32	337.08	375.45	345.28	352.82	366.58

SPOP	4377.27	4993.11	4685.34	4517.82	4041.14	4369.55
BHLHB2	2890.24	2142.83	2256.79	2509.82	2590.74	2771.66
ETS1	318.86	283.30	280.09	291.28	297.86	301.08
OSBPL9	1248.81	1498.25	1344.75	1407.49	1342.45	1381.47
CDC42EP4	349.00	299.21	328.65	308.10	321.83	313.13
PCTK1	1598.93	1413.91	1499.08	1357.50	1497.27	1497.35
LOC10004	510.66	453.93	506.92	477.86	481.32	470.01
SULT1A1	259.56	229.63	235.70	234.65	246.14	261.72
PRSS35	303.44	438.08	379.38	435.26	328.93	315.81
ABCA8A	157.19	175.40	165.58	174.56	165.58	166.49
EMID2	194.20	174.18	190.65	185.52	183.12	195.98
CD82	242.27	280.41	278.29	280.43	256.38	251.66
THBD	177.96	160.85	166.82	175.65	168.19	180.02
PDYN	564.67	446.60	487.61	522.67	530.79	559.38
NPM3-PS1	677.23	603.42	735.44	611.57	635.44	603.10
RYR3	252.17	285.35	280.18	300.03	237.87	268.50
RANBP3L	279.88	234.93	262.12	258.74	262.10	256.80
ZIC1	303.99	250.65	284.10	294.74	278.42	276.19
ST8SIA2	159.44	141.62	147.94	155.87	151.68	148.55
MKX	438.89	391.22	385.95	403.25	415.71	421.37
CCK	4818.67	4050.72	4451.65	4480.14	5111.62	5122.93
RPRM	1636.35	2904.01	2434.56	2352.35	1823.11	1672.55
CPNE9	1264.90	982.09	1046.20	1051.74	1185.53	1341.66
DUSP6	1181.47	975.13	1035.56	1045.70	1256.03	1220.21
RGS6	250.35	223.66	238.42	239.69	236.99	235.06
OTOP2	179.91	158.77	173.93	168.85	169.39	171.00
ASAH2	340.34	386.58	342.07	358.83	359.79	353.92
CDH7	243.50	207.90	216.06	224.86	229.74	228.98
ELOVL6	756.68	848.26	785.77	833.47	797.93	745.41
ADRA2A	415.49	601.35	561.49	583.31	442.53	432.46
CD47	1431.79	1651.05	1705.41	1621.39	1524.21	1477.91
DYNC1LI2	313.30	354.77	327.67	325.06	331.00	327.37
PRDX3	605.39	686.42	680.17	658.36	644.19	655.15
CHST1	7898.66	6610.38	7156.60	7109.08	7321.06	7216.56
4933439C	2067.27	1799.22	2061.91	2077.09	1932.44	2285.46
AGFG1	530.58	477.07	519.76	523.94	507.75	486.78
CAMKK1	828.92	735.68	786.70	826.35	780.26	781.73
CD83	363.63	316.53	324.92	349.44	341.73	370.67
HSD17B11	242.19	271.71	230.26	245.31	229.48	230.82
PIGP	915.53	1037.52	887.37	977.14	958.83	948.24
FGF10	254.50	295.15	292.33	289.72	265.89	260.87
GPR83	1087.45	959.60	1019.90	987.57	1022.87	989.21
SLA	416.64	581.02	560.89	567.69	389.53	369.73

CASP1	232.58	200.49	205.56	207.37	220.36	218.37
THSD7B	176.61	202.84	194.76	194.53	184.03	188.43
GPR21	199.99	235.50	214.63	208.15	214.67	214.01
H2AFY	865.85	987.68	941.55	946.11	911.74	936.09
PRICKLE1	1970.16	2297.85	2078.46	2218.00	2095.07	2190.24
FBXO32	169.35	185.87	178.33	175.58	176.26	176.57
ZYX	1971.59	2287.55	2164.05	2134.91	1857.09	1889.25
CRTAC1	553.01	663.51	617.71	591.17	588.48	630.69
RTN4RL1	648.52	521.49	552.85	577.60	599.94	641.02
DNAJB1	2796.87	2438.78	2792.62	2491.59	2624.52	2587.70
ADCYAP1	433.84	360.11	386.34	360.42	406.12	440.27
TRPC7	329.52	285.79	338.03	332.50	308.98	310.01
PER1	778.30	643.71	682.48	755.71	728.59	753.31
INPP4B	189.56	209.95	196.16	200.93	181.30	184.57
CSF1R	351.57	312.92	329.99	313.89	336.10	346.25
NOS1AP	338.57	413.59	336.06	326.02	364.56	314.60
WDR60	325.31	287.55	304.81	334.17	308.26	301.12
GRIK1	433.54	491.88	462.23	450.41	455.63	418.67
GCNT2	694.93	552.58	590.49	617.41	658.94	674.98
FAM132B	169.53	187.78	179.80	178.09	176.07	173.53
LMO3	322.55	364.94	354.46	335.42	308.21	298.72
LOC10004	282.24	247.33	278.06	282.86	270.13	272.96
ARHGAP2	363.12	462.50	435.66	419.14	380.00	376.15
HIST2H3B	1149.89	1028.72	1121.36	1151.02	1100.54	1056.16
DBNDD2	1962.29	2267.09	2263.98	2108.01	2044.10	1860.67
RASL11B	2320.70	2734.13	2511.37	2313.46	2407.48	2280.35
LOC10004	833.55	1011.17	1068.05	1074.03	876.24	830.74
CGGBP1	1504.27	1320.62	1495.34	1477.30	1438.94	1450.67
SCHIP1	3360.97	2906.10	3221.90	3278.62	3210.34	3345.23
TNNC1	661.09	385.53	549.43	568.08	623.77	665.74
RAB3B	266.92	302.22	282.90	294.47	278.55	290.79
CPNE8	307.04	278.94	262.56	278.05	295.98	289.91
LINGO2	416.21	347.73	374.95	364.14	400.06	394.93
GBP2	185.16	183.29	295.88	239.48	178.80	175.44
NEU2	522.39	610.93	536.87	563.41	545.87	545.96
PADI6	164.27	193.63	191.54	186.80	157.56	157.46
CAMTA2	6252.58	5461.76	5698.88	5405.97	6003.96	6235.03
HAPLN2	179.78	208.66	208.11	198.23	187.11	194.19
EPB4.9	2208.69	2012.11	2034.88	2074.39	2132.18	2229.23
LOC10004	4000.94	2921.47	3217.07	3406.41	3729.92	3737.90
TPD52L1	277.73	246.07	255.56	249.01	268.99	272.63
CHRNA5	167.67	189.90	175.71	181.60	173.52	175.54
MT3	1417.98	1280.06	1446.32	1288.16	1367.27	1297.53

H2-T23	333.01	387.44	349.89	353.86	345.27	334.26
ODZ4	1386.94	1162.36	1242.41	1317.37	1327.34	1219.20
MYOC	160.49	139.86	149.69	154.45	153.83	151.43
SYT17	199.62	173.68	186.34	191.52	192.65	183.60
SLC1A3	6404.05	5588.08	6344.83	5963.60	6633.87	6784.67
OSBP2	570.25	487.79	482.44	476.17	600.94	589.13
EGR4	1280.54	915.08	1027.40	1023.88	1208.43	1195.97
STAC2	1044.30	1186.82	1024.64	1069.60	1089.10	1001.50
SEPT5	1306.74	1151.57	1158.89	1125.56	1256.53	1272.18
ERDR1	380.35	329.79	399.57	387.37	365.48	374.24
COCH	232.94	189.50	192.97	212.67	242.24	223.63
SLC2A13	2557.38	2199.50	2108.99	2174.50	2450.06	2595.53
LOC10004	539.61	450.74	510.91	507.31	516.64	543.69
6330406I1	477.05	430.71	408.67	448.87	462.75	477.33
SLC7A4	1505.26	1696.64	1579.82	1710.71	1558.99	1615.19
TMCC2	626.15	525.47	619.99	638.06	600.65	658.68
HRASLS	430.83	390.80	413.81	391.27	419.16	392.00
HEY1	1881.82	1649.42	1694.87	1747.76	1812.52	1778.13
SPHK1	188.59	171.63	181.56	173.33	183.46	185.39
AUTS2	272.83	233.45	263.01	242.87	262.62	261.50
SLC24A3	3474.81	2996.30	3037.47	3052.50	3617.58	3788.91
RGS9	446.05	556.30	618.82	571.05	428.62	451.07
SPSB1	525.76	723.62	692.42	699.95	545.76	538.12
AGGF1	730.21	641.56	669.94	689.93	705.90	687.04
HS3ST2	201.25	224.33	219.47	204.56	207.12	210.29
PPP1R1B	447.71	579.56	561.10	540.40	461.81	438.68
SERINC2	173.64	198.93	190.00	192.46	178.75	188.86
ZDHHC9	342.63	434.80	413.25	384.49	354.69	365.45
HDAC11	652.14	561.43	646.75	573.83	629.45	638.18
OG9X	173.06	195.53	176.69	178.27	167.97	170.51
DBP	2433.97	2024.49	2370.01	2374.54	2342.47	2152.20
MARCKSL1	345.14	270.70	330.30	318.97	356.32	376.71
ISLR2	731.94	968.39	790.27	827.55	670.33	741.76
TMEM38A	509.84	584.14	521.37	540.57	492.66	530.52
VAT1L	1087.26	920.50	943.19	1099.46	1047.12	1094.54
PDLIM1	421.92	522.82	480.35	464.58	433.89	445.82
MMD	8482.89	7429.00	7817.46	8419.75	8233.30	8384.10
VANGL2	282.49	338.08	310.16	313.27	288.44	284.02
HKDC1	348.57	277.23	300.78	314.74	338.41	360.98
D12ERTD6	915.64	1105.71	1097.55	1096.81	934.58	888.64
BHLHB5	249.12	196.43	217.38	241.43	237.05	249.49
DCLK1	5309.08	5845.87	5358.84	5536.80	5426.92	5851.93
ARPP21	5417.17	4769.25	5119.25	5156.64	5308.69	5420.91

2810022L0	273.70	233.00	264.96	256.97	281.76	287.37
3110035E1	5513.09	6445.62	6082.39	6396.53	5664.65	5964.72
BCL11B	1427.67	1721.76	1772.31	1763.39	1472.74	1429.29
ARHGAP29	322.49	290.55	303.67	305.72	315.97	320.13
B3GNT8	164.76	143.54	158.46	154.82	160.39	159.98
LRRTM2	706.84	806.81	738.34	783.44	725.77	688.12
MAPK4	278.03	243.79	253.20	257.42	272.60	262.83
RNF144A	467.14	401.80	432.28	484.11	480.24	490.50
FOXN3	359.62	424.53	403.54	379.58	349.20	332.17
PIK3R3	1187.29	1434.08	1228.86	1276.70	1158.59	1157.54
CHN2	980.66	888.74	891.89	986.77	998.18	980.46
NXPH2	321.93	377.02	341.24	344.33	331.06	328.48
FANCD2	250.87	311.61	309.35	282.76	255.54	252.15
DOCK4	518.71	445.36	457.82	451.84	505.19	513.24
SLC39A10	705.56	565.26	573.47	594.46	736.76	733.99
MEF2C	5061.75	4151.96	4717.64	4632.15	5167.78	4596.98
SEMA3F	223.14	248.10	239.83	239.48	218.99	219.25
IGSF21	738.35	980.74	880.79	880.45	716.54	738.39
PPM2C	564.01	486.89	491.18	515.53	553.51	523.49
GJB6	1879.40	1500.93	1581.09	1605.20	1923.77	2008.96
YWHAZ	7326.44	6495.13	7192.16	7008.91	7191.60	7357.89
SYNE1	1317.36	1122.68	1130.12	1154.03	1291.57	1178.14
RAI14	377.14	490.11	478.38	498.71	383.77	373.36
RASGRF2	160.73	145.56	151.04	157.30	162.96	157.08
AGPAT4	632.47	735.58	770.45	720.51	645.04	623.76
GARNL3	3111.10	3929.62	3485.82	3424.77	3183.61	3047.90
PRR13	2259.57	2043.92	2155.98	2138.58	2232.08	2352.09
SCCPDH	3674.87	3155.48	3695.48	3582.46	3728.55	3737.46
PDZRN3	675.29	464.16	518.09	565.16	684.88	754.68
GFM1	237.02	269.98	236.99	232.07	233.75	243.52
GRB7	170.83	193.71	186.30	187.09	172.83	179.57
EFNA5	1454.37	1237.09	1340.07	1242.22	1433.79	1403.20
COBL	510.36	442.30	494.33	462.78	517.51	522.71
LOC100048	509.25	423.93	496.10	564.95	500.51	512.43
KRT12	798.13	651.34	652.84	649.98	788.71	785.70
RPH3A	1019.21	1170.50	1051.98	1015.18	1030.49	1100.79
B2M	2120.13	1922.61	2287.40	2135.58	2103.02	2141.42
DDAH1	2335.58	2778.16	2719.25	2637.77	2306.61	2173.58
AW049604	735.76	642.00	701.70	686.61	728.07	708.46
OGFRL1	1850.47	2215.60	1924.08	2173.10	1871.61	1776.90
SDK1	212.21	236.58	244.32	230.62	213.62	212.66
TNFRSF19	306.00	264.12	293.35	285.22	307.80	294.36
PHLDA3	376.51	416.07	394.19	382.81	374.31	381.36

WHRN	488.05	548.20	550.86	542.56	490.67	489.83
LY6G6E	203.15	239.89	221.57	229.51	202.03	194.69
RBP1	207.50	178.82	193.55	189.03	206.60	195.47
TAC1	497.81	559.88	549.63	540.89	499.75	526.13
WNT2	225.73	256.38	251.13	250.95	226.74	240.86
BC064033	165.23	146.40	157.02	152.50	165.76	164.01
KLF7	1347.77	1583.65	1388.04	1424.63	1354.48	1397.90
DKKL1	328.91	282.41	298.58	302.60	327.49	326.08
SAMD9L	279.71	322.76	330.02	294.42	278.45	279.08
IGFBP2	225.21	198.58	211.95	209.89	225.82	225.08
CNTNAP4	829.68	1049.15	958.28	1044.39	826.29	836.71
TCF19	189.50	171.58	184.79	187.11	189.03	185.36
ENSMUSG	219.67	257.36	262.19	258.44	219.33	204.73
NRIP3	2235.97	2759.90	2442.87	2549.27	2241.60	2358.07
ARNTL	480.37	408.99	402.87	447.18	479.08	527.21
OLFML2B	354.31	405.01	357.14	375.57	354.57	354.81
LRFN2	417.14	500.49	462.73	471.15	416.79	410.72
