# Supplementary material for: Molecular laterality encodes stress susceptibility in the medial prefrontal cortex
Source: Mol Brain. 2021 Jun 14;14:92. doi: 10.1186/s13041-021-00802-w (PMC8201740; doi:10.1186/s13041-021-00802-w)
Supplement: Supplementary file 6 — Additional file 6: Table S4. The 526 genes exhibiting laterality listed, according to their log2L/R values. Laterality genes with positive values are expressed more highly in the left mPFC, and those with negative values are expressed more highly in the right mPFC. The genes used in rank order analysis in Fig. 1e. [file 13041_2021_802_MOESM6_ESM.pdf]

Supplementary table 4
The 526 genes exhibiting laterality listed, according to their log_2 L/R values. Laterality genes with positive values are expressed more highly in the left mPFC, and those with negative values are expressed more highly in the right mPFC. The genes used in rank order analysis in Figure 1e.

DEG Gene	Log2(L/R) Susceptible	DEG Gene	Log2(L/R) Resilient	DEG Gene	Log2(L/R) Control
CUX2	0.93	S100A8	0.36	MEGF9	0.40
WFS1	0.83	ACTR1A	0.31	CPLX2	0.34
TNNC1	0.78	GBP2	0.31	NETO1	0.27
STARD8	0.57	NPM3-PS1	0.27	FUT9	0.26
CAR4	0.56	LYZ	0.25	OPALIN	0.25
PDZRN3	0.54	WFS1	0.24	LPGAT1	0.24
VIP	**0.53**	SCN4B	0.22	SLC35F3	0.24
SLC13A4	0.52	IFITM3	0.22	PRR18	0.22
DDIT4L	0.51	BTBD3	0.21	SULF1	0.22
IGF2	0.51	MFGE8	0.20	NOS1AP	0.21
EGR2	0.50	PLLP	0.20	EVI2A	0.21
EVC2	0.49	CNP	0.19	ANO4	0.20
CDKN1C	0.49	DUSP1	0.19	KCNA1	0.19
EGR4	0.48	HIST1H2A(	0.19	MOG	0.18
CPNE6	0.48	RIMS3	0.18	HTR1F	0.17
CPNE4	0.47	SCD1	0.18	MYL4	0.17
DCN	0.47	MORN4	0.18	MAL	0.17
LOC10004(	0.45	JOSD1	0.17	BTBD3	0.17
BHLHB2	0.43	HDAC11	0.17	MEF2C	0.17
PVRL3	0.42	CUX2	0.17	FA2H	0.16
TIAM1	0.40	ZBTB8B	0.17	TLE4	0.16
RILPL1	0.39	MT3	0.17	DUSP1	0.16
BTBD3	0.38	SAMD9L	0.16	ZFPM2	0.16
JUNB	0.38	DNAJB1	0.16	NEFM	0.15
PALMD	0.38	ARC	0.16	GJC2	0.15
CPNE9	0.37	CAMK2A	0.16	IL11RA1	0.15
LOC10004!	0.36	INA	0.16	KCNK13	0.15
C1QTNF4	0.36	UACA	0.15	CLDN11	0.15
DACT2	0.36	S100A6	0.15	PLEKHA2	0.15
LPL	0.36	PCTK1	0.14	PLEKHB1	0.15
RELN	0.36	PLEKHB1	0.14	TSPAN2	0.14

GRASP	0.35	MGP	0.14	ODZ3	0.14
SPAG5	0.35	ACSL5	0.14	SOX5	0.14
MARCKSL1	0.35	MAL	0.14	S100A8	0.14
RREB1	0.35	REEP5	0.14	LGI2	0.14
RIMS3	0.35	FANCD2	0.13	CTGF	0.14
MGP	0.35	FSTL4	0.13	DBNDD2	0.14
PRELP	0.35	TRIB2	0.13	SEMA5A	0.13
BHLHB5	0.34	COTL1	0.13	SYNE1	0.13
HPCA	0.34	NPTX2	0.13	MYO5B	0.13
OGN	0.34	SLC44A1	0.13	MBP	0.13
PDYN	0.34	MAG	0.12	CRYAB	0.13
D8ERTD82	0.34	OLFML3	0.12	CNP	0.13
HBA-A1	0.33	NGFRAP1	0.12	RPRM	0.12
IGSF3	0.33	PLXNB3	0.12	LITAF	0.12
GJB2	0.33	NBL1	0.12	ODZ4	0.12
GCNT2	0.33	RASL11B	0.12	MOBP	0.12
HKDC1	0.33	OPRK1	0.12	DBP	0.12
6030405A	0.33	MEGF9	0.12	GRIK1	0.12
EFCAB1	0.33	RGS9	0.12	PLLP	0.12
LYZ	0.32	AUTS2	0.11	STAC2	0.12
GJB6	0.32	DUSP18	0.11	PIGZ	0.12
FOSB	0.32	HSD11B1	0.11	NFE2L3	0.12
SLC39A10	0.32	EFNA5	0.11	COCH	0.12
SLC6A13	0.32	ADSSL1	0.11	UGT8A	0.12
RTN4RL1	0.31	PDLIM2	0.11	OPRK1	0.11
CITED4	0.31	ZDHHC9	0.10	GLTP	0.11
FAM148C	0.31	CIB2	0.10	ANLN	0.11
LOC10004	0.30	DBNDD2	0.10	DRD1A	0.11
COCH	0.30	PROSAPIP	0.10	PLP1	0.11
OTOF	0.30	HS3ST2	0.10	ARSG	0.11
FSTL4	0.30	ADCYAP1	0.10	BCAS1	0.10
PLCH2	0.29	CDKN1C	0.10	CHRM2	0.10
KRT12	0.29	B2M	0.10	GLRA2	0.10
SLC7A11	0.29	ITPKA	0.10	EFHD1	0.10
2510009E	0.29	ITGB4	0.10	NMRAL1	0.10
PKNOX2	0.29	TMEM125	0.10	ENSMUSG	0.10
CACNG3	0.29	D930015E	0.10	PPP1R14A	0.10
BDNF	0.29	AGPAT4	0.10	ELOVL6	0.10
ECHDC2	0.29	SYTL2	0.10	CARHSP1	0.10
MEF2C	0.29	COBL	0.10	LOC10004	0.10
TLE1	0.28	CDC42EP4	0.09	HRASLS	0.10
ZIC1	0.28	LGI2	0.09	NIPA1	0.09
FOS	0.28	PRKG2	0.09	THAP3	0.09

DUSP6	0.28	SLC1A3	0.09	CDC42EP2	0.09
PER2	0.27	FOXN3	0.09	SC4MOL	0.09
PER1	0.27	PRELP	0.09	AGAP1	0.09
MFGE8	0.27	SLC35F3	0.09	NGFRAP1	0.09
CDH8	0.27	MBP	0.09	NDRG1	0.09
ARC	0.27	EPHX1	0.09	LASS2	0.09
NBL1	0.27	ADAMTS4	0.09	MAG	0.09
NEURL	0.27	SLC13A4	0.09	NRP1	0.09
SERPINF1	0.27	CDC42EP2	0.09	DDAH1	0.09
ADCYAP1	0.27	PRR18	0.09	WASF1	0.08
TGM2	0.27	LOC10004	0.09	PBRM1	0.08
1300013J1	0.27	SDK1	0.08	FOS	0.08
SLC2A1	0.27	IGF2	0.08	INSC	0.08
GUCY1A3	0.27	DARC	0.08	TRIM59	0.08
PTPRK	0.27	MYL4	0.08	EGR2	0.08
DBP	0.27	BC030499	0.08	ARC	0.08
FMO1	0.27	ANLN	0.08	PPM2C	0.08
HAP1	0.27	HRASLS	0.08	RBP1	0.08
VGF	0.27	LMO3	0.08	TPBG	0.08
LOC10004	0.26	TLE1	0.08	SLC44A1	0.08
VASN	0.26	2510009E0	0.08	FIGN	0.08
KCTD4	0.26	CAMTA2	0.08	RASL11B	0.08
2810405K0	0.26	H47	0.07	LOC10004	0.08
HSPA2	0.26	IL33	0.07	LRRTM2	0.08
LOC10004	0.26	NR2F6	0.07	MT3	0.08
LINGO2	0.26	ATG16L1	0.07	NPM3-PS1	0.08
NR2F6	0.26	ANO4	0.07	SLA	0.08
PRKG2	0.26	CD47	0.07	RIMS1	0.08
UACA	0.26	THAP3	0.07	OGFRL1	0.07
CHST1	0.26	CSF1R	0.07	PPP1R1B	0.07
ODZ4	0.25	5330439J0	0.07	PDLIM2	0.07
S100A11	0.25	HAPLN2	0.07	D12ERTD6	0.07
TMCC2	0.25	HAP1	0.07	FOXN3	0.07
RANBP3L	0.25	CLDN11	0.07	IL33	0.07
SLC4A3	0.25	FCHO1	0.07	NFIB	0.07
S100A8	0.25	SPHK1	0.07	SYT17	0.07
CCK	0.25	SERGEF	0.07	ELAVL2	0.07
ANXA11	0.25	FBF1	0.06	LRRN1	0.07
CACNA1H	0.24	CHRM2	0.06	STK4	0.07
EPHX1	0.24	CRTAC1	0.06	DCBLD1	0.07
ACSL5	0.24	CPLX2	0.06	CADPS2	0.06
BMP1	0.24	BOK	0.06	TNFRSF19	0.06
VAT1L	0.24	FA2H	0.06	GARNL3	0.06

FBF1	0.24	NETO1	0.06	GADD45A	0.06
ANKRD6	0.24	LYZ2	0.06	AGFG1	0.06
WASF1	0.24	CTGF	0.06	HIST2H3B	0.06
FCHO1	0.24	PDE4D	0.06	PRSS35	0.06
ZFP148	0.23	AI593442	0.06	RNMT	0.06
DUSP1	0.23	UBQLN4	0.06	ACTR1A	0.06
EFNA5	0.23	PALMD	0.06	OLIG1	0.06
PNCK	0.23	FAM148C	0.06	UNC5B	0.06
2810022L0	0.23	ARHGAP25	0.06	ATP8B1	0.06
ARNTL	0.23	SERPINF1	0.06	BC067047	0.05
DARC	0.23	AKAP8L	0.05	1700019D0	0.05
CALB2	0.23	CITED4	0.05	HBA-A1	0.05
SYNE1	0.23	PPP1R1B	0.05	LY6G6E	0.05
CDH7	0.23	KCTD4	0.05	ADSSL1	0.05
OSBP2	0.23	SPOP	0.05	RASGRF2	0.05
AUTS2	0.22	4930544G2	0.05	MAPK4	0.05
CBLN4	0.22	RPH3A	0.05	AGPAT4	0.05
C1QC	0.22	MARCKSL1	0.05	GPR83	0.05
DUSP18	0.22	RPRM	0.05	C130090K2	0.05
ACCN2	0.22	KCNK6	0.05	RGS4	0.05
NPY1R	0.22	CACNA1H	0.05	H2-T23	0.05
ARSJ	0.22	PDLIM1	0.05	SCN4B	0.05
CDC42EP4	0.22	TMEM178	0.05	DIAP3	0.05
KLF2	0.22	PRDX3	0.05	LMO3	0.05
TRIB2	0.22	GPR83	0.05	TRF	0.04
CLCN2	0.22	SCCPDH	0.04	CD47	0.04
DOCK4	0.22	ERDR1	0.04	PLXNB3	0.04
DKKL1	0.22	DIAP3	0.04	GM129	0.04
SCCPDH	0.22	RHBDL3	0.04	TGM2	0.04
SLC26A4	0.22	GPR21	0.04	BCL11B	0.04
ITPKA	0.22	2810022L0	0.04	DUSP6	0.04
SLC2A13	0.22	RNF19A	0.04	TMEM63A	0.04
RNF144A	0.22	DDAH1	0.04	HIST1H2A(	0.04
ANXA2	0.22	NOS1AP	0.04	RAI14	0.04
HDAC11	0.22	OPALIN	0.04	GAB1	0.04
RNF19A	0.22	SOX5	0.04	CDC42EP4	0.04
RBP1	0.21	SST	0.04	AW049604	0.04
CASP1	0.21	OTOP2	0.04	AGGF1	0.04
SLC24A3	0.21	ZFP810	0.04	AB112350	0.04
LYZ2	0.21	PHLDA3	0.04	ATP5F1	0.04
TNFRSF19	0.21	LINGO2	0.04	LOC10004	0.03
PPM2C	0.21	BC064033	0.04	5330439J0	0.03
DLK2	0.21	SEPT5	0.04	WDR60	0.03

SCHIP1	0.21	OTX1	0.04	ADRA2A	0.03
2310021P1	0.21	TNFRSF19	0.04	BC030499	0.03
ZDHHC14	0.21	FHL1	0.04	ARSJ	0.03
BC067047	0.21	BC067047	0.04	EFNA5	0.03
COBL	0.21	INPPL1	0.04	DOK4	0.03
ZBTB7C	0.21	TTYH2	0.04	HSD11B1	0.03
ERDR1	0.21	4930511J1	0.04	ST8SIA2	0.03
TRPC7	0.21	EMID2	0.04	CPNE8	0.03
FHL1	0.21	HPCA	0.04	NEURL	0.03
B930076A(	0.20	EXTL3	0.04	RANBP3L	0.03
MARCH4	0.20	DDIT4L	0.04	OSBP2	0.03
SYT17	0.20	PVRL3	0.04	TCF19	0.03
4933439C2	0.20	DOK4	0.04	ADAMTS4	0.03
CD83	0.20	TPD52L1	0.04	HEY1	0.03
AKAP8L	0.20	GRIK1	0.04	FGF10	0.03
SERGEF	0.20	YWHAZ	0.04	GBP2	0.03
NPTX2	0.20	TBC1D13	0.04	RNF19A	0.03
CAMK2A	0.20	PADI6	0.04	CD82	0.03
B3GNT8	0.20	DRD1A	0.03	CHN2	0.03
MEGF9	0.20	RBP1	0.03	QDPR	0.02
MYOC	0.20	CAMK1G	0.03	AI314976	0.02
PSME1	0.20	ARSG	0.03	ASAH2	0.02
IFITM3	0.20	B3GNT8	0.03	LYZ2	0.02
DNAJB1	0.20	NEURL	0.03	MYOC	0.02
EXTL3	0.20	NRP1	0.03	VANGL2	0.02
AW049604	0.20	ECHDC2	0.03	ADAMTSL2	0.02
SLC1A3	0.20	HBA-A1	0.03	OTOF	0.02
5330439J0	0.20	AW049604	0.03	LRFN2	0.02
CAMTA2	0.20	GFM1	0.03	KHDRBS2	0.02
ADAMTSL2	0.19	PACS2	0.03	FAM132B	0.02
MMD	0.19	PSME1	0.03	CHST1	0.02
WNT4	0.19	NDST1	0.03	ITGB4	0.02
ACTA2	0.19	CACNB3	0.03	DNAJB1	0.02
LOC10004	0.19	SLC7A11	0.03	SPSB1	0.02
HEY1	0.19	CARHSP1	0.03	LOC10004	0.02
SYTL2	0.19	CALB2	0.03	2810405K(	0.02
4930511J1	0.19	MEF2C	0.03	BCL9L	0.02
GM129	0.19	RILPL1	0.03	FANCD2	0.02
MAPK4	0.19	CRYAB	0.03	LINGO2	0.02
EXDL2	0.19	GARNL3	0.03	ADORA1	0.02
BOK	0.19	KCNK13	0.03	PIGP	0.02
NAPEPLD	0.19	BDNF	0.03	DYNC1LI2	0.02
CGGBP1	0.19	HTR1F	0.02	INPPL1	0.02

RHBDL3	0.19	TRPC7	0.02	JUNB	0.02
AGGF1	0.19	IGSF3	0.02	FAM134B	0.02
RXFP3	0.19	ODZ3	0.02	BC064033	0.02
4930544G2	0.19	TAC1	0.02	EGR4	0.01
FKBP1A	0.19	FBXO32	0.02	TLE1	0.01
EHMT2	0.18	WHRN	0.02	ARHGAP25	0.01
ARPP21	0.18	GAL3ST1	0.02	6330503K2	0.01
GTPBP6	0.18	NMRAL1	0.02	CASP1	0.01
SEPT5	0.18	ADORA1	0.02	SLC26A4	0.01
IGFBP2	0.18	ENSMUSG0	0.02	RGS6	0.01
GPR83	0.18	MOBP	0.02	LPL	0.01
OTOP2	0.18	ZYX	0.02	ZIC1	0.01
FAM13C	0.18	DOCK4	0.02	NXPH2	0.01
AI593442	0.18	OSBP2	0.02	RAB5A	0.01
BC030499	0.18	RANBP3L	0.02	GAL3ST1	0.01
WDR60	0.18	RHOG	0.02	TMEM125	0.01
KHDRBS2	0.18	PIGZ	0.02	EPHA7	0.01
PCTK1	0.18	CGGBP1	0.02	D8ERTD82	0.01
SULT1A1	0.18	AB182283	0.02	DDIT4L	0.01
NAV1	0.18	GJC2	0.02	SDK1	0.01
TPD52L1	0.17	AI314976	0.02	DKKL1	0.01
BC064033	0.17	GTPBP6	0.02	AUTS2	0.01
YWHAZ	0.17	NTSR1	0.02	EG434858	0.01
UBQLN4	0.17	FOSB	0.02	KRT12	0.01
CAMKK1	0.17	LITAF	0.02	SLC39A10	0.01
MAPRE2	0.17	S100A11	0.02	TTYH2	0.01
MORN4	0.17	ANXA11	0.01	CDH7	0.00
NOL4	0.17	CLCN2	0.01	IGFBP2	0.00
ST8SIA2	0.17	IGFBP2	0.01	GPR21	0.00
MID1	0.17	FAM132B	0.01	B3GNT8	0.00
JOSD1	0.17	ANXA3	0.01	PLEKHG3	0.00
ETS1	0.17	FGF10	0.01	GUCY1A3	0.00
LOC10004	0.17	LOC10004	0.01	D930028F1	0.00
CSF1R	0.17	WNT4	0.01	WHRN	0.00
RYR1	0.17	CPNE4	0.01	ACTA2	0.00
TCF4	0.17	CLCN4-2	0.01	ATG16L1	0.00
LOC10004	0.17	ADAMTSL2	0.01	PIK3R3	0.00
NPM3-PS1	0.17	RIMS1	0.01	PADI6	0.00
CYP1B1	0.17	PRR13	0.01	LOC10004	0.00
PROSAPIP1	0.17	COL5A1	0.01	PEBP1	0.00
MKX	0.17	DYNC1LI2	0.01	PCTK1	0.00
NGFRAP1	0.16	INSC	0.01	NEU2	0.00
RGS6	0.16	VIP	**0.01**	OLFML2B	0.00

H47	0.16	JUNB	0.01	SEMA3F	0.00
BCL9L	0.16	RAMP3	0.01	FBXO32	0.00
TBC1D13	0.16	ANKRD6	0.01	H47	0.00
ZBTB8B	0.16	SEMA5A	0.01	CAMKK1	0.00
RGL1	0.16	LOC10004[illegible]	0.01	LOC10004[illegible]	0.00
HIST2H3B	0.16	HSPA2	0.01	CCK	0.00
ZFP810	0.16	CHST1	0.01	SAMD9L	0.00
COTL1	0.16	EFCAB1	0.01	SCCPDH	0.00
HIST1H2A[illegible]	0.16	BMP1	0.01	TIAM1	0.00
EMID2	0.16	FMO1	0.01	SERPINF1	0.00
EFEMP2	0.16	BCL11B	0.01	TRPC7	0.00
CIB2	0.16	EHMT2	0.01	AB182283	-0.01
CACNB3	0.16	SULT1A1	0.01	NDST1	-0.01
PDE4D	0.16	KRT12	0.01	TRIM36	-0.01
AB112350	0.15	ZDHHC14	0.01	CITED4	-0.01
FBXL10	0.15	EGR4	0.00	PVRL3	-0.01
RGS4	0.15	LOC10004[illegible]	0.00	ADAMTSL[illegible]	-0.01
AGFG1	0.15	EFHD1	0.00	TCF4	-0.01
D930015E[illegible]	0.15	EFEMP2	0.00	BDNF	-0.01
KCNK6	0.15	SEMA3F	0.00	ABCA8A	-0.01
ANXA3	0.15	THSD7B	0.00	C1QC	-0.01
ARHGAP2[illegible]	0.15	ZFP148	0.00	JOSD1	-0.01
MT3	0.15	WNT2	0.00	HSD17B11	-0.01
6330406I1	0.15	D12ERTD6	0.00	TMEM19	-0.01
OLFML3	0.15	PPP1R14A	0.00	VSTM2B	-0.01
SAP130	0.15	IGSF21	0.00	EFEMP2	-0.01
THBD	0.15	PLCH2	0.00	KCNK6	-0.01
D930028F[illegible]	0.14	EDG2	0.00	VIP	**-0.01**
PRR13	0.14	ZBTB7C	0.00	TMEM178	-0.01
TCF19	0.14	AB112350	0.00	MID1	-0.01
RASGRF2	0.14	DBP	0.00	CGGBP1	-0.01
FLT1	0.14	LOC10004[illegible]	0.00	OTOP2	-0.01
S100A6	0.14	SCN3B	0.00	COBL	-0.01
CHN2	0.14	2310021P1	-0.01	VASN	-0.01
B2M	0.14	FBXL10	-0.01	CAR14	-0.01
HRASLS	0.14	GRB7	-0.01	RHOG	-0.01
IGSF9	0.14	H2AFY	-0.01	LOC10004[illegible]	-0.02
SCN3B	0.14	SLC24A3	-0.01	SPHK1	-0.02
HSD11B1	0.14	CPNE9	-0.01	ETS1	-0.02
CPNE8	0.14	RGS6	-0.01	CHRNA5	-0.02
CAMK1G	0.14	LOC10004[illegible]	-0.01	TTC19	-0.02
SPHK1	0.14	STK4	-0.01	S100A6	-0.02
RASGRF1	0.14	LASS2	-0.01	SEPT5	-0.02

EPB4.9	0.13	CCK	-0.01	ANXA3	-0.02
ATG16L1	0.12	KIF5A	-0.01	CDH8	-0.02
ACTR1A	0.10	DLK2	-0.01	CNTNAP4	-0.02
GBP2	0.01	STARD8	-0.01	CPNE6	-0.02
FBXO32	-0.13	ARHGAP29	-0.01	COTL1	-0.02
COL5A1	-0.14	PLEKHG3	-0.01	ARHGAP29	-0.02
EG434858	-0.14	ARPP21	-0.01	TPD52L1	-0.02
DCLK1	-0.14	BCAS1	-0.01	MKX	-0.02
ADAMTSL4	-0.14	4933439C2	-0.01	FCHO1	-0.02
PHLDA3	-0.14	CD82	-0.01	HDAC11	-0.02
MIF4GD	-0.14	PNCK	-0.01	DARC	-0.02
INPPL1	-0.15	AGFG1	-0.01	CYP1B1	-0.02
DIAP3	-0.15	OGN	-0.01	UACA	-0.02
INPP4B	-0.15	CASP1	-0.01	DUSP18	-0.02
FAM132B	-0.15	MARCH4	-0.01	LOC100045	-0.02
FIGN	-0.15	OG9X	-0.01	OG9X	-0.02
AB182283	-0.15	NXPH2	-0.01	HS3ST2	-0.02
MTMR2	-0.15	PTPRK	-0.01	DOCK4	-0.02
ATP8B1	-0.15	C1QC	-0.01	EVC2	-0.02
RAB5A	-0.15	KCNA1	-0.01	PRKG2	-0.02
CHRM2	-0.15	DUSP6	-0.01	FBF1	-0.02
SEMA3F	-0.15	ENPP6	-0.01	FKBP1A	-0.02
MYO5B	-0.15	VANGL2	-0.01	PRDX3	-0.02
PIGZ	-0.16	KLF2	-0.01	PER2	-0.02
HS3ST2	-0.16	SPSB1	-0.02	SST	-0.02
SDK1	-0.16	KHDRBS2	-0.02	ZYX	-0.02
ABCA8A	-0.16	H2-T23	-0.02	ZFP148	-0.03
ODZ3	-0.16	FUT9	-0.02	MTMR2	-0.03
CLCN4-2	-0.16	UNC5B	-0.02	INPP4B	-0.03
EPHA7	-0.16	SLA	-0.02	PDE4D	-0.03
ELOVL6	-0.16	TCF19	-0.02	B2M	-0.03
HSD17B11	-0.17	RAPGEF4	-0.02	MMD	-0.03
INSC	-0.17	SERINC2	-0.02	PHLDA3	-0.03
WHRN	-0.17	CDH8	-0.02	INA	-0.03
FUT9	-0.17	DKKL1	-0.02	2810022L0	-0.03
LGI2	-0.17	DACT2	-0.02	PALMD	-0.03
THAP3	-0.17	IGSF9	-0.02	ARPP21	-0.03
TAC1	-0.17	BCL9L	-0.02	RNF144A	-0.03
STK4	-0.17	D930028F1	-0.02	SLAIN1	-0.03
RAMP3	-0.17	ACCN2	-0.02	SLC1A3	-0.03
SLC7A4	-0.17	GJB6	-0.02	YWHAZ	-0.03
6330503K2	-0.17	DCBLD1	-0.02	LOC100048	-0.03
CDC42EP2	-0.17	MIF4GD	-0.02	THSD7B	-0.03

OG9X	-0.18	EXDL2	-0.02	ERDR1	-0.03
OLIG1	-0.18	ARSJ	-0.02	GCNT2	-0.03
LMO3	-0.18	MAPK4	-0.02	CIB2	-0.03
RYR3	-0.18	1700019D(	-0.02	PSME1	-0.04
ELAVL2	-0.18	UAP1	-0.02	DLK2	-0.04
RAB3B	-0.18	RREB1	-0.02	IGSF3	-0.04
DCBLD1	-0.18	RNMT	-0.02	SLC7A11	-0.04
DYNC1LI2	-0.18	LOC10004	-0.02	4930544G	-0.04
CHRNA5	-0.18	MOG	-0.02	RREB1	-0.04
NFE2L3	-0.18	SCHIP1	-0.03	4930511J1	-0.04
PIGP	-0.18	RGL1	-0.03	NTSR1	-0.04
PRDX3	-0.18	GUCY1A3	-0.03	H2AFY	-0.04
TMEM63A	-0.18	C1QTNF4	-0.03	RELN	-0.04
GRB7	-0.18	SLC26A4	-0.03	PDLIM1	-0.04
GRIK1	-0.18	LRFN2	-0.03	GRASP	-0.04
AI314976	-0.18	GAB1	-0.03	NDRL	-0.04
TTC19	-0.18	TMEM63A	-0.03	EFCAB1	-0.04
WNT2	-0.18	VGF	-0.03	OTX1	-0.04
ASAH2	-0.18	SLC4A3	-0.03	OSBPL9	-0.04
STAC2	-0.18	PLP1	-0.03	SCN3B	-0.04
OPRK1	-0.18	EPB4.9	-0.03	CSF1R	-0.04
TRIM36	-0.18	ATP8B1	-0.03	ZDHHC9	-0.04
PRSS23	-0.19	GRASP	-0.03	IGSF21	-0.04
GFM1	-0.19	CYP1B1	-0.03	KCTD4	-0.04
SPOP	-0.19	PLEKHA2	-0.03	6330406I1	-0.04
H2AFY	-0.19	NAV1	-0.03	LOC10004(	-0.05
TMEM19	-0.19	D8ERTD82	-0.03	RELL1	-0.05
LRRTM2	-0.19	SYNE1	-0.03	KLF7	-0.05
CADPS2	-0.19	CPNE6	-0.03	FMO1	-0.05
OLFML2B	-0.19	EVC2	-0.03	MFGE8	-0.05
SERINC2	-0.20	1300013J1	-0.03	EDG2	-0.05
TMEM38A	-0.20	FIGN	-0.03	PER1	-0.05
TMEM125	-0.20	6030405A	-0.03	ZBTB8B	-0.05
CAR14	-0.20	B930076A(	-0.03	PNCK	-0.05
RPH3A	-0.20	INPP4B	-0.03	SKP1A	-0.05
THSD7B	-0.20	PEBP1	-0.03	EPHX1	-0.05
SLC35F3	-0.20	RASGRF1	-0.04	SERGEF	-0.05
IL11RA1	-0.20	PCBP2	-0.04	SLC7A4	-0.05
NDST1	-0.20	EVI2A	-0.04	TBC1D13	-0.05
CD47	-0.21	SLC6A13	-0.04	MARCH4	-0.05
SAMD9L	-0.21	KLF7	-0.04	HAPLN2	-0.05
1700019D(	-0.21	HIST2H3B	-0.04	CALB2	-0.05
PLLP	-0.21	LOC10004	-0.04	SPAG5	-0.05

DBNDD2	-0.21	SYT17	-0.04	ANKRD6	-0.05
CD82	-0.21	TMCC2	-0.04	CAMTA2	-0.05
AGAP1	-0.21	AGGF1	-0.04	GRB7	-0.06
ATP5F1	-0.21	GJB2	-0.04	OLFML3	-0.06
FGF10	-0.21	NPY1R	-0.04	ENPP6	-0.06
RNMT	-0.21	OTOF	-0.04	ZBTB7C	-0.06
ZYX	-0.21	SLC2A13	-0.04	IGSF9	-0.06
HAPLN2	-0.21	HEY1	-0.04	PRSS23	-0.06
REEP5	-0.22	QDPR	-0.04	BOK	-0.06
ARSG	-0.22	MYOC	-0.05	GFM1	-0.06
AGPAT4	-0.22	MTMR2	-0.05	ACCN2	-0.06
PLXNB3	-0.22	FAM13C	-0.05	SCHIP1	-0.06
PLEKHG3	-0.22	FOS	-0.05	NAPEPLD	-0.06
H2-T23	-0.22	DCLK1	-0.05	CUX2	-0.06
TTYH2	-0.22	NDRL	-0.05	GJB2	-0.06
ADORA1	-0.22	OLIG1	-0.05	LYZ	-0.06
SOX5	-0.22	CHRNA5	-0.05	RAB3B	-0.06
PHLDB1	-0.22	SAP130	-0.05	GJB6	-0.06
PBRM1	-0.22	TNNC1	-0.05	CPNE4	-0.06
EFHD1	-0.22	TIAM1	-0.05	NOL4	-0.06
PRICKLE1	-0.22	SLAIN1	-0.05	VAT1L	-0.06
CPLX2	-0.22	RYR1	-0.05	SAP130	-0.06
DOK4	-0.22	2810405K(	-0.05	PRICKLE1	-0.06
3110035E1	-0.23	LY6G6E	-0.05	EPB4.9	-0.06
NEU2	-0.23	WASF1	-0.05	CBLN4	-0.06
NETO1	-0.23	SLC39A10	-0.05	KIF5A	-0.06
VSTM2B	-0.23	TMEM38A	-0.05	SLC6A13	-0.06
NIPA1	-0.23	ZIC1	-0.05	PACS2	-0.06
NXPH2	-0.23	SULF1	-0.05	ECHDC2	-0.06
ENSMUSG(	-0.23	ADRA2A	-0.05	SCD1	-0.06
NMRAL1	-0.23	PIK3R3	-0.06	EXDL2	-0.07
C130090K2	-0.23	COL6A1	-0.06	SLC24A3	-0.07
RAPGEF4	-0.23	ETS1	-0.06	WNT4	-0.07
INA	-0.23	TTC19	-0.06	NPY1R	-0.07
TRIM59	-0.23	ZFPM2	-0.06	RYR1	-0.07
KLF7	-0.23	CDH7	-0.06	ITPKA	-0.07
PACS2	-0.23	RAB3B	-0.06	MIF4GD	-0.07
LRRN1	-0.23	RASGRF2	-0.06	FAM13C	-0.07
SKP1A	-0.23	PRSS23	-0.06	PTPRK	-0.07
TPBG	-0.23	TRIM36	-0.06	RAPGEF4	-0.07
SCD1	-0.24	ANXA2	-0.06	TRIB2	-0.07
GPR21	-0.24	TMEM19	-0.06	CAMK1G	-0.07
RASL11B	-0.24	RAI14	-0.06	IFITM3	-0.07

PADI6	-0.24	SLC2A1	-0.06	ANXA2	-0.07
ANO4	-0.24	TCF4	-0.06	NRIP3	-0.07
FOXN3	-0.24	6330503K2	-0.06	LOC10004	-0.07
LY6G6E	-0.24	NFIB	-0.06	RGS9	-0.07
SLAIN1	-0.24	NRIP3	-0.06	BHLHB5	-0.07
PPP1R14A	-0.24	ELAVL2	-0.06	ZFP810	-0.07
PDLIM2	-0.24	STAC2	-0.06	TAC1	-0.07
KIF5A	-0.25	TPBG	-0.06	FOSB	-0.07
RELL1	-0.25	GADD45A	-0.06	3110035E1	-0.07
DDAH1	-0.25	RTN4RL1	-0.06	NPTX2	-0.08
FAM134B	-0.25	MKX	-0.06	NAV1	-0.08
HTR1F	-0.26	FAM134B	-0.06	S100A11	-0.08
NTSR1	-0.26	GCNT2	-0.06	PRR13	-0.08
UAP1	-0.26	GM129	-0.06	PDYN	-0.08
VANGL2	-0.26	CADPS2	-0.06	RILPL1	-0.08
OGFRL1	-0.26	HKDC1	-0.07	EXTL3	-0.08
PCBP2	-0.26	OSBPL9	-0.07	RHBDL3	-0.08
ADAMTS4	-0.26	PHLDB1	-0.07	AI593442	-0.08
OSBPL9	-0.26	UGT8A	-0.07	DACT2	-0.08
LRFN2	-0.26	ISLR2	-0.07	D930015E(	-0.08
CRTAC1	-0.26	VASN	-0.07	KLF2	-0.08
CARHSP1	-0.26	CBLN4	-0.07	SLC2A1	-0.08
GADD45A	-0.27	ASAH2	-0.07	SERINC2	-0.08
GLTP	-0.27	NEU2	-0.07	MARCKSL1	-0.08
BCL11B	-0.27	PPM2C	-0.07	CLCN2	-0.08
LOC10004	-0.27	CAMKK1	-0.07	ZDHHC14	-0.08
D12ERTD6	-0.27	OLFML2B	-0.07	GTPBP6	-0.08
PIK3R3	-0.27	3110035E1	-0.07	MORN4	-0.08
LOC10004	-0.28	TGM2	-0.07	STARD8	-0.08
RIMS1	-0.28	THBD	-0.07	SLC2A13	-0.08
DRD1A	-0.28	ST8SIA2	-0.08	FHL1	-0.08
RHOG	-0.28	AGAP1	-0.08	CAMK2A	-0.08
KCNK13	-0.29	FKBP1A	-0.08	PHLDB1	-0.09
NOS1AP	-0.29	RGS4	-0.08	RGL1	-0.09
NFIB	-0.29	DCN	-0.08	WNT2	-0.09
PLEKHA2	-0.30	ABCA8A	-0.08	CACNB3	-0.09
ITGB4	-0.30	LOC10004(	-0.08	SULT1A1	-0.09
GAB1	-0.30	RXFP3	-0.08	HKDC1	-0.09
NRIP3	-0.30	ACTA2	-0.08	UAP1	-0.09
IL33	-0.31	LOC10004(	-0.08	TNNC1	-0.09
UNC5B	-0.31	CPNE8	-0.08	RPH3A	-0.10
PDLIM1	-0.31	ODZ4	-0.08	RTN4RL1	-0.10
PEBP1	-0.31	ELOVL6	-0.09	BHLHB2	-0.10

ADSSL1	-0.31	NAPEPLD	-0.09	EMID2	-0.10
LPGAT1	-0.31	TRF	-0.09	THBD	-0.10
FANCD2	-0.31	LRRTM2	-0.09	FBXL10	-0.10
NRP1	-0.32	SPAG5	-0.09	1300013J1	-0.10
COL6A1	-0.32	NFE2L3	-0.09	CRTAC1	-0.10
RGS9	-0.32	RELN	-0.09	HAP1	-0.10
LOC10004	-0.32	HSD17B11	-0.09	HSPA2	-0.10
GAL3ST1	-0.33	PRICKLE1	-0.09	RAMP3	-0.10
SST	-0.33	SKP1A	-0.09	PRELP	-0.10
MYL4	-0.33	LPGAT1	-0.10	ACSL5	-0.10
LOC10004	-0.33	LOC10004	-0.10	SYTL2	-0.10
LASS2	-0.33	RYR3	-0.10	UBQLN4	-0.10
GARNL3	-0.34	C130090K	-0.10	CDKN1C	-0.10
KCNA1	-0.34	PDYN	-0.10	OGN	-0.10
TMEM178	-0.34	LPL	-0.10	PCBP2	-0.10
SLC44A1	-0.34	GLRA2	-0.10	EHMT2	-0.11
CNTNAP4	-0.34	SC4MOL	-0.10	TMEM38A	-0.11
OPALIN	-0.34	CD83	-0.10	COL5A1	-0.11
ZDHHC9	-0.34	TLE4	-0.11	RXFP3	-0.11
ARHGAP2	-0.35	MMD	-0.11	DCLK1	-0.11
LOC10004	-0.36	MID1	-0.11	PROSAPIP	-0.11
SC4MOL	-0.36	EGR2	-0.11	CLCN4-2	-0.11
CNP	-0.37	CAR4	-0.11	SPOP	-0.11
NDRL	-0.37	PER2	-0.11	BMP1	-0.12
PPP1R1B	-0.37	MAPRE2	-0.11	ADCYAP1	-0.12
PLP1	-0.37	VSTM2B	-0.11	2510009E	-0.12
RAI14	-0.38	CACNG3	-0.11	CD83	-0.12
TLE4	-0.38	SLC7A4	-0.11	CAR4	-0.12
EVI2A	-0.39	IL11RA1	-0.12	WFS1	-0.12
LITAF	-0.39	RELL1	-0.12	CACNA1H	-0.12
OTX1	-0.39	LRRN1	-0.12	ANXA11	-0.12
ISLR2	-0.40	LOC10004	-0.12	NR2F6	-0.12
IGSF21	-0.41	TSPAN2	-0.12	SLC4A3	-0.12
NEFM	-0.42	CNTNAP4	-0.12	DCN	-0.13
EDG2	-0.43	PDZRN3	-0.13	TMCC2	-0.13
ENPP6	-0.43	EG434858	-0.13	ARNTL	-0.14
NDRG1	-0.46	MYO5B	-0.13	NBL1	-0.14
GLRA2	-0.46	EPHA7	-0.13	PDZRN3	-0.14
CRYAB	-0.46	ADAMTSL	-0.13	MAPRE2	-0.14
ANLN	-0.46	PBRM1	-0.13	2310021P1	-0.15
SPSB1	-0.46	WDR60	-0.13	ISLR2	-0.15
TSPAN2	-0.46	TRIM59	-0.13	MGP	-0.15
GJC2	-0.48	6330406I1	-0.14	FSTL4	-0.15

PLEKHB1	-0.48	FLT1	-0.14	AKAP8L	-0.15
SLA	-0.48	PIGP	-0.14	RIMS3	-0.15
QDPR	-0.49	COCH	-0.14	6030405A1	-0.16
ZFPM2	-0.50	GLTP	-0.14	LOC100047	-0.16
SCN4B	-0.51	CAR14	-0.14	RASGRF1	-0.16
MOBP	-0.52	NIPA1	-0.14	SLC13A4	-0.17
UGT8A	-0.53	CHN2	-0.15	RYR3	-0.17
PRSS35	-0.53	PER1	-0.15	CPNE9	-0.18
ADRA2A	-0.53	NEFM	-0.15	REEP5	-0.19
MAG	-0.56	NOL4	-0.15	HPCA	-0.19
MOG	-0.57	ARNTL	-0.15	FLT1	-0.20
SULF1	-0.57	BHLHB5	-0.15	IGF2	-0.21
MBP	-0.58	BHLHB2	-0.15	PLCH2	-0.21
SEMA5A	-0.59	RNF144A	-0.16	LOC100045	-0.21
MAL	-0.59	OGFRL1	-0.18	COL6A1	-0.22
CLDN11	-0.60	LOC100048	-0.19	CACNG3	-0.22
FA2H	-0.62	RAB5A	-0.19	VGF	-0.24
BCAS1	-0.68	ATP5F1	-0.20	4933439C2	-0.24
PRR18	-0.69	PRSS35	-0.20	B930076A0	-0.26
TRF	-0.73	PKNOX2	-0.21	FAM148C	-0.26
RPRM	-0.83	VAT1L	-0.22	C1QTNF4	-0.26
CTGF	-0.90	NDRG1	-0.24	PKNOX2	-0.30
