# Supplementary material for: Molecular laterality encodes stress susceptibility in the medial prefrontal cortex
Source: Mol Brain. 2021 Jun 14;14:92. doi: 10.1186/s13041-021-00802-w (PMC8201740; doi:10.1186/s13041-021-00802-w)
Supplement: Supplementary file 7 — Additional file 7: Table S5. Differences in the expression of the housekeeping genes, Actb(b-actin), Gapdh(glyceraldehyde-3-phosphate dehydrogenase), B2m ((b2microglobinbetween control and resilient/susceptible mice, summarized as log2FC values and p-values for each gene. [file 13041_2021_802_MOESM7_ESM.pdf]

**Supplementary Table 5**
Differences in the expression of the housekeeping genes, *Actb* (β-actin), *Gapdh(*glyceraldehyde-3-phosphate dehydrogenase), *B2m* (($b_2$ microglobin between control and resilient/susceptible mice, summarized as $log_2$ FC values and *p*-values for each gene.

| Control group | Test group | Gene | log2 fold- | t | p-value | Adjusted p-value |
|---|---|---|---|---|---|---|
| Control Left | Resilient Left | *Actb* | 0.101 | 2.148 | 0.048 | **0.263** |
| Control Left | Resilient Left | *Gapdh* | -0.030 | -0.542 | 0.596 | **0.821** |
| Control Left | Resilient Left | *B2m* | 0.121 | 2.748 | 0.015 | **0.144** |
| Control Left | Susceptible Left | *Actb* | 0.118 | 2.755 | 0.015 | **0.130** |
| Control Left | Susceptible Left | *Gapdh* | 0.076 | 1.486 | 0.158 | **0.446** |
| Control Left | Susceptible Left | *B2m* | 0.012 | 0.291 | 0.775 | **0.914** |
| Control Right | Resilient Right | *Actb* | 0.045 | 0.959 | 0.353 | **0.791** |
| Control Right | Resilient Right | *Gapdh* | -0.106 | -1.874 | 0.080 | **0.488** |
| Control Right | Resilient Right | *B2m* | -0.004 | -0.091 | 0.928 | **0.986** |
| Control Right | Susceptible Right | *Actb* | 0.146 | 3.404 | 0.004 | **0.069** |
| Control Right | Susceptible Right | *Gapdh* | -0.064 | -1.252 | 0.230 | **0.580** |
| Control Right | Susceptible Right | *B2m* | -0.116 | -3.870 | 0.001 | **0.037** |

**Normalized Avg. Expression**

| | Control Left | Control Ri | Resilient L | Resilient R | Susceptibl | Susceptible Right |
|---|---|---|---|---|---|---|
| *Gapdh* | 9076.67811 | 9743.384 | 8987.215 | 9055.841 | 9682.672 | 9326.445 |
| *Actb* | 4288.406856 | 4225.192 | 4483.022 | 4356.701 | 4536.954 | 4672.977 |
| *B2m* | 2140.939598 | 2141.417 | 2287.402 | 2135.578 | 2120.134 | 1922.615 |
