# Supplementary material for: Molecular laterality encodes stress susceptibility in the medial prefrontal cortex
Source: Mol Brain. 2021 Jun 14;14:92. doi: 10.1186/s13041-021-00802-w (PMC8201740; doi:10.1186/s13041-021-00802-w)
Supplement: Supplementary file 8 — Additional file 8: Table S6. GO analysis using GeneMANIA. Genes with leftward or rightward laterality were used as input to GeneMANIA, and the associated GO terms were reported. [file 13041_2021_802_MOESM8_ESM.pdf]

Supplementary table 6
GO analysis using GeneMANIA. Genes with leftward or rightward laterality were used as input to GeneMANIA, and the associated GO terms were reported.

GO analysis of Laterality genes

Method: GeneMANIA via Cytoscape (Differentially_expressed_genes_cutoff(adj.P.Val < 0.05))

GO_term_II	GO annotation	Q-value	Gene	Total (	Genes_in_the_input	Genes_not_in_the_input
**Laterality_to_SL**						
GO:0050804	regulation of synaptic trai	4.30E-04	17	235	Adcyap1,Bdnf,Camk2a,Egr2,Itpka,	Asic1,Camk2b,Egr1,Neurl1a,Stx1a
GO:0004683	calmodulin-dependent pr	0.00192627	6	20	Camk1g,Camk2a,Camkk1,Itpka,Pr	Camk2b
GO:0048168	regulation of neuronal syı	0.00218368	8	50	Bdnf,Camk2a,Egr2,Rasgrf1,Vgf	Camk2b,Egr1,Neurl1a
GO:0031012	extracellular matrix	0.00245384	17	292	Cdh8,Coch,Dcn,Efemp2,Fkbp1a,Lp	Col26a1,Ctgf
GO:0071248	cellular response to metal	0.00632879	8	61	Fos,Fosb,Junb,Mef2c,Mt3	Dmtn,Jun,Nptx1
GO:0071277	cellular response to calciu	0.00711211	6	29	Fos,Fosb,Junb,Mef2c	Dmtn,Jun
GO:0071241	cellular response to inorg	0.0093424	8	67	Fos,Fosb,Junb,Mef2c,Mt3	Dmtn,Jun,Nptx1
GO:0048167	regulation of synaptic pla	0.01233113	10	119	Bdnf,Camk2a,Egr2,Itpka,Rasgrf1,R	Camk2b,Egr1,Neurl1a
GO:0048306	calcium-dependent protei	0.01352877	7	52	Anxa11,Mgp,S100a11,S100a6,Tnn	Stx1a
GO:0048511	rhythmic process	0.01570336	12	184	Adcyap1,Arntl,Casp1,Dbp,Egr2,Nr	Bhlhe40
GO:0007611	learning or memory	0.01718652	12	189	Bdnf,Casp1,Igf2,Mef2c,Pde4d,Relr	Asic1,Egr1,Jun,Pde1b
GO:0044548	S100 protein binding	0.01718652	4	11	Anxa11,Anxa2,S100a11,S100a6	
GO:0010038	response to metal ion	0.0210811	11	163	Anxa11,Fos,Fosb,Junb,Mef2c,Mt3,	Camk2b,Dmtn,Jun,Nptx1
GO:0003012	muscle system process	0.02428711	13	233	Acta2,Camta2,Fkbp1a,Flt1,Gucy1a	Ctgf
GO:0050890	cognition	0.02428711	12	200	Bdnf,Casp1,Igf2,Mef2c,Pde4d,Relr	Asic1,Egr1,Jun,Pde1b
GO:0000977	RNA polymerase II regula	0.02810838	9	114	Dbp,Egr4,Junb,Mef2c,Per1,Tcf4	Egr1,Jun,Tbr1
GO:0014074	response to purine-contai	0.02810838	8	88	Casp1,Egr2,Egr4,Fkbp1a,Pde4d,Ry	Dmtn,Egr1
GO:0007623	circadian rhythm	0.02810838	8	87	Arntl,Casp1,Dbp,Nr2f6,Per1,Per2,F	Bhlhe40
GO:0000982	RNA polymerase II core p	0.03180586	9	117	Arntl,Dbp,Hey1,Mef2c,Tcf4	Bhlhe40,Egr1,Jun,Nr4a1

GO:0001012	RNA polymerase II regula	0.04010161	9	122	Dbp,Egr4,Junb,Mef2c,Per1,Tcf4	Egr1,Jun,Tbr1
GO:0010035	response to inorganic sub	0.04010161	13	252	Anxa11,Fos,Fosb,Junb,Mef2c,Mt3,	Camk2b,Dmtn,Jun,Nptx1,Nr4a1
GO:0008021	synaptic vesicle	0.0427941	8	98	Bdnf,Mt3,Npy1r,Otof,Sept5,Sphk1	Cdk16,Stx1a
GO:0000976	transcription regulatory re	0.0427941	10	154	Dbp,Egr4,Junb,Mef2c,Per1,Tcf4,Zf	Egr1,Jun,Tbr1
GO:0006936	muscle contraction	0.0427941	11	188	Acta2,Fkbp1a,Flt1,Gucy1a3,Npy1r,	Ctgf
GO:0005184	neuropeptide hormone ac	0.0427941	4	16	Adcyap1,Cck,Pdyn,Vgf	
GO:0030136	clathrin-coated vesicle	0.0427941	10	157	Bdnf,Lyz2,Mt3,Npy1r,Otof,Sept5,S	Cdk16,Lyz1,Stx1a
GO:0016358	dendrite development	0.04554216	10	159	Bdnf,Btbd3,Cobl,Igsf9,Itpka,Reln,S	Camk2b,Neurl1a,Ngef
GO:0051592	response to calcium ion	0.04554216	7	74	Anxa11,Fos,Fosb,Junb,Mef2c	Dmtn,Jun
GO:0060998	regulation of dendritic sp	0.05336543	5	33	Itpka,Reln	Camk2b,Neurl1a,Ngef
GO:0032922	circadian regulation of ge	0.0591729	4	18	Arntl,Per1,Prkg2	Bhlhe40
GO:0060999	positive regulation of den	0.07177436	4	19	Itpka,Reln	Camk2b,Neurl1a
GO:0071320	cellular response to cAMF	0.07434673	5	36	Egr2,Egr4,Pde4d	Dmtn,Egr1
GO:0006937	regulation of muscle cont	0.08261317	8	112	Flt1,Gucy1a3,Pde4d,Ryr1,Scn3b,Sp	Ctgf
GO:0010975	regulation of neuron proj	0.08261317	13	286	Adcyap1,Cobl,Hap1,Itpka,Mt3,Rel	Camk2b,Neurl1a,Ngef,Tbr1
GO:0061001	regulation of dendritic sp	0.08261317	4	20	Itpka,Reln	Camk2b,Ngef
GO:0001077	RNA polymerase II core p	0.08575635	7	85	Arntl,Dbp,Mef2c,Tcf4	Egr1,Jun,Nr4a1
GO:0035914	skeletal muscle cell differe	0.09424042	6	61	Bcl9l,Egr2,Fos,Mef2c	Egr1,Nr4a1
GO:0090257	regulation of muscle syste	0.09849911	9	148	Flt1,Gucy1a3,Mef2c,Pde4d,Ryr1,Sc	Ctgf

Laterality_to_SR

GO:0007272	ensheathment of neurons	2.55E-07	13	90	Cldn11,Fa2h,Gal3st1,Mal,Mbp,Mtr	Myrf,Ndrg1,Nkx6-2,Olig2
GO:0008366	axon ensheathment	2.55E-07	13	90	Cldn11,Fa2h,Gal3st1,Mal,Mbp,Mtr	Myrf,Ndrg1,Nkx6-2,Olig2
GO:0042552	myelination	2.02E-06	12	88	Fa2h,Gal3st1,Mal,Mbp,Mtmr2,Plp	Myrf,Ndrg1,Nkx6-2,Olig2
GO:0033267	axon part	5.24E-05	14	173	Adora1,Chrm2,Drd1a,Grik1,Ina,Kc	Aatk,Ermn,Nefh
GO:0019228	neuronal action potential	7.05E-05	10	78	Cldn11,Drd1a,Gal3st1,Mal,Mbp,Pl	Myrf,Nkx6-2,Olig2
GO:0001508	action potential	7.87E-05	12	128	Cldn11,Drd1a,Gal3st1,Mal,Mbp,Pl	Myrf,Nkx6-2,Olig2
GO:0043209	myelin sheath	1.21E-04	8	46	Cnp,Gjc2,Mag,Mbp,Pllp,Plp1,Tspa	Ermn
GO:0042063	gliogenesis	2.85E-04	13	179	Drd1a,Fa2h,Fgf10,Nfib,Plp1,Sox5,T	Gfap,Myrf,Ndrg1,Nkx6-2,Olig2,Sox10

GO:0010001	glial cell differentiation	2.85E-04	12	148	Drd1a,Fa2h,Fgf10,Nfib,Plp1,Sox5	Gfap,Myrf,Ndrg1,Nkx6-2,Olig2,Sox10
GO:0060053	neurofilament cytoskeletc	0.01019012	4	11	Ina,Nefm,Nrp1	Nefh
GO:0048709	oligodendrocyte different	0.01142335	7	62	Fa2h,Plp1,Sox5	Myrf,Nkx6-2,Olig2,Sox10
GO:0021782	glial cell development	0.01493911	7	66	Drd1a,Fa2h,Plp1	Gfap,Myrf,Ndrg1,Nkx6-2
GO:0031345	negative regulation of cel	0.01493911	8	92	Inppl1,Nrp1,Plxnb3,Sema3f,Sema!	Aatk,Gfap,Lpar1
GO:0032291	axon ensheathment in cei	0.01935836	4	14	Fa2h,Plp1	Myrf,Nkx6-2
GO:0022010	central nervous system m	0.01935836	4	14	Fa2h,Plp1	Myrf,Nkx6-2
GO:0050804	regulation of synaptic trai	0.01935836	12	235	Adora1,Cplx2,Drd1a,Grik1,Neto1,I	Gfap,S100b
GO:0010975	regulation of neuron proj	0.02758903	13	286	Inppl1,Islr2,Nefm,Nrp1,Rapgef4,Se	Aatk,Gfap,Klk6,Lpar1,Rnd2,Tenm3
GO:0048169	regulation of long-term n	0.03157084	5	32	Drd1a,Grik1,Neto1,Rims1	S100b
GO:0060560	developmental growth inv	0.03873472	9	143	Dclk1,Fgf10,Islr2,Nrp1,Sema3f,Ser	Aatk,Rnd2
GO:0001505	regulation of neurotransm	0.05743738	8	118	Cadps2,Cplx2,Pebp1,Rab3b,Rims1	Cplx1,Gfap,Vamp1
GO:0048167	regulation of synaptic pla	0.05743738	8	119	Adora1,Cplx2,Drd1a,Grik1,Neto1,I	Gfap,S100b
GO:0050769	positive regulation of neu	0.05743738	9	153	Islr2,Nrp1,Sema5a,Trf	Gfap,Nkx6-2,Olig2,Rnd2,Sox10
GO:0061387	regulation of extent of ce	0.06262494	6	62	Islr2,Nrp1,Sema3f,Sema5a	Aatk,Rnd2
GO:0071526	semaphorin-plexin signali	0.07051721	4	21	Nrp1,Plxnb3,Sema3f,Sema5a	
GO:0030517	negative regulation of axo	0.08201655	4	22	Nrp1,Sema3f,Sema5a	Aatk
GO:0048521	negative regulation of bel	0.08429841	5	42	Adora1,Drd1a,Nrp1,Sema3f,Sema5a	
GO:0014015	positive regulation of glic	0.09102638	5	43	Trf	Gfap,Nkx6-2,Olig2,Sox10

Laterality_to_SR

	log2									
ensheathme	sl/cl	sr/cr	rl/cl	rr/cr	cl	cr	rl	rr	sl	sr
Cldn11	-0.4968415	0.24831513	0.21596379	0.29303865	12.879171	12.7300697	13.0928128	13.0230296	12.3798835	12.9790939
Fa2h	-0.4324396	0.35020092	0.24544011	0.34836525	10.1265214	9.96429911	10.3727468	10.3126718	9.69560386	10.3139678
Gal3st1	-0.1441335	0.19409389	0.07424831	0.06347833	8.22833122	8.21767388	8.30091658	8.27926268	8.08255917	8.41034047
Mal	-0.640986	0.12114878	0.08103634	0.11376356	10.3322939	10.1622049	10.4143046	10.2791568	9.69166826	10.2837299
Mbp	-0.4973675	0.2120399	0.11258043	0.15414523	12.5755392	12.4452558	12.6871393	12.5994269	12.0773827	12.6574414
Mtmr2	-0.1318853	-0.0078268	-0.0945223	-0.0751252	7.98232154	8.0076583	7.88776747	7.93324288	7.85043985	8.00038793
Plp1	-0.4856655	-0.005598	-0.0984382	0.02850332	13.5304486	13.4231716	13.427864	13.4550626	13.0447593	13.4176123
Trf	-0.4485447	0.32620563	0.28403745	0.41163899	10.340288	10.2956684	10.6207763	10.7061126	9.88974048	10.6202687
Ugt8a	-0.5260096	0.11986226	-0.0144965	0.16680927	9.41500408	9.29987405	9.40047989	9.46654771	8.8898202	9.41937231

myelination	sl/cl	sr/cr	rl/cl	rr/cr	cl	cr	rl	rr	sl	sr
Fa2h	-0.4324396	0.35020092	0.24544011	0.34836525	10.1265214	9.96429911	10.3727468	10.3126718	9.69560386	10.3139678
Gal3st1	-0.1441335	0.19409389	0.07424831	0.06347833	8.22833122	8.21767388	8.30091658	8.27926268	8.08255917	8.41034047
Mal	-0.640986	0.12114878	0.08103634	0.11376356	10.3322939	10.1622049	10.4143046	10.2791568	9.69166826	10.2837299
Mbp	-0.4973675	0.2120399	0.11258043	0.15414523	12.5755392	12.4452558	12.6871393	12.5994269	12.0773827	12.6574414
Mtmr2	-0.1318853	-0.0078268	-0.0945223	-0.0751252	7.98232154	8.0076583	7.88776747	7.93324288	7.85043985	8.00038793
Plp1	-0.4856655	-0.005598	-0.0984382	0.02850332	13.5304486	13.4231716	13.427864	13.4550626	13.0447593	13.4176123
Trf	-0.4485447	0.32620563	0.28403745	0.41163899	10.340288	10.2956684	10.6207763	10.7061126	9.88974048	10.6202687
Ugt8a	-0.5260096	0.11986226	-0.0144965	0.16680927	9.41500408	9.29987405	9.40047989	9.46654771	8.8898202	9.41937231

axon part	sl/cl	sr/cr	rl/cl	rr/cr	cl	cr	rl	rr	sl	sr
Adora1	-0.1338236	0.1003699	0.05985892	0.05416839	9.33627468	9.31955509	9.3952606	9.37412175	9.20128855	9.42024795
Chrm2	-0.0813274	0.17318973	0.01679958	0.05453759	7.54162009	7.43962265	7.55818603	7.49460942	7.46018545	7.6129532
Drd1a	-0.1765097	0.21604001	0.23741997	0.31572839	8.86725015	8.75885329	9.10488409	9.0704584	8.69063002	8.97086436
Grik1	-0.072822	0.23120946	0.02076328	0.10541359	8.83170475	8.7096613	8.85245319	8.81509304	8.76002423	8.9421728
Ina	-0.2731247	-0.0694545	-0.1171084	-0.2991165	10.6803309	10.7085568	10.5643238	10.4078388	10.4077866	10.6389384

Kcna1	-0.4424686	0.08849535	-0.3137182	-0.1077382	10.2156711	10.0233773	9.90217433	9.91586804	9.77487177	10.1121415
Mag	-0.5023372	0.13825412	0.23783388	0.19952505	11.129455	11.0432527	11.3675842	11.2440043	10.6292264	11.1859215
Mbp	-0.4973675	0.2120399	0.11258043	0.15414523	12.5755392	12.4452558	12.6871393	12.5994269	12.0773827	12.6574414
Nefm	-0.6966246	-0.1205161	-0.7623033	-0.4588159	10.4736389	10.3194685	9.71999434	9.8680906	9.77961686	10.1984458
Nrp1	-0.1900102	0.21172684	0.05960549	0.11234177	9.85418363	9.76844352	9.91360028	9.88126168	9.66545395	9.98046094
Pebp1	-0.2176271	0.09540048	-0.1432215	-0.1051665	9.67634237	9.67640452	9.53433882	9.56918498	9.45985269	9.77031938

action poten	sl/cl	sr/cr	rl/cl	rr/cr	cl	cr	rl	rr	sl	sr
Cldn11	-0.4968415	0.24831513	0.21596379	0.29303865	12.879171	12.7300697	13.0928128	13.0230296	12.3798835	12.9790939
Drd1a	-0.1765097	0.21604001	0.23741997	0.31572839	8.86725015	8.75885329	9.10488409	9.0704584	8.69063002	8.97086436
Gal3st1	-0.1441335	0.19409389	0.07424831	0.06347833	8.22833122	8.21767388	8.30091658	8.27926268	8.08255917	8.41034047
Mal	-0.640986	0.12114878	0.08103634	0.11376356	10.3322939	10.1622049	10.4143046	10.2791568	9.69166826	10.2837299
Mbp	-0.4973675	0.2120399	0.11258043	0.15414523	12.5755392	12.4452558	12.6871393	12.5994269	12.0773827	12.6574414
Plp1	-0.4856655	-0.005598	-0.0984382	0.02850332	13.5304486	13.4231716	13.427864	13.4550626	13.0447593	13.4176123
Scn4b	-0.4978921	0.06151567	-0.1730431	-0.3446934	9.84566841	9.80003476	9.67274781	9.45229825	9.34966899	9.85907937
Tac1	-0.0055481	0.0888648	0.13727475	0.03991981	8.96505961	9.03926907	9.10231572	9.07918394	8.95944831	9.12897664
Ugt8a	-0.5260096	0.11986226	-0.0144965	0.16680927	9.41500408	9.29987405	9.40047989	9.46654771	8.8898202	9.41937231

myelin sheat	sl/cl	sr/cr	rl/cl	rr/cr	cl	cr	rl	rr	sl	sr
Cnp	-0.3646252	0.13276521	0.16825952	0.10568009	9.2598682	9.13205903	9.42970538	9.23671832	8.89672933	9.26428733
Gjc2	-0.5293398	0.09736653	-0.0465779	0.07902822	8.58174596	8.42882303	8.53286329	8.5157479	8.0500122	8.52632836
Mag	-0.5023372	0.13825412	0.23783388	0.19952505	11.129455	11.0432527	11.3675842	11.2440043	10.6292264	11.1859215
Mbp	-0.4973675	0.2120399	0.11258043	0.15414523	12.5755392	12.4452558	12.6871393	12.5994269	12.0773827	12.6574414
Pllp	-0.2119891	0.11572973	0.05951065	-0.0180157	8.50748681	8.38544897	8.56730289	8.36825376	8.29587932	8.50392392
Plp1	-0.4856655	-0.005598	-0.0984382	0.02850332	13.5304486	13.4231716	13.427864	13.4550626	13.0447593	13.4176123
Tspan2	-0.4886947	0.1170349	-0.0599009	0.20788379	9.71959636	9.57584744	9.66097785	9.78437388	9.23154056	9.69267373

gliogenesis	sl/cl	sr/cr	rl/cl	rr/cr	cl	cr	rl	rr	sl	sr
Drd1a	-0.1765097	0.21604001	0.23741997	0.31572839	8.86725015	8.75885329	9.10488409	9.0704584	8.69063002	8.97086436

Fa2h	-0.4324396	0.35020092	0.24544011	0.34836525	10.1265214	9.96429911	10.3727468	10.3126718	9.69560386	10.3139678
Fgf10	-0.0631123	0.17801331	0.13697511	0.15079431	8.05467223	8.02717273	8.19146421	8.17851742	7.99152521	8.20529331
Nfib	-0.2392624	0.12221731	0.09876637	0.2289528	10.8983554	10.8273665	10.9957295	11.0567948	10.6590926	10.9533488
Plp1	-0.4856655	-0.005598	-0.0984382	0.02850332	13.5304486	13.4231716	13.427864	13.4550626	13.0447593	13.4176123
Sox5	-0.1310938	0.22812017	0.0308673	0.12802004	8.74566103	8.60606734	8.77651582	8.73340211	8.61447431	8.83356156
Trf	-0.4485447	0.32620563	0.28403745	0.41163899	10.340288	10.2956684	10.6207763	10.7061126	9.88974048	10.6202687

negative reg	sl/cl	sr/cr	rl/cl	rr/cr	cl	cr	rl	rr	sl	sr
Inppl1	-0.1294311	0.03230877	0.03186388	0.00817119	8.92350402	8.90773747	8.95555856	8.91591735	8.79442635	8.94023305
Nrp1	-0.1900102	0.21172684	0.05960549	0.11234177	9.85418363	9.76844352	9.91360028	9.88126168	9.66545395	9.98046094
Plxnb3	-0.1652361	0.09823752	0.0882861	0.01284341	7.60214255	7.55765347	7.69017826	7.57054015	7.43724566	7.65538275
Sema3f	0.02672611	0.17885969	0.13011803	0.12813663	7.774697	7.77641174	7.90585245	7.90378746	7.80180671	7.95476837
Sema5a	-0.1510265	0.57078098	0.25644608	0.38060153	9.65494491	9.5212659	9.91225324	9.90194337	9.50496153	10.0927684

Laterality_to_SL

	log2									
regulation o	sl/cl	sr/cr	rl/cl	rr/cr	cl	cr	rl	rr	sl	sr
Adcyap1	0.09652559	-0.2897159	-0.068392	-0.2877376	8.66575308	8.78223906	8.59374472	8.49353133	8.7610087	8.49227686
Bdnf	0.12760208	-0.1694664	-0.0357513	-0.0685744	8.66874524	8.67647392	8.63305174	8.60780939	8.79788667	8.50798419
Camk2a	0.12882427	-0.1567393	-0.0339431	-0.2757515	11.2437229	11.3286758	11.209452	11.0528715	11.3724021	11.1729444
Egr2	0.1205971	-0.2925413	-0.2422923	-0.0516785	8.42609713	8.34323047	8.18358463	8.29218916	8.54643106	8.05074068
Itpka	0.26070549	-0.0256763	0.00884585	-0.1581371	11.1199921	11.1890521	11.1286664	11.0297254	11.3800044	11.1622499
Mef2c	-0.0300646	-0.1470211	-0.1313618	0.01062349	12.3353297	12.1664706	12.2038497	12.177467	12.3054209	12.0195774
Rasgrf1	0.11672462	-0.1812076	-0.0345787	-0.1620232	12.398056	12.5613233	12.3635828	12.3992456	12.5152882	12.3800608
Reln	0.14123147	-0.2556522	-0.1338787	-0.0846739	10.6569021	10.6959541	10.5223473	10.6130308	10.7981784	10.4407711
Rims3	0.22824467	-0.2805787	0.03815738	-0.2989062	11.0699563	11.2236609	11.1078499	10.9242068	11.2986932	10.9486058
Slc1a3	-0.0506126	-0.2791044	-0.0636584	-0.1854076	12.6956356	12.7280637	12.6313656	12.5419685	12.6447689	12.4481357
Sphk1	0.03945189	-0.1117222	-0.015064	-0.0971277	7.51933983	7.53441056	7.50431706	7.43740312	7.55908468	7.42314252
Vgf	0.13758889	-0.3677197	0.00520092	-0.2064293	11.7571927	11.9973946	11.7639741	11.7908362	11.8946736	11.6295875

calmodulin-c	sl/cl	sr/cr	rl/cl	rr/cr	cl	cr	rl	rr	sl	sr
Camk1g	0.10612734	-0.1024396	0.05001433	-0.0560137	7.65068212	7.72306371	7.70072602	7.66697336	7.75690015	7.62076028
Camk2a	0.12882427	-0.1567393	-0.0339431	-0.2757515	11.2437229	11.3286758	11.209452	11.0528715	11.3724021	11.1729444
Camkk1	0.08680031	-0.0902278	0.0113367	0.07996236	9.60781445	9.61052248	9.61966235	9.69061379	9.69509732	9.52292868
Itpka	0.26070549	-0.0256763	0.00884585	-0.1581371	11.1199921	11.1890521	11.1286664	11.0297254	11.3800044	11.1622499
Pnck	0.27751316	-0.0042949	0.18244772	0.14282146	9.26929273	9.31840743	9.45072453	9.46204834	9.54661863	9.31408364

regulation o	sl/cl	sr/cr	rl/cl	rr/cr	cl	cr	rl	rr	sl	sr
Bdnf	0.12760208	-0.1694664	-0.0357513	-0.0685744	8.66874524	8.67647392	8.63305174	8.60780939	8.79788667	8.50798419
Camk2a	0.12882427	-0.1567393	-0.0339431	-0.2757515	11.2437229	11.3286758	11.209452	11.0528715	11.3724021	11.1729444
Egr2	0.1205971	-0.2925413	-0.2422923	-0.0516785	8.42609713	8.34323047	8.18358463	8.29218916	8.54643106	8.05074068
Rasgrf1	0.11672462	-0.1812076	-0.0345787	-0.1620232	12.398056	12.5613233	12.3635828	12.3992456	12.5152882	12.3800608
Vgf	0.13758889	-0.3677197	0.00520092	-0.2064293	11.7571927	11.9973946	11.7639741	11.7908362	11.8946736	11.6295875

extracellular	sl/cl	sr/cr	rl/cl	rr/cr	cl	cr	rl	rr	sl	sr
Cdh8	0.08893948	-0.2012953	-0.1547624	-0.1534314	8.27062641	8.28853421	8.11600179	8.13509101	8.35970332	8.08726355
Coch	-0.0565005	-0.2386535	-0.3278033	-0.0738228	7.92029158	7.80496381	7.59223842	7.7324557	7.86379483	7.56605329
Dcn	0.26647432	-0.327092	0.05421368	0.00448201	7.79608504	7.9219823	7.85031762	7.92638793	8.06354737	7.5948231
Efemp2	0.09344833	-0.0733703	0.05995699	0.04798374	7.66117521	7.6710178	7.72120993	7.71864232	7.75492436	7.59835253
Fkbp1a	0.19804557	-0.0118103	-0.0036162	0.04749424	10.9504141	10.9743669	10.9454352	11.0211114	11.1473892	10.9623845
Lpl	0.31549974	-0.031869	0.08601434	0.1983453	9.05054192	9.03880789	9.13658353	9.23715674	9.36590902	9.0079158
Mfge8	0.31055336	-0.0100499	0.30838402	0.06177415	11.283777	11.3305338	11.5930208	11.3939321	11.5948333	11.3211435
Mgp	0.40483863	-0.0911722	0.26417314	-0.0223393	8.23126739	8.37753607	8.4950024	8.35543043	8.63611336	8.28691995
Ogn	0.21950223	-0.2245187	0.00579398	-0.0885217	7.34917028	7.45345781	7.3530724	7.36487056	7.56796729	7.22894316
Prelp	0.12294417	-0.3277017	0.0544473	-0.1362137	8.39816175	8.50077478	8.45350326	8.36535998	8.52143349	8.17293199
Reln	0.14123147	-0.2556522	-0.1338787	-0.0846739	10.6569021	10.6959541	10.5223473	10.6130308	10.7981784	10.4407711
Serpinf1	0.17639379	-0.0973705	0.12151625	0.06156399	7.97677732	7.98134704	8.09818411	8.04308175	8.1533511	7.88447542
Slc1a3	-0.0506126	-0.2791044	-0.0636584	-0.1854076	12.6956356	12.7280637	12.6313656	12.5419685	12.6447689	12.4481357
Tgm2	0.13518367	-0.0913342	-0.0691249	0.04673249	7.98714543	7.94366064	7.91688814	7.99118453	8.12131822	7.85272549
Wnt4	0.17876613	-0.0788835	0.04648612	-0.0337847	7.7148636	7.78208308	7.7613498	7.74879927	7.8942101	7.70323475

cellular respo	sl/cl	sr/cr	rl/cl	rr/cr	cl	cr	rl	rr	sl	sr
Fos	-0.2624533	-0.455578	-0.4137438	-0.283863	10.0623312	9.97857408	9.64783855	9.69475951	9.80032217	9.52333992
Fosb	0.28143187	-0.1159542	0.09832603	0.01068175	8.28905709	8.36338796	8.38787577	8.37213876	8.57150972	8.24726027
Junb	0.18004256	-0.1798274	0.01917093	0.02973117	9.40098938	9.38523326	9.42808086	9.41736685	9.58202471	9.20586532
Mef2c	-0.0300646	-0.1470211	-0.1313618	0.01062349	12.3353297	12.1664706	12.2038497	12.177467	12.3054209	12.0195774
Mt3	0.0532317	-0.0195133	0.08144431	-0.01045	10.4170777	10.3415544	10.4981682	10.3310949	10.4696196	10.3219908

regulation of	sl/cl	sr/cr	rl/cl	rr/cr	cl	cr	rl	rr	sl	sr
Bdnf	0.12760208	-0.1694664	-0.0357513	-0.0685744	8.66874524	8.67647392	8.63305174	8.60780939	8.79788667	8.50798419
Camk2a	0.12882427	-0.1567393	-0.0339431	-0.2757515	11.2437229	11.3286758	11.209452	11.0528715	11.3724021	11.1729444
Egr2	0.1205971	-0.2925413	-0.2422923	-0.0516785	8.42609713	8.34323047	8.18358463	8.29218916	8.54643106	8.05074068

Itpka	0.26070549	-0.0256763	0.00884585	-0.1581371	11.1199921	11.1890521	11.1286664	11.0297254	11.3800044	11.1622499
Rasgrf1	0.11672462	-0.1812076	-0.0345787	-0.1620232	12.398056	12.5613233	12.3635828	12.3992456	12.5152882	12.3800608
Reln	0.14123147	-0.2556522	-0.1338787	-0.0846739	10.6569021	10.6959541	10.5223473	10.6130308	10.7981784	10.4407711
Vgf	0.13758889	-0.3677197	0.00520092	-0.2064293	11.7571927	11.9973946	11.7639741	11.7908362	11.8946736	11.6295875

calcium-depe	sl/cl	sr/cr	rl/cl	rr/cr	cl	cr	rl	rr	sl	sr
Anxa11	0.14754893	-0.2194118	-0.0840244	-0.2185909	9.59022805	9.71026	9.50681787	9.49206641	9.74007564	9.49202712
Mgp	0.40483863	-0.0911722	0.26417314	-0.0223393	8.23126739	8.37753607	8.4950024	8.35543043	8.63611336	8.28691995
S100a11	0.26329042	-0.0659372	0.18417631	0.09258529	7.81770584	7.89292558	8.00167417	7.98643866	8.08096493	7.82677051
S100a6	0.13499227	-0.0234087	0.11044799	-0.0541073	7.91541976	7.93286231	8.02615886	7.87877074	8.0515614	7.90949127
Tnnc1	0.08188474	-0.7882321	-0.1829694	-0.2306899	9.28487368	9.3788124	9.10180408	9.14995107	9.36871187	8.59068756
Wfs1	0.45320352	-0.4895689	0.23021222	-0.1297208	8.85948358	8.97696769	9.08969445	8.8477454	9.31488397	8.48819898

rhythmic pro	sl/cl	sr/cr	rl/cl	rr/cr	cl	cr	rl	rr	sl	sr
Adcyap1	0.09652559	-0.2897159	-0.068392	-0.2877376	8.66575308	8.78223906	8.59374472	8.49353133	8.7610087	8.49227686
Arntl	0.00140087	-0.3659316	-0.2515586	-0.2367656	8.90413201	9.0422237	8.65415333	8.80469783	8.90799548	8.67591143
Casp1	0.07762033	-0.1220736	-0.1004216	-0.0740955	7.78369877	7.7706169	7.68340443	7.69609256	7.86155825	7.64740887
Dbp	0.05722823	-0.0895153	0.01755925	0.1417314	11.1938134	11.0715951	11.2106771	11.2134315	11.2490945	10.9833418
Egr2	0.1205971	-0.2925413	-0.2422923	-0.0516785	8.42609713	8.34323047	8.18358463	8.29218916	8.54643106	8.05074068
Nr2f6	0.28849364	-0.0933661	0.22953673	0.03265728	8.57882937	8.70113769	8.80782207	8.73311686	8.86644464	8.60721369
Per1	0.09723122	-0.2265054	-0.0931869	0.00468232	9.50895917	9.55710719	9.41464555	9.56167939	9.60417626	9.3302759
Per2	0.20516066	-0.0965107	0.14376313	0.22634882	9.88853196	9.91288898	10.0293073	10.138607	10.0905698	9.81561375
Prkg2	0.18333267	-0.0981914	0.19671833	0.0834756	7.94434734	7.96768602	8.14183608	8.05161357	8.12919738	7.8700331
Schip1	0.06648906	-0.2007781	0.00548063	-0.0263123	11.6485113	11.7078905	11.6536944	11.678871	11.7146621	11.5048699
Vgf	0.13758889	-0.3677197	0.00520092	-0.2064293	11.7571927	11.9973946	11.7639741	11.7908362	11.8946736	11.6295875

learning or n	sl/cl	sr/cr	rl/cl	rr/cr	cl	cr	rl	rr	sl	sr
Bdnf	0.12760208	-0.1694664	-0.0357513	-0.0685744	8.66874524	8.67647392	8.63305174	8.60780939	8.79788667	8.50798419
Casp1	0.07762033	-0.1220736	-0.1004216	-0.0740955	7.78369877	7.7706169	7.68340443	7.69609256	7.86155825	7.64740887

Igf2	0.3954169	-0.3239219	0.24960236	-0.0437366	9.35187174	9.55812134	9.60086189	9.51780487	9.74699395	9.23515832
Mef2c	-0.0300646	-0.1470211	-0.1313618	0.01062349	12.3353297	12.1664706	12.2038497	12.177467	12.3054209	12.0195774
Pde4d	0.10786678	-0.0744097	0.05068213	-0.0344082	7.53192062	7.55779704	7.58317474	7.52384994	7.63971659	7.48450477
Reln	0.14123147	-0.2556522	-0.1338787	-0.0846739	10.6569021	10.6959541	10.5223473	10.6130308	10.7981784	10.4407711
Serpinf1	0.17639379	-0.0973705	0.12151625	0.06156399	7.97677732	7.98134704	8.09818411	8.04308175	8.1533511	7.88447542
Vip	0.20402177	-0.3278889	-0.0709145	-0.0893611	10.5107037	10.5211764	10.4403917	10.4295802	10.7158088	10.1904719

S100 protein	sl/cl	sr/cr	rl/cl	rr/cr	cl	cr	rl	rr	sl	sr
Anxa11	0.14754893	-0.2194118	-0.0840244	-0.2185909	9.59022805	9.71026	9.50681787	9.49206641	9.74007564	9.49202712
Anxa2	0.28829742	-0.0014408	0.18054056	0.16720577	8.11126032	8.1842222	8.29169149	8.35152042	8.39976386	8.18282751
S100a11	0.26329042	-0.0659372	0.18417631	0.09258529	7.81770584	7.89292558	8.00167417	7.98643866	8.08096493	7.82677051
S100a6	0.13499227	-0.0234087	0.11044799	-0.0541073	7.91541976	7.93286231	8.02615886	7.87877074	8.0515614	7.90949127

response to	sl/cl	sr/cr	rl/cl	rr/cr	cl	cr	rl	rr	sl	sr
Anxa11	0.14754893	-0.2194118	-0.0840244	-0.2185909	9.59022805	9.71026	9.50681787	9.49206641	9.74007564	9.49202712
Fos	-0.2624533	-0.455578	-0.4137438	-0.283863	10.0623312	9.97857408	9.64783855	9.69475951	9.80032217	9.52333992
Fosb	0.28143187	-0.1159542	0.09832603	0.01068175	8.28905709	8.36338796	8.38787577	8.37213876	8.57150972	8.24726027
Junb	0.18004256	-0.1798274	0.01917093	0.02973117	9.40098938	9.38523326	9.42808086	9.41736685	9.58202471	9.20586532
Mef2c	-0.0300646	-0.1470211	-0.1313618	0.01062349	12.3353297	12.1664706	12.2038497	12.177467	12.3054209	12.0195774
Mt3	0.0532317	-0.0195133	0.08144431	-0.01045	10.4170777	10.3415544	10.4981682	10.3310949	10.4696196	10.3219908
Tnnc1	0.08188474	-0.7882321	-0.1829694	-0.2306899	9.28487368	9.3788124	9.10180408	9.14995107	9.36871187	8.59068756

cognition	sl/cl	sr/cr	rl/cl	rr/cr	cl	cr	rl	rr	sl	sr
Bdnf	0.12760208	-0.1694664	-0.0357513	-0.0685744	8.66874524	8.67647392	8.63305174	8.60780939	8.79788667	8.50798419
Casp1	0.07762033	-0.1220736	-0.1004216	-0.0740955	7.78369877	7.7706169	7.68340443	7.69609256	7.86155825	7.64740887
Igf2	0.3954169	-0.3239219	0.24960236	-0.0437366	9.35187174	9.55812134	9.60086189	9.51780487	9.74699395	9.23515832
Mef2c	-0.0300646	-0.1470211	-0.1313618	0.01062349	12.3353297	12.1664706	12.2038497	12.177467	12.3054209	12.0195774
Pde4d	0.10786678	-0.0744097	0.05068213	-0.0344082	7.53192062	7.55779704	7.58317474	7.52384994	7.63971659	7.48450477
Reln	0.14123147	-0.2556522	-0.1338787	-0.0846739	10.6569021	10.6959541	10.5223473	10.6130308	10.7981784	10.4407711

Serpinf1	0.17639379	-0.0973705	0.12151625	0.06156399	7.97677732	7.98134704	8.09818411	8.04308175	8.1533511	7.88447542
Vip	0.20402177	-0.3278889	-0.0709145	-0.0893611	10.5107037	10.5211764	10.4403917	10.4295802	10.7158088	10.1904719
