# Supplementary material for: Molecular laterality encodes stress susceptibility in the medial prefrontal cortex
Source: Mol Brain. 2021 Jun 14;14:92. doi: 10.1186/s13041-021-00802-w (PMC8201740; doi:10.1186/s13041-021-00802-w)
Supplement: Supplementary file 9 — Additional file 9: Table S7. Summary of statistics [file 13041_2021_802_MOESM9_ESM.pdf]

Supplementary Table 7. Summary of statistics

Figure	Test	Comparison	Sample number	Stat	p-value	Summary
Figure 2a	independent samples t-test	susceptible left vs. susceptible right	n = 9	t_{16} = -8.324	p = 0.000000330	**
Figure 2b	independent samples t-test	susceptible left vs. susceptible right	n = 8	t_{14} = -7.235	p = 0.00000432	**
Figure 2c	independent samples t-test	susceptible left vs. susceptible right	n = 9	t_{16} = -6.756	p = 0.00000462	**
Figure 2d	independent samples t-test	susceptible left vs. susceptible right	n = 7	t_{12} = -6.114	p = 0.0000522	**
Figure 2e	independent samples t-test	susceptible left vs. susceptible right	n = 5	t_{8} = 8.665	p = 0.0000245	**
Figure 2f	independent samples t-test	susceptible left vs. susceptible right	n = 8	*t14* = 6.096	p = 0.0000276	**
Figure 2g	independent samples t-test	susceptible left vs. susceptible right	n = 7	t_{12} = 2.844	p = 0.0148	*
Figure 2h	independent samples t-test	susceptible left vs. susceptible right	n = 12	t_{22} = 8.454	p = 0.000000023[illegible]	**
Figure 3a	Kruskal-Wallis One Way Analysis of Variance on Ranks	control vs. resilient vs. susceptible	control = 5, resilient = 5, rusceptible = 5	H = 9.620, df = 2	p = 0.008	**
	Turkey test post-hoc			between control and susceptible, *p < 0.05, between control and resilient, p > 0.05. between resilient and susceptible, *p < 0.05,		
Figure 3b	two-way ANOVA	Group: control vs. resilient vs. susceptible	control = 8, resilient = 7, susceptible =7	F(2, 38) = 14.445	p < 0.001	**
		Lateraility : Left vs. Right		F(1, 38) = 3.634	p = 0.064	NS
		Group X Laterailty		F(2, 38) = 4.489	p = 0.018	*

	Holm-sidak post-hoc analysis			laterality within susceptible **p = 0.002, laterality within resilient p = 0.357; within left mPFC, control and resilient, ***p* < 0.001, control and susceptible, *p* = 0.120, resilient and susceptible, **p* = 0.016; within right mPFC, control and resilient, **p* = 0.025, control and susceptible, ***p* < 0.001, resilient and susceptible, *p* = 0.166		
Figure 3d	independent samples t-test	scramble shRNA vs. CTGF shRNA	scrambled = 3, CTGF shRNA = 3	t_4 = 2.850	*p* = 0.0464	*
Figure 3e	two-way RM ANOVA	Group: non-stress vs. stress	non-stress = 14, stress = 16	F(1, 28) = 1.731	*p* = 0.199	NS
		Repeated trial: No Target vs. Target		F(1, 28) = 7.721	*p* = 0.010	*
		Stress X Target interaction		F(1, 28) = 5.126	*p* = 0.032	*
	Holm-sidak post-hoc analysis			within non-stressed, effect of target, ***p* = 0.002; within stressed, effect of target, *p* = 0.709; within non-target, effect of stress, *p* = 0.739, within target, effect of stress, **p* = 0.019,		
Figure 3f	two-way RM ANOVA	Group: non-stress vs. stress	non-stress = 13, stress = 17	F(1, 28) = 2.514	*p* = 0.124	NS
		Repeated trial: No Target vs. Target		F(1, 28) = 20.319	*p* < 0.001	**
		Stress X Target interaction		F(1, 28) = 4.478	*p* = 0.043	*

	Holm-sidak post-hoc analysis			within non-stressed, effect of target, ***p* < 0.001; within stressed, effect of target, *p* = 0.080; within non-target, effect of stress, *p* = 0.949, within target, effect of stress, **p* = 0.014		
Figure 3g	two-way RM ANOVA	Group: non-stress vs. stress	non-stress = 11, stress = 15	F(1, 24) = 1.968	*p* = 0.173	NS
		Repeated trial: No Target vs. Target		F(1, 24) = 58.986	*p* < 0.001	**
		Stress X Target interaction		F(1, 24) = 2.480	*p* = 0.128	NS
	Holm-sidak post-hoc analysis for main target effect			within non-stressed, effect of target, **p <0.001; within stressed, effect of target, **p <0.001		
Figure 4c left	two-way RM ANOVA	Group: non-stress vs. stress	non-stress = 8, stress = 11	F(1, 17) = 4.008	*p* = 0.061	NS
		Repeated trial: No Target vs. Target		F(1, 17) = 0.00147	*p* = 0.970	NS
		Stress X Target interaction		F(1, 17) = 17.763	*p* < 0.001	**
	Holm-sidak post-hoc analysis			within non-stress, effect of target, **p* = 0.012, within stress the effect of target, ***p* = 0.005, within target presence, effect of stress, ***p* < 0.001		
Figure 4c right	two-way RM ANOVA	Group: non-stress vs. stress	non-stress = 10, stress = 11	F(1, 19) = 3.371	*p* = 0.082	NS
		Repeated trial: No Target vs. Target		F (1, 19) = 18.637	*p* < 0.001	**
		Stress X Target interaction		F(1, 19) = 3.965	*p* = 0.061	NS
	Holm-sidak post-hoc analysis for main target effect			within non-stressed, effect of target *p* = 0.125, within stressed, effect of target, ***p* < 0.001		
Figure 4d left	two-way RM ANOVA	Group: control vs. R-CTGF	control = 8, R-CTGF = 10	F(1, 16) = 1.266	*p* = 0.277	NS
		Repeated trial: No Target vs. Target		F(1, 16) = 0.143	*p* = 0.710	NS

		Stress X Target interaction		$F(1, 16) = 8.921$	$p = 0.009$	**
	Holm-sidak post-hoc analysis			within control, effect of target, *$p = 0.038$, within R-CTGF, effect of target, $p = 0.068$, within target presence, the effect of R-CTGF, *$p = 0.025$		
Figure 4d right	independent samples t-test	Cntrol vs. R-CTGF	control = 8, R-CTGF = 10	$t_{16} = 3.020$	$p = 0.00814$	**
Figure 4e left	Mann-Whitney Rank Sum Test	Control vs. R-CTGF	control = 5, R-CTGF = 9	T = 54	$p = 0.033$	*
Figure 4e right	independent samples t-test	Control vs. R-CTGF	control = 5, R-CTGF = 9	$t_{12} = -2.547$	$p = 0.0256$	*
Figure 5b	independent samples t-test	Control vs. L-CTGF	control = 4, L-CTGF = 4	$t_6 = -6.142$	$p < 0.01$	**
Figure 5c left	two-way RM ANOVA	Group: non-stress vs. stress	non-stress = 13, stress = 21	$F(1, 32) = 2.517$	$p = 0.122$	NS
		Repeated trial: No Target vs. Target		$F(1, 32) = 29.886$	$p < 0.001$	**
		Stress X Target interaction		$F(1, 32) = 9.537$	$p = 0.004$	**
	Holm-sidak post-hoc analysis			within non-stressed, the effect of target, **$p < 0.001$; within stressed, the effect of target, $p = 0.063$; within target presence, effect of stress, **$p = 0.004$		
Figure 5c right	two-way RM ANOVA	Group: non-stress vs. stress	non-stress = 14, stress = 15	$F(1, 27) = 0.0540$	$p = 0.818$	NS
		Repeated trial: No Target vs. Target		$F(1, 27) = 20.560$	$p < 0.001$	**
		Stress X Target interaction		$F(1, 27) = 1.293$	$p = 0.266$	NS
	Holm-sidak post-hoc analysis for main target effect			within L-CTGF non-stressed, the effect of target,**$p < 0.001$; within L-CTGF stressed, the effect of target, *$p = 0.021$		
Figure 5d left	independent samples t-test	Control vs. L-CTGF	control = 6, L-CTGF = 8	$t_{12} = 1.728$	$p = 0.110$	NS
Figure 5d right	independent samples t-test	Control vs. L-CTGF	control = 6, L-CTGF = 8	$t_{12} = 0.315$	$p = 0.758$	NS
